# Divergent asymmetric synthesis of (–)-ganocins A–C and (+)-cochlearol B via a non-biomimetic strategy

**DOI:** 10.1038/s41467-026-78287-1

**Published:** 2026-10-15

**Authors:** Hailong Xu, Yu Yang, Jinghua Wu, Huanfeng Jiang, Zhiqiang Ma

**Affiliations:** 1 Key Lab of Functional Molecular Engineering of Guangdong Province, School of Chemistry & Chemical Engineering, South China University of Technology, Guangzhou, PR China; 2 State Key Laboratory of Natural and Biomimetic Drugs, Peking University, Beijing, PR China; 3 School of Chemical Science and Technology, Yunnan University, Kunming, PR China

**Keywords:** Natural product synthesis, Synthetic chemistry methodology, Asymmetric synthesis

## Abstract

Natural products are of substantial value in drug discovery screening campaigns. Their three-dimensional structures exhibit high complementarity with target proteins, via a multimodal combination of hydrogen bonds, hydrophobic contacts, electrostatic interactions, and van der Waals interactions. Skeletal diversity directly determines the three-dimensional shape diversity of compound libraries. Therefore, the rapid access to distinct molecular skeletons significantly increases the odds of drug discovery. Ganocins A–C and cochlearol B, *Ganoderma* meroterpenoids, feature distinct skeletons and diverse bioactivities. Despite tremendous efforts by synthetic chemists, a unified asymmetric synthetic strategy towards all four members has remained elusive. Here, we report the unified, asymmetric total synthesis of (–)-ganocins A–C and (+)-cochlearol B via a non-biomimetic strategy, featuring diastereoselective oxa-Michael addition, acid-controlled divergent cascade aldol/etherification cyclization or aldol/dehydration, and SmI_2_-mediated 4-*exo*-trig reductive cross-coupling. This meticulously designed synthetic strategy establishes a general solution to a problem previously deemed intractable by biomimetic approaches.

## Abstract (web summary)

*Ganoderma* meroterpenoids, ganocins A–C and cochlearol B, feature distinct skeletons and diverse bioactivities but a unified asymmetric synthetic strategy towards all four members has remained elusive. Here, the authors report the unified, asymmetric total synthesis of (–)-ganocins A–C and (+)-cochlearol B via a non-biomimetic strategy.

## Introduction

Natural products have long been invaluable sources for the discovery of therapeutic drugs^1,2^. Fifty-eight natural product-related drugs were approved globally from January 2014 to June 2025^3^. The three-dimensional structures of natural products enable a high degree of complementarity with target proteins through a sophisticated interplay of precise structural recognition and modulation of protein conformation^4,5^. This relies on a multimodal combination of hydrogen bonds, hydrophobic contacts, electrostatic interactions, and van der Waals interactions, ensuring high affinity and specificity. Skeletal diversity primarily determines the three-dimensional shape diversity of a compound library^6,7^. Thus, the rapid and efficient construction of distinct molecular skeletons substantially increases the odds of drug discovery compared with a single-scaffold library.

**Fig. 1:**
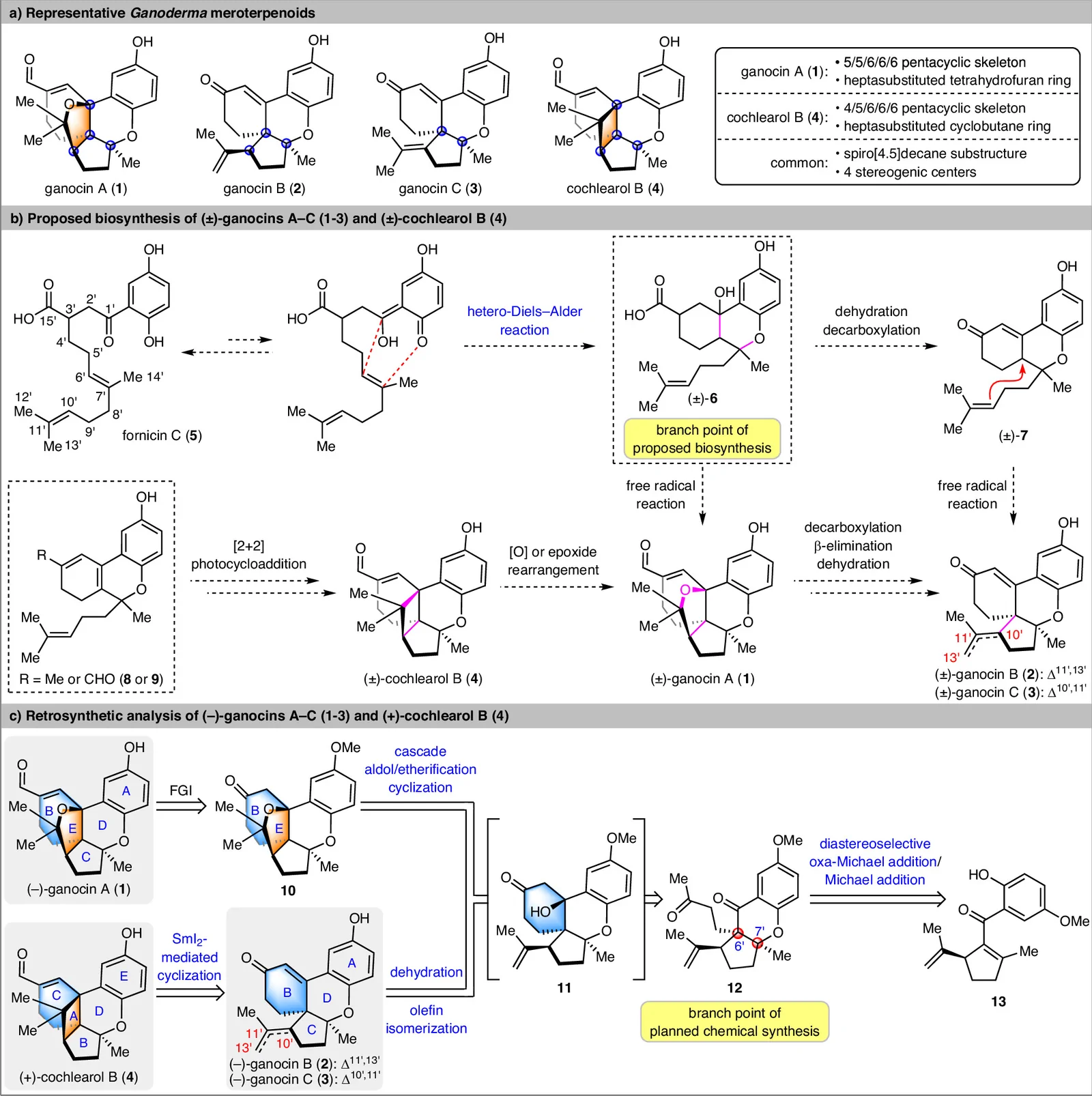
*Ganoderma* meroterpenoid natural products. **a** Representative *Ganoderma* meroterpenoids: ganocins A–C (**1**−**3**) and cochlearol B (**4**). **b** Proposed biosynthesis of (±)-ganocins A–C (**1**−**3**) and (±)-cochlearol B (**4**). **c** Retrosynthetic analysis of (–)-ganocins A–C (**1**−**3**) and (+)-cochlearol B (**4**).

*Ganoderma*, a medicinal fungus used for centuries to treat various diseases, has been studied extensively in China, Korea, Japan, and the United States of America^8^. To date, a variety of natural products, including meroterpenoids, triterpenoids, polysaccharides, sesquiterpenoids, steroids, and alkaloids, have been isolated from the genus *Ganoderma*^9^. Among these, *Ganoderma* meroterpenoids are structurally characterized by a 1,2,4-trisubstituted phenyl group and the unsaturated terpene subunit, exhibiting diverse biological activities, such as antitumor, antifibrotic, antiallergic, antioxidant, and antimicrobial activities^10–13^. Their distinct molecular skeletons and diverse biological activities have attracted considerable attention from both pharmacological and synthetic communities. Ganocins A–C (**1**−**3**) are three *Ganoderma* meroterpenoids that were isolated from the fruiting bodies of *Ganoderma cochlear* in racemic form by Qiu and co-workers in 2014 (Fig. 1a)^14^. In the same year, cochlearol B (**4**) was also isolated from *Ganoderma cochlear* in racemic form by Cheng and co-workers^15^. Notably, the enantiomers (+)- and (–)-cochlearol B (**4** and *ent*-**4**), obtained via chiral HPLC separation, exhibited significantly distinct inhibitory effects against phosphorylated Smad2 and Smad3 (p-Smads). Therefore, developing asymmetric synthetic strategies for this class of *Ganoderma* meroterpenoids is of critical importance.

Structurally, ganocins A–C (**1**−**3**) and cochlearol B (**4**) share a 5/6/6/6 tetracyclic scaffold incorporating a spiro[4.5]decane moiety (Fig. 1a). Specifically, ganocin A (**1**) features a heptasubstituted tetrahydrofuran ring (highlighted in orange) and four contiguous stereocenters, including three quaternary stereocenters—two embedded within the tetrahydrofuran ring. Cochlearol B (**4**) is characterized by a *gem*-dimethylcyclobutane core (highlighted in orange) bearing two contiguous all-carbon quaternary stereocenters.

Biosynthetically, Qiu et al. proposed that (±)-ganocins A–C (**1**−**3**) are derived from fornicin C (**5**)^14^ (Fig. 1b). In their pathway, a hetero-Diels−Alder (HDA) reaction of **5** initially affords racemic chromane **6**. Subsequent radical cyclization yields (±)-ganocin A (**1**). Alternatively, dehydration and decarboxylation of intermediate **6** lead to enone **7**, whose radical cyclization furnishes both (±)-ganocins B (**2**) and C (**3**). Meanwhile, on the basis of structural similarities between (±)-ganocins A–C (**1**−**3**) and (±)-cochlearol B (**4**), an alternative biosynthetic pathway has been proposed by Hao’s group^16^ and by Li and Tang’s group^17^. Their hypothesis begins with an intramolecular [2 + 2] photocycloaddition of chromene **8** or **9**, affording (±)-cochlearol B (**4**). Subsequent oxidative cleavage of the cyclobutane ring or epoxide rearrangement directly affords (±)-ganocin A (**1**). This intermediate then undergoes sequential decarboxylation, β-elimination, and dehydration to furnish (±)-ganocins B (**2**) and C (**3**). In the biogenetic hypothesis for ganocins A–C and cochlearol B, the racemic chromane **6**, which is generated via a HDA reaction, defines the branch point of biosynthesis. When multiple congeners of a natural product family are targeted for chemical synthesis, the most efficient strategy is typically the preparation of a versatile late-stage intermediate, whose diversification enables access to the entire collection. In this context, mimicking biosynthetic pathways has proven to be an effective strategy for constructing complex natural products.

To date, eight elegant total syntheses have been reported^16–24^, most of which focus on individual natural products; six of these are racemic, and the majority are biomimetic (see Supplementary Fig. 1 for details). In the current biomimetic syntheses of ganocins A–C and cochlearol B, the key chromane moieties were typically constructed via a biomimetic HDA reaction. Since this HDA reaction inherently generates racemic intermediates, the biomimetic chemical synthesis ultimately affords the target natural products as racemates. The scarcity of asymmetric and divergent synthetic approaches has substantially hindered rigorous biological evaluation of these natural products. To overcome this long-standing challenge, the identification of a non-biomimetic intermediate for asymmetric synthesis is required. Herein, we report that chiral chromanone **12**—a versatile non-biomimetic intermediate—serves as a branch point in our chemical synthesis (Fig. 1c). Leveraging this intermediate, we have accomplished the divergent asymmetric total synthesis of (–)-ganocins A–C (**1**−**3**) and (+)-cochlearol B (**4**) from (*R*)-limonene in 11, 8, 8, and 15 steps, respectively. Notably, the 11-step synthesis of (–)-ganocin A (**1**) represents the shortest asymmetric synthetic route to date—a significant reduction from the previously reported 17-step route. This streamlined synthetic platform provides a foundation for further rigorous biological evaluation.

## Results

### Retrosynthetic analysis

Skeletal-diversity-oriented synthesis critically relies on the strategic identification and rapid assembly of a versatile common intermediate bearing multiple functional groups. We envisioned that compound **12** (Fig. 1c), with its strategically positioned functionalities, would serve as this pivotal intermediate. Specifically, (–)-ganocin A (**1**) could be synthesized from pentacyclic intermediate **10** through functional group interconversions (FGIs) (Fig. 1c). Intermediate **10** would itself accessible from **12** via a one-pot cascade reaction to construct the B and E rings^25–33^, involving an intramolecular aldol reaction of **12** to give **11**, followed by etherification between the tertiary alcohol and the pendant isopropenyl group. Furthermore, we hypothesized that SmI_2_-mediated cyclization of the enone group in (–)-ganocin B (**2**) could form the strained cyclobutane (A ring) of (+)-cochlearol B (**4**). The B ring of (–)-ganocin B (**2**) would be constructed directly from intermediate **12** via intramolecular aldol reaction/dehydration. Subsequent olefin isomerization would then provide (–)-ganocin C (**3**). Finally, compound **12** could be synthesized from enone **13** through a diastereoselective oxa-Michael/Michael addition sequence.

The synthetic strategy encounters significant challenges: (1) condition-controlled pathway differentiation is required to direct the common intermediate **12** toward two distinct molecular skeletons. This demands precise manipulation of its reactivity under orthogonal conditions; (2) implementing an aldol/etherification cascade cyclization to reduce synthetic steps remains an adventurous approach, as such transformations are scarcely documented in natural product synthesis and necessitate extensive optimization; (3) the construction of the heptasubstituted cyclobutane motif in (+)-cochlearol B (**4**) via non-[2 + 2] cycloaddition approaches poses a persistent synthetic challenge due to the inherent ring strain.

### Rapid construction of the versatile common intermediate 12

**Fig. 2:**
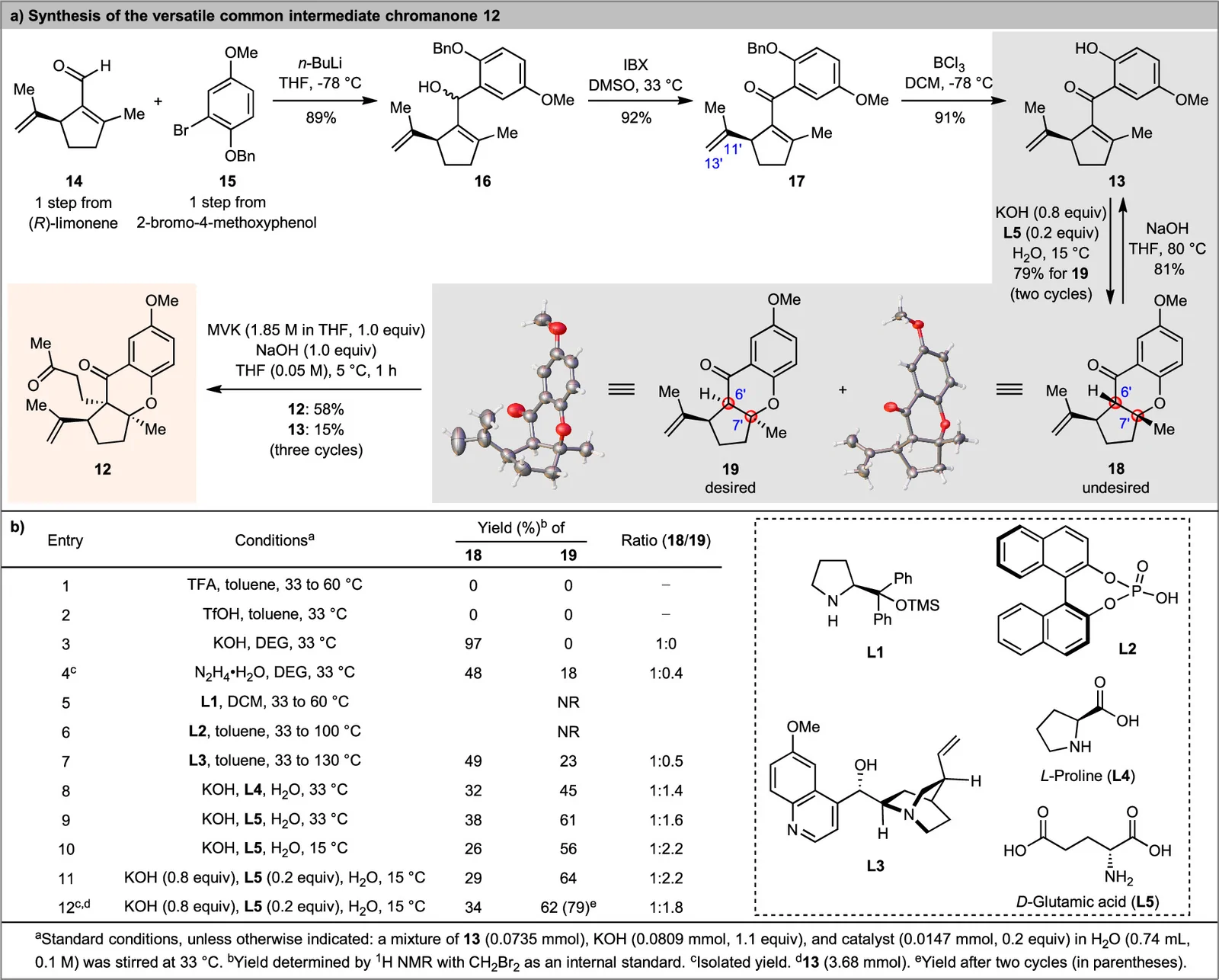
Rapid construction of the versatile common intermediate chromanone **12**. **a** Synthesis of the versatile common intermediate chromanone **12**. **b** Investigation of the oxa-Michael addition of **13**. Bn benzyl, THF tetrahydrofuran, IBX 2-iodoxybenzoic acid, DMSO dimethyl sulfoxide, DCM dichloromethane, TFA trifluoroacetic acid, Tf trifluoromethanesulfonyl, DEG diethylene glycol, N_2_H_4_·H_2_O hydrazine hydrate, MVK methyl vinyl ketone.

Our synthetic efforts commenced with the preparation of common intermediate **12** (Fig. 2a). Beginning with known aldehyde **14** (available from (*R*)-limonene in one step)^34^ and benzyl ether **15** (available from 2-bromo-4-methoxyphenol in one step)^35^, the union of the two fragments by 1,2-addition, wherein fragment **15** first undergoes lithium halogen exchange with *n*-BuLi, followed by addition of aldehyde **14**, afforded secondary alcohol **16** in excellent yield as an inconsequential mixture of epimers. Oxidation of alcohol **16** with 2-iodoxybenzoic acid (IBX) furnished enone **17** in good yield. Subsequent treatment of **17** with BCl_3_ in dichloromethane (DCM) at −78 °C resulted in smooth and selective debenzylation, delivering phenol **13** in 91% yield. Notably, it was observed that the C11′−C13′ double bond was prone to hydration under some acidic deprotection conditions (see Supplementary Table 1).

We next focused our attention on building the tetrahydropyran moiety with contiguous stereocenters at C6′ and C7′ by employing a diastereoselective oxa-Michael addition^36–40^. However, this transformation proved challenging. Initially, treatment of **13** with trifluoroacetic acid (TFA) and trifluoromethanesulfonic acid (TfOH) led to decomposition or undesired side products (see Fig. 2b, entries 1 and 2, for a complete list of conditions used; see Supplementary Table 2). Unexpectedly, exposing **13** to KOH afforded the undesired diastereomer **18** in 97% yield (entry 3). The structure of **18** was confirmed by X-ray crystallography (Deposition numbers 2492584 (for **18**), 2492585 (for **19**), 2492583 (for **10**), 2492586 (for **21**), and 2492587 (for **27**) contain the supplementary crystallographic data for this paper. These data are provided free of charge by the joint Cambridge Crystallographic Data Centre and Fachinformationszentrum Karlsruhe Access Structures service www.ccdc.cam.ac.uk/structures). This observed stereochemistry could be rationalized by the presence of a β-oriented isopropenyl group, which directs the phenol group in **13** to approach from the less sterically hindered α-face. Inspired by the work of She^35,41^, subjecting **13** to hydrazine hydrate (N_2_H_4_·H_2_O) in diethylene glycol (DEG) delightfully afforded **18** and the desired product **19**, for the first time, in a low yield (18%) (entry 4). The structure and absolute configuration of **19** were unambiguously confirmed by X-ray crystallography (Deposition numbers 2492584 (for **18**), 2492585 (for **19**), 2492583 (for **10**), 2492586 (for **21**), and 2492587 (for **27**) contain the supplementary crystallographic datafor this paper. These data are provided free of charge by the joint Cambridge Crystallographic Data Centre andFachinformationszentrum Karlsruhe Access Structures service www.ccdc.cam.ac.uk/structures). Although formed as a minor product, this encouraging result prompted us to further optimize the reaction conditions. Further screening of conditions—including reagents (PhNHNH_2_, PhNH_2_), temperature variations, and solvents—did not significantly improve the yield of **19**. Additionally, we screened many types of chiral organocatalysts, including prolinol analogues, phosphoric acids, and cinchona alkaloids. Unfortunately, catalysts **L1** and **L2** proved ineffective, yielding none of the desired product (entries 5 and 6). The use of catalyst **L3** still enabled the formation of product **19** as the minor product (entry 7). On the basis of these results, and inspired by the work of Tanaka^42^, Zheng^43^, and Porto^44^, we next screened various chiral amino acid catalysts with KOH in H_2_O at 33 °C. To our delight, the use of *L*-proline (**L4**) led to the formation of compound **19** as the major product (dr = 1:1.4, entry 8). The ratio was slightly increased by employing *D*-glutamic acid (**L5**) (entry 9). Consequently, through systematic optimization of reaction parameters, we improved the diastereomeric ratio of **18**/**19** to 1:2.2 and the yield of **19** to 64% (entries 10 and 11). Scaling up the reaction to gram scale maintained comparable yield (62%; entry 12), demonstrating the robustness of the reaction. Furthermore, the undesired tricycle **18** could undergo retro-Michael reaction in NaOH/tetrahydrofuran (THF) to regenerate phenol **13** in 81% yield, which was suitable for the oxa-Michael process again. As a result, the desired tricycle **19** was obtained in 79% overall yield after two cycles (entry 12). Mechanistically, we propose that the ketone in **13** condenses with *D*-glutamic acid to form an imine intermediate. The β-oriented isopropenyl group induces steric shielding of the α*-*face by the imine-bound side chain, thereby directing the phenol nucleophile to attack from the β-face^35^, ultimately affording the correct C7′ stereochemistry.

With tricycle **19** in hand, our attention turned to a Michael addition reaction to install the side chain (Fig. 2a). To this end, we attempted a range of Michael addition conditions for **19** and methyl vinyl ketone (MVK) (Supplementary Tables 3–5)^18^. Under various acid or base conditions, attempts either led to olefin isomerization, recovery of starting material, or retro-oxa-Michael reactions. Ultimately, treatment of **19** with NaOH in THF at 5 °C provided diketone **12** as a single diastereomer in 58% yield, along with retro-oxa-Michael product **13** in 15% yield (after three cycles with reisolation of starting material).

### Acid-dependent divergent reaction pathways

**Fig. 3:**
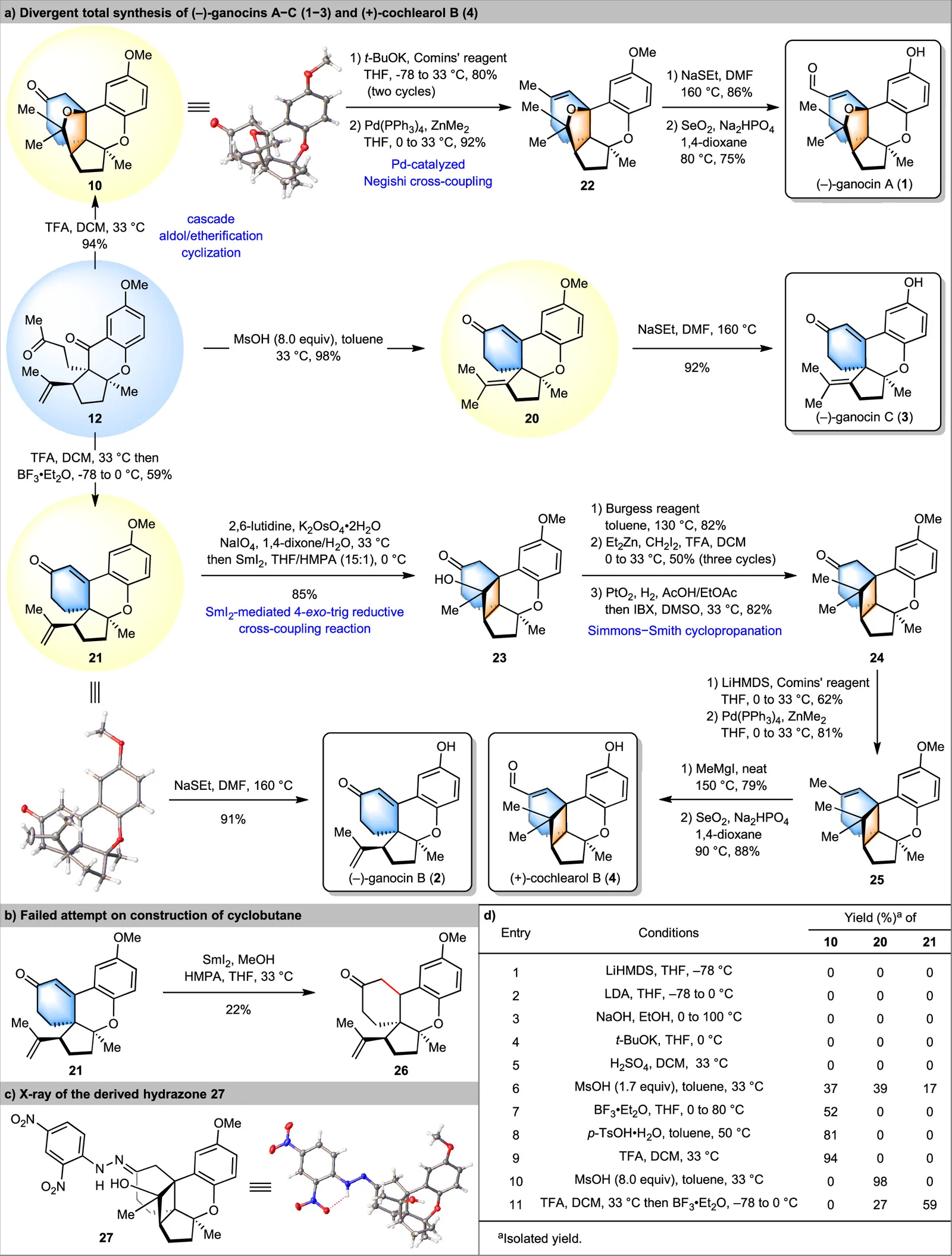
Completion of the synthesis. **a** Divergent total synthesis of (–)-ganocins A−C (**1**−**3**) and (+)-cochlearol B (**4**). **b** Failed attempt on construction of cyclobutane. **c** X-ray of the derived hydrazone **27**. **d** Acid-dependent divergent reaction pathways. LiHMDS lithium bis(trimethylsilyl)amide, LDA lithium diisopropylamide, Ms methanesulfonyl, Ts toluenesulfonyl, DMF *N*,*N*-dimethylformamide, HMPA hexamethylphosphoramide.

Having successfully synthesized key common intermediate **12**, we proceeded to explore its application in aldol reaction. Notably, during the debenzylation study of compound **17**, we observed an unexpected acid-catalyzed hydration of the olefin moiety. This serendipitous finding prompted us to hypothesize the feasibility of a tandem aldol-etherification reaction^45–51^. Specifically, we proposed that diketone **12** undergoes an acid-mediated intramolecular aldol reaction to generate a six-membered ring intermediate **11** (Fig. 1c), which features a tertiary alcohol. Under the same acidic conditions, this tertiary alcohol could engage in an intramolecular nucleophilic attack on the isopropenyl group to result in an etherification cyclization. This transformation would construct the 5/5/6/6/6 pentacyclic skeleton bearing a highly substituted tetrahydrofuran core. Furthermore, we hypothesized that under alternative conditions, this tertiary alcohol could undergo dehydration to form an α,β-unsaturated ketone, thereby furnishing the 5/6/6/6 tetracyclic framework. Systematic screening of reaction conditions (Fig. 3a, d, and see Supplementary Table 6 for details) revealed that initial treatment of diketone **12** under basic conditions (LiHMDS, LDA, NaOH, *t*-BuOK) failed to induce any transformation (entries 1−4). Subsequently, treatment of **12** with H_2_SO_4_ did not yield the desired products (entry 5). Notably, treatment of **12** with methanesulfonic acid (MsOH) in toluene generated three distinct products through competitive pathways: the pentacyclic compound **10** (37% yield) via a tandem aldol-etherification sequence; the tandem aldol-dehydration product **21** (17%); and the olefin isomerization derivative **20** (39%), formed by further isomerization of **21** (entry 6). The simultaneous formation of these valuable intermediates prompted us to systematically optimize conditions for selectively accessing each product as the major component. Specifically, Treatment of **12** with BF_3_·Et_2_O in THF afforded compound **10** in only 52% yield, while *p*-toluenesulfonic acid monohydrate (*p*-TsOH·H_2_O) significantly increased the yield to 81% (entries 7 and 8). Ultimately, TFA/DCM achieved **10** in 94% yield (entry 9). To favor **20** formation, increasing MsOH to 8.0 equivalents resulted in 98% yield of **20** (entry 10). Furthermore, sequential treatment of **12** with TFA in DCM and then BF_3_·Et_2_O afforded **21** as the major product in 59% yield (entry 11). The structures of compounds **10** and **21** were confirmed by X-ray crystallography (Deposition numbers 2492584 (for **18**), 2492585 (for **19**), 2492583 (for **10**), 2492586 (for **21**), and 2492587 (for **27**) contain the supplementary crystallographic datafor this paper. These data are provided free of charge by the joint Cambridge Crystallographic Data Centre andFachinformationszentrum Karlsruhe Access Structures service www.ccdc.cam.ac.uk/structures). Collectively, these optimized conditions demonstrate precise control over divergent pathways from **12**, enabling selective skeletal diversification.

Of note, in contrast to the findings of Wang and co-workers in analogous aldol reaction studies^18^, our substrate **12**, featuring a methyl ether protecting group, yielded a distinctly different outcome under acidic conditions. While their substrate, protected as a *tert*-butyldimethylsilyl (TBS) ether, afforded exclusively the aldol condensation/TBS deprotection product^18^, our substrate **12** produced the tandem aldol-etherification product **10**. We tentatively attribute the distinct outcome to the pronounced steric bulk of the TBS group, which restricts the substrate’s conformational flexibility and thereby redirects the reaction pathway instead of etherification.

### Total syntheses of (–)-ganocins A–C and (+)-cochlearol B

With **10,** **20**, and **21** in hand, efforts were focused on the installation of the remaining functionalities toward the divergent total synthesis of (–)-ganocins A–C (**1**−**3**) and (+)-cochlearol B (Fig. 3a). For this purpose, regioselective deprotonation of ketone **10** with *t*-BuOK followed by enolate capture with Comins’ reagent generated the vinyl triflate in 80% overall yield after two cycles, which was then exposed to Negishi cross-coupling conditions^52^ to afford the trisubstituted alkene **22** in 92% yield. Subjecting **22** to NaSEt in DMF at 160 °C afforded the phenol in 86% yield^19^. Ultimately, regio-selective allylic oxidation^53^ completed the asymmetric synthesis of (–)-ganocin A (**1**) in only 11 steps from (*R*)-limonene, representing the shortest asymmetric synthetic route to date—a 6-step reduction compared to the previously reported 17-step approach^22^. With respect to synthesis of (–)-ganocins B and C (**2** and **3**), treatment of **20** with NaSEt^19^ in DMF at 160 °C afforded (–)-ganocin C (**3**) in 92% yield, while **21** gave (–)-ganocin B (**2**) in 91% yield under identical conditions, completing the asymmetric total syntheses of these two natural products. This concise sequence underscores the high synthetic efficiency enabled by our strategically designed tandem transformations.

At this stage, the primary objective was the synthesis of (+)-cochlearol B (**4**). The construction of the highly strained cyclobutane ring posed a significant synthetic challenge^54–57^. Guided by the retrosynthetic analysis (Fig. 1c), we initially pursued SmI_2_-mediated cyclization to assemble the cyclobutane ring. We envisioned that initiating the radical from the enone could achieve regioselective benzylic radical generation, which would cyclize with the isopropenyl group. Specifically, subjecting **21** to SmI_2_ conditions^58–62^ was expected to afford **24** directly. However, various conditions were examined, yet only ketone **26** was obtained (Fig. 3b). Further attempts by adding radical-trapping reagents^63–65^—including 4-bromobenzenethiol, 4-mercaptophenol, CCl_4_, and BrCCl_3_—proved unsuccessful (Supplementary Table 7). These results underscore the inherent synthetic challenges in constructing the strained cyclobutane ring.

Given the undesired outcome and comprehensive literature analysis, we strategically converted the isopropenyl moiety in **21** to a ketone group. We envisioned that this modification would enable a SmI_2_-mediated 4-*exo*-trig reductive cross-coupling reaction between the newly formed ketone and cyclic enone motifs in **21**. After optimization (Supplementary Table 8), oxidative cleavage of the alkene in **21** was achieved using 2,6-lutidine, potassium osmate dihydrate (K_2_OsO_4_·2H_2_O), and sodium periodate (NaIO_4_). Subsequent SmI_2_-mediated 4-*exo*-trig reductive cyclization efficiently forged the strained cyclobutanol **23** in 85% yield, representing a notable achievement given the challenges encountered in constructing four-membered ring. Its structure and absolute configuration were unambiguously confirmed by X-ray crystallography of the derived hydrazone **27** (Fig. 3c) (Deposition numbers 2492584 (for **18**), 2492585(for **19**), 2492583 (for **10**), 2492586 (for **21**), and 2492587 (for **27**) contain the supplementary crystallographic datafor this paper. These data are provided free of charge by the joint Cambridge Crystallographic Data Centre andFachinformationszentrum Karlsruhe Access Structures service www.ccdc.cam.ac.uk/structures). Additionally, we explored alternative strategies including Michael addition of the aldehyde and the enone, titanium(III)-mediated radical cyclization of an epoxide^66^, Oppolzer cyclization^67^, and photocatalytic reactions induced by the super photoreductant CBZ6^68,69^ (Supplementary Fig. 2). However, all these approaches were ineffective. Subsequently, cyclobutanol **23** was converted to **24** via a three-step sequence involving dehydration, modified Simmons−Smith cyclopropanation, and hydrogenation/oxidation. The total synthesis of (+)-cochlearol B (**4**) was then achieved from **24** through sequential regioselective triflation, Negishi cross-coupling to afford **25**, followed by demethylation^17^ and allylic oxidation. Notably, we optimized the allylic oxidation conditions of Li and Tang’s group^17^ by adding Na_2_HPO_4_, increasing the yield from 60 to 88%. The spectroscopic data of synthetic (+)-cochlearol B (**4**) were in agreement with those of natural **4**^15^.

## Discussion

In summary, we have accomplished the divergent asymmetric total synthesis of the *Ganoderma* meroterpenoids (–)-ganocins A–C (**1**−**3**) and (+)-cochlearol B (**4**) from (*R*)-limonene in 11, 8, 8, and 15 steps, respectively. The synthesis relied on a diastereoselective amino acid-catalyzed oxa-Michael addition to construct the chromanone framework and install the C7′ quaternary stereocenter. A common intermediate **12** was divergently elaborated to either the 5/5/6/6/6 pentacyclic scaffold via an acid-mediated cascade aldol/etherification cyclization or the 5/6/6/6 tetracyclic framework via an aldol condensation. Moreover, a SmI_2_-mediated 4-*exo*-trig reductive cross-coupling reaction between ketone and cyclic enone motifs efficiently forged the highly strained and substituted cyclobutanol ring. Our strategy overcomes the inherent limitations of biomimetic approaches and achieves a unified asymmetric total synthesis of all four natural products within this family. We envision that the developed methods, particularly the cascade aldol/etherification cyclization, will establish a general synthetic platform for accessing other natural products with complex frameworks, such as sinulochmodin C and entangolensins C and D.

## Methods

### General

All reactions were carried out under a nitrogen atmosphere anhydrous conditions, and all reagents were purchased from commercial suppliers without further purification. Unless stated otherwise, reactions were performed at 33 °C. Anhydrous THF (tetrahydrofuran), DCM (dichloromethane), PhMe (toluene), and DMF (*N,N*-dimethylformamide) were obtained from an Inert PS-MD-4 solvent system. Anhydrous CH_3_CN (acetonitrile), benzene, PhCl (chlorobenzene), DMSO (dimethyl sulfoxide), MeOH (methanol), EtOH (ethanol), *t*-BuOH (*tert*-butanol), *i*-PrOH (isopropanol), EtOAc (ethyl acetate), and 1,4-dioxane were purchased from Energy Chemical, Ltd. HMPA (hexamethylphosphoramide), Et_3_N (triethylamine), and Et_2_O (diethyl ether) were obtained from commercial sources and subjected to anhydrous treatment prior to use. AcOH (acetic acid) was commercially available and was used without anhydrous treatment. ^1^H NMR and ^13^C NMR spectra were recorded on a Bruker Avance 400 MHz NMR spectrometer (400 MHz for ^1^H, 100 MHz for ^13^C) or a Bruker Avance 500 MHz NMR spectrometer (500 MHz for ^1^H, 125 MHz for ^13^C). Chemical shifts were referenced using residual undeuterated solvent (CDCl_3_: δ 7.26, 77.16 ppm; C_6_D_6_: δ 7.16, 128.06 ppm; C_5_D_5_N: 7.19, 149.0 ppm; (CD_3_)_2_CO: δ 2.05, 29.84 ppm unless otherwise stated) at 298 K. HRMS(ESI) spectra were acquired on a Waters Xevo G3 QTof mass spectrometer. IR spectra were recorded on a Nicolet 210 spectrophotometer. Melting points were determined using a hot stage apparatus. Optical rotations were measured with a Rudolph Research Automatic Polarimeter IV. Single-crystal structures were measured on a SuperNova Dual, Cu at zero, AtlasS2 diffractometer. TLC was performed on commercially prepared silica gel plates (GF254, 200–300 mesh) and visualized under UV light at 254 nm or by staining with phosphomolybdic acid or potassium permanganate solution.

### Detailed experimental procedures

The detailed experimental procedures were provided in Supplementary Information.

## Supplementary information

Supplementary Information

Transparent Peer Review File

## Data Availability

All relevant data supporting the findings of this study, including experimental procedures and compound characterizations, are available within the Supplementary Information. The X-ray crystallographic coordinates for structures **18**, **19**, **10**, **21**, and **27** reported in this study have been deposited at the Cambridge Crystallographic Data Centre (CCDC) with the accession codes CCDC 2492584, CCDC 2492585, CCDC 2492583, CCDC 2492586, and CCDC 2492587 (www.ccdc.cam.ac.uk/data_request/cif), respectively. All data are available from the corresponding author upon request.

## References

[1] Newman, D. J. & Cragg, G. M. Natural products as sources of new drugs over the nearly four decades from 01/1981 to 09/2019. *J. Nat. Prod.* **83**, 770–803 (2020). 10.1021/acs.jnatprod.9b01285 32162523

[2] Rodrigues, T., Reker, D., Schneider, P. & Schneider, G. Counting on natural products for drug design. *Nat. Chem.* **8**, 531–541 (2016). 10.1038/nchem.2479 27219696

[3] Butler, M. S., Capon, R. J., Blaskovich, M. A. T. & Henderson, I. R. Natural product-derived compounds in clinical trials and drug approvals. *Nat. Prod. Rep.* **43**, 20–88 (2026). 10.1039/d5np00031a 40923383

[4] Talele, T. T. Opportunities for tapping into three-dimensional chemical space through a quaternary carbon. *J. Med. Chem.* **63**, 13291–13315 (2020). 10.1021/acs.jmedchem.0c00829 32805118

[5] Choi, Y. et al. Natural product-derived drugs: structural insights into their biological mechanisms. *Biomolecules* **15**, 1303 (2025). 10.3390/biom15091303 PMC12467776 41008610

[6] O’ Connor, C. J., Beckmann, H. S. G. & Spring, D. R. Diversity-oriented synthesis: producing chemical tools for dissecting biology. *Chem. Soc. Rev.* **41**, 4444–4456 (2012). 10.1039/c2cs35023h 22491328

[7] Galloway, W. R. J. D., Isidro-Llobet, A. & Spring, D. R. Diversity-oriented synthesis as a tool for the discovery of novel biologically active small molecules. *Nat. Commun.* **1**, 80 (2010). 10.1038/ncomms1081 20865796

[8] Paterson, R. R. M. *Ganoderma*—a therapeutic fungal biofactory. *Phytochemistry* **67**, 1985–2001 (2006). 10.1016/j.phytochem.2006.07.004 16905165

[9] Wang, L. et al. Traditional uses, chemical components and pharmacological activities of the genus *Ganoderma* P. Karst.: a review. *RSC Adv.* **10**, 42084–42097 (2020). 10.1039/d0ra07219b PMC9057998 35516772

[10] Peng, X. & Qiu, M. Meroterpenoids from *Ganoderma* species: a review of last five years. *Nat. Prod. Bioprospect.* **8**, 137–149 (2018). 10.1007/s13659-018-0164-z PMC5971034 29722004

[11] Zhao, M., Tang, Y., Xie, J., Zhao, Z. & Cui, H. Meroterpenoids produced by fungi: occurrence, structural diversity, biological activities, and their molecular targets. *Eur. J. Med. Chem.* **209**, 112860 (2021). 10.1016/j.ejmech.2020.112860 33032085

[12] Peng, X.-R., Unsicker, S. B., Gershenzon, J. & Qiu, M.-H. Structural diversity, hypothetical biosynthesis, chemical synthesis, and biological activity of *Ganoderma* meroterpenoids. *Nat. Prod. Rep.* **40**, 1354–1392 (2023). 10.1039/d3np00006k 37051770

[13] Zhang, J.-J., Qin, F.-Y. & Cheng, Y.-X. Insights into *Ganoderma* fungi meroterpenoids opening a new era of racemic natural products in mushrooms. *Med. Res. Rev.* **44**, 1221–1266 (2024). 10.1002/med.22006 38204140

[14] Peng, X.-R. et al. Four new polycyclic meroterpenoids from *Ganoderma cochlear*. *Org. Lett.* **16**, 5262–5265 (2014). 10.1021/ol5023189 25192355

[15] Dou, M. et al. Cochlearols A and B, polycyclic meroterpenoids from the fungus *Ganoderma cochlear* that have renoprotective activities. *Org. Lett.* **16**, 6064–6067 (2014). 10.1021/ol502806j 25402560

[16] Gao, Z. et al. A unified and bio-inspired total syntheses of cochlearol B and ganocins A-C via a cyclobutane as overbred intermediate. Preprint at 10.26434/chemrxiv-2022-xxmgl (2022).

[17] Wang, Z. et al. Bioinspired synthesis of cochlearol B and ganocin A. *Chin. Chem. Lett.* **35**, 109247 (2024).

[18] Liu, Y., Zhou, C.-J., Li, Q. & Wang, H. Total synthesis of (±)-ganocins B and C. *Org. Biomol. Chem.* **14**, 10362–10365 (2016). 10.1039/c6ob02049f 27779269

[19] Shao, H., Gao, X., Wang, Z.-T., Gao, Z. & Zhao, Y.-M. Divergent biomimetic total syntheses of ganocins A–C, ganocochlearins C and D, and cochlearol T. *Angew. Chem. Int. Ed.* **59**, 7419–7424 (2020). 10.1002/anie.202000677 32096300

[20] Mashiko, T. et al. Total synthesis of cochlearol B via intramolecular [2+2] photocycloaddition. *Angew. Chem. Int. Ed.* **60**, 24484–24487 (2021). 10.1002/anie.202110556 34533883

[21] Richardson, A. D., Vogel, T. R., Traficante, E. F., Glover, K. J. & Schindler, C. S. Total synthesis of (+)-cochlearol B by an approach based on a catellani reaction and visible-light-enabled [2+2] cycloaddition. *Angew. Chem. Int. Ed.* **61**, e2022012 (2022). 10.1002/anie.202201213 PMC9401860 35417620

[22] Mashiko, T. et al. Enantioselective total syntheses of (+)-ganocin A and (–)-cochlearol B. *Org. Lett.* **25**, 8382–8386 (2023). 10.1021/acs.orglett.3c03572 37955425

[23] Zhou, Q. et al. Total synthesis of *Ganoderma* meroterpenoids cochlearol B and its congeners driven by structural similarity and biological homology. *Chem. Eur. J.* **30**, e202400084 (2024). 10.1002/chem.202400084 38228507

[24] Kong, C.-C. et al. Formal synthesis of (±)-ganocins B and C. *Tetrahedron Lett.* **170**, 155776 (2025).

[25] Parsons, P. J., Penkett, C. S. & Shell, A. J. Tandem reactions in organic synthesis: novel strategies for natural product elaboration and the development of new synthetic methodology. *Chem. Rev.* **96**, 195–206 (1996). 10.1021/cr950023+ 11848750

[26] Sears, J. E. & Boger, D. L. Tandem intramolecular Diels–Alder/1,3-dipolar cycloaddition cascade of 1,3,4-oxadiazoles: initial scope and applications. *Acc. Chem. Res.* **49**, 241–251 (2016). 10.1021/acs.accounts.5b00510 PMC4755904 26813287

[27] Liu, X.-Y. & Qin, Y. Indole alkaloid synthesis facilitated by photoredox catalytic radical cascade reactions. *Acc. Chem. Res.* **52**, 1877–1891 (2019). 10.1021/acs.accounts.9b00246 31264824

[28] Gao, K., Hu, J. & Ding, H. Tetracyclic diterpenoid synthesis facilitated by ODI-cascade approaches to bicyclo[3.2.1]octane skeletons. *Acc. Chem. Res.* **54**, 875–889 (2021). 10.1021/acs.accounts.0c00798 33508196

[29] Lisnyak, V. G., Sherwood, T. C. & Snyder, S. A. The development of reaction cascades to synthesize dimeric coccinellid alkaloids. *Acc. Chem. Res.* **54**, 1610–1622 (2021). 10.1021/acs.accounts.0c00806 33646762

[30] Zhao, J.-X. et al. Structural elucidation and bioinspired total syntheses of ascorbylated diterpenoid hongkonoids A–D. *J. Am. Chem. Soc.* **140**, 2485–2492 (2018). 10.1021/jacs.7b10135 29394051

[31] Yang, B. et al. Asymmetric total synthesis and biosynthetic implications of perovskones, hydrangenone, and hydrangenone B. *J. Am. Chem. Soc.* **143**, 6370–6375 (2021). 10.1021/jacs.1c02674 33886312

[32] Liao, X.-Z., Wang, R., Wang, X. & Li, G. Enantioselective total synthesis of (‒)-lucidumone enabled by tandem Prins cyclization/cycloetherification sequence. *Nat. Commun.* **15**, 2647 (2024). 10.1038/s41467-024-46896-3 PMC10966040 38531853

[33] Li, B., Tan, C., Ma, T. & Jia, Y. Bioinspired total synthesis of bipolarolides A and B. *Angew. Chem. Int. Ed.* **63**, e202319306 (2024). 10.1002/anie.202319306 38212293

[34] Wang, Y.-Q., Xu, K., Min, L. & Li, C.-C. Asymmetric total syntheses of hypoestin A, albolic acid, and ceroplastol II. *J. Am. Chem. Soc.* **144**, 10162–10167 (2022). 10.1021/jacs.2c04633 35657330

[35] Liu, L. et al. Total synthesis of (–)−8-*epi*-chromazonarol enabled by a unique N_2_H_4_·H_2_O promoted intramolecular oxa-Michael cyclization reaction. *Org. Chem. Front.* **5**, 3013–3017 (2018).

[36] Nising, C. F. & Bräse, S. The oxa-Michael reaction: from recent developments to applications in natural product synthesis. *Chem. Soc. Rev.* **37**, 1218–1228 (2008). 10.1039/b718357g 18497934

[37] Nising, C. F. & Bräse, S. Recent developments in the field of oxa-Michael reactions. *Chem. Soc. Rev.* **41**, 988–999 (2012). 10.1039/c1cs15167c 21796323

[38] Hu, J., Bian, M. & Ding, H. Recent application of oxa-Michael reaction in complex natural product synthesis. *Tetrahedron Lett.* **57**, 5519–5539 (2016).

[39] Wang, Y. & Du, D.-M. Recent advances in organocatalytic asymmetric oxa-Michael addition triggered cascade reactions. *Org. Chem. Front.* **7**, 3266–3283 (2020).

[40] Ahmad, T. & Ullah, N. The oxa-Michael reaction in the synthesis of 5- and 6-membered oxygen-containing heterocycles. *Org. Chem. Front.* **8**, 1329–1344 (2021).

[41] Song, H. et al. Total syntheses of (–)−15-oxopuupehenol and (+)-puupehenone and formal syntheses of (–)-puupehenol and (+)-puupehedione. *Org. Chem. Front.* **7**, 35–42 (2020).

[42] Tanaka, K. & Sugino, T. Efficient conversion of 2′-hydroxychalcones into flavanones and flavanols in a water suspension medium. *Green Chem.* **3**, 133–134 (2001).

[43] Jiang, H., Zheng, X., Yin, Z. & Xie, J. An efficient catalytic synthesis of flavanones under green conditions. *J. Chem. Res.* **35**, 220–221 (2011).

[44] de Matos, I. L., Birolli, W. G., Santos, D. D. A., Nitschke, M. & Porto, A. L. M. Stereoselective reduction of flavanones by marine-derived fungi. *Mol. Catal.* **513**, 111734 (2021).

[45] Nicolaou, K. C., Li, A., Edmonds, D. J., Tria, G. S. & Ellery, S. P. Total synthesis of platensimycin and related natural products. *J. Am. Chem. Soc.* **131**, 16905–16918 (2009). 10.1021/ja9068003 PMC2783699 19874023

[46] Tian, C. et al. Total syntheses of periconiasins A–E. *Angew. Chem. Int. Ed.* **55**, 6992–6996 (2016). 10.1002/anie.201602439 27121397

[47] Zhao, X., Li, W., Wang, J. & Ma, D. Convergent route to *ent*-kaurane diterpenoids: total synthesis of lungshengenin D and 1α,6α-diacetoxy-*ent*-kaura-9(11), 16-dien-12,15-dione. *J. Am. Chem. Soc.* **139**, 2932–2935 (2017). 10.1021/jacs.7b00140 28186744

[48] Xu, Z.-J. et al. Divergent total synthesis of euphoranginol C, euphoranginone D, *ent*-trachyloban-3β-ol, *ent*-trachyloban-3-one, excoecarin E, and *ent*−16α-hydroxy-atisane-3-one. *Angew. Chem. Int. Ed.* **59**, 19919–19923 (2020). 10.1002/anie.202009128 32696611

[49] Mohri, T., Takahashi, Y., Kwon, E., Kuwahara, S. & Ogura, Y. Stereocontrolled total synthesis of (–)-isocelorbicol and its elaboration to natural dihydro-β-agarofuran esters. *Org. Lett.* **22**, 9234–9238 (2020). 10.1021/acs.orglett.0c03419 33226244

[50] Mashiko, T., Nagata, E., Sakate, H., Kamo, S. & Sugita, K. Total synthesis of (+)-*ent*-vetiverianine A via Lewis acid-mediated cyclization. *Org. Chem. Front.* **9**, 6849–6852 (2022).

[51] Panda, S., Chavan, N. D. & Ghorai, P. Intramolecular cascade cyclization of cyclobutanone: asymmetric construction of cyclobutanone fused oxa-spirocycles. *Org. Lett.* **27**, 541–545 (2025). 10.1021/acs.orglett.4c03593 39772593

[52] Ebner, C. & Carreira, E. M. Pentafulvene for the synthesis of complex natural products: total syntheses of (±)-pallambins A and B. *Angew. Chem. Int. Ed.* **54**, 11227–11230 (2015). 10.1002/anie.201505126 26230353

[53] Kuroda, Y. et al. Synthesis and bioactivity studies of phomactin terpenoids. *Nat. Chem.* **10**, 938–945 (2018). 10.1038/s41557-018-0084-x 30061613

[54] Poplata, S., Tröster, A., Zou, Y.-Q. & Bach, T. Recent advances in the synthesis of cyclobutanes by olefin [2+2] photocycloaddition reactions. *Chem. Rev.* **116**, 9748–9815 (2016). 10.1021/acs.chemrev.5b00723 PMC5025837 27018601

[55] Wang, M. & Lu, P. Catalytic approaches to assemble cyclobutane motifs in natural product synthesis. *Org. Chem. Front.* **5**, 254–259 (2018).

[56] Li, J., Gao, K., Bian, M. & Ding, H. Recent advances in the total synthesis of cyclobutane-containing natural products. *Org. Chem. Front.* **7**, 136–154 (2020).

[57] Zhao, W., Al-Ahmad, R. & Dai, M. Total synthesis of (–)-psathyrin a enabled by radical cyclization. *J. Am. Chem. Soc.* **147**, 32365–32369 (2025). 10.1021/jacs.5c11534 PMC12426916 40853837

[58] Edmonds, D. J., Johnston, D. & Procter, D. J. Samarium(II)-iodide-mediated cyclizations in natural product synthesis. *Chem. Rev.* **104**, 3371–3404 (2004). 10.1021/cr030017a 15250745

[59] Nicolaou, K. C., Ellery, S. P. & Chen, J. S. Samarium diiodide mediated reactions in total synthesis. *Angew. Chem. Int. Ed.* **48**, 7140–7165 (2009). 10.1002/anie.200902151 PMC2771673 19714695

[60] Harb, H. Y. & Procter, D. J. SmI_2_-mediated carbonyl-alkene couplings for the synthesis of small carbocyclic rings. *Synlett* **23**, 6–20 (2012).

[61] Szostak, M., Fazakerley, N. J., Parmar, D. & Procter, D. J. Cross-coupling reactions using samarium(II) iodide. *Chem. Rev.* **114**, 5959–6039 (2014). 10.1021/cr400685r 24758360

[62] Gao, Y. & Ma, D. Samarium iodide-mediated C–C bond formation in the total synthesis of natural products. *Nat. Synth.* **1**, 275–288 (2022).

[63] Banwell, M. G., Hockless, D. C. R. & McLeod, M. D. Chemoenzymatic total syntheses of the sesquiterpene (–)-patchoulenone. *New J. Chem.* **27**, 50–59 (2003).

[64] Wang, N. et al. Visible-light-promoted tandem thiol–ene click reaction/transannular cyclization and regioselective cyclopropane ring-opening to construct sulfur-containing *Euphorbia* diterpenes. *Org. Lett.* **25**, 597–602 (2023). 10.1021/acs.orglett.2c04116 36662155

[65] Cen, K. et al. Bioinspired divergent synthesis of aspersteroids A and B. *J. Am. Chem. Soc.* **146**, 6481–6486 (2024). 10.1021/jacs.4c01016 38421318

[66] González-Delgado, J. A., Arteaga, J. F., Herrador, M. M. & Barrero, A. F. First total synthesis of (+)-apotrisporin E and (+)-apotrientriols A–B: a cyclization approach to apocarotenoids. *Org. Biomol. Chem.* **11**, 5404–5408 (2013). 10.1039/c3ob41226a 23863958

[67] Burns, B. et al. Palladium catalysed tandem cyclisation-anion capture processes. Part 1. Background and hydride ion capture by alkyl- and π-allyl-palladium species. *Tetrahedron* **48**, 7297–7320 (1992).

[68] Wang, H., Qu, J.-P. & Kang, Y.-B. CBZ6 as a recyclable organic photoreductant for pinacol coupling. *Org. Lett.* **23**, 2900–2903 (2021). 10.1021/acs.orglett.1c00537 33797933

[69] Wang, S.-D., Yang, B., Zhang, H., Qu, J.-P. & Kang, Y.-B. Reductive cleavage of C–X or N–S bonds catalyzed by super organoreductant CBZ6. *Org. Lett.* **25**, 816–820 (2023). 10.1021/acs.orglett.2c04346 36693162

