# Supplementary material for: Divergent asymmetric synthesis of (–)-ganocins A–C and (+)-cochlearol B via a non-biomimetic strategy
Source: Nat Commun. 2026 Oct 15;17:11650. doi: 10.1038/s41467-026-78287-1 (PMC13759510; doi:10.1038/s41467-026-78287-1)
Supplement: Supplementary file 1 [file 41467_2026_78287_MOESM1_ESM.pdf]

Supplementary Information

Divergent Asymmetric Synthesis of (–)-Ganocins A–C and (+)-Cochlearol B via a Non-biomimetic Strategy

Hailong Xu[1,‡], Yu Yang[1,‡], Jinghua Wu[1], Huanfeng Jiang[1] & Zhiqiang Ma[1,2,3,*]

[1]Key Lab of Functional Molecular Engineering of Guangdong Province, School of Chemistry & Chemical Engineering, South China University of Technology, Wushan Road-381, Guangzhou 510641, P. R. China.

[2]State Key Laboratory of Natural and Biomimetic Drugs, Peking University, Beijing 100191, P. R. China.

[3]School of Chemical Science and Technology, Yunnan University, Kunming 650091, P. R. China.

‡These authors contributed equally: Hailong Xu, Yu Yang.

*e-mail: cezqma@scut.edu.cn

Table of Contents

1. General Experimental Procedures……S2

2. Summary of Prior Synthetic Reports……S3

3. Experimental Procedures and Experimental Data……S5

4. X-ray Crystallographic Data……S31

5. Comparison of the Spectra and Data of (–)-Ganocins A–C……S36

6. Comparison of the Spectra and Data of (+)-Cochlearol B……S42

7. ^{1}H and ^{13}C NMR Spectra of the Synthetic Intermediates, Ganocins A–C and Cochlearol B……S44

8. Supplementary References……S82

1. General Experimental Procedures

All reactions were carried out under nitrogen atmosphere anhydrous conditions and all reagents were purchased from commercial suppliers without further purification. Unless stated otherwise, reactions were performed at 33 °C. Anhydrous THF (tetrahydrofuran), DCM (dichloromethane), PhMe (toluene), and DMF (*N,N*-dimethylformamide) were obtained from an Inert PS-MD-4 solvent system. Anhydrous CH_3CN (acetonitrile), benzene, PhCl (chlorobenzene), DMSO (dimethyl sulfoxide), MeOH (methanol), EtOH (ethanol), *t*-BuOH (*tert*-butanol), *i*-PrOH (isopropanol), EtOAc (ethyl acetate), and 1,4-dioxane were purchased from Energy Chemical, Ltd. HMPA (hexamethylphosphoramide), Et_3N (triethylamine) and Et_2O (diethyl ether) were obtained from commercial sources and subjected to anhydrous treatment prior to use. AcOH (acetic acid) was commercially available and was used without anhydrous treatment. ^{1}H NMR and ^{13}C NMR spectra were recorded on a Bruker Avance 400 MHz NMR spectrometer (400 MHz for ^{1}H, 100 MHz for ^{13}C) or a Bruker Avance 500 MHz NMR spectrometer (500 MHz for ^{1}H, 125 MHz for ^{13}C). Chemical shifts were referenced using residual undeuterated solvent ($CDCl_3$: δ 7.26, 77.16 ppm; C_6D_6: δ 7.16, 128.06 ppm; C_5D_5N: 7.19, 149.0 ppm; $(CD_3)_2CO$: δ 2.05, 29.84 ppm unless otherwise stated) at 298 K. HRMS(ESI) spectra were acquired on a Waters Xevo G3 QTof mass spectrometer. IR spectra were recorded on a Nicolet 210 spectrophotometer. Melting points were determined using a hot stage apparatus. Optical rotations were measured with a Rudolph Research Automatic Polarimeter IV. Single-crystal structures were measured on a SuperNova, Dual, Cu at zero, AtlasS2 diffractometer. TLC was performed on commercially prepared silica gel plates (GF254, 200-300 mesh) and visualized under UV light at 254 nm or by staining with phosphomolybdic acid or potassium permanganate solution.

2. Summary of Prior Synthetic Reports

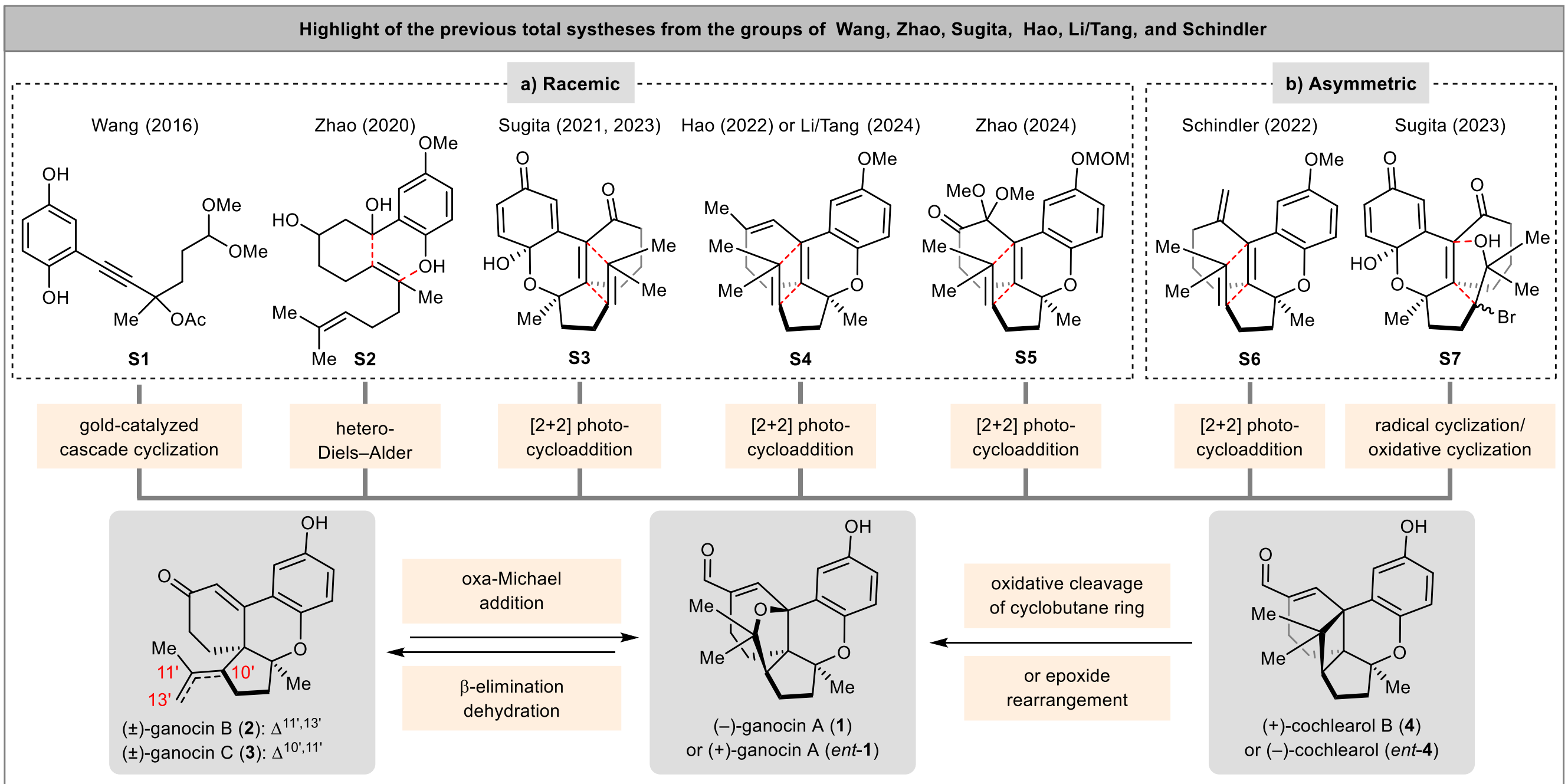

Groups	ganocin A (**1**)	ganocin B (**2**)	ganocin C (**3**)	cochlearol B (**4**)	stereochemical outcome	chromane/chromene	tetrahydrofuran ring	cyclobutane ring
Wang (2016)	—	11 steps (6%)	11 steps (6%)	—	racemic	gold-catalyzed cascade cyclization	—	—
Zhao (2020)	13 steps (0.3%)	9 steps (2%)	9 steps (2%)	—	racemic	hetero-Diels–Alder	oxa-Michael addition	—
Sugita (2021)	—	—	—	16 steps (9%)	racemic	oxidative cyclization	—	All five research groups employed [2+2] photo-cycloaddition to synthesize cyclobutane.
Hao (2022)	10 steps (2%)	12 steps (0.3%)	12 steps (0.7%)	8 steps (4%)	racemic	hetero-Diels–Alder	oxidative cleavage of cyclobutane ring	
Li/Tang (2024)	8 steps (7%)	—	—	7 steps (11%)	racemic	hetero-Diels–Alder	oxidative cleavage of cyclobutane ring	
Zhao (2024)	—	—	—	11 steps (7%)	racemic	Pd-catalyzed cascade reaction	—	
Schindler (2022)	—	—	—	14 steps (0.9%)	asymmetric (chiral resolution)	Kabbe condensation	—	
Sugita (2023)	17 steps (9%)	—	—	16 steps (9%)	asymmetric	oxidative cyclization	oxidative cyclization (create a ring in a step)	
this work	11 steps (11%)	8 steps (13%)	8 steps (22%)	15 steps (1.5%)	asymmetric	amino acid catalyzed oxa-Michael addition	cascade aldol/ etherification cyclization (create two ring in a step)	SmI_2-mediated carbonyl–alkene coupling

Supplementary Figure 1. Total synthesis of ganocins A–C (1−3) and cochlearol B (4). a Racemic. **b** Asymmetric.

In the past decade, numerous elegant racemic syntheses of ganocins A–C (**1–3**) and cochlearol B (**4**) have been achieved (Supplementary Figure 1a)[1–6]. In 2016, Wang and Li et al. reported the first total synthesis of racemic ganocins B (**2**) and C (**3**) in 11 steps with 6% overall yields, which features a gold-catalyzed intramolecular cascade cyclization of propargyl acetate **S1** to construct the 6/6/5 tricyclic skeleton[1]. In 2020, Zhao and co-workers achieved the divergent total synthesis of racemic ganocins A–C (**1–3**) in 13, 9, and 9 steps with 0.3%, 2%, and 2% overall yields, respectively[2]. Notable steps in their synthesis include a biomimetic hetero-Diels–Alder annulation of **S2** to construct the tricyclic scaffold and an intramolecular oxa-Michael addition/triflation cascade reaction to forge the tetrahydrofuran ring. In the subsequent year, Sugita et al. reported the first total synthesis of racemic cochlearol B (**4**) in 16 steps with 9% overall yields[3]. The key steps include an oxidative cyclization and intramolecular [2+2] photocycloaddition of enone **S3** to construct the pentacyclic skeleton. In 2022, Hao et al. disclosed the unified and biomimetic synthesis of racemic ganocins A–C (**1–3**)

and cochlearol B (**4**) (10, 12, 12, and 8 steps with 2%, 0.3%, 0.7%, and 4% overall yields, respectively)[4]. They used an intramolecular [2+2] photocycloaddition of diene **S4** to construct the cyclobutane part of the pentacyclic skeleton, and either a complex carbocation rearrangement of epoxide or oxidative cleavage of cyclobutane to convert the cyclobutane intermediate into a tetrahydrofuran moiety present in ganocin A. In 2024, besides [2+2] photocycloaddition of diene **S4**, Li and Tang et al. utilized epoxide rearrangement or 2,3-dicyano-5,6-dichlorobenzoquinone (DDQ)-induced oxidative cleavage of cyclobutane to efficiently convert cochlearol B (**4**) (7 steps with 11% overall yields) to ganocin A (**1**) (8 steps with 7% overall yields)[5]. In the same year, Zhao and co-workers reported the total synthesis of racemic cochlearol B (**4**) in 11 steps with 7% overall yields[6]. Their synthesis features a novel Pd-catalyzed Wacker-type/cross-coupling cascade reaction and an intramolecular [2+2] photocycloaddition of alkene **S5**.

So far, only one asymmetric synthesis of ganocin A (**1**) and two asymmetric syntheses of cochlearol B (**4**) have been reported, respectively (Supplementary Figure 1b)[7,8]. For example, in 2022, Schindler and co-workers disclosed the first asymmetric total synthesis of (+)-cochlearol B (**4**) using chiral resolution in 14 steps with 0.9% overall yields[7], including an organocatalytic Kabbe condensation and a visible-light-mediated [2+2] photocycloaddition of diene **S6** to construct the bicyclo[3.2.0]heptane core. One year later, Sugita and co-workers reported asymmetric syntheses of (+)-ganocin A (*ent*-**1**) and (–)-cochlearol B (*ent*-**4**) based on their previous racemic synthesis[8]. Using an asymmetric Corey−Bakshi−Shibata reduction to install the stereocenter, they completed the total synthesis of (–)-cochlearol B (*ent*-**4**) in 16 steps with 9% overall yields. Subsequently, they achieved the first asymmetric synthesis of (+)-ganocin A (*ent*-**1**) in 17 steps with 9% overall yields via intramolecular radical cyclization of bromohydrin **S7** and benzylic oxidative cyclization.

Our group accomplished the divergent asymmetric total synthesis of the (–)-ganocins A–C (**1−3**) and (+)-cochlearol B (**4**) from (*R*)-limonene in 11, 8, 8, and 15 steps with 11%, 13%, 22%, and 1.5% overall yields, respectively. The synthesis features an amino acid-catalyzed diastereoselective oxa-Michael addition to construct the chromanone framework, acid-dependent divergent reaction pathways of a common intermediate, wherein aldol/dehydration provides the 5/6/6/6 tetracyclic framework whereas an efficient cascade aldol/etherification cyclization leads to the 5/5/6/6/6 pentacyclic skeleton, and a SmI_2-mediated 4-*exo*-trig reductive cross-coupling reaction that rapidly forges the highly strained cyclobutanol ring.

3. Experimental Procedures and Experimental Data

Preparation of Compound 14

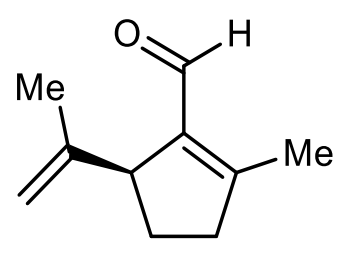

14

Compound **14** was prepared according to the literature procedure[9]. Analytical data matches with the literature.

Preparation of Compound 15

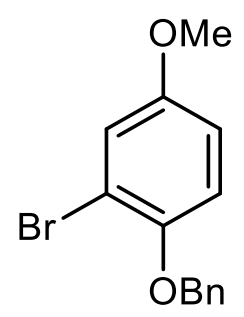

15

Compound **15** was prepared according to the literature procedure[10]. Analytical data matches with the literature.

Preparation of Compound 16

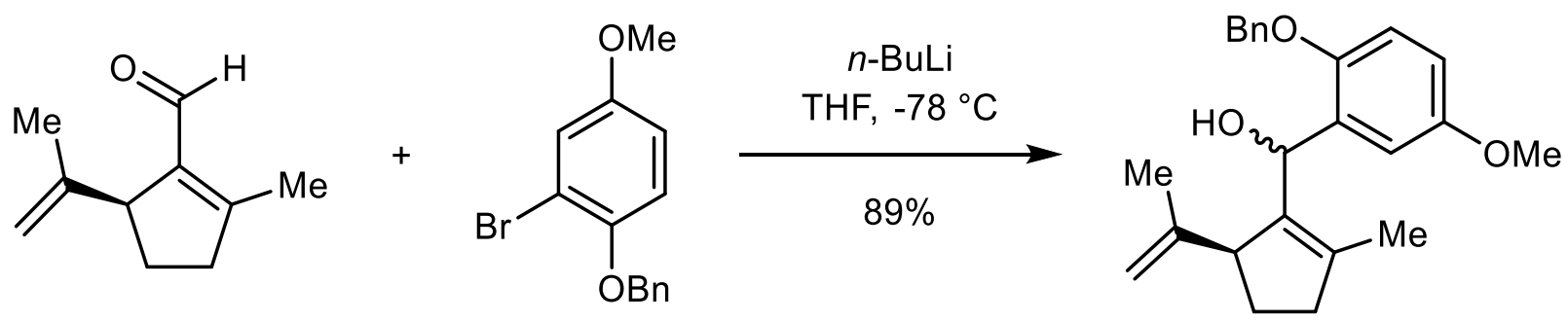

14 **15** **16**

To a solution of **15** (17.5 g, 60.0 mmol,1.5 equiv) in THF (250 mL) was added dropwise *n*-BuLi (2.5 M in hexane, 24.8 mL, 62.0 mmol, 1.55 equiv) at –78 °C and stirred at this temperature for 10 min, a solution of compound **14** (6.0 g, 40.0 mmol, 1.0 equiv) in THF (102 mL) was added dropwise. After stirring at same temperature for 2 h, the reaction was quenched with saturated aqueous NH_4Cl solution. The reaction mixture was extracted with EtOAc and the combined organic layers were washed with brine, dried over $MgSO_4$, filtered and concentrated under reduced pressure. The residue was purified by silica gel column chromatography (petroleum ether:EtOAc = 50:1→20:1→10:1→5:1) to afford **16** as a mixture of two diastereomers (12.9 g, 89% yield) as a yellow oil.

***Note**: The two diastereomers can be separated by preparative thin layer chromatography (PTLC, petroleum ether:EtOAc = 8:1) to afford **16a** and **16b**.*

Data for 16a:

TLC: R_f = 0.6 (petroleum ether:EtOAc = 8:1).

$[\alpha]_D^{26}$ = +169 (c = 1.0, $CHCl_3$).

^{1}H NMR (500 MHz, $CDCl_3$) δ 7.40 – 7.28 (m, 5H), 7.09 (d, *J* = 3.1 Hz, 1H), 6.80 (d, *J* = 8.8 Hz, 1H), 6.72 (dd, *J* = 8.8, 3.1 Hz, 1H), 5.90 (d, *J* = 6.2 Hz, 1H), 5.00 (s, 2H), 4.80 (s, 1H), 4.73 (s, 1H), 3.79 (s, 3H), 3.18 (d, *J* = 9.5 Hz,1H), 2.58 (d, *J* = 6.6 Hz, 1H), 2.44 (dt, *J* = 16.8, 8.5 Hz, 1H), 2.15 (ddd, *J* = 16.3, 9.8, 2.4 Hz, 1H), 1.97 – 1.87 (m, 1H), 1.73 (s, 3H), 1.61 (s, 3H), 1.59 – 1.52 (m, 1H).

^{13}C NMR (125 MHz, $CDCl_3$) δ 153.7, 152.1, 149.7, 139.0, 137.4, 135.3, 133.3, 128.5, 128.0, 127.7, 113.2, 112.7, 112.4, 110.2, 70.8, 67.2, 55.8, 52.9, 37.9, 29.3, 19.8, 14.3.

HRMS(ESI)$^+$ calcd for $C_{24}H_{28}O_3Na^+$ [M+Na]$^+$: 387.1931; found: 387.1935.

FT-IR (cm^{-1}): 2923, 1494, 1454, 1209, 1045, 1021, 734, 696.

Data for 16b:

TLC: R_f = 0.5 (petroleum ether:EtOAc = 8:1).

$[\alpha]_D^{26}$ = +48 (c = 0.5, $CHCl_3$).

^{1}H NMR (400 MHz, $CDCl_3$) δ 7.41 – 7.29 (m, 5H), 7.00 (d, *J* = 3.1 Hz, 1H), 6.80 (d, *J* = 8.8 Hz, 1H), 6.71 (dd, *J* = 8.8, 3.1 Hz, 1H), 5.58 (s, 1H), 5.04 (d, *J* = 11.6 Hz, 1H), 5.01 (d, *J* = 11.6 Hz, 1H), 4.51 (s, 2H), 3.76 (s, 3H), 3.26 (d, *J* = 9.3 Hz, 1H), 2.50 – 2.35 (m, 1H), 2.29 – 2.17 (m, 1H), 2.07 – 1.93 (m, 1H), 1.76 (s, 3H), 1.64 – 1.55 (m, 1H), 1.50 (s, 3H).

^{13}C NMR (125 MHz, $CDCl_3$) δ 153.7, 150.4, 148.7, 138.7, 137.2, 135.9, 132.4, 128.7, 128.1, 127.7, 114.2, 112.53, 112.50, 110.2, 70.8, 68.0, 55.9, 55.2, 38.7, 29.1, 19.3, 15.0.

HRMS(ESI)$^+$ calcd for $C_{24}H_{28}O_3Na^+$ $[M+Na]^+$: 387.1931; found: 387.1932.

FT-IR (cm^{-1}): 3468, 2918, 2845, 1494, 1454, 1207, 1044, 1016, 733.

Preparation of Compound 17

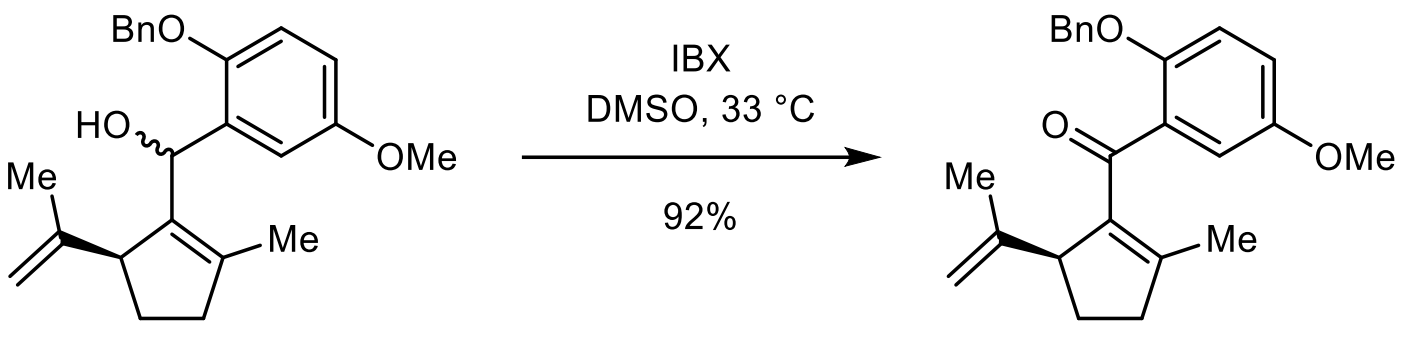

16 **17**

To a solution of **16** (2.96 g, 8.13 mmol, 1.0 equiv) in DMSO (81.0 mL) was added 2-iodoxybenzoic acid (IBX, 2.73 g, 9.76 mmol, 1.2 equiv) at 33 °C. After stirring at same temperature for 1.5 h, the reaction was quenched with a mixture of saturated aqueous $NaHCO_3$ solution and saturated aqueous $Na_2S_2O_3$ solution. The reaction mixture was extracted with EtOAc and the combined organic layers were washed with brine, dried over Na_2SO_4, filtered and concentrated under reduced pressure. The residue was purified by silica gel column chromatography (petroleum ether:EtOAc = 20:1→10:1) to afford **17** (2.70 g, 92% yield) as a yellow oil.

TLC: R_f = 0.5 (petroleum ether:EtOAc = 10:1).

$[\alpha]_D^{25}$ = –21 (c = 1.0, $CHCl_3$).

^{1}H NMR (500 MHz, $CDCl_3$) δ 7.39 – 7.27 (m, 5H), 6.94 – 6.85 (m, 3H), 5.00 (d, *J* = 11.5 Hz, 1H), 4.98 (d, *J* = 11.6 Hz, 1H), 4.54 (s, 1H), 4.50 (s, 1H), 3.76 (s, 3H), 3.73 – 3.65 (m, 1H), 2.48 (dt, *J* = 16.3, 7.4 Hz, 1H), 2.23 (ddd, *J* = 17.0, 8.9, 5.7 Hz, 1H), 2.02 – 1.91 (m, 1H), 1.80 (s, 3H), 1.61 – 1.51 (m, 1H), 1.54 (s, 3H).

^{13}C NMR (125 MHz, $CDCl_3$) δ 195.0, 153.9, 153.8, 150.3, 147.9, 139.8, 137.0, 132.4, 128.5, 127.9, 127.4, 117.3, 114.4, 114.3, 110.2 71.5, 55.9, 54.5, 39.6, 28.9, 20.4, 16.3.

HRMS(ESI)$^+$ calcd for $C_{24}H_{26}O_3Na^+$ $[M+Na]^+$: 385.1774; found: 385.1779.

FT-IR (cm^{-1}): 2939, 2910, 1628, 1493, 1454, 1283, 1220, 1043, 736.

Supplementary Table 1. Optimization of selective debenzylation of compound 17

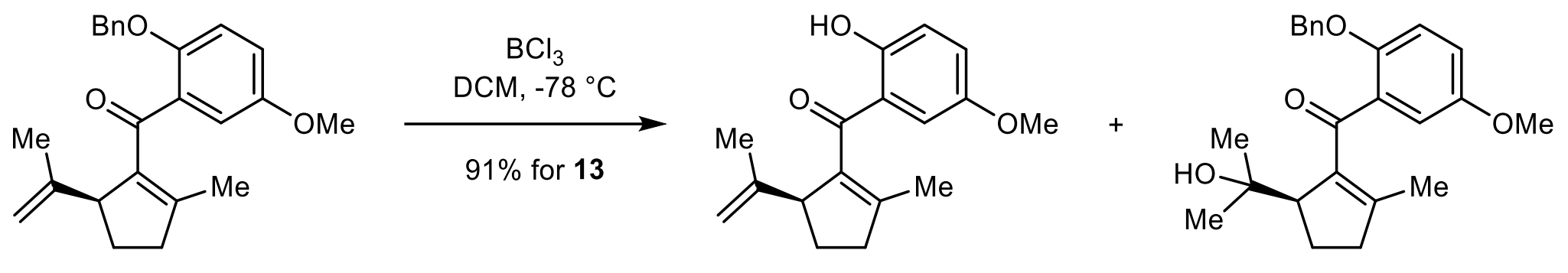

Entry	Conditions	13 (%)[a]	S8 (%)[a]
1	$TiCl_4$, DCM, 0 °C	0	28
2	TFA, toluene, 0 °C	0	<10
3	$FeCl_3$, DCM, 0 °C	0	53
4	BCl_3, DCM, –78 °C	91	0
5	DDQ, DCM/H_2O (10:1), 33 to 50 °C	complex mixture	

[a]Isolated yield. TFA = trifluoroacetic acid. DDQ = 2,3-dichloro-5,6-dicyano-1,4 benzoquinone.

Preparation of Compound 13

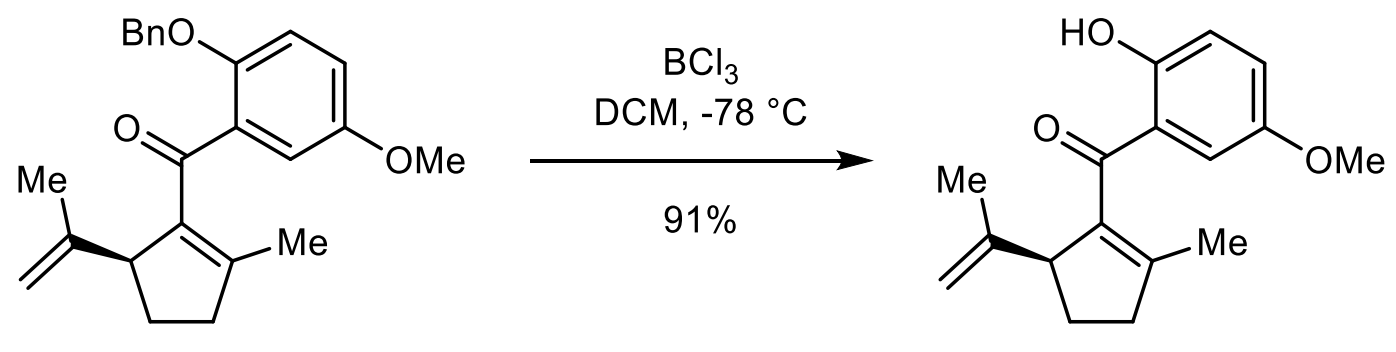

To a solution of **17** (14.5 g, 40.1 mmol,1.0 equiv) in DCM (400 mL) was added BCl_3 (1 M in hexane, 48.1 mL, 48.1 mmol, 1.2 equiv) at –78 °C. After stirring at same temperature for 1 h, the reaction was quenched with MeOH and saturated aqueous $NaHCO_3$ solution. The reaction mixture was extracted with DCM and the combined organic layers were washed with brine, dried over Na_2SO_4, filtered and concentrated under reduced pressure. The residue was purified by silica gel column chromatography (petroleum ether:EtOAc = 20:1→10:1) to afford **13** (9.92 g, 91% yield) as a yellow solid.

TLC: R_f = 0.7 (petroleum ether:EtOAc = 9:1).

M.p.: 61.1–62.2 °C.

$[\alpha]_D^{25}$ = +82 (c = 1.0, $CHCl_3$).

^{1}H NMR (500 MHz, $CDCl_3$) δ 11.85 (s, 1H), 7.15 (d, *J* = 3.1 Hz, 1H), 7.10 (dd, *J* = 9.1, 3.1 Hz, 1H), 6.93 (d, *J* = 9.0 Hz, 1H), 4.73 (s, 1H), 4.67 (s, 1H), 3.99 – 3.92 (m, 1H), 3.76 (s, 3H), 2.65 – 2.55 (m, 1H), 2.48 (dt, *J* = 17.1, 8.2 Hz, 1H), 2.29 – 2.20 (m, 1H), 1.83 – 1.73 (m, 1H), 1.74 (s, 3H), 1.66 (s, 3H).

^{13}C NMR (125 MHz, $CDCl_3$) δ 201.9, 157.1, 151.8, 148.2, 146.8, 137.4, 123.9, 119.9, 119.2, 115.7, 111.5, 57.0, 56.1, 39.1, 29.2, 20.0, 17.0.

HRMS(ESI)$^+$ calcd for $C_{17}H_{20}O_3Na^+$ $[M+Na]^+$: 295.1305; found: 295.1306.

FT-IR (cm^{-1}): 3647, 2939, 1635, 1586, 1483, 1282, 1209, 1039, 791.

Preparation of Compound S8

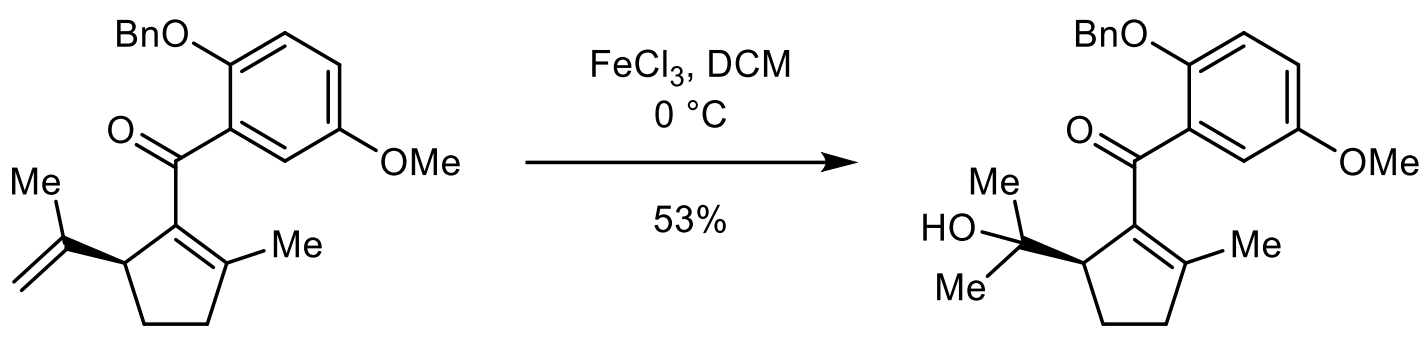

17 **S8**

To a solution of **17** (5.4 mg, 0.0149 mmol,1.0 equiv) in DCM (0.2 mL) was added $FeCl_3$ (8.0 mg, 0.0492 mmol, 3.3 equiv) at 0 °C. After stirring at same temperature for 43 min, the reaction was quenched with saturated aqueous $NaHCO_3$ solution. The reaction mixture was extracted with EtOAc and the combined organic layers were washed with brine, dried over Na_2SO_4, filtered and concentrated under reduced pressure. The residue was purified by PTLC (petroleum ether:EtOAc = 5:1) to afford **S8** (3.0 mg, 53% yield) as a yellow oil.

TLC: R_f = 0.2 (petroleum ether:EtOAc = 5:1).

$[\boldsymbol{\alpha}]_D^{24}$ = +10 (c = 0.2, $CHCl_3$).

^{1}H NMR (500 MHz, $CDCl_3$) δ 7.39 – 7.30 (m, 5H), 7.00 (d, *J* = 3.0 Hz, 1H), 6.95 (dd, *J* = 8.9, 3.0 Hz, 1H), 6.92 (d, *J* = 8.9 Hz, 1H), 5.01 (d, *J* = 11.5 Hz, 1H), 4.98 (d, *J* = 11.6 Hz, 1H), 3.79 (s, 3H), 3.49 (brs, 1H), 3.28 – 3.17 (m, 1H), 2.52 – 2.40 (m, 1H), 2.21 – 2.09 (m, 1H), 1.88 – 1.76 (m, 1H), 1.61 (s, 3H), 1.60 – 1.55 (m, overlap with H_2O, 1H), 1.15 (s, 3H), 1.02 (s, 3H).

^{13}C NMR (125 MHz, $CDCl_3$) δ 197.2, 156.0, 154.2, 150.7, 139.4, 136.8, 132.1, 128.7, 128.3, 127.7, 118.3, 114.7, 114.6, 73.7, 71.7, 59.2, 56.0, 40.1, 29.5, 26.2, 25.3, 16.7.

HRMS(ESI)$^+$ calcd for $C_{24}H_{29}O_4^+$ $[M+H]^+$: 381.2060; found: 381.2065.

FT-IR (cm^{-1}): 3446, 2967, 2917, 1603, 1492, 1414, 1218, 1042, 737.

Preparation of Ligands

L1-**L6** and **L9**-**L20** were commercially available and used as received.

L7[11] and **L8**[12] were synthesized according to literature procedures. Analytical data matches with the literature.

Supplementary Table 2. Optimization of oxa-Michael reaction of compound 13

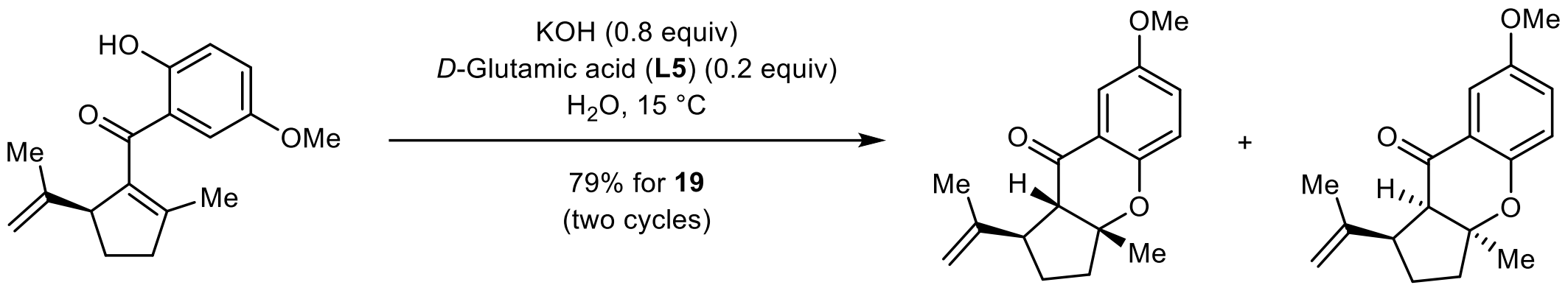

13 **18** undesired **19** desired

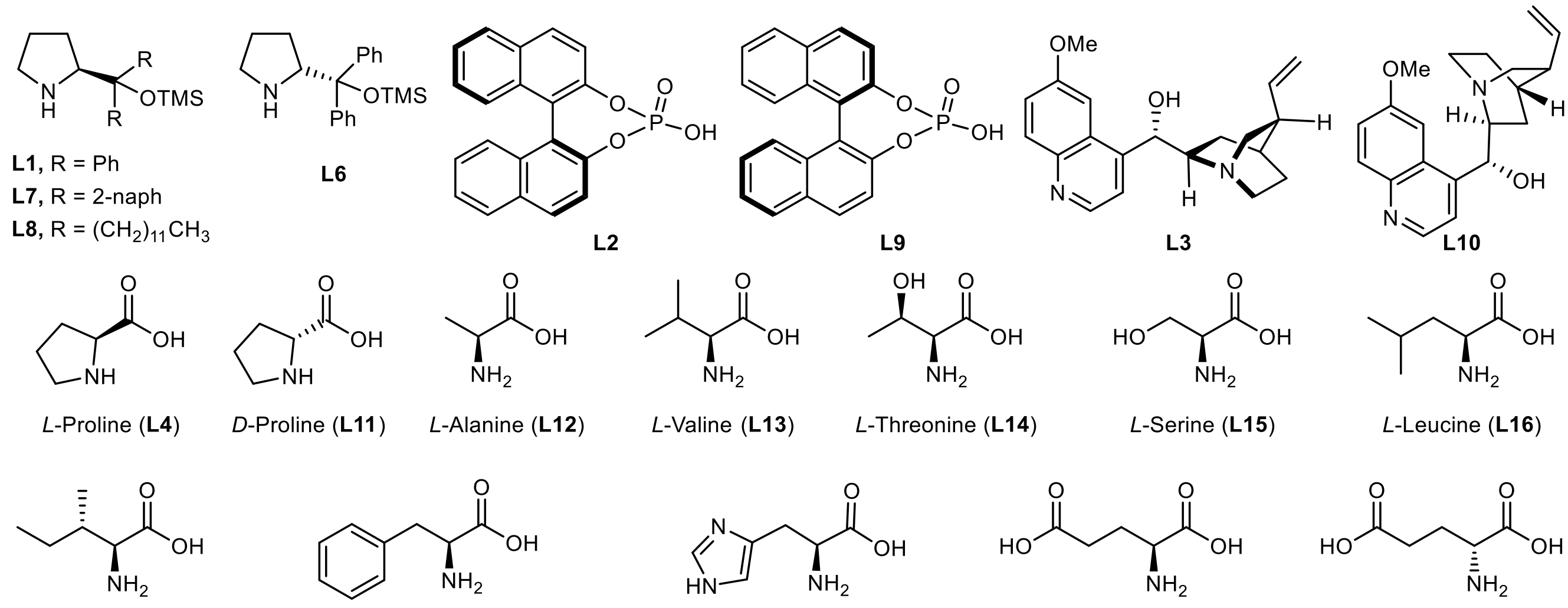

L1, R = Ph
L7, R = 2-naph
L8, R = $(CH_2)_{11}CH_3$
L6 **L2** **L9** **L3** **L10**

L-Proline (**L4**) *D*-Proline (**L11**) *L*-Alanine (**L12**) *L*-Valine (**L13**) *L*-Threonine (**L14**) *L*-Serine (**L15**) *L*-Leucine (**L16**)

L-Isoleucine (**L17**) *L*-Phenylalanine (**L18**) *L*-Histidine (**L19**) *L*-Glutamic acid (**L20**) *D*-Glutamic acid (**L5**)

Entry	Conditions[a]	**18** (%)[b]	**19** (%)[b]	Ratio of **18:19**
1	TFA, Toluene, 33 to 60 °C	0	0	-
2	TfOH, Toluene, 33 °C	0	0	-
3	KOH, DEG, 100 °C	97	0	1:0
4[c]	N_2H_4•H_2O, DEG, 33 °C	48	18	1:0.4
5[c,d]	N_2H_4•H_2O, DEG, 100 °C	48	16	1:0.3
6[c,d]	N_2H_4•H_2O, DEG, 200 °C	36	0	1:0
7	$PhNHNH_2$, DEG, 33 °C	38	17	1:0.4
8	$PhNH_2$, DEG, 33 °C	54	26	1:0.5
9	**L1**, DCM, 33 to 60 °C		NR	
10	**L6**, DCM, 33 to 60 °C		NR	
11	**L7**, DCM, 33 to 60 °C		NR	
12	**L8**, DCM, 33 to 60 °C		NR	
13	**L2**, toluene, 33 to 100 °C		NR	
14	**L9**, toluene, 33 to 100 °C		NR	
15	**L3**, toluene, 33 to 130 °C	49	23	1:0.5
16	**L10**, toluene, 33 to 130 °C	59	23	1:0.4

17	KOH (1.1 equiv), *L*-Proline (**L4**) (0.2 equiv), H_2O, 33 °C	32	45	1:1.4
18	KOH (1.1 equiv), *D*-Proline (**L11**) (0.2 equiv), H_2O, 33 °C	70	23	1:0.3
19	KOH (1.1 equiv), *L*- Alanine (**L12**) (0.2 equiv), H_2O, 33 °C	49	42	1:0.9
20	KOH (1.1 equiv), *L*-Valine (**L13**) (0.2 equiv), H_2O, 33 °C	50	45	1:0.9
21	KOH (1.1 equiv), *L*-Threonine (**L14**) (0.2 equiv) H_2O, 33 °C	48	50	1:1.0
22	KOH (1.1 equiv), *L*-Serine (**L15**) (0.2 equiv), H_2O, 33 °C	59	35	1:0.6
23	KOH (1.1 equiv), *L*-Leucine (**L16**) (0.2 equiv), H_2O, 33 °C	49	51	1:1.0
24	KOH (1.1 equiv), *L*-Isoleucine (**L17**) (0.2 equiv), H_2O, 33 °C	49	47	1:1.0
25	KOH (1.1 equiv), *L*-Phenylalanine (**L18**) (0.2 equiv), H_2O, 33 °C	51	43	1:0.8
26	KOH (1.1 equiv), *L*-Histidine (**L19**) (0.2 equiv), H_2O, 33 °C	58	40	1:0.7
27	KOH (1.1 equiv), *L*-Glutamic acid (**L20**) (0.2 equiv), H_2O, 33 °C	44	52	1:1.2
28	KOH (1.1 equiv), *D*-Glutamic acid (**L5**) (0.2 equiv), H_2O, 33 °C	38	61	1:1.6
29[c,e]	KOH (1.1 equiv), *D*-Glutamic acid (**L5**) (0.2 equiv), H_2O, 33 °C	53	45	1:0.8
30	KOH (1.1 equiv), $PhNHNH_2$ (0.2 equiv), H_2O, 33 °C	63	16	1:0.3
31	KOH (1.1 equiv), $PhNH_2$ (0.2 equiv), H_2O, 33 °C	67	19	1:0.3
32	KOH (1.1 equiv), **L1** (0.2 equiv), H_2O, 33 °C	41	58	1:1.4
33	KOH (1.1 equiv), **L6** (0.2 equiv), H_2O, 33 °C	40	57	1:1.4
34	KOH (1.1 equiv), **L7** (0.2 equiv), H_2O, 33 °C	46	46	1:1.0
35	KOH (1.1 equiv), **L8** (0.2 equiv), H_2O, 33 °C	47	51	1:1.1
36	KOH (1.1 equiv), **L2** (0.2 equiv), H_2O, 33 °C	41	41	1:1.0
37	KOH (1.1 equiv), **L9** (0.2 equiv), H_2O, 33 °C	43	38	1:0.9
38	KOH (1.1 equiv), **L3** (0.2 equiv), H_2O, 33 °C	39	60	1:1.5
39	KOH (1.1 equiv), **L10** (0.2 equiv), H_2O, 33 °C	41	48	1:1.2
40	KOH (1.1 equiv), *D*-Glutamic acid (**L5**) (0.2 equiv), H_2O, 23 °C	36	52	1:1.4
41	KOH (1.1 equiv), *D*-Glutamic acid (**L5**) (0.2 equiv), H_2O, 15 °C	26	56	1:2.2
42	KOH (1.1 equiv), *D*-Glutamic acid (**L5**) (0.2 equiv), H_2O, 5 °C	22	44	1:2.0
43	KOH (1.1 equiv), *D*-Glutamic acid (**L5**) (1.2 equiv), H_2O, 15 °C		NR	
44	KOH (0.8 equiv), *D*-Glutamic acid (**L5**) (0.2 equiv), H_2O, 15 °C	29	64	1:2.2
45	KOH (0.8 equiv), **L1** (0.2 equiv), H_2O, 15 °C	31	62	1:2.0
46	KOH (0.8 equiv), **L3** (0.2 equiv), H_2O, 15 °C	24	39	1:1.6
47[c,f]	KOH (0.8 equiv), *D*-Glutamic acid (**L5**) (0.2 equiv), H_2O, 15 °C	34	62(79)[g]	1:1.8

[a]all reactions were performed on a 20.0 mg scale in 0.74 mL solvent except as otherwise indicated. [b]Yield determined by ^1H NMR with CH_2Br_2 as an internal standard. [c]Isolated yield. [d]**13** (5.0 mg). [e]**13** (500 mg). [f]**13** (1.0 g). [g]Yield after two cycles (in parentheses). TfOH = trifluoromethanesulfonic acid. DEG = diethylene glycol. $N_2H_4 \cdot H_2O$ = hydrazine hydrate. NR = No reaction.

Preparation of Compounds 18 and 19

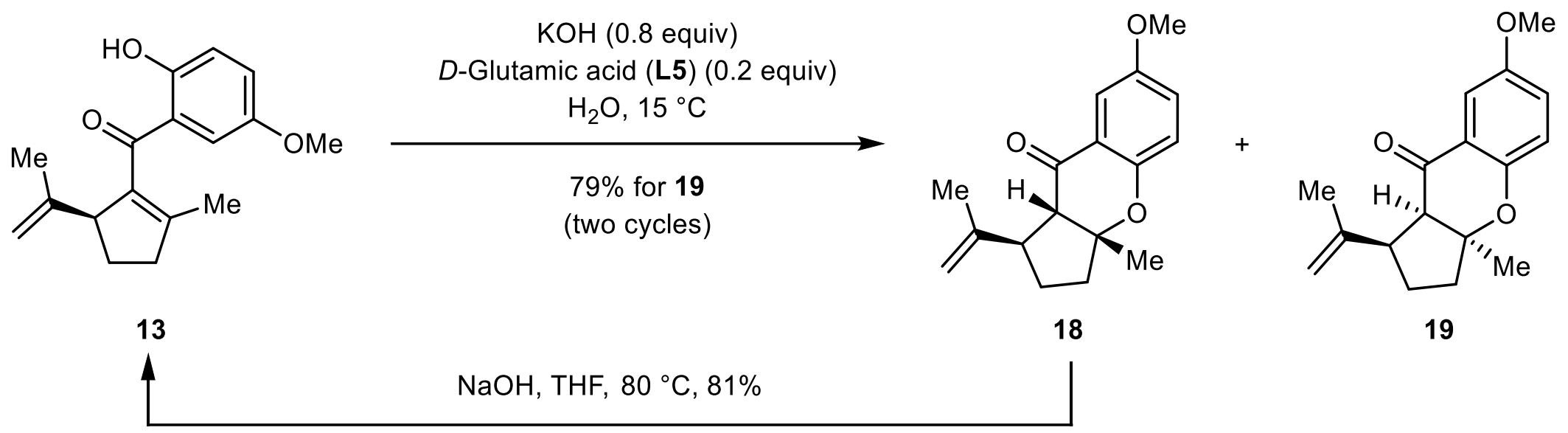

To a suspension of **13** (1.0 g, 3.68 mmol, 1.0 equiv) in H_2O (18.4 mL) were added a solution of KOH (165 mg, 2.94 mmol, 0.8 equiv) in H_2O (18.4 mL) and *D*-glutamic acid (**L5**) (108 mg, 0.735 mmol, 0.2 equiv) at 15 °C. After stirring at same temperature for 59 h, the reaction was quenched with saturated aqueous NH_4Cl solution. The reaction mixture was extracted with EtOAc and the combined organic layers were washed with brine, dried over Na_2SO_4, filtered and concentrated under reduced pressure. The residue was purified by silica gel column chromatography (petroleum ether:EtOAc = 60:1→20:1) to afford desired **19** (620 mg, 62% yield) as a yellow solid and **18** (344 mg, 34% yield) as a yellow solid.

To a solution of **18** (344 mg, 1.26 mmol,1.0 equiv) in THF (12.6 mL) was added NaOH (212 mg, 5.31 mmol, 4.2 equiv) at 33 °C. After stirring under reflux for 1.5 h, the reaction mixture was cooled to 33 °C and quenched with saturated aqueous NH_4Cl solution. The reaction mixture was extracted with EtOAc and the combined organic layers were washed with brine, dried over Na_2SO_4, filtered and concentrated under reduced pressure. The residue was purified by silica gel column chromatography (petroleum ether:EtOAc = 20:1) to afford desired **13** (278 mg, 81% yield) as a yellow solid. The first procedure was repeated with **13** one more time to give **19** as a yellow solid (168 mg, total 788 mg, 79% overall yield).

Data for 18:

TLC: R_f = 0.4 (petroleum ether:EtOAc = 15:1).

M.p.: 62.7–63.0 °C.

$[\alpha]_D^{25}$ = –129 (c = 1.0, $CHCl_3$).

^{1}H NMR (500 MHz, $CDCl_3$) δ 7.29 (d, *J* = 3.2 Hz, 1H), 7.08 (dd, *J* = 9.0, 3.2 Hz, 1H), 6.84 (d, *J* = 9.0 Hz, 1H), 4.78 (s, 1H), 4.77 (s, 1H), 3.78 (s, 3H), 3.00 (dt, *J* = 11.2, 8.7 Hz, 1H), 2.57 (d, *J* = 11.6 Hz, 1H), 2.28 – 2.14 (m, 2H), 1.94 – 1.84 (m, 1H), 1.80 – 1.68 (m, 1H), 1.76 (s, 3H), 1.41 (s, 3H).

^{13}C NMR (125 MHz, $CDCl_3$) δ 194.1, 153.8, 153.7, 144.5, 125.5, 119.8, 118.0, 112.3, 107.4, 91.0, 58.9, 55.8, 50.8, 39.0, 29.1, 21.8, 19.3.

HRMS (ESI)$^+$ calcd for $C_{17}H_{20}O_3Na^+$ $[M+Na]^+$: 295.1305; found: 295.1303.

FT-IR (cm^{-1}): 2966, 1679, 1618, 1486, 1466, 1283, 1219, 1035, 894.

Data for 19:

TLC: R_f = 0.3 (petroleum ether:EtOAc = 15:1).

M.p.: 63.3–63.4 °C.

$[\alpha]_D^{25}$ = +98 (c = 1.0, $CHCl_3$).

^{1}H NMR (500 MHz, $CDCl_3$) δ 7.23 (d, *J* = 3.2 Hz, 1H), 7.04 (dd, *J* = 9.0, 3.2 Hz, 1H), 6.80 (d, *J* = 9.0 Hz, 1H), 4.70 (s, 1H), 4.68 (s, 1H), 3.77 (s, 3H), 3.25 (dt, *J* = 11.9, 8.8 Hz, 1H), 2.86 (d, *J* = 12.0 Hz, 1H), 2.24 (ddd, *J* = 13.3, 7.3, 1.7 Hz, 1H), 2.19 – 2.08 (m, 1H), 2.01 – 1.93 (m, 1H), 1.85 – 1.71 (m, 1H), 1.53 (s, 3H), 1.42 (s, 3H).

^{13}C NMR (125 MHz, $CDCl_3$) δ 193.7, 154.9, 153.7, 144.7, 125.4, 120.5, 119.8, 113.5, 106.8, 89.3, 58.4, 55.8, 50.4, 40.6, 30.3, 23.5, 23.0.

HRMS(ESI)$^+$ calcd for $C_{17}H_{20}O_3Na^+$ $[M+Na]^+$: 295.1305; found: 295.1307.

FT-IR (cm^{-1}): 2925, 2854, 1676, 1487, 1431, 1279, 1036, 826.

Supplementary Table 3. Optimization of Michael addition of compound 19

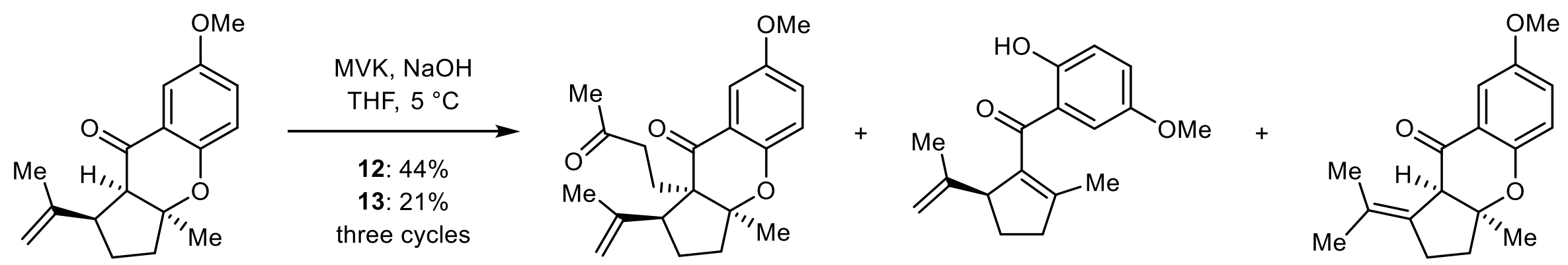

19 **12** **13** **S9**

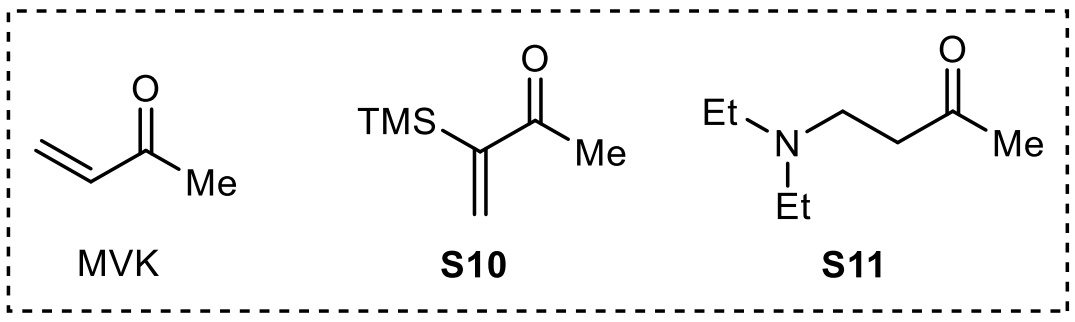

Entry	Conditions[a]	12 (%)[b]	13 (%)[b]	S9 (%)[b]
1	MVK, H_2SO_4, benzene, 33 to 60 °C	0	0	82
2	MVK, *p*-TsOH•H_2O, toluene, 33 to 60 °C	0	0	99
3	MVK, $TiCl_4$, DCM, 0 to 60 °C	0	0	72
4	MVK, BF_3•Et_2O, DCM, 0 to 33 °C	0	0	0
5[d]	MVK, LDA, THF, 0 °C	0	trace	0
6[d]	MVK, LiHMDS, THF, 33 °C	0	0	0
7[d]	MVK, DBU, THF, 50 °C	0	trace	0
8[d]	MVK, NaOMe, MeOH, 33 °C	0	trace	0
9[d]	MVK, EtONa, EtOH, 0 °C	0	31	0
10	MVK, *t*-BuOK, THF, 0 °C	0	80	0
11	MVK, *t*-BuOK,18-Crown-6, THF, 0 °C	0	84	0
12	MVK, *t*-BuOLi, THF, 0 to 33 °C		complex mixture	
13	MVK, Piperidine, toluene, 60 °C		NR	
14	MVK, TMG, DCM, 55 °C		NR	
15	MVK, Et_2NH, CH_3CN, 80 °C		NR	
16	MVK, HMDS, DCM, 0 to 60 °C		NR	
17	MVK, K_2CO_3, THF, 60 °C		NR	
18[c,e,f]	MVK, NaOH, THF, 5 °C	44	21	0
19[d]	**S10**, LDA, THF, –78 to 33 °C	0	<10	0
20[c]	**S10**, KOH, THF, –20 to 33 °C	16	26	0
21[c,d]	**S11**, KOH, THF/PhCl, –20 to 33 °C	<5	17	0
22	**S11**, Et_3N, benzene, 100 °C		NR	

[a]all reactions were performed on a 10.0 mg scale in 0.4 mL solvent except as otherwise indicated. [b]Yield determined by ^1H NMR with CH_2Br_2 as an internal standard. [c]Isolated yield. [d]A large amount of **19** remains. [e]**19** (1.0 g). [f]Yield of three cycles. MVK = methyl vinyl ketone. *p*-TsOH•H_2O = *p*-toluenesulfonic acid monohydrate. LDA = lithium diisopropylamide. LiHMDS = lithium bis(trimethylsilyl)amide. DBU = 1,8-diazabicyclo[5.4.0]undec-7-ene. TMG = 1,1,3,3-tetramethylguanidine. HMDS = hexamethyldisilazane.

Supplementary Table 4. Optimization of the Michael addition of compound 19 with MVK

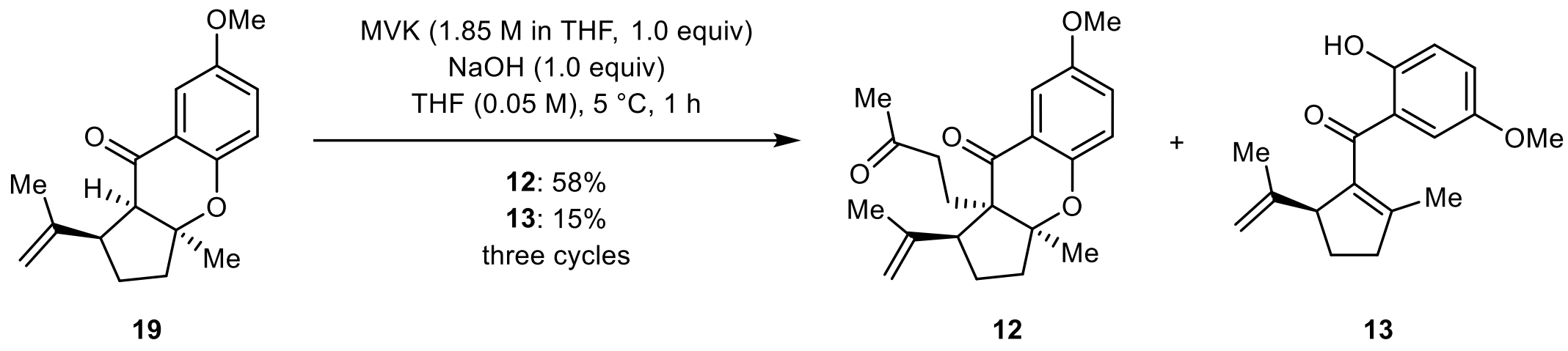

Entry	Conditions[a]	12 (%)[b]	13 (%)[b]	19 (%)[b]
1[c]	MVK (1.85 M in THF, 1.0 equiv), NaOH (0.5 equiv), THF (0.1 M), 5 °C, 1 h	38	12	23
2[d]	MVK (0.5 M in THF, 1.0 equiv), NaOH (0.5 equiv), THF (0.1 M), 5 °C, 1 h (Add dropwise via syringe pump over 20 min.)	26	12	35
3[d]	MVK (0.5 M in THF, 1.0 equiv), NaOH (0.5 equiv), THF (0.1 M), 5 °C, 1 h (Add dropwise via syringe pump over 40 min.)	30	12	30
4[d]	MVK (1.2 equiv), NaOH (0.5 equiv), THF (0.1 M), 5 °C, 2 h (Add dropwise via syringe pump over 36 min.)	33	23	11
5[d]	MVK (1.85 M in THF, 1.0 equiv), NaOH (0.5 equiv), THF (0.1 M), 5 °C, 1 h (Add dropwise via syringe pump over 30 min.)	34	12	37
6[e]	MVK (1.85 M in THF, 1.0 equiv), NaOH (0.5 equiv), THF (0.1 M), 5 °C, 1 h (Add dropwise one portion via a syringe pump every 5 min.)	35	11	40
7[e]	MVK (1.85 M in THF, 1.0 equiv), NaOH (0.5 equiv), THF (0.1 M), 5 °C, 1 h (Add dropwise one portion via a syringe pump every 20 min.)	37	12	36
8[e]	MVK (1.85 M in THF, 1.0 equiv), NaOH (0.5 equiv), THF (0.05 M), 5 °C, 1 h	32	8	48
9[e]	MVK (1.85 M in THF, 1.0 equiv), NaOH (1.0 equiv), THF (0.05 M), 5 °C, 1 h	34	8	47
10[e,f,g]	MVK (1.85 M in THF, 1.0 equiv), NaOH (1.0 equiv), THF (0.05 M), 5 °C, 1 h	35 (58)	7 (15)	45 (7)
11[e]	MVK (1.85 M in THF, 1.0 equiv), NaOH (1.0 equiv), THF (0.05 M), 5 °C, 2 h	36	15	33
12[e]	MVK (1.85 M in THF, 1.0 equiv), NaOH (2.0 equiv), THF (0.05 M), 5 °C, 1 h	35	10	42
13[e]	MVK (1.85 M in THF, 1.5 equiv), NaOH (1.0 equiv), THF (0.05 M), 5 °C, 1 h	38	9	27
14[e]	MVK (1.85 M in THF, 2.0 equiv), NaOH (1.0 equiv), THF (0.05 M), 5 °C, 40 min	35	7	20
15[e]	MVK (1.85 M in THF, 1.0 equiv), NaOH (1.0 equiv), THF/PhMe (0.05 M), 5 °C, 1 h	31	4	51
16[e]	MVK (1.85 M in THF, 1.0 equiv), NaOH (1.0 equiv), THF/PhCl (0.05 M), 5 °C, 1 h	31	6	52
17[e]	MVK (1.85 M in THF, 1.0 equiv), KOH (1.0 equiv), THF/PhCl (0.05 M), 5 °C, 4 h	33	14	39
18[e]	MVK (1.85 M in THF, 1.0 equiv), KOH (1.0 equiv), THF (0.05 M), 5 °C, 1 h	42	19	24

[a]all reactions were performed on a 165 mg scale in 6.1 mL solvent except as otherwise indicated. [b]Isolated yield. [c]General procedure A. [d]General procedure B. [e]General procedure C. [f]**19** (1.0 g). [g]Yield of three cycles (in parentheses).

General procedure A: To a solution of **19** (165 mg, 0.606 mmol, 1.0 equiv) in THF (6.1 mL) was added solid NaOH (12.1 mg, 0.303 mmol, 0.5 equiv) at 5 °C, and the mixture was stirred at this temperature for 5 min. A solution of MVK (1.85 M in THF, 109 μL, 0.202 mmol, 0.33 equiv) was added dropwise to the reaction mixture. After stirring for 5 min, another portion of the MVK solution (1.85 M in THF, 109 μL, 0.202 mmol, 0.33 equiv) was added dropwise to the reaction mixture. After stirring for 5 min, a third portion of the MVK solution (1.85 M in THF, 109 μL, 0.202 mmol, 0.33 equiv) was added dropwise to the reaction mixture, and stirring was continued for another 1 h at 5 °C. The reaction mixture was quenched with saturated aqueous NH_4Cl solution and extracted with EtOAc and the combined organic layers were washed with brine, dried over Na_2SO_4, filtered and concentrated under reduced pressure. The residue

was purified by silica gel column chromatography (petroleum ether:EtOAc = 60:1→30:1→8:1) to afford **12** (78.2 mg, 38% yield) as a yellow oil, **13** (20.4 mg, 12% yield) and **19** (38.0 mg, 23% yield).

General procedure B: To a solution of **19** (165 mg, 0.606 mmol, 1.0 equiv) in THF (6.1 mL) was added solid NaOH (12.1 mg, 0.303 mmol, 0.5 equiv) at 5 °C, and the mixture was stirred at this temperature for 5 min. A solution of MVK (1.85 M in THF, 327 μL, 0.606 mmol, 1.0 equiv) was added via syringe pump to the reaction mixture over 30 min, and stirring was continued for another 1 h at 5 °C. The reaction mixture was quenched with saturated aqueous NH_4Cl solution and extracted with EtOAc and the combined organic layers were washed with brine, dried over Na_2SO_4, filtered and concentrated under reduced pressure. The residue was purified by silica gel column chromatography (petroleum ether:EtOAc = 60:1→30:1→8:1) to afford **12** (70.1 mg, 34% yield) as a yellow oil, **13** (19.0 mg, 12% yield) and **19** (61.0 mg, 37% yield).

General procedure C: To a solution of **19** (165 mg, 0.606 mmol, 1.0 equiv) in THF (12.1 mL) was added solid NaOH (24.0 mg, 0.606 mmol, 1.0 equiv) at 5 °C, and the mixture was stirred at this temperature for 5 min. A solution of MVK (1.85 M in THF, 109 μL, 0.202 mmol, 0.33 equiv) was added via syringe pump to the reaction mixture over 5 min. After stirring for 5 min, another portion of the MVK solution (1.85 M in THF, 109 μL, 0.202 mmol, 0.33 equiv) was added via syringe pump to the reaction mixture over 5 min. After stirring for 5 min, a third portion of the MVK solution (1.85 M in THF, 109 μL, 0.202 mmol, 0.33 equiv) was added via syringe pump to the reaction mixture over 5 min, and stirring was continued for another 1 h at 5 °C. The reaction mixture was quenched with saturated aqueous NH_4Cl solution and extracted with EtOAc and the combined organic layers were washed with brine, dried over Na_2SO_4, filtered and concentrated under reduced pressure. The residue was purified by silica gel column chromatography (petroleum ether:EtOAc = 60:1→30:1→8:1) to afford **12** (69.9 mg, 34% yield) as a yellow oil, **13** (13.6 mg, 8% yield) and **19** (78.0 mg, 47% yield).

Preparation of Compound 12

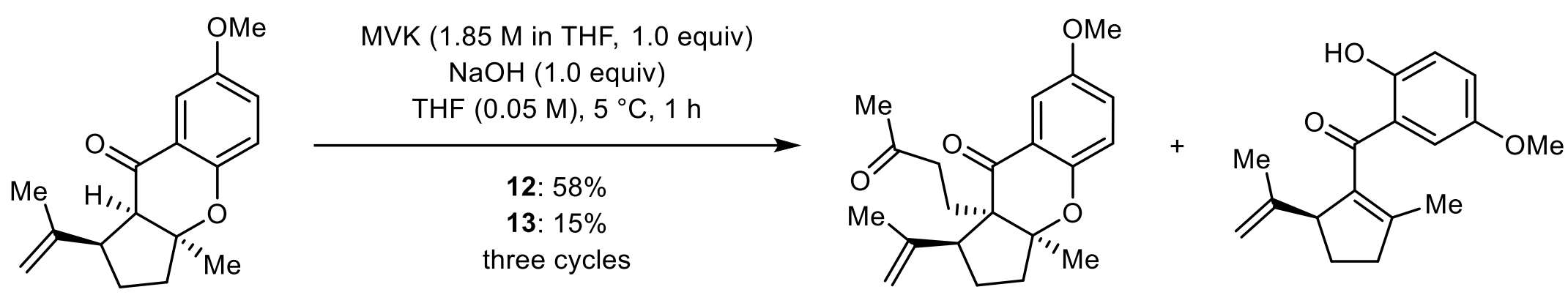

19 **12** **13**

To a solution of **19** (1.0 g, 3.68 mmol, 1.0 equiv) in THF (73.5 mL) was added solid NaOH (147 mg, 3.68 mmol, 1.0 equiv) at 5 °C, and the mixture was stirred at this temperature for 5 min. A solution of MVK (1.85 M in THF, 662 μL, 1.23 mmol, 0.33 equiv) was added via syringe pump to the reaction mixture over 5 min. After stirring for 5 min, another portion of the MVK solution (1.85 M in THF, 662 μL, 1.23 mmol, 0.33 equiv) was added via syringe pump to the reaction mixture over 5 min. After stirring for 5 min, a third portion of the MVK solution (1.85 M in THF, 662 μL, 1.23 mmol, 0.33 equiv) was added via syringe pump to the reaction mixture over 5 min, and stirring was continued for another 1 h at 5 °C. The reaction mixture was quenched with saturated aqueous NH_4Cl solution and extracted with EtOAc and the combined organic layers were washed with brine, dried over Na_2SO_4, filtered and concentrated under reduced pressure. The residue was purified by silica gel column chromatography (petroleum ether:EtOAc = 60:1→30:1→8:1) to afford **12** (441 mg, 35% yield) as a yellow oil, **13** (73.0 mg, 7% yield) and **19** (446 mg, 45% yield). This procedure was repeated another two times to give **12** as a yellow oil (294 mg, total 735 mg, 58% yield), **13** (74.3 mg, total 147 mg, 15% yield) and **19** (72.8 mg, 7% yield).

Data for 12:

TLC: R_f = 0.4 (petroleum ether:EtOAc = 5:1).

$[\boldsymbol{\alpha}]_D^{25}$ = +65 (c = 1.0, $CHCl_3$).

^{1}H NMR (500 MHz, $CDCl_3$) δ 7.18 (d, J = 3.1 Hz, 1H), 7.03 (dd, J = 9.0, 3.2 Hz, 1H), 6.77 (d, J = 9.0 Hz, 1H), 4.77 (s, 1H), 4.74 (s, 1H), 3.77 (s, 3H), 2.94 (ddd, J = 18.0, 10.6, 4.7 Hz, 1H), 2.73 (t, J = 8.8 Hz, 1H), 2.56 (ddd, J = 17.9, 10.4, 5.2 Hz, 1H), 2.17 – 2.09 (m, 1H), 2.12 (s, 3H), 2.06 – 1.92 (m, 3H), 1.92 – 1.83 (m, 2H), 1.60 (s, 3H), 1.42 (s, 3H).

^{13}C NMR (125 MHz, $CDCl_3$) δ 208.5, 196.3, 154.0, 153.9, 144.8, 125.0, 121.9, 119.9, 114.0, 107.3, 92.2, 60.5, 55.84, 55.76, 39.6, 37.8, 30.2, 29.9, 28.1, 22.9, 21.6.

HRMS$(ESI)^+$ calcd for $C_{21}H_{26}O_4Na^+$ $[M+Na]^+$: 365.1723; found: 365.1728.

FT-IR (cm^{-1}): 2952, 1716, 1670, 1488, 1429, 1277, 1153, 1034, 828.

Preparation of Compound S9

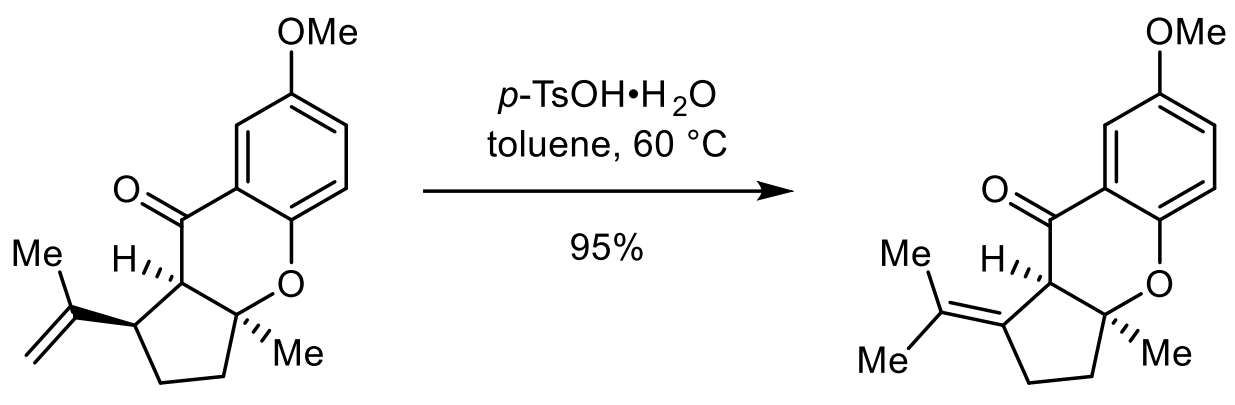

19 **S9**

To a solution of **19** (20.0 mg, 0.0735 mmol,1.0 equiv) in toluene (0.74 mL) was added *p*-TsOH•H_2O (42.0 mg, 0.221 mmol, 3.0 equiv) at 33 °C. After stirring at 60 °C for 3.5 h, the reaction was cooled down to 33 °C and quenched by saturated aqueous $NaHCO_3$ solution. The reaction mixture was extracted with EtOAc and the combined organic layers were washed with brine, dried over Na_2SO_4, filtered and concentrated under reduced pressure. The residue was purified by PTLC (petroleum ether:EtOAc = 5:1) to afford **S9** (19.0 mg, 95% yield) as a colorless oil.

TLC: R_f = 0.9 (petroleum ether:EtOAc = 5:1).

$[\alpha]_D^{24}$ = +239 (c = 0.2, $CHCl_3$).

^{1}H NMR (500 MHz, $CDCl_3$) δ 7.27 (d, *J* = 3.2 Hz, 1H), 7.07 (dd, *J* = 9.0, 3.2 Hz, 1H), 6.84 (d, *J* = 9.0 Hz, 1H), 3.78 (s, 3H), 3.37 – 3.32 (m, 1H), 2.46 – 3.36 (m, 1H), 2.36 – 2.27 (m, 1H), 2.14 (ddd, *J* = 12.8, 9.5, 6.8 Hz, 1H), 1.81 – 1.78 (m, 3H), 1.74 – 1.67 (m, 1H), 1.67 – 1.64 (m, 3H), 1.46 (s, 3H).

^{13}C NMR (125 MHz, $CDCl_3$) δ 193.3, 154.1, 153.7, 130.0, 129.6, 125.4, 119.5, 119.0, 107.6, 87.9, 57.8, 55.9, 35.5, 27.8, 24.6, 21.8, 21.6.

HRMS$(ESI)^+$ calcd for $C_{17}H_{20}O_3Na^+$ $[M+Na]^+$: 295.1305; found: 295.1308.

FT-IR (cm^{-1}): 2932, 1676, 1488, 1429, 1280, 1230, 1034, 824, 736.

Supplementary Table 5. Failed Michael addition of compound 19

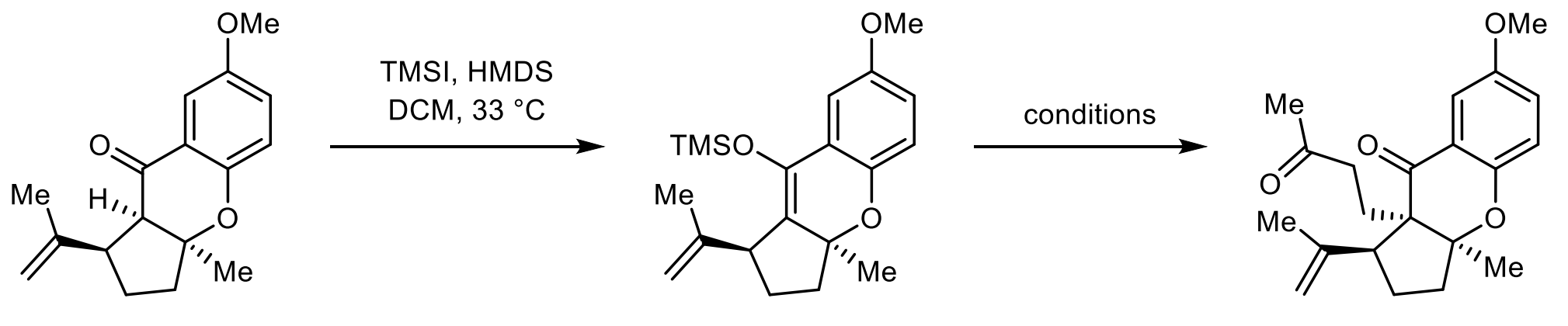

19 **S12** **12**

Entry	Conditions	Result
1	MVK, *i*-PrOH, BF_3•Et_2O, $MeNO_2$, DCM, –78 to 0 °C	NP
2	MVK, MeLi, THF, –50 to –78 °C	NP
3	MVK, $ZnCl_2$, Et_2O, 0 to 33 °C	NP

NP = no product.

***Note**: Only the hydrolysis product **19** and the subsequent retro-oxa-Michael product **13** were observed.*

Supplementary Table 6. Optimization of acid-dependent divergent cyclization of compound 12

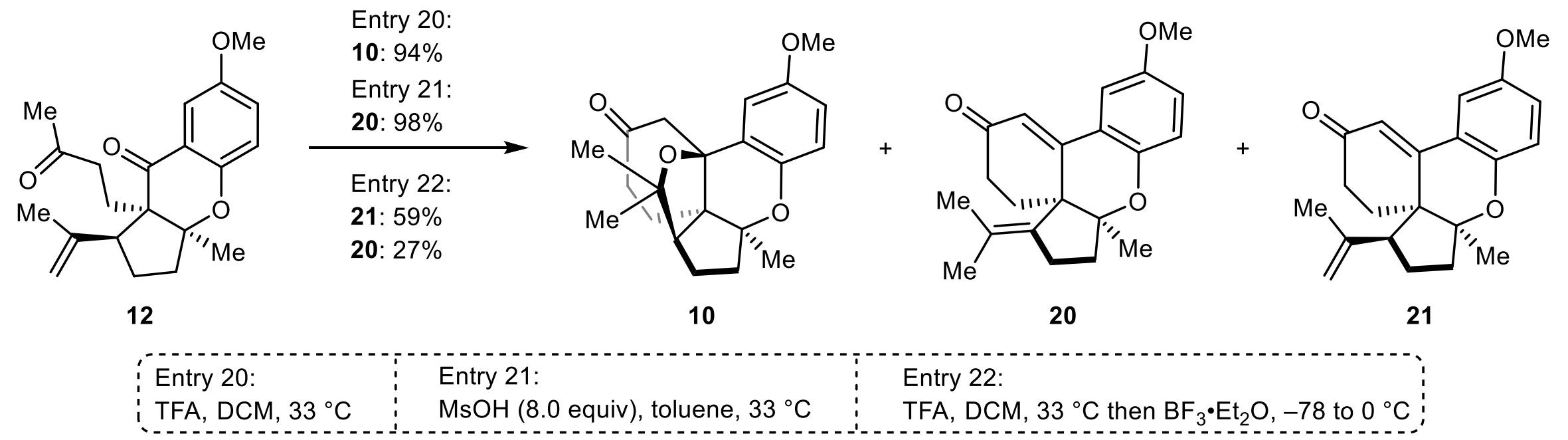

Entry	Conditions	**10** (%)[a]	**20** (%)[a]	**21** (%)[a]
1	LiHMDS, THF, –78 °C	0	0	0
2	LDA, THF, –78 to 0 °C		NR	
3	*t*-BuOK, *t*-BuOH, 33 °C	0	0	0
4	*t*-BuOK, THF, 0 °C	0	0	0
5	NaOMe, MeOH, 0 to 100 °C	0	0	0
6	NaOH, EtOH, 0 to 100 °C	0	0	0
7	KOH, MeOH, 80 °C		complex mixture	
8	LiOH, *i*-PrOH, 100 °C		NR	
9	Cs_2CO_3, CH_3CN, 100 °C	0	0	0
10	K_2CO_3, MeOH, 100 °C		NR	
11	NaH, THF, 0 to 60 °C	0	0	0
12	Morpholine, EtOH, 100 °C		NR	
13	Piperidine, AcOH, benzene, 100 °C		NR	
14	Pyrrolidine, AcOH, benzene, 100 °C		NR	
15	*L*-proline, MeOH, 100 °C		NR	
16	H_2SO_4, DCM, 33 °C	0	0	0
17	MsOH (1.7 equiv), toluene, 33 °C	37	39	17
18	BF_3•Et_2O, THF, 0 to 80 °C	52	0	0
19	*p*-TsOH•H_2O, toluene, 50 °C	81	0	0
20	TFA, DCM, 33 °C	94	0	0
21	MsOH (8.0 equiv), toluene, 33 °C	0	98	0
22	TFA, DCM, 33 °C then BF_3•Et_2O, –78 to 0 °C	0	27	59

[a]Isolated yield.

Preparation of Compound 10

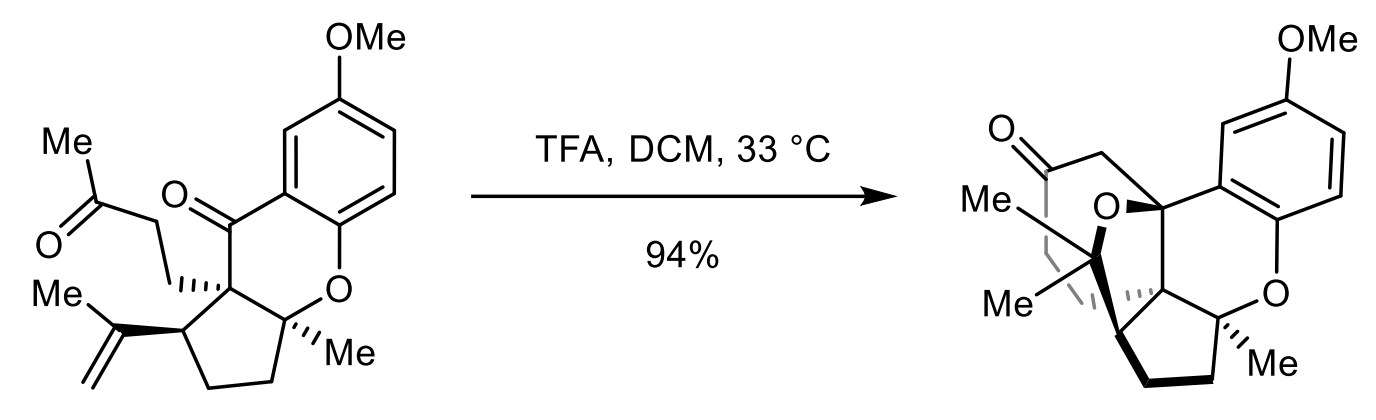

To a solution of **12** (200 mg, 0.585 mmol,1.0 equiv) in DCM (5.8 mL) was added trifluoroacetic acid (TFA, 52.0 μL, 0.701 mmol, 1.2 equiv) at 33 °C. After stirring at same temperature for 29 h, another portion of TFA (9.0 μL, 0.117 mmol, 0.2 equiv) was added at 33 °C. After stirring at same temperature for 14 h, a third portion of TFA (9.0 μL, 0.117 mmol, 0.2 equiv) was added at 33 °C. After stirring at same temperature for 5.5 h, the reaction was quenched with saturated aqueous $NaHCO_3$ solution. The reaction mixture was extracted with EtOAc and the combined organic layers were washed with brine, dried over Na_2SO_4, filtered and concentrated under reduced pressure. The residue was purified by silica gel column chromatography (petroleum ether:EtOAc = 10:1→5:1) to afford **10** (188 mg, 94% yield) as a yellow solid.

TLC: R_f = 0.7 (petroleum ether:EtOAc = 3:1).
M.p.: 142.0–143.7 °C.
$[\alpha]_D^{25}$ = +133 (c = 0.2, $CHCl_3$).
^{1}H NMR (500 MHz, $CDCl_3$) δ 6.98 (d, *J* = 2.8 Hz, 1H), 6.75 (d, *J* = 8.7 Hz, 1H), 6.72 (dd, *J* = 8.9, 2.8 Hz, 1H), 3.75 (s, 3H), 2.61 (d, *J* = 16.2 Hz, 1H), 2.57 – 2.40 (m, 4H), 2.27 (td, *J* = 13.8, 4.3 Hz, 1H), 2.07 – 1.94 (m, 1H), 1.78 (dt, *J* = 13.8, 3.5 Hz, 1H), 1.70 – 1.58 (m, 3H), 1.48 (s, 3H), 1.28 (s, 3H), 0.98 (s, 3H).
^{13}C NMR (125 MHz, $CDCl_3$) δ 210.0, 154.7, 145.4, 131.8, 119.1, 115.4, 110.5, 87.4, 82.9, 80.5, 61.5, 59.8, 55.8, 52.1, 39.1, 35.2, 31.2, 29.9, 24.7, 23.7, 23.1.
HRMS(ESI)$^+$ calcd for $C_{21}H_{27}O_4^+$ $[M+H]^+$: 343.1904; found: 343.1906.
FT-IR (cm^{-1}): 2964, 1724, 1492, 1240, 1134, 1013, 941, 815.

Preparation of Compound 20

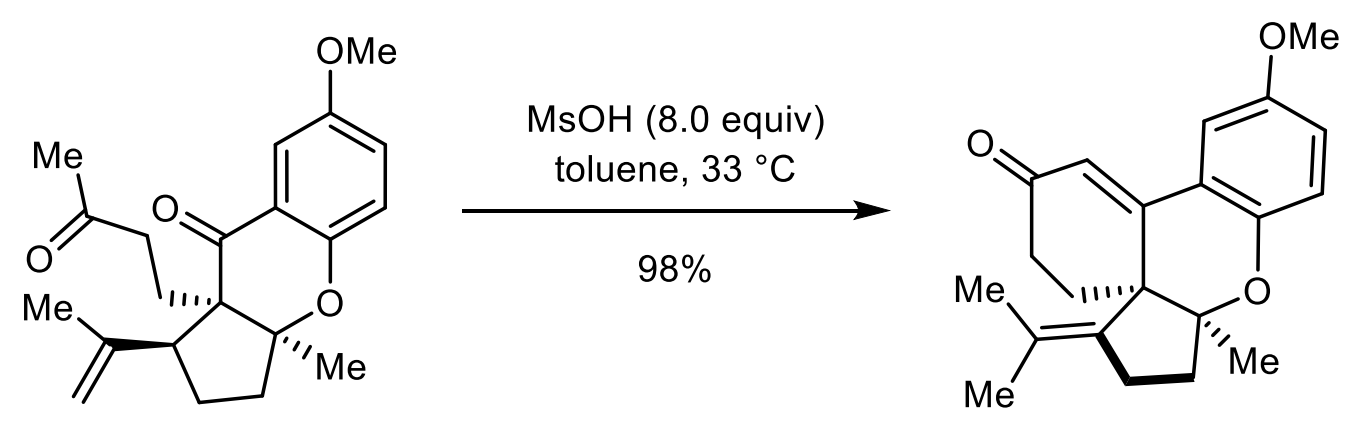

To a solution of **12** (20.0 mg, 0.0585 mmol,1.0 equiv) in toluene (0.59 mL) was added methanesulfonic acid (MsOH, 30.0 μL, 0.468 mmol, 8.0 equiv) at 33 °C. After stirring at same temperature for 1 h, the reaction was quenched with saturated aqueous $NaHCO_3$ solution. The reaction mixture was extracted with EtOAc and the combined organic layers were washed with brine, dried over Na_2SO_4, filtered and concentrated under reduced pressure. The residue was purified by silica gel column chromatography (petroleum ether:EtOAc = 8:1) to afford **20** (18.6 mg, 98%) as a yellow oil.

TLC: R_f = 0.4 (petroleum ether:EtOAc = 5:1).
$[\alpha]_D^{26}$ = –663 (c = 1.0, $CHCl_3$).

¹H NMR (400 MHz, $CDCl_3$) δ 7.01 (d, *J* = 2.9 Hz, 1H), 6.91 (dd, *J* = 8.9, 3.0 Hz, 1H), 6.71 (d, *J* = 8.9 Hz, 1H), 6.58 (s, 1H), 3.77 (s, 3H), 2.69 – 2.53 (m, 2H), 2.48 – 2.37 (m, 2H), 2.08 (ddd, *J* = 13.8, 9.2, 2.0 Hz, 1H), 2.01 – 1.90 (m, 2H), 1.83 (ddd, *J* = 12.9, 4.9, 2.9 Hz, 1H), 1.54 (s, 3H), 1.53 (s, 3H), 1.24 (s, 3H).

¹³C NMR (100 MHz, $CDCl_3$) δ 200.1, 155.4, 153.6, 149.1, 133.6, 127.3, 122.0, 121.0, 119.0, 118.0, 108.0, 91.0, 55.9, 52.0, 34.7, 34.4, 30.9, 28.4, 23.5, 19.1, 17.7.

HRMS(ESI)$^+$ calcd for $C_{21}H_{24}O_3Na^+$ $[M+Na]^+$: 347.1618; found: 347.1620.

FT-IR (cm^{-1}): 2926, 2855, 1662, 1594, 1489, 1277, 1243, 1168, 1037, 795.

Preparation of Compound 21

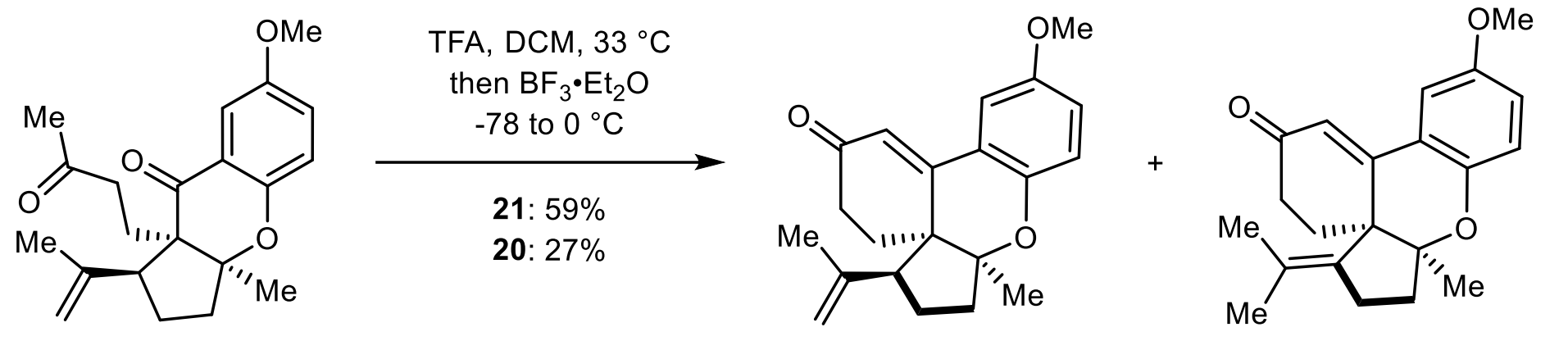

12 **21** **20**

To a solution of **12** (100 mg, 0.292 mmol, 1.0 equiv) in DCM (2.9 mL) was added TFA (26.0 µL, 0.351 mmol, 1.2 equiv) at 33 °C. After stirring at same temperature for 23 h, BF_3•Et_2O (148 µL, 1.17 mmol, 4.0 equiv) was added at –78 °C. After stirring at 0 °C for 40 min, the reaction was quenched with saturated aqueous $NaHCO_3$ solution. The reaction mixture was extracted with EtOAc and the combined organic layers were washed with brine, dried over Na_2SO_4, filtered and concentrated under reduced pressure. The residue was purified by silica gel column chromatography (petroleum ether:EtOAc = 30:1) to afford **21** (56.0 mg, 59% yield) as a yellow solid and **20** (25.4 mg, 27% yield) as a yellow oil.

Data for 21:

TLC: R_f = 0.7 (petroleum ether:EtOAc = 3:1).

M.p.: 116.9–118.3 °C.

$[\alpha]_D^{25}$ = –133 (c = 0.2, $CHCl_3$).

¹H NMR (500 MHz, $CDCl_3$) δ 7.00 (d, *J* = 2.9 Hz, 1H), 6.88 (dd, *J* = 8.9, 2.9 Hz, 1H), 6.68 (d, *J* = 8.9 Hz, 1H), 6.55 (s, 1H), 4.61 (s, 2H), 3.75 (s, 3H), 3.13 (t, *J* = 8.0 Hz, 1H), 2.75 (ddd, *J* = 18.5, 14.1, 4.8 Hz, 1H), 2.53 (ddd, *J* = 17.8, 4.8, 2.3 Hz, 1H), 2.28 – 2.12 (m, 2H), 2.10 – 1.96 (m, 3H), 1.79 (ddd, *J* = 12.8, 4.9, 2.3 Hz, 1H), 1.40 (s, 3H), 1.27 (s, 3H).

¹³C NMR (125 MHz, $CDCl_3$) δ 199.1, 153.6, 152.5, 149.4, 145.1, 121.0, 120.6, 119.4, 114.4, 106.5, 89.0, 55.7, 52.9, 51.5, 37.9, 33.9, 33.4, 28.0, 23.0, 19.2 (one signal is missing due to insufficient relaxation time).

HRMS(ESI)$^+$ calcd for $C_{21}H_{25}O_3^+$ $[M+H]^+$: 325.1798; found: 325.1799.

FT-IR (cm^{-1}): 2925, 2853, 1659, 1589, 1491, 1240, 1167, 1039, 819.

Preparation of Compound S13

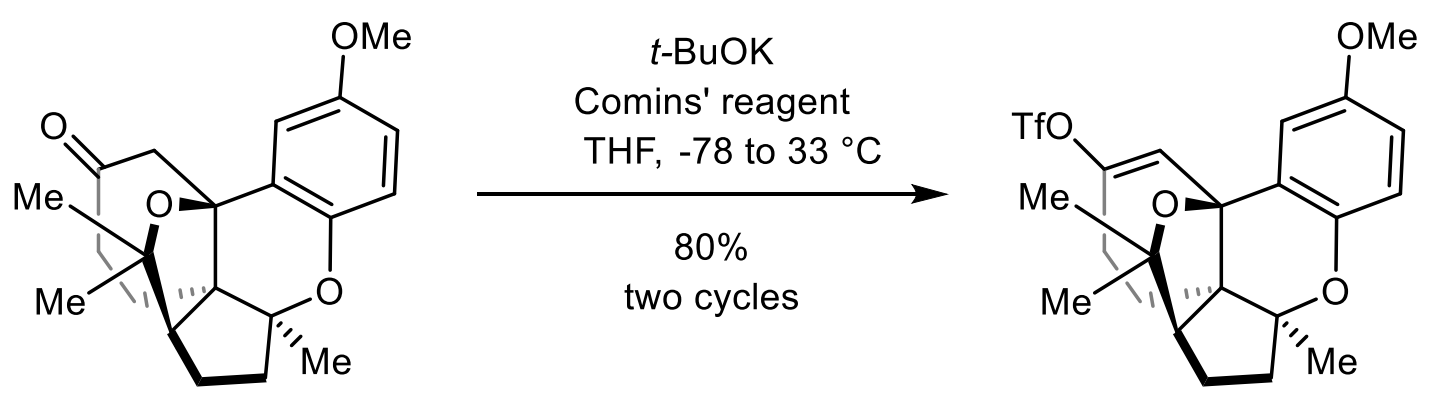

10 **S13**

To a solution of **10** (50.0 mg, 0.146 mmol,1.0 equiv) in THF (1.5 mL) was added *t*-BuOK (1.0 M in THF, 0.18 mL, 0.175 mmol, 1.2 equiv) at –78 °C. The resulting solution was stirred at –78 °C for 10 min. A solution of Comins' reagent (68.8 mg, 0.175 mmol, 1.2 equiv)

in THF (0.75 mL) was added to the reaction mixture at –78 °C. After stirring at 33 °C for 1.5 h, the reaction was quenched by saturated aqueous NH_4Cl solution. The reaction mixture was extracted with EtOAc and the combined organic layers were washed with brine, dried over Na_2SO_4, filtered and concentrated under reduced pressure. The residue was purified by silica gel column chromatography (petroleum ether:EtOAc = 50:1→30:1→5:1) to afford **S13** (41.8 mg, 60% yield) as a yellow oil and **10** (17.0 mg, 34% yield). This procedure was repeated one more time to give **S13** as a yellow oil (13.8 mg, total 55.6 mg, 80% yield).

TLC: R_f = 0.8 (petroleum ether:EtOAc = 9:1).
$[\alpha]_D^{25}$ = +27 (c = 0.2, $CHCl_3$).
^{1}H NMR (500 MHz, C_6D_6) δ 7.09 (d, *J* = 2.9 Hz, 1H), 6.82 (d, *J* = 8.7 Hz, 1H), 6.60 (dd, *J* = 8.7, 3.0 Hz, 1H), 6.04 (s, 1H), 3.30 (s, 3H), 2.22 – 2.08 (m, 2H), 2.02 – 1.93 (m, 2H), 1.56 – 1.48 (m, 1H), 1.45 (ddd, *J* = 14.0, 7.0, 4.8 Hz, 1H), 1.41 – 1.27 (m, 3H), 1.22 (s, 3H), 1.09 (s, 3H), 1.05 (s, 3H).
^{13}C NMR (125 MHz, C_6D_6) δ 155.3, 149.5, 147.4, 131.3, 123.1, 119.7, 119.1 (q, *J* = 318.6 Hz), 114.8, 111.9, 89.1, 83.4, 79.5, 60.8, 60.7, 55.2, 40.3, 32.6, 30.5, 26.0, 25.8, 24.5, 23.4.
^{19}F NMR (471 MHz, $CDCl_3$) δ –73.70.
HRMS(ESI)$^+$ calcd for $C_{22}H_{25}F_3O_6SNa^+$ [M+Na]$^+$: 497.1216; found: 497.1220.
FT-IR (cm^{-1}): 2922, 2850, 1491, 1419, 1212, 1141, 882, 758.

Preparation of Compound 22

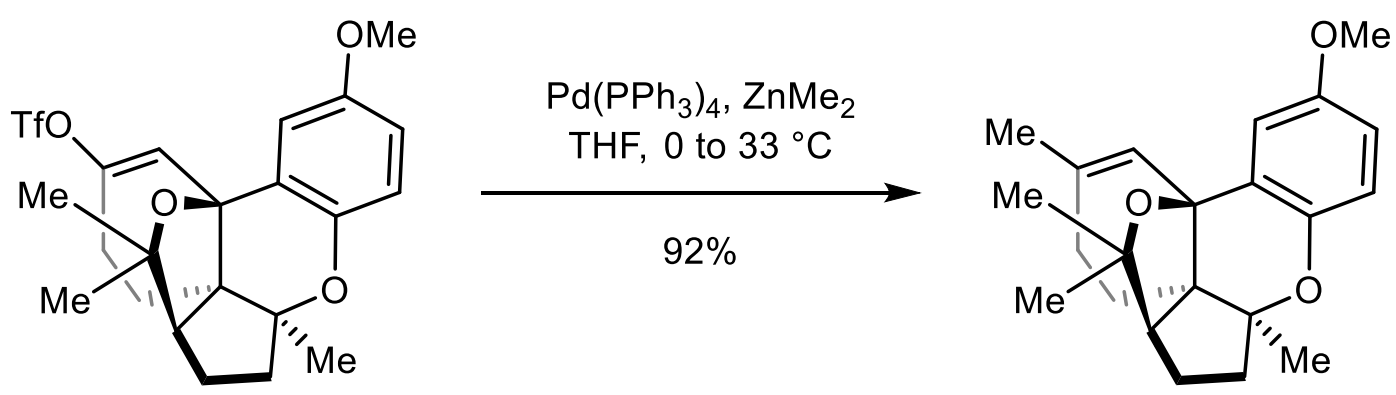

S13 **22**

To a suspension of anhydrous $ZnCl_2$ (546 mg, 4.01 mmol) in THF (8.0 mL) was added MeMgBr (3.0 M in Et_2O, 3.0 mL, 9.0 mmol) at 0 °C, and the mixture was stirred at this temperature for 1 h. The solids were allowed to settle before the solution was used[13]. To a solution of **S13** (26.7 mg, 0.0563 mmol,1.0 equiv) in THF (0.56 mL) was added $Pd(PPh_3)_4$ (13.0 mg, 0.0113 mmol, 0.2 equiv) at 0 °C, and stirred at this temperature for 5 min, freshly prepared $ZnMe_2$ (0.364 M in THF, 1.5 mL, 0.563 mmol, 10.0 equiv) was added. After stirring at 33 °C for 1.5 h, the reaction was quenched by careful addition of pH 7 buffer at 0 °C. The reaction mixture was extracted with EtOAc and the combined organic layers were washed with brine, dried over Na_2SO_4, filtered and concentrated under reduced pressure. The residue was purified by silica gel column chromatography (petroleum ether:EtOAc = 50:1→20:1) to afford **22** (17.6 mg, 92% yield) as a yellow oil.

TLC: R_f = 0.7 (petroleum ether:EtOAc = 9:1).
$[\alpha]_D^{25}$ = +40 (c = 0.2, $CHCl_3$).
^{1}H NMR (500 MHz, $CDCl_3$) δ 7.07 (d, *J* = 2.9 Hz, 1H), 6.72 (d, *J* = 8.7 Hz, 1H), 6.67 (dd, *J* = 8.7, 2.9 Hz, 1H), 5.49 – 5.45 (m, 1H), 3.78 (s, 3H), 2.42 – 2.36 (m, 1H), 2.24 – 2.06 (m, 2H), 1.98 – 1.88 (m, 2H), 1.82 – 1.74 (m, 1H), 1.72 – 1.60 (m, 3H), 1.69 (s, 3H), 1.38 (s, 3H), 1.31 (s, 3H), 1.12 (s, 3H).
^{13}C NMR (125 MHz, $CDCl_3$) δ 154.5, 147.1, 135.6, 133.7, 126.5, 118.9, 113.6, 111.8, 89.1, 82.5, 78.4, 62.5, 59.8, 55.9, 40.9, 33.0, 31.3, 28.2, 25.9, 24.5, 23.8, 23.7.
HRMS(ESI)$^+$ calcd for $C_{22}H_{29}O_3^+$ [M+H]$^+$: 341.2111; found: 341.2111.
FT-IR (cm^{-1}): 3053, 2924, 1491, 1265, 1147, 1042, 742.

Preparation of Compound S14

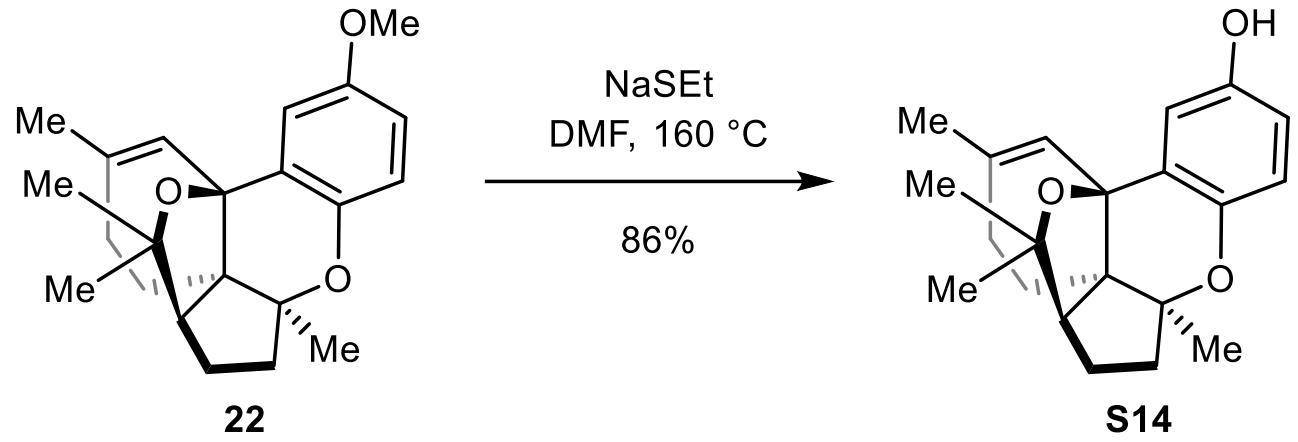

To a solution of **22** (5.0 mg, 0.0147 mmol, 1.0 equiv) in DMF (0.2 mL) was added a suspension of NaSEt (29.0 mg, 0.2939 mmol, 20.0 equiv) in DMF (0.4 mL) at 33 °C, the resulting mixture was degassed by freeze-pump-thaw cycling (three times). After stirring at 160 °C for 12 h, the reaction mixture was cooled down to 33 °C and quenched by brine. The reaction mixture was extracted with EtOAc and the combined organic layers were washed with brine, dried over Na_2SO_4, filtered and concentrated under reduced pressure. The residue was purified by PTLC (petroleum ether:EtOAc = 5:1) to afford **S14** (4.1 mg, 86% yield) as a yellow oil.

TLC: R_f = 0.2 (petroleum ether:EtOAc = 9:1).

$[\boldsymbol{\alpha}]_D^{25}$ = +56 (c = 0.2, $CHCl_3$).

^{1}H NMR (500 MHz, $CDCl_3$) δ 7.01 (d, *J* = 3.0 Hz, 1H), 6.64 (d, *J* = 8.5 Hz, 1H), 6.56 (dd, *J* = 8.5, 2.9 Hz, 1H), 5.49 (s, 1H), 5.05 (brs, 1H), 2.44 – 2.38 (m, 1H), 2.21 – 2.09 (m, 2H), 1.99 – 1.86 (m, 2H), 1.81 – 1.56 (m, 4H), 1.69 (s, 3H), 1.35 (s, 3H), 1.32 (s, 3H), 1.15 (s, 3H).

^{13}C NMR (125 MHz, $CDCl_3$) δ 150.3, 147.1, 135.9, 133.4, 126.3, 119.1, 114.9, 113.5, 89.3, 82.7, 78.4, 62.2, 59.9, 40.9, 32.9, 31.1, 28.1, 25.9, 24.6, 23.8, 23.5.

HRMS$(ESI)^+$ calcd for $C_{21}H_{27}O_3^+$ $[M+H]^+$: 327.1955; found: 327.1960.

FT-IR (cm^{-1}): 3305, 2967, 2928, 1492, 1447, 1276, 1261, 971, 750.

Preparation of (–)-ganocin A (1)

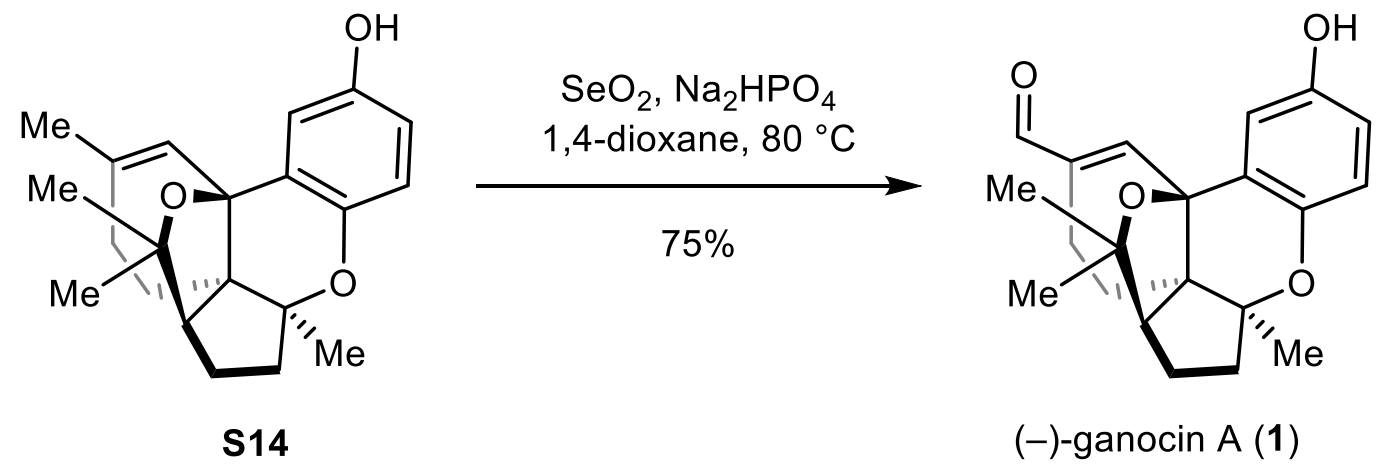

To a solution of **S14** (4.8 mg, 0.0148 mmol, 1.0 equiv) in 1,4-dioxane (0.2 mL) were added SeO_2 (8.2 mg, 0.0740 mmol, 5.0 equiv) and Na_2HPO_4 (10.5 mg, 0.0740 mmol, 5.0 equiv) at 33 °C. After stirring at 80 °C for 3 h, another portion of SeO_2 (8.2 mg, 0.0740 mmol, 5.0 equiv) and Na_2HPO_4 (10.5 mg, 0.0740 mmol, 5.0 equiv) was added at 33 °C. After stirring at 80 °C for 1.5 h, a third portion of SeO_2 (4.9 mg, 0.0444 mmol, 3.0 equiv) and Na_2HPO_4 (6.3 mg, 0.0444 mmol, 3.0 equiv) was added at 33 °C. After stirring at 80 °C for 30 min, the reaction mixture was cooled to 33 °C and filtered through a pad of celite. The filtrate was concentrated under reduced pressure. The residue was purified by PTLC (petroleum ether:EtOAc = 2:1) to afford (–)-ganocin A (**1**) (3.8 mg, 75% yield) as a colorless oil.

TLC: R_f = 0.5 (petroleum ether:EtOAc = 2:1).

$[\boldsymbol{\alpha}]_D^{26}$ = –8 (c = 0.1, $CHCl_3$).

^{1}H NMR (500 MHz, $CDCl_3$) δ 9.44 (s, 1H), 7.03 (d, *J* = 2.8 Hz, 1H), 6.70 (d, *J* = 8.5 Hz, 1H), 6.66 (dd, *J* = 8.6, 2.9 Hz, 1H), 6.64 – 6.61 (m, 1H), 2.44 – 2.38 (m, 2H), 2.25 – 2.16 (m, 1H), 2.16 – 2.06 (m, 2H), 1.76 – 1.64 (m, 3H), 1.62 – 1.56 (m, overlap with H_2O, 1H), 1.45 (s, 3H), 1.32 (s, 3H), 1.18 (s, 3H).

^{13}C NMR (125 MHz, $CDCl_3$) δ 193.5, 151.1, 150.3, 146.8, 139.3, 130.5, 119.5, 116.0, 113.5, 88.6, 84.0, 78.1, 62.2, 60.7, 39.5, 32.6, 31.1, 25.8, 24.0, 23.5, 19.3.

HRMS(ESI)$^+$ calcd for $C_{21}H_{24}O_4Na^+$ $[M+Na]^+$: 363.1567; found: 363.1566.
FT-IR (cm^{-1}): 3345, 2923, 1685, 1493, 1449, 1182, 1144, 878, 790.

Preparation of (–)-ganocin C (3)

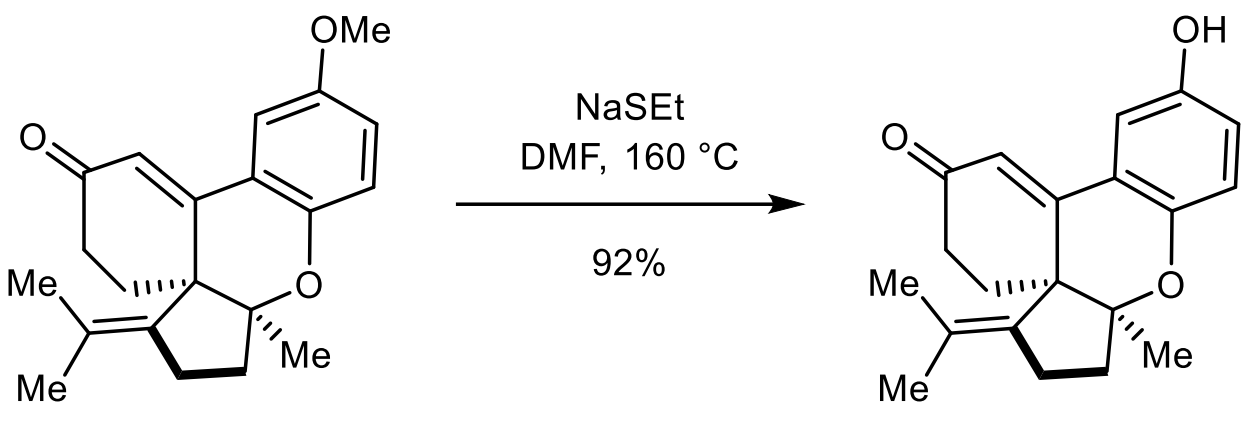

20 (–)-ganocin C (**3**)

To a solution of **20** (20.0 mg, 0.0617 mmol, 1.0 equiv) in DMF (0.2 mL) was added a suspension of NaSEt (109 mg, 1.23 mmol, 20.0 equiv) in DMF (0.4 mL) at 33 °C, the resulting mixture was degassed by freeze-pump-thaw cycling (three times). After stirring at 160 °C for 7 h, the reaction was cooled down to 33 °C and quenched by brine. The reaction mixture was extracted with EtOAc and the combined organic layers were washed with brine, dried over Na_2SO_4, filtered and concentrated under reduced pressure. The residue was purified by silica gel column chromatography (petroleum ether:EtOAc = 3:1) to afford (–)-ganocin C (**3**) (17.6 mg, 92% yield) as a yellow foam.

TLC: R_f = 0.2 (petroleum ether:EtOAc = 3:1).
$[\alpha]_D^{24}$ = –288 (c = 0.13, MeOH).
^{1}H NMR (500 MHz, C_5D_5N) δ 7.64 (d, *J* = 2.9 Hz, 1H), 7.15 (dd, *J* = 8.8, 2.9 Hz, 1H), 6.97 (s, 1H), 6.90 (d, *J* = 8.8 Hz, 1H), 2.68 – 2.56 (m, 2H), 2.49 (dt, *J* = 16.3, 3.5 Hz, 1H), 2.36 – 2.28 (m, 1H), 2.06 (ddd, *J* = 13.7, 9.3, 1.8 Hz, 1H), 1.92 – 1.82 (m, 2H), 1.68 (ddd, *J* = 13.0, 4.7, 2.9 Hz, 1H), 1.54 (s, 3H), 1.36 (s, 3H), 1.22 (s, 3H).
^{13}C NMR (125 MHz, C_5D_5N) δ 198.6, 154.4, 152.5, 147.9, 134.4, 126.5, 122.4, 122.0, 118.90, 118.86, 111.3, 90.5, 52.2, 34.52, 34.46, 30.9, 28.4, 23.0, 18.7, 17.3.
HRMS(ESI)$^+$ calcd for $C_{20}H_{23}O_3^+$ $[M+H]^+$: 311.1642; found: 311.1644.
FT-IR (cm^{-1}): 3304, 2917, 1641, 1585, 1490, 1458, 1170, 872, 736.

Preparation of (–)-ganocin B (2)

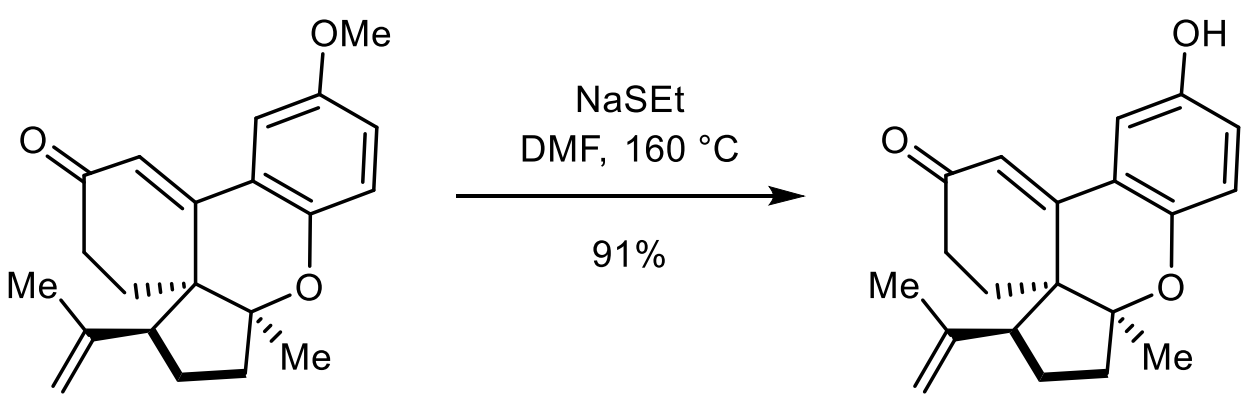

21 (–)-ganocin B (**2**)

To a solution of **21** (30.0 mg, 0.0926 mmol, 1.0 equiv) in DMF (0.2 mL) was added a suspension of NaSEt (82.0 mg, 0.926 mmol, 10.0 equiv) in DMF (0.9 mL) at 33 °C, the resulting mixture was degassed by freeze-pump-thaw cycling (three times). After stirring at 160 °C for 5.5 h, the reaction was cooled down to 33 °C and quenched by brine. The reaction mixture was extracted with EtOAc and the combined organic layers were washed with brine, dried over Na_2SO_4, filtered and concentrated under reduced pressure. The residue was purified by silica gel column chromatography (petroleum ether:EtOAc = 5:1→2:1) to afford (–)-ganocin B (**2**) (26.3 mg, 91% yield) as a yellow oil.

TLC: R_f = 0.2 (petroleum ether:EtOAc = 3:1).
$[\alpha]_D^{22}$ = –177 (c = 0.13, MeOH).

^{1}H NMR (500 MHz, C_5D_5N) δ 7.63 (d, *J* = 3.0 Hz, 1H), 7.20 (dd, *J* = 8.7, 2.8 Hz, 1H), 6.92 (d, *J* = 8.7 Hz, 1H), 6.91 (s, 1H), 4.79 (s, 1H), 4.72 – 4.68 (m, 1H), 3.12 (t, *J* = 8.1 Hz, 1H), 2.79 (ddd, *J* = 18.4, 14.0, 4.9 Hz, 1H), 2.55 (ddd, *J* = 18.0, 4.9, 2.2 Hz, 1H), 2.30 – 2.19 (m, 1H), 2.15 – 2.06 (m, 1H), 1.98 – 1.83 (m, 3H), 1.59 (ddd, *J* = 12.8, 4.9, 2.3 Hz, 1H), 1.46 (s, 3H), 1.24 (s, 3H).

^{13}C NMR (125 MHz, C_5D_5N) δ 198.0, 152.7, 151.8, 148.4, 145.9, 121.9, 120.9, 120.5, 119.4, 114.3, 109.9, 88.6, 53.1, 51.7, 37.8, 34.0, 33.6, 28.2, 22.5, 18.9.

HRMS(ESI)$^+$ calcd for $C_{20}H_{23}O_3^+$ $[M+H]^+$: 311.1642; found: 311.1645.

FT-IR (cm^{-1}): 3293, 2949, 1638, 1582, 1491, 1458, 1247, 1170, 871.

Preparation of Compound 26

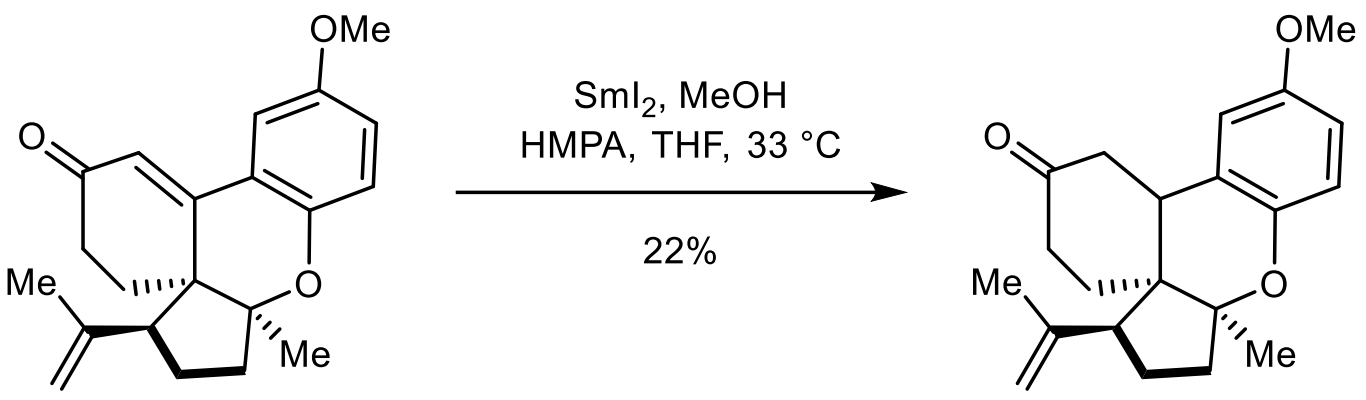

21 **26**

To a solution of **21** (5.0 mg, 0.0154 mmol, 1.0 equiv) in THF (0.2 mL) were added HMPA (27.0 μL, 0.154 mmol, 10.0 equiv) and MeOH (3.0 μL, 0.0742 mmol, 4.8 equiv) at 33 °C, the resulting mixture was degassed by freeze-pump-thaw cycling (three times). SmI_2 (0.1 M in THF, 34.0 μL, 0.0339 mmol, 2.2 equiv) was added to the reaction mixture at 33 °C. After stirring at same temperature for 40 min, the reaction was quenched with saturated aqueous NH_4Cl solution. The reaction mixture was extracted with EtOAc and the combined organic layers were washed with brine, dried over Na_2SO_4, filtered and concentrated under reduced pressure. The residue was purified by PTLC (petroleum ether:EtOAc = 5:1) to afford **26** (1.1 mg, 22% yield) as a colorless oil and **21** (1.7 mg, 34% yield).

Data for 26:

TLC: R_f = 0.6 (petroleum ether:EtOAc = 5:1).

$[\alpha]_D^{25}$ = +86 (c = 0.1, $CHCl_3$).

^{1}H NMR (500 MHz, $CDCl_3$) δ 6.72 – 6.66 (m, 2H), 6.52 – 6.49 (m, 1H), 4.69 – 4.65 (m, 2H), 3.73 (s, 3H), 3.13 (dd, *J* = 13.4, 4.8 Hz, 1H), 3.06 – 2.96 (m, 2H), 2.90 (ddd, *J* = 15.5, 4.8, 1.9 Hz, 1H), 2.80 (ddd, *J* = 16.2, 13.1, 7.0 Hz, 1H), 2.60 – 2.52 (m, 1H), 2.10 – 2.03 (m, 1H), 2.02 – 1.95 (m, 1H), 1.93 – 1.85 (m, 1H), 1.84 – 1.73 (m, 3H), 1.31 (s, 3H), 1.20 (s, 3H).

^{13}C NMR (125 MHz, $CDCl_3$) δ 210.7, 153.4, 148.5, 146.6, 124.2, 117.0, 116.8, 113.2, 109.9, 88.4, 55.9, 53.5, 47.8, 40.7, 38.3, 37.9, 37.8, 36.5, 28.5, 20.8, 20.6.

HRMS(ESI)$^+$ calcd for $C_{21}H_{26}O_3Na^+$ $[M+Na]^+$: 349.1774; found: 349.1773.

FT-IR (cm^{-1}): 3433, 2923, 2853, 1712, 1493, 1264, 1039, 803, 744.

Supplementary Table 7. Failed attempts on radical-trapping reagents

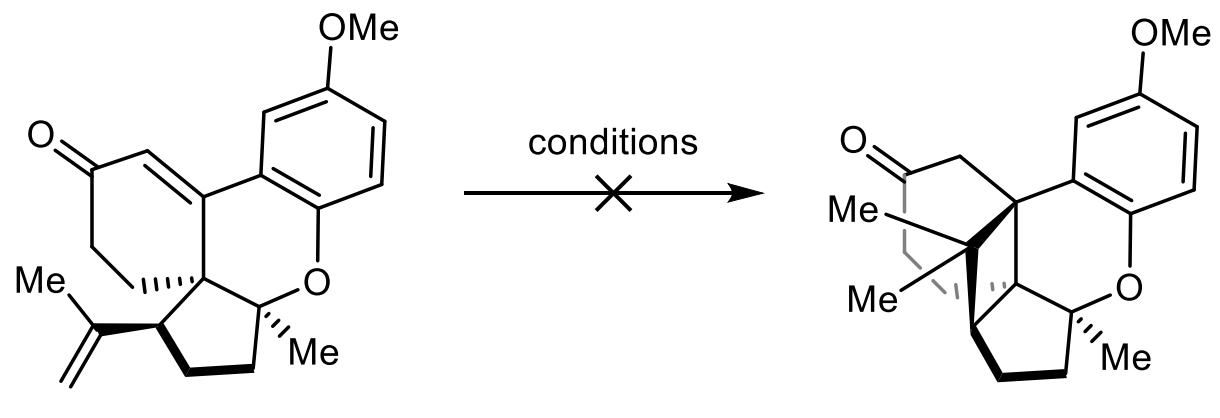

21 **24**

Entry	Conditions	Result
1	SmI_2, 4-bromobenzenethiol, HMPA, THF, 0 °C	NP
2	SmI_2, *t*-BuOH,4-bromobenzenethiol, HMPA, THF, 33 °C	NP
3	SmI_2, *t*-BuOH, 4-mercaptophenol, HMPA, THF, 33 °C	NP
4	SmI_2, *t*-BuOH, toluenethiol, HMPA, THF, 33 °C	NP
5	SmI_2, *t*-BuOH, 4-mercaptopyridine, HMPA, THF, 33 °C	NP
6	SmI_2, *t*-BuOH, CCl_4, HMPA, THF, 33 °C	NP
7	SmI_2, *t*-BuOH, $BrCCl_3$, HMPA, THF, 33 °C	NP
8	SmI_2, *t*-BuOH, C_4F_9I, HMPA, THF, 33 °C	NP

***Note**: NP = no detectable **24**. Only ketone **26** and compounds with unknown structures were observed.*

Preparation of Compound 23

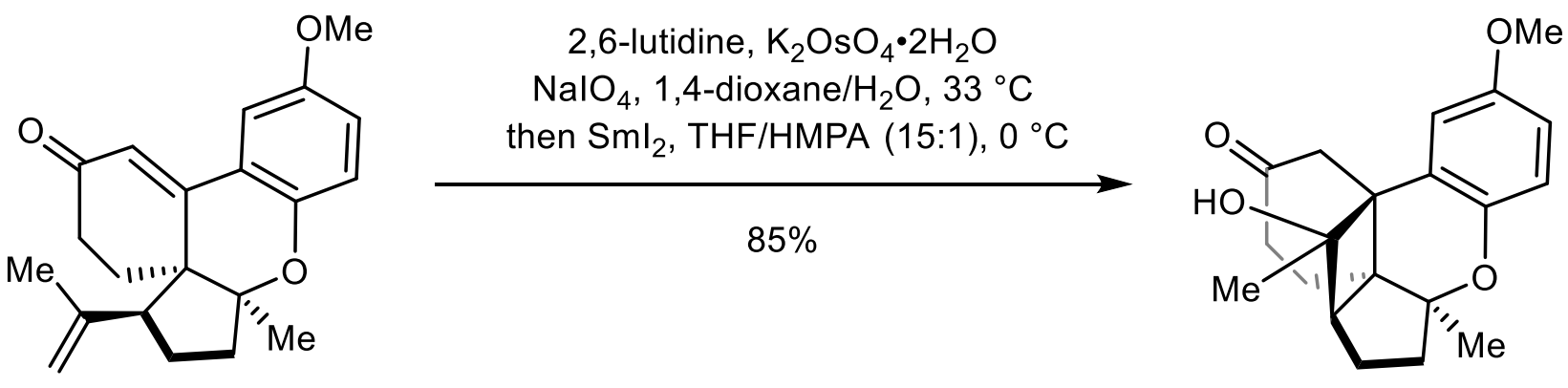

21 **23**

To a solution of **21** (160 mg, 0.494 mmol, 1.0 equiv) in 1,4-dioxane (18.5 mL) and H_2O (6.2 mL) were added 2,6-lutidine (118 μL, 0.987 mmol, 2.0 equiv), $K_2OsO_4{\cdot}2H_2O$ (18.0 mg, 0.0494 mmol, 0.1 equiv) and $NaIO_4$ (422 mg, 1.97 mmol, 4.0 equiv) at 33 °C. After stirring at same temperature for 1.5 h, the reaction mixture was concentrated under reduced pressure and diluted with EtOAc. The resulting mixture was filtered through a pad of celite. The filtrate was concentrated under reduced pressure. The crude product was evenly divided into four portions, and each portion was dissolved in THF (4.0 mL) and HMPA (0.27 mL). The resulting mixture was degassed by freeze-pump-thaw cycling (three times). SmI_2 (0.1 M in THF, 2.5 mL, 0.247 mmol, 2.0 equiv) was added to the reaction mixture at 0 °C. After stirring at same temperature for 40 min, another portion of SmI_2 (0.1 M in THF, 1.25 mL, 0.125 mmol, 1.01 equiv) was added at 0 °C. After stirring for 30 min, a third portion of SmI_2 (0.1 M in THF, 1.25 mL, 0.125 mmol, 1.01 equiv) was added at 0 °C. After stirring for 40 min, a fourth portion of SmI_2 (0.1 M in THF, 0.6 mL, 0.0617 mmol, 0.5 equiv) was added at 0 °C. After stirring at same temperature for 1 h, the reaction was quenched by saturated aqueous NH_4Cl solution. The reaction was repeated another 3 times with same scale and the combined reaction mixture was extracted with EtOAc. The combined organic layers were washed with brine, dried over Na_2SO_4, filtered and concentrated under reduced pressure. The residue was purified by silica gel column chromatography (petroleum ether:EtOAc = 50:1→20:1) to afford **23** (137mg, 85% yield) as a yellow oil.

TLC: R_f = 0.7 (petroleum ether:EtOAc = 1:1).
$[\alpha]_D^{25}$ = +5 (c = 0.2, $CHCl_3$).

^{1}H NMR (500 MHz, $CDCl_3$) δ 6.87 (d, J = 8.8 Hz, 1H), 6.75 (dd, J = 8.7, 2.9 Hz, 1H), 6.54 (d, J = 2.9 Hz, 1H), 3.76 (s, 3H), 3.11 (d, J = 16.1 Hz, 1H), 2.65 – 2.61 (m, 1H), 2.58 (d, J = 16.1 Hz, 1H), 2.50 (brs, 1H), 2.49 – 2.43 (m, 2H), 2.17 – 2.08 (m, 1H), 2.01 – 1.91 (m, 2H), 1.77 (dt, J = 14.4, 5.4 Hz, 1H), 1.73 – 1.65 (m, 2H), 1.29 (s, 3H), 1.28 (s, 3H).

^{13}C NMR (125 MHz, $CDCl_3$) δ 211.7, 155.1, 146.7, 128.6, 120.1, 113.7, 112.3, 87.5, 75.1, 55.8, 51.5, 47.6, 47.2, 47.1, 39.2, 36.2, 25.5, 25.4, 21.95, 21.93.

HRMS(ESI)$^+$ calcd for $C_{20}H_{24}O_4Na^+$ $[M+Na]^+$: 351.1567; found: 351.1568.

FT-IR (cm^{-1}): 3726, 3481, 2924, 2853, 1709, 1492, 1218, 960, 767.

Supplementary Table 8. Optimization of SmI_2-mediated 4-*exo*-trig reductive ketone–enone coupling of S15

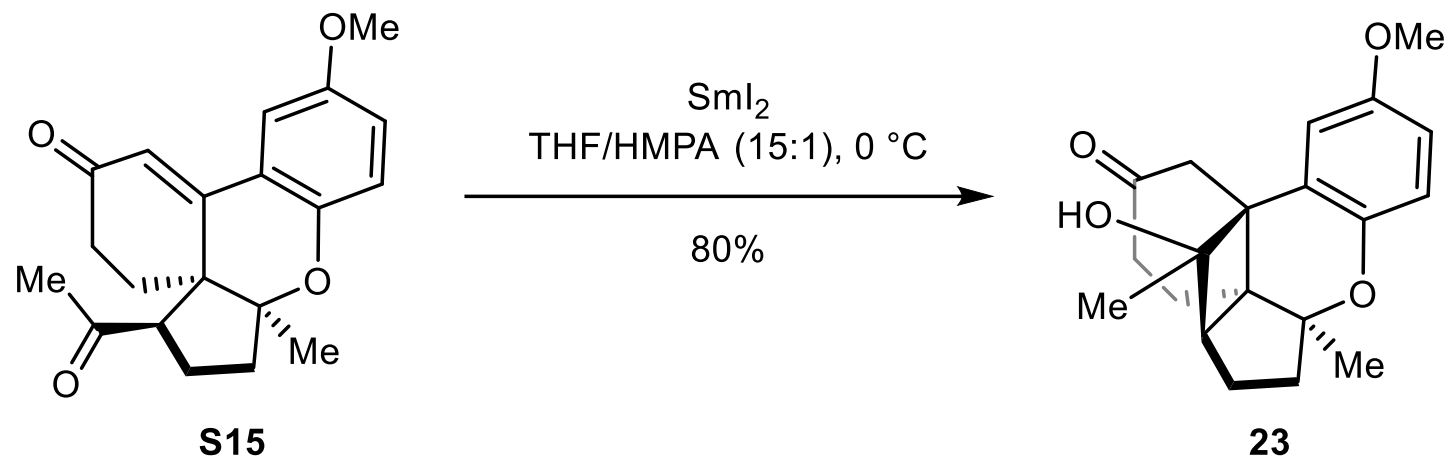

Entry	Conditions	**23** (%)^a
1	SmI_2, THF, 0 °C	42
2	SmI_2, THF/H_2O (15:1), 0 °C	36
3	SmI_2, THF/MeOH (3:1), 0 °C	33
4	SmI_2, THF/*t*-BuOH (15:1), 0 °C	46
5	SmI_2, THF/*t*-BuOH/HMPA (30:1:2), 0 °C	73
6	SmI_2, THF/HMPA (15:1), 0 °C	80

^{a}Isolated yield.

***Note**: Purified compound **S15** was employed for the optimization of the above reaction conditions.*

Preparation of Compound 27

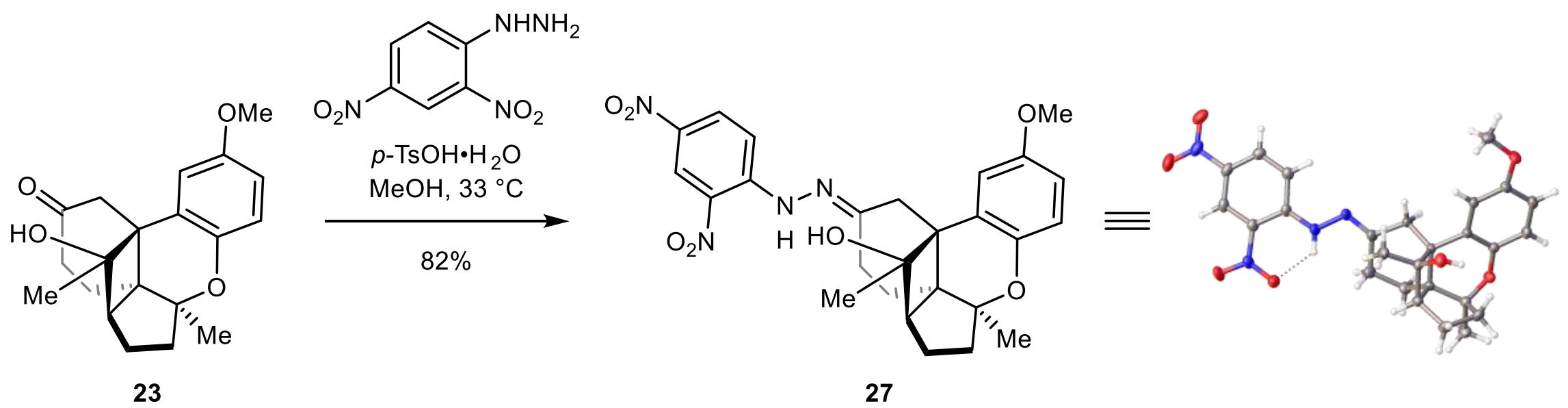

To a solution of **23** (8.0 mg, 0.0244 mmol, 1.0 equiv) in MeOH (0.41 mL) were added *p*-TsOH•H_2O (3.7 mg, 0.0195 mmol, 0.8 equiv) and 2,4-dinitrophenylhydrazine (7.2 mg, 0.0366 mmol, 1.5 equiv) at 33 °C. After stirring at same temperature for 1 h, the reaction mixture was concentrated under reduced pressure and diluted with EtOAc. The resulting mixture was quenched with saturated aqueous $NaHCO_3$ solution. The reaction mixture was extracted with EtOAc and the combined organic layers were washed with brine, dried over Na_2SO_4, filtered and concentrated under reduced pressure. The residue was purified by PTLC (petroleum ether:EtOAc = 1:1) to afford **27** (10.1 mg, 82% yield) as a red solid.

TLC: R_f = 0.8 (petroleum ether:EtOAc = 1:1).

M.p.: 216–217 °C.

$[\boldsymbol{\alpha}]_D^{23}$ = –84 (c = 0.1 $CHCl_3$).

^{1}H NMR (500 MHz, $CDCl_3$) δ 10.97 (s, 1H), 9.12 (d, *J* = 2.6 Hz, 1H), 8.29 (dd, *J* = 9.6, 2.6 Hz, 1H), 7.90 (d, *J* = 9.6 Hz, 1H), 6.85 (d, *J* = 8.7 Hz, 1H), 6.74 (dd, *J* = 8.7, 2.9 Hz, 1H), 6.70 (d, *J* = 3.0 Hz, 1H), 3.79 (s, 3H), 3.32 (d, *J* = 15.0 Hz, 1H), 2.85 (d, *J* = 15.0 Hz, 1H), 2.68 – 2.55 (m, 3H), 2.51 (dt, *J* = 18.6, 6.5 Hz, 1H), 2.16 – 2.06 (m, 1H), 2.02 – 1.91 (m, 2H), 1.89 – 1.81 (m, 1H), 1.75 – 1.65 (m, 2H), 1.40 (s, 3H), 1.28 (s, 3H).

^{13}C NMR (125 MHz, $CDCl_3$) δ 158.7, 155.2, 147.3, 145.1, 138.1, 130.2, 129.4, 128.6, 123.6, 120.1, 116.5, 113.5, 112.7, 87.6, 75.4, 55.8, 47.4, 47.3, 39.4, 38.7, 25.6, 25.2, 24.5, 22.0, 21.7 (one signal is missing due to insufficient relaxation time).

HRMS$(ESI)^+$ calcd for $C_{26}H_{29}O_7N_4^+$ $[M+H]^+$: 509.2031; found: 509.2032.

FT-IR (cm^{-1}): 3528, 3316, 2926, 1618, 1517, 1334, 1137, 832, 743.

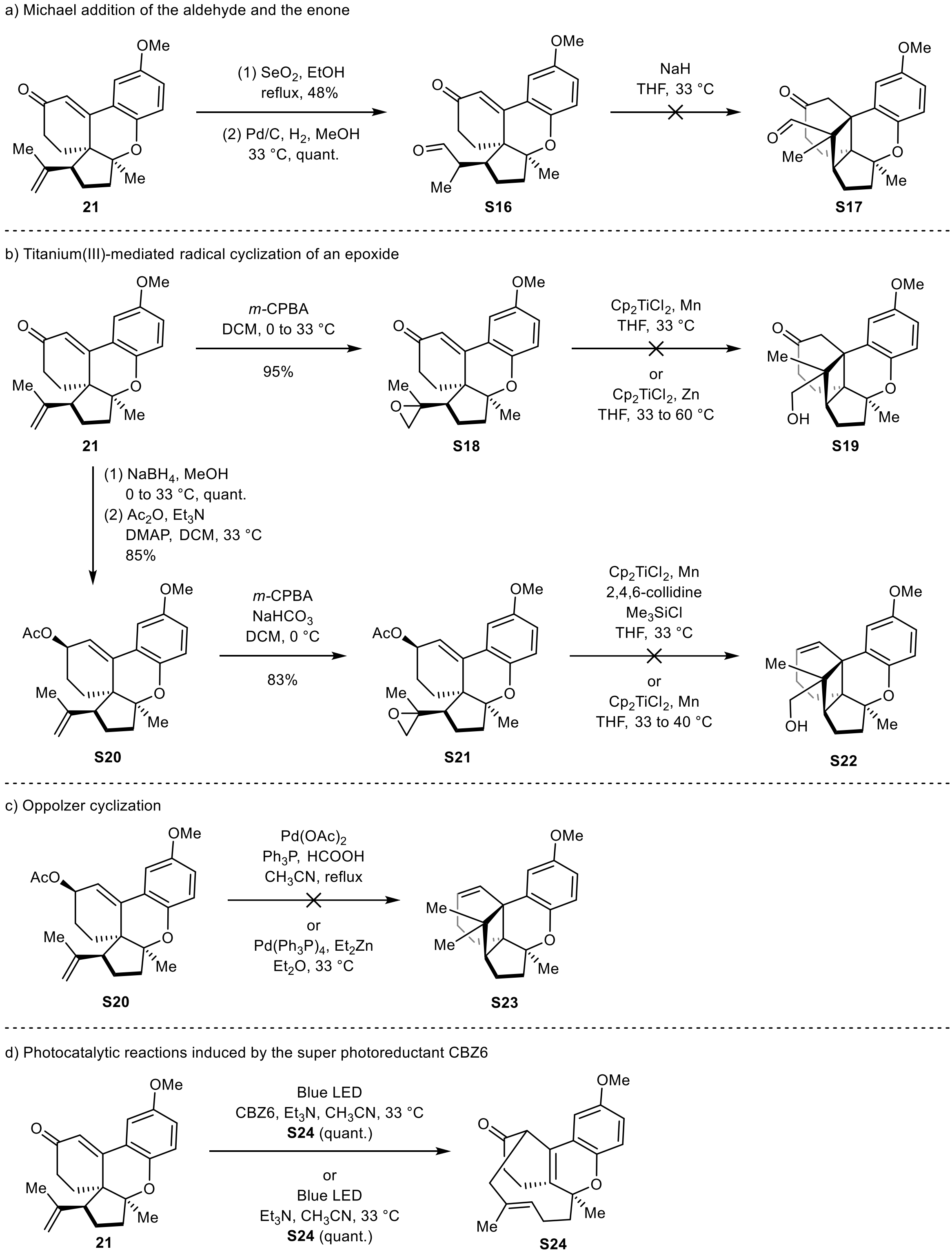

Note: *Analytical data of compound* ***S24*** *matches the literature[6].*

Supplementary Figure 2. Failed attempts. a Michael addition of the aldehyde and the enone**. b** Titanium(III)-mediated radical cyclization of an epoxide. **c** Oppolzer cyclization. **d** Photocatalytic reactions induced by the super photoreductant CBZ6.

Preparation of Compound S25

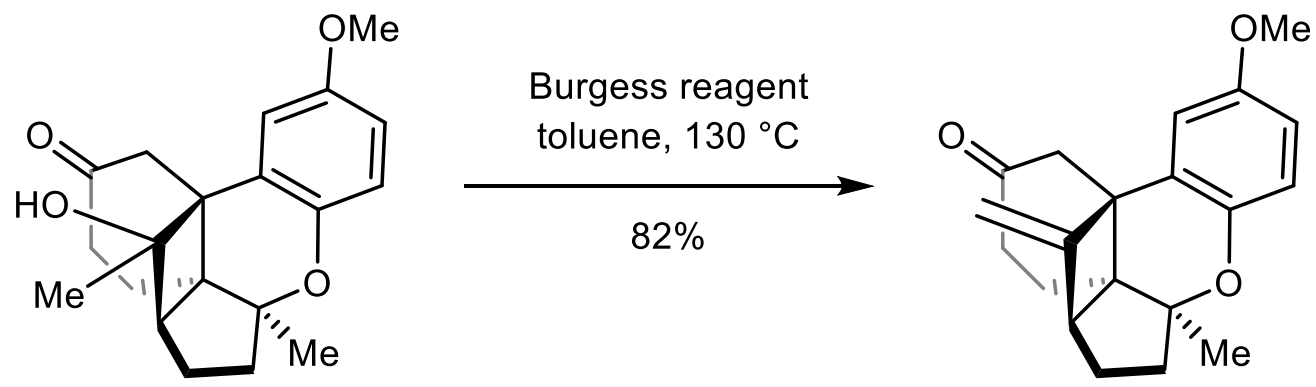

23 **S25**

To a solution of **23** (30.0 mg, 0.0914 mmol, 1.0 equiv) in toluene (0.91 mL) was added Burgess reagent (87.1 mg, 0.366 mmol, 4.0 equiv) at 33 °C. After stirring at 130 °C for 2 h, the reaction was cooled down to 33 °C and quenched by brine. The reaction was repeated another 9 times with same scale and the combined reaction mixture was extracted with EtOAc. The combined organic layers were washed with brine, dried over Na_2SO_4, filtered and concentrated under reduced pressure. The residue was purified by silica gel column chromatography (petroleum ether:EtOAc = 15:1→10:1) to afford **S25** (233 mg, 82% yield) as a yellow oil.

TLC: R_f = 0.9 (petroleum ether:EtOAc = 2:1).

$[\boldsymbol{\alpha}]_D^{26}$ = +52 (c = 0.2, $CHCl_3$).

^{1}H NMR (500 MHz, $CDCl_3$) δ 6.78 – 6.75 (m, 1H), 6.71 – 6.66 (m, 2H), 5.19 – 5.15 (m, 1H), 4.95 – 4.91 (m, 1H), 3.74 (s, 3H), 3.11 (d, *J* = 7.1 Hz, 1H), 2.84 (d, *J* = 14.9 Hz, 1H), 2.57 (d, *J* = 15.0 Hz, 1H), 2.51 (ddd, *J* = 19.6, 5.9, 3.5 Hz, 1H), 2.42 (ddd, *J* = 19.6, 11.6, 5.7 Hz, 1H), 2.29 (td, *J* = 12.7, 7.1 Hz, 1H), 2.18 (ddd, *J* = 14.2, 11.7, 5.9 Hz, 1H), 1.91 (ddd, *J* = 14.3, 5.6, 3.5 Hz, 1H), 1.79 – 1.70 (m, 1H), 1.63 (dd, *J* = 13.0, 7.1 Hz, 1H), 1.53 (dd, *J* = 12.9, 6.7 Hz, 1H), 1.39 (s, 3H).

^{13}C NMR (125 MHz, $CDCl_3$) δ 211.3, 156.7, 154.4, 145.1, 130.4, 119.1, 113.2, 111.4, 109.1, 85.9, 55.7, 51.9, 47.6, 47.1, 46.0, 36.4, 35.8, 29.5, 23.7, 23.4.

HRMS$(ESI)^+$ calcd for $C_{20}H_{23}O_3^+$ $[M+H]^+$: 311.1642; found: 311.1643.

FT-IR (cm^{-1}): 3732, 3626, 2938, 1715, 1493, 1140, 1037, 810.

Preparation of Compound S26

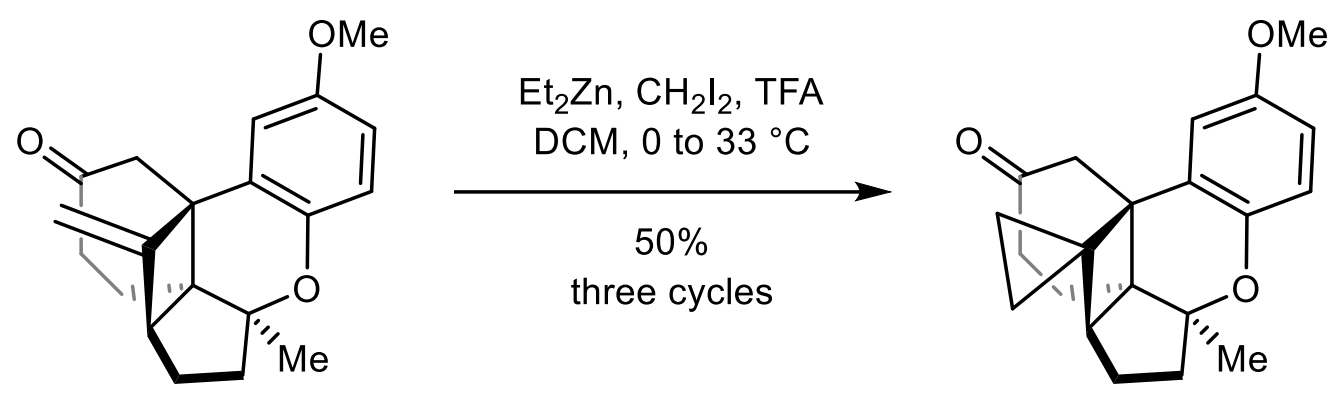

S25 **S26**

To a solution of Et_2Zn (2.0 M in hexanes, 0.6 mL, 1.19 mmol) in DCM (5.0 mL) was added slowly a solution of TFA (65.0 μL, 0.865 mmol) in DCM (2.5 mL) at 0 °C. After stirring at this temperature for 10 min, CH_2I_2 (70.0 μL, 0.865 mmol) was added and stirring was continued for another 20 min at 0 °C. To a solution of **S25** (9.0 mg, 0.0290 mmol,1.0 equiv) in DCM (0.6 mL) was added freshly prepared $CF_3CO_2ZnCH_2I$ (0.1 M in DCM, 2.9 mL, 0.290 mmol, 10.0 equiv) at 0 °C. After stirring at 33 °C for 1 h, the reaction was quenched with saturated aqueous $NaHCO_3$ solution and extracted with EtOAc. The reaction was repeated another 15 times with same scale and combined organic layers were washed with saturated aqueous $NaHCO_3$ solution and brine, dried over Na_2SO_4, filtered and concentrated under reduced pressure. The residue was purified by HPLC (SHIMADZU LC-20AR, Ultimate XB-C4, 10 × 250 mm, 5 μm, eluent A: water, eluent B: acetonitrile, gradient: T = 0 min: 30% B, T = 30 min: 60% B, T = 45 min: 70% B, 3.8 mL/min; **S25**, retention time: 30.0 min; **S26**, retention time: 31.6 min) to afford **S26** (48.0 mg, 34% yield) as a yellow solid and **S25** (63.0 mg, 44% yield). This procedure was repeated another two times to give **S26** as a yellow solid (27.5 mg, total 75.5 mg, 50% yield).

TLC: R_f = 0.7 (petroleum ether:EtOAc = 3:1).

M.p.: 141.2–143.3 °C.

$[\alpha]_D^{25}$ = +50 (c = 0.2, $CHCl_3$).

^{1}H NMR (500 MHz, $CDCl_3$) δ 6.81 (d, *J* = 8.8 Hz, 1H), 6.69 (dd, *J* = 8.8, 2.9 Hz, 1H), 6.04 (d, *J* = 2.9 Hz, 1H), 3.72 (s, 3H), 2.61 – 2.55 (m, 3H), 2.45 – 2.39 (m, 2H), 2.37 – 2.29 (m, 1H), 2.17 (ddd, *J* = 14.2, 10.1, 7.5 Hz, 1H), 1.90 (dt, *J* = 14.3, 4.5 Hz, 1H), 1.60 – 1.50 (m, overlap with H_2O, 2H), 1.41 (s, 3H), 1.36 (dd, *J* = 12.0, 7.0 Hz, 1H), 0.91 – 0.84 (m, 1H), 0.51 – 0.46 (m, 1H), 0.46 – 0.41 (m, 1H), 0.36 – 0.29 (m, 1H).

^{13}C NMR (125 MHz, $CDCl_3$) δ 212.2, 154.0, 145.4, 129.3, 119.0, 112.9, 112.0, 85.1, 55.8, 49.9, 46.8, 44.3, 40.8, 36.8, 36.1, 29.3, 26.0, 23.60, 23.56, 12.4, 7.1.

HRMS(ESI)$^+$ calcd for $C_{21}H_{24}O_3Na^+$ [M+Na]$^+$: 347.1618; found: 347.1620.

FT-IR (cm^{-1}): 2940, 2864, 1713, 1492, 1262, 1143, 1039, 804, 767.

Preparation of Compound 24

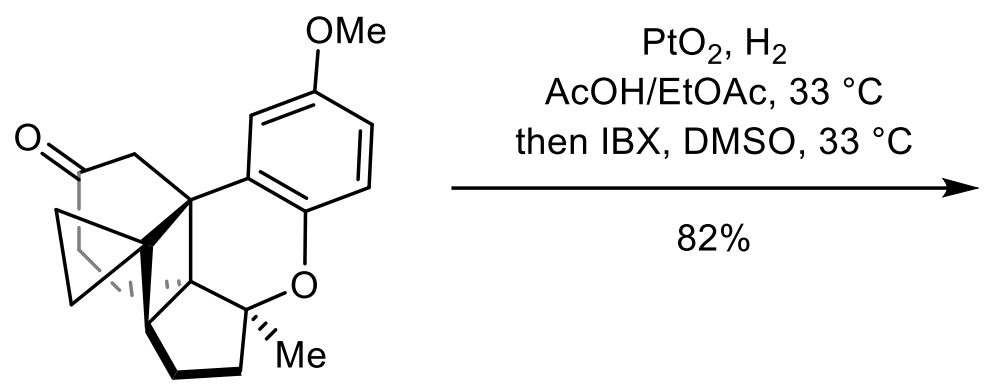

S26 **24**

To a solution of **S26** (8.0 mg, 0.0247 mmol,1.0 equiv) in EtOAc (0.3 mL) and AcOH (0.3 mL) was added PtO_2 (22.0 mg, 0.0987 mmol, 4.0 equiv) at 33 °C. The reaction mixture was degassed with H_2 for three times and equipped with a H_2 balloon. After stirring at same temperature for 1 h, the resulting mixture was filtered through a pad of celite and washed with EtOAc. The filtrate was concentrated under reduced pressure to give the crude product. To a solution of above crude product in DMSO (0.5 mL) was added 2-iodoxybenzoic acid (IBX, 14.0 mg, 0.0494 mmol, 2.0 equiv) at 33 °C. After stirring at same temperature for 45 min, the reaction was quenched with a mixture of saturated aqueous $NaHCO_3$ solution and saturated aqueous $Na_2S_2O_3$ solution. The reaction mixture was extracted with EtOAc and the combined organic layers were washed with brine, dried over Na_2SO_4, filtered and concentrated under reduced pressure. The residue was purified by PTLC (petroleum ether:EtOAc = 4:1) to afford **24** (6.6 mg, 82% yield) as a colorless oil.

TLC: R_f = 0.4 (petroleum ether:EtOAc = 5:1).

$[\alpha]_D^{25}$ = +12 (c = 0.2, $CHCl_3$).

^{1}H NMR (500 MHz, $CDCl_3$) δ 6.77 (d, *J* = 8.8 Hz, 1H), 6.68 (dd, *J* = 8.8, 2.9 Hz, 1H), 6.43 (d, *J* = 2.9 Hz, 1H), 3.75 (s, 3H), 3.13 (d, *J* = 16.4 Hz, 1H), 2.60 – 2.44 (m, 2H), 2.31 (d, *J* = 16.4 Hz, 1H), 2.28 – 2.24 (m, 1H), 2.16 – 2.08 (m, 1H), 2.03 (ddd, *J* = 14.7, 10.0, 4.9 Hz, 1H), 1.86 (dt, *J* = 14.3, 5.7 Hz, 1H), 1.78 – 1.71 (m, 1H), 1.66 – 1.59 (m, 2H), 1.34 (s, 3H), 1.16 (s, 3H), 0.76 (s, 3H).

^{13}C NMR (125 MHz, $CDCl_3$) δ 212.8, 154.0, 146.5, 130.7, 119.2, 113.1, 112.9, 85.9, 55.8, 49.9, 48.8, 46.3, 43.3, 40.3, 39.0, 36.5, 28.8, 26.2, 24.1, 23.5, 22.6.

HRMS(ESI)$^+$ calcd for $C_{21}H_{27}O_3^+$ [M+H]$^+$: 327.1955; found: 327.1958.

FT-IR (cm^{-1}): 2923, 2856, 1711, 1492, 1223, 1135, 1041, 961, 767.

Preparation of Compound S27

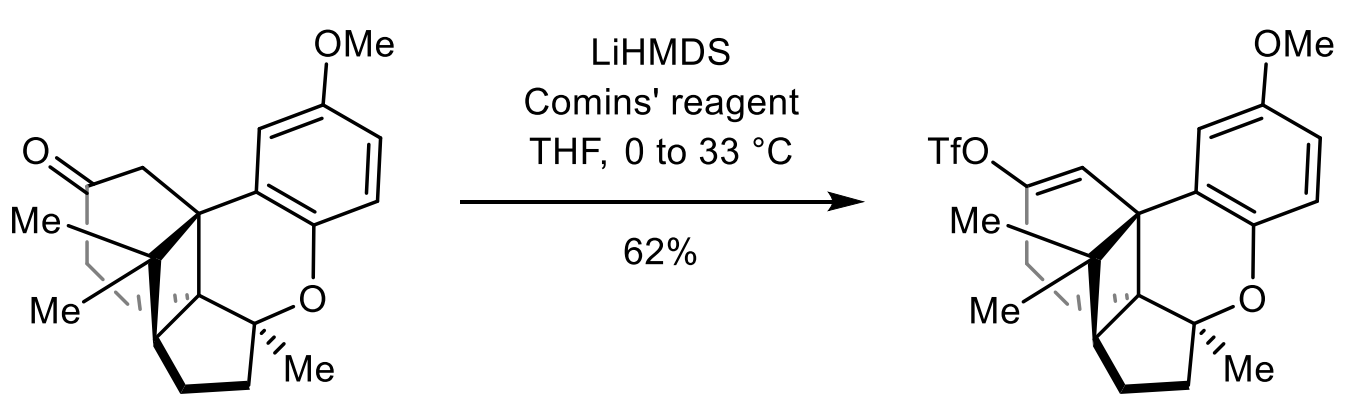

24 **S27**

To a solution of **24** (9.0 mg, 0.0276 mmol, 1.0 equiv) in THF (0.4 mL) was added LiHMDS (1.0 M in THF, 36.0 μL, 0.0359 mmol, 1.3 equiv) at 0 °C. The resulting solution was stirred at 0 °C for 10 min. A solution of Comins' reagent (54.0 mg, 0.138 mmol, 5.0 equiv) in THF (0.2 mL) was added to the reaction mixture at 0 °C. After stirring at 33 °C for 2 h, the reaction was quenched by saturated aqueous NH_4Cl solution. The reaction mixture was extracted with EtOAc and the combined organic layers were washed with brine, dried over Na_2SO_4, filtered and concentrated under reduced pressure. The residue was purified by silica gel column chromatography (petroleum ether:DCM = 3:1→petroleum ether:EtOAc = 8:1) to afford **S27** (7.8 mg, 62% yield) as a colorless oil.

TLC: R_f = 0.6 (petroleum ether:DCM = 3:1).
$[\alpha]_D^{21}$ = +132 (c = 0.1, $CHCl_3$)
^{1}H NMR (500 MHz, $CDCl_3$) δ 6.80 (d, *J* = 8.7 Hz, 1H), 6.71 (dd, *J* = 8.8, 2.9 Hz, 1H), 6.54 (d, *J* = 2.9 Hz, 1H), 6.00 (d, *J* = 2.2 Hz, 1H), 3.78 (s, 3H), 2.57 – 2.42 (m, 2H), 2.17 – 2.08 (m, 1H), 2.04 – 1.99 (m, 1H), 1.80 – 1.68 (m, 2H), 1.66 – 1.59 (m, 3H), 1.27 (s, 3H), 1.08 (s, 3H), 0.80 (s, 3H).
^{13}C NMR (125 MHz, $CDCl_3$) δ 154.2, 149.4, 147.1, 128.9, 124.1, 119.5, 118.7 (q, *J* = 318.4 Hz), 113.0, 112.7, 86.7, 55.9, 49.5, 46.3, 45.0, 44.9, 38.7, 29.5, 27.9, 26.3, 24.1, 23.3, 21.5.
^{19}F NMR (471 MHz, $CDCl_3$) δ –73.94.
HRMS(ESI)$^+$ calcd for $C_{22}H_{26}O_5F_3S^+$ [M+H]$^+$: 459.1448; found: 459.1442.
FT-IR (cm^{-1}): 2951, 1493, 1419, 1211, 1141, 891, 851, 741.

Preparation of Compound 25

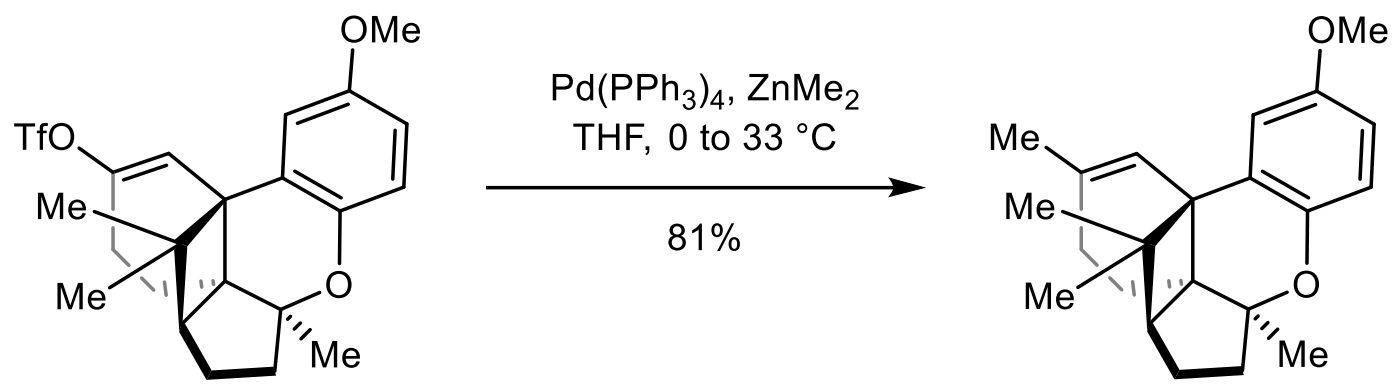

To a solution of **S27** (6.3 mg, 0.0138 mmol, 1.0 equiv) in THF (0.2 mL) was added $Pd(PPh_3)_4$ (3.2 mg, 0.00275 mmol, 0.2 equiv) at 0 °C, and stirred at this temperature for 5 min, $ZnMe_2$ (1 M in hexane, 0.28 mL, 0.275 mmol, 20.0 equiv) was added. After stirring at 33 °C for 2 h, the reaction was quenched by careful addition of pH 7 buffer at 0 °C. The reaction mixture was extracted with EtOAc and the combined organic layers were washed with brine, dried over Na_2SO_4, filtered and concentrated under reduced pressure. The residue was purified by PTLC (petroleum ether:EtOAc = 30:1) to afford **25** (3.6 mg, 81% yield) as a colorless oil.

TLC: R_f = 0.8 (petroleum ether:EtOAc = 30:1).
$[\alpha]_D^{21}$ = +112 (c = 0.1, $CHCl_3$).
^{1}H NMR (500 MHz, $CDCl_3$) δ 6.75 (d, *J* = 8.3 Hz, 1H), 6.68 – 6.62 (m, 2H), 5.57 (s, 1H), 3.77 (s, 3H), 2.14 – 1.98 (m, 3H), 1.95 – 1.90 (m, 1H), 1.78 (s, 3H), 1.74 – 1.67 (m, 1H), 1.62 – 1.53 (m, overlap with H_2O, 2H), 1.45 (dd, *J* = 9.8, 5.2 Hz, 2H), 1.27 (s, 3H), 0.94 (s, 3H), 0.78 (s, 3H).
^{13}C NMR (125 MHz, $CDCl_3$) δ 153.8, 147.2, 135.4, 131.5, 127.6, 118.8, 113.6, 111.7, 86.8, 55.8, 49.2, 45.9, 43.8, 43.7, 38.6, 29.3, 28.1, 26.9, 24.3, 24.2, 23.6, 21.8.
HRMS(ESI)$^+$ calcd for $C_{22}H_{28}NaO_2^+$ [M+Na]$^+$: 347.1982; found: 347.1985.
FT-IR (cm^{-1}): 3477, 2921, 2852, 1491, 1226, 1140, 1045, 795.

Preparation of Compound S28

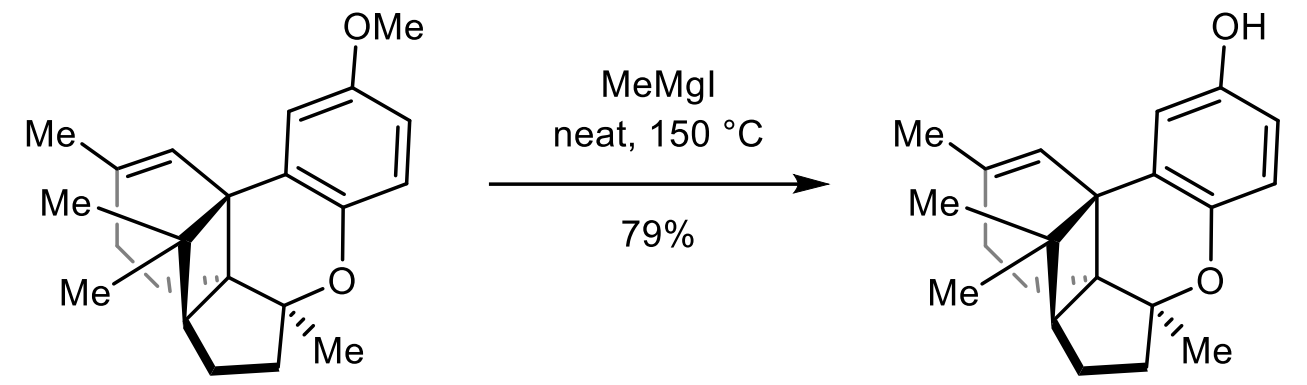

25 **S28**

To a solution of **25** (3.7 mg, 0.0114 mmol,1.0 equiv) in Et_2O (0.2 mL) was added MeMgI (3.0 M in Et_2O, 0.23 mL, 0.684 mmol, 60.0 equiv) at 0 °C. After gently removing Et_2O under vacuum, the resulting residue was heated to 150 °C under vacuum for 1 h. After cooling to 33 °C and then back to 0 °C, the residue was diluted in Et_2O. Then, the reaction mixture was carefully quenched with saturated aqueous NH_4Cl solution. The reaction mixture was extracted with EtOAc and the combined organic layers were washed with brine, dried over $MgSO_4$, filtered and concentrated under reduced pressure. The residue was purified by PTLC (petroleum ether:EtOAc = 9:1) to afford **S28** (2.8 mg, 79% yield) as a yellow oil.

TLC: R_f = 0.3 (petroleum ether:EtOAc = 9:1).

$[\alpha]_D^{22}$ = +50 (c = 0.1, $CHCl_3$).

^{1}H NMR (500 MHz, $CDCl_3$) δ 6.70 (d, *J* = 8.5 Hz, 1H), 6.60 (d, *J* = 2.9 Hz, 1H), 6.56 (dd, *J* = 8.5, 3.0 Hz, 1H), 5.56 (s, 1H), 4.37 (brs, 1H), 2.15 – 1.97 (m, 3H), 1.93 (dd, *J* = 7.8, 2.9 Hz, 1H), 1.79 (s, 3H), 1.74 – 1.67 (m, 1H), 1.62 – 1.54 (m, overlap with H_2O, 2H), 1.47 – 1.41 (m, 2H), 1.26 (s, 3H), 0.93 (s, 3H), 0.77 (s, 3H).

^{13}C NMR (125 MHz, $CDCl_3$) δ 149.5, 147.3, 135.5, 131.8, 127.5, 119.2, 114.4, 113.8, 86.9, 49.3, 46.1, 43.9, 43.7, 38.8, 29.4, 28.1, 27.0, 24.4, 24.3, 23.6, 21.8.

HRMS$(ESI)^+$ calcd for $C_{21}H_{27}O_2^+$ $[M+H]^+$: 311.2006; found: 311.2010.

FT-IR (cm^{-1}): 3386, 2921, 2851, 1491, 1448, 1206, 1081, 815, 749.

Preparation of (+)-cochlearol B (4)

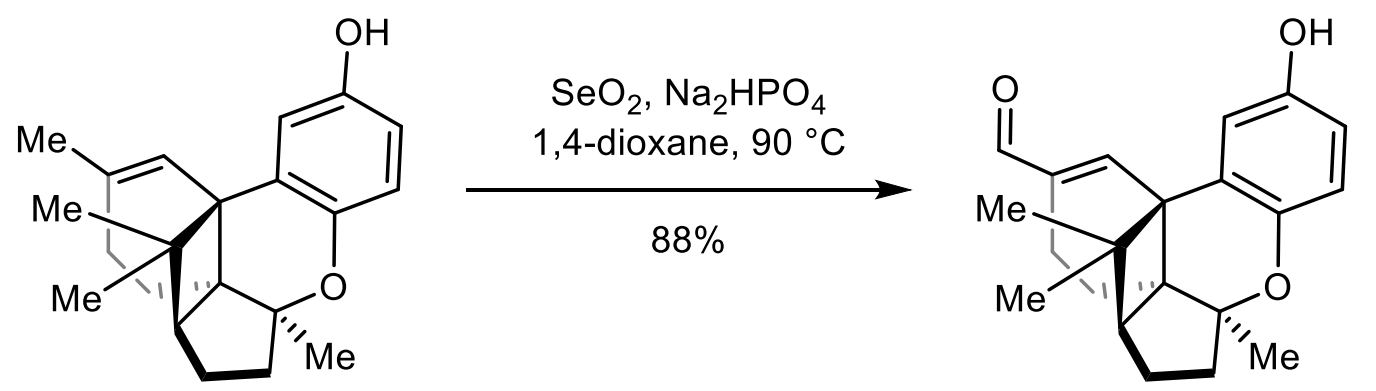

S28 (+)-cochlearol B (**4**)

To a solution of **S28** (2.7 mg, 0.00870 mmol, 1.0 equiv) in 1,4-dioxane (0.2 mL) were added SeO_2 (1.3 mg, 0.0113 mmol, 1.3 equiv) and Na_2HPO_4 (6.2 mg, 0.0435 mmol, 5.0 equiv) at 33 °C. After stirring at 90 °C for 30 min, another portion of SeO_2 (1.3 mg, 0.0113 mmol, 1.3 equiv) and Na_2HPO_4 (6.2 mg, 0.0435 mmol, 5.0 equiv) was added at 33 °C. After stirring at 90 °C for 30 min, the reaction mixture was cooled to 33 °C and filtered through a pad of celite. The filtrate was concentrated under reduced pressure. The residue was purified by PTLC (petroleum ether:EtOAc = 2:1) to afford (+)-cochlearol B (**4**) (2.5 mg, 88% yield) as a colorless oil.

TLC: R_f = 0.4 (petroleum ether:EtOAc = 2:1).

$[\alpha]_D^{25}$ = +144 (c = 0.1, MeOH).

^{1}H NMR (500 MHz, $(CD_3)_2CO$) δ 9.55 (s, 1H), 7.87 (s, 1H), 7.07 (d, *J* = 2.3 Hz, 1H), 6.80 (d, *J* = 1.9 Hz, 1H), 6.70 – 6.63 (m, 2H), 2.64 (ddd, *J* = 16.6, 4.7, 3.6 Hz, 1H), 2.20 – 2.11 (m, 1H), 2.10 – 2.07 (m, overlap, 1H), 2.04 – 1.99 (m, 1H), 1.78 – 1.72 (m, 1H), 1.71 – 1.60 (m, 3H), 1.38 (ddd, *J* = 13.4, 11.5, 4.7 Hz, 1H), 1.29 (s, 3H), 1.01 (s, 3H), 0.89 (s, 3H).

^{13}C NMR (125 MHz, $(CD_3)_2CO$) δ 193.4, 156.4, 152.4, 147.3, 142.1, 129.1, 119.9, 115.4, 115.1, 87.2, 50.3, 48.2, 46.1, 45.1, 39.0, 29.1, 26.8, 24.6, 23.8, 21.8, 19.5.

HRMS$(ESI)^+$ calcd for $C_{21}H_{25}O_3^+$ $[M+H]^+$: 325.1798; found: 325.1800.

FT-IR (cm^{-1}): 3414, 2923, 2851, 1676, 1631, 1492, 1449, 1209, 1134, 789, 751.

4. X-ray Crystallographic Data

X-ray crystallographic data for compound 18 (CCDC: 2492584)

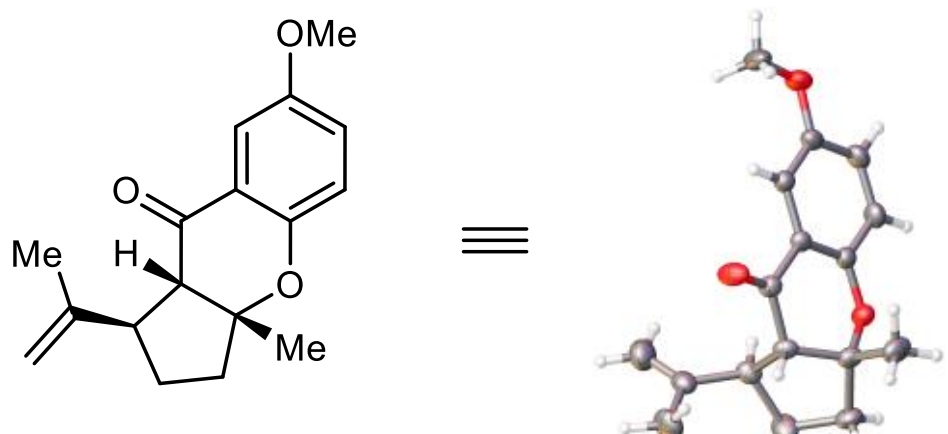

18

The crude product **18** was purified by silica gel column chromatography (petroleum ether/EtOAc). Concentration of the combined fractions containing **18** under reduced pressure using a rotary evaporator directly afforded colorless single crystals. The crystal structure of compound **18** was solved using OLEX2.

Supplementary Table 9. Crystal data and structure refinement for 18

Empirical formula	$C_{17}H_{20}O_3$
Formula weight	272.33
Temperature/K	169.99(10)
Crystal system	orthorhombic
Space group	$P2_12_12_1$
a/Å	7.78061(12)
b/Å	10.93062(16)
c/Å	17.3290(2)
α/°	90
β/°	90
γ/°	90
Volume/Å^3	1473.78(4)
Z	4
ρ_{calc}g/cm^3	1.227
μ/mm^{-1}	0.666
F(000)	584.0
Crystal size/mm^3	0.16 × 0.12 × 0.1
Radiation	Cu Kα (λ = 1.54184)
2Θ range for data collection/°	9.566 to 147.796
Index ranges	-8 ≤ h ≤ 9, -13 ≤ k ≤ 13, -21 ≤ l ≤ 21
Reflections collected	15707
Independent reflections	2959 [R_{int} = 0.0461, R_{sigma} = 0.0254]
Data/restraints/parameters	2959/0/193
Goodness-of-fit on F^2	1.038
Final R indexes [I>=2σ (I)]	R_1 = 0.0350, wR_2 = 0.0907
Final R indexes [all data]	R_1 = 0.0359, wR_2 = 0.0918
Largest diff. peak/hole / e Å^{-3}	0.15/-0.13
Flack/Hooft parameter	-0.09(8)/-0.09(8)

X-ray crystallographic data for compound 19 (CCDC: 2492585)

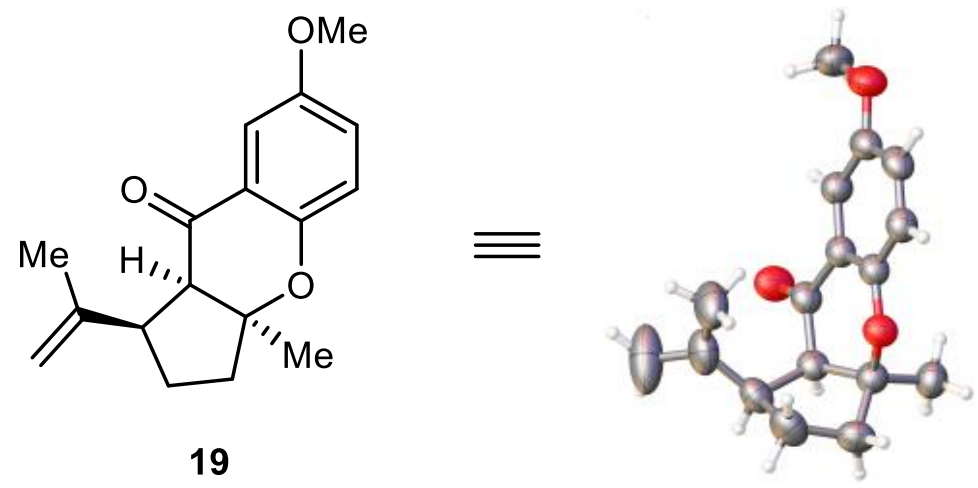

19

The crude product **19** was purified by silica gel column chromatography (petroleum ether/EtOAc). Concentration of the combined fractions containing **19** under reduced pressure using a rotary evaporator directly afforded colorless single crystals. The crystal structure of compound **19** was solved using OLEX2.

Supplementary Table 10. Crystal data and structure refinement for 19

Empirical formula	$C_{17}H_{20}O_3$
Formula weight	272.33
Temperature/K	170.00
Crystal system	monoclinic
Space group	$P2_1$
a/Å	7.6156(2)
b/Å	7.6565(2)
c/Å	12.4216(3)
α/°	90
β/°	90.1590(10)
γ/°	90
Volume/Å^3	724.29(3)
Z	2
ρ_{calc}g/cm^3	1.249
μ/mm^{-1}	0.677
F(000)	292.0
Crystal size/mm^3	0.15 × 0.12 × 0.09
Radiation	CuKα (λ = 1.54178)
2Θ range for data collection/°	7.116 to 136.404
Index ranges	-9 ≤ h ≤ 9, -9 ≤ k ≤ 9, -14 ≤ l ≤ 14
Reflections collected	21546
Independent reflections	2643 [R_{int} = 0.0448, R_{sigma} = 0.0308]
Data/restraints/parameters	2643/1/185
Goodness-of-fit on F^2	1.208
Final R indexes [I>=2σ (I)]	R_1 = 0.0433, wR_2 = 0.1248
Final R indexes [all data]	R_1 = 0.0436, wR_2 = 0.1249
Largest diff. peak/hole / e Å^{-3}	0.21/-0.18
Flack/Hooft parameter	0.09(4)/0.07(5)

X-ray crystallographic data for compound 10 (CCDC: 2492583)

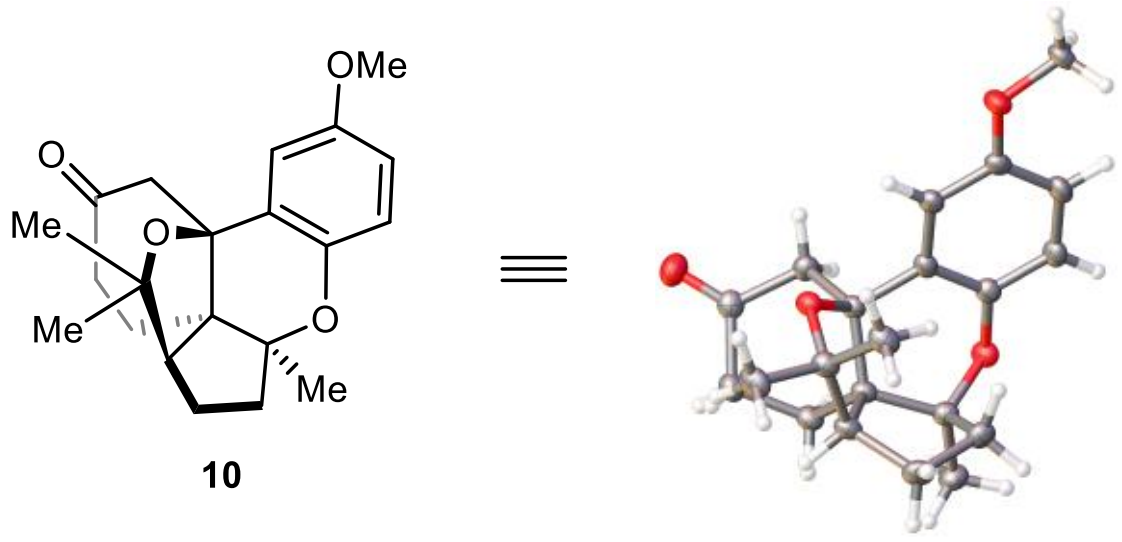

Anti-solvent vapor-assisted crystallization[14]: At room temperature, compound **10** was dissolved in ethyl ether. Single crystals of **10** were grown via slow diffusion of anti-solvent *n*-heptane vapor into the solution. The crystal structure of compound **10** was solved using OLEX2.

Supplementary Table 11. Crystal data and structure refinement for 10

Empirical formula	$C_{21}H_{26}O_4$
Formula weight	342.42
Temperature/K	150.00(10)
Crystal system	orthorhombic
Space group	$P2_12_12_1$
a/Å	8.3952(3)
b/Å	13.2868(4)
c/Å	15.9184(4)
α/°	90
β/°	90
γ/°	90
Volume/Å^3	1775.62(9)
Z	4
ρ_{calc}g/cm^3	1.281
μ/mm^{-1}	0.703
F(000)	736.0
Crystal size/mm^3	0.16 × 0.12 × 0.1
Radiation	Cu Kα (λ = 1.54184)
2Θ range for data collection/°	8.668 to 147.998
Index ranges	-8 ≤ h ≤ 10, -16 ≤ k ≤ 14, -18 ≤ l ≤ 19
Reflections collected	6693
Independent reflections	3500 [R_{int} = 0.0396, R_{sigma} = 0.0486]
Data/restraints/parameters	3500/0/230
Goodness-of-fit on F^2	1.022
Final R indexes [I>=2σ (I)]	R_1 = 0.0481, wR_2 = 0.1267
Final R indexes [all data]	R_1 = 0.0506, wR_2 = 0.1308
Largest diff. peak/hole / e Å^{-3}	0.29/-0.28
Flack parameter	0.32(15)

X-ray crystallographic data for compound 21 (CCDC: 2492586)

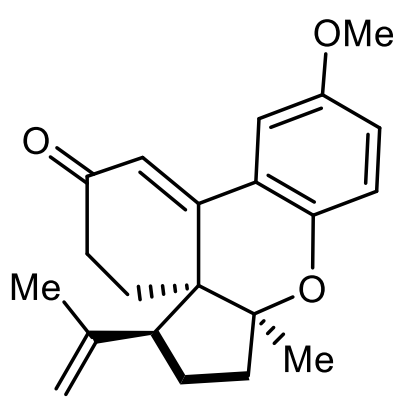

21

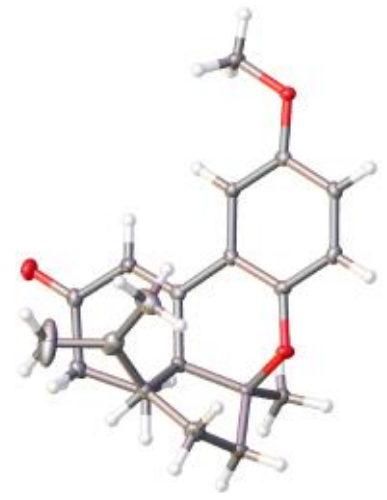

The crude product **21** was purified by silica gel column chromatography (petroleum ether/EtOAc). Concentration of the combined fractions containing **21** under reduced pressure using a rotary evaporator directly afforded colorless single crystals. The crystal structure of compound **21** was solved using OLEX2.

Supplementary Table 12. Crystal data and structure refinement for 21

Empirical formula	$C_{21}H_{24}O_3$
Formula weight	324.40
Temperature/K	100.00
Crystal system	monoclinic
Space group	$P2_1$
a/Å	7.3731(3)
b/Å	7.9107(4)
c/Å	14.7748(7)
α/°	90
β/°	101.009(2)
γ/°	90
Volume/Å^3	845.90(7)
Z	2
ρ_{calc}g/cm^3	1.274
μ/mm^{-1}	0.666
F(000)	348.0
Crystal size/mm^3	0.15 × 0.12 × 0.1
Radiation	CuKα (λ = 1.54178)
2Θ range for data collection/°	6.094 to 136.45
Index ranges	-8 ≤ h ≤ 8, -9 ≤ k ≤ 9, -17 ≤ l ≤ 17
Reflections collected	32484
Independent reflections	3069 [R_{int} = 0.0404, R_{sigma} = 0.0229]
Data/restraints/parameters	3069/1/220
Goodness-of-fit on F^2	1.110
Final R indexes [I>=2σ (I)]	R_1 = 0.0331, wR_2 = 0.0865
Final R indexes [all data]	R_1 = 0.0331, wR_2 = 0.0866
Largest diff. peak/hole / e Å^{-3}	0.22/-0.25
Flack/Hooft parameter	0.08(3)/0.10(3)

X-ray crystallographic data for compound 27 (CCDC: 2492587)

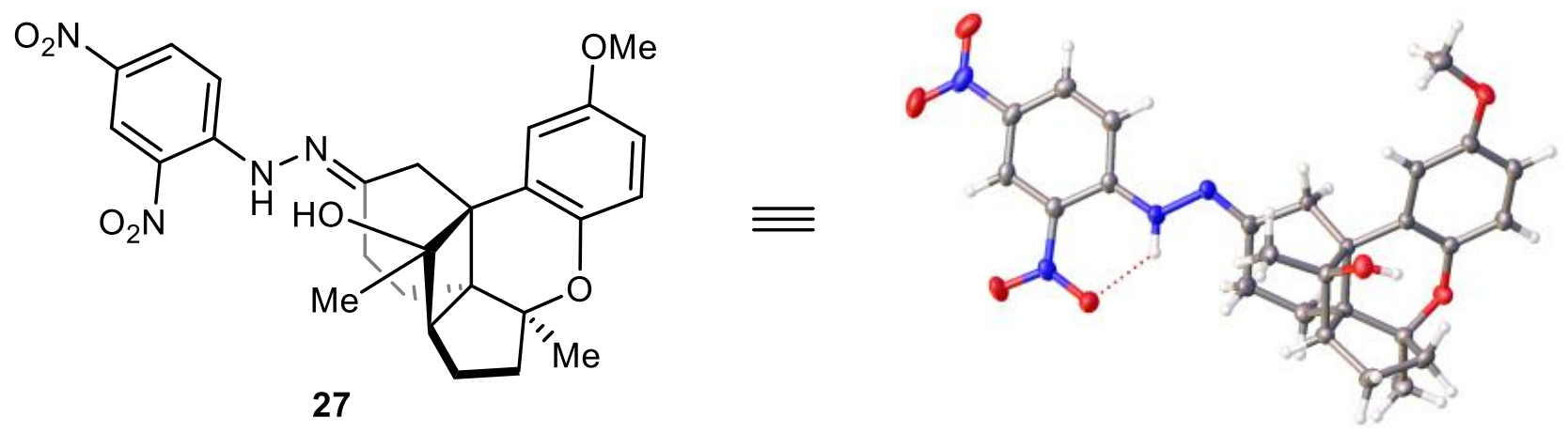

At room temperature, compound **27** was dissolved in a mixture of dichloromethane and petroleum ether. The solution was then allowed to evaporate slowly, yielding colorless crystals. The crystal structure of compound **27** was solved using OLEX2.

Supplementary Table 13. Crystal data and structure refinement for 27

Empirical formula	$C_{26}H_{28}N_4O_7$
Formula weight	508.52
Temperature/K	150.00
Crystal system	monoclinic
Space group	$P2_1$
a/Å	7.2940(5)
b/Å	15.9994(12)
c/Å	20.4598(15)
α/°	90
β/°	92.493(2)
γ/°	90
Volume/Å^3	2385.4(3)
Z	4
ρ_{calc}g/cm^3	1.416
μ/mm^{-1}	0.868
F(000)	1072.0
Crystal size/mm^3	0.15 × 0.13 × 0.11
Radiation	CuKα (λ = 1.54178)
2Θ range for data collection/°	4.322 to 136.42
Index ranges	-8 ≤ h ≤ 8, -19 ≤ k ≤ 19, -24 ≤ l ≤ 24
Reflections collected	77313
Independent reflections	8649 [R_{int} = 0.0457, R_{sigma} = 0.0333]
Data/restraints/parameters	8649/1/676
Goodness-of-fit on F^2	1.054
Final R indexes [I>=2σ (I)]	R_1 = 0.0329, wR_2 = 0.0836
Final R indexes [all data]	R_1 = 0.0330, wR_2 = 0.0837
Largest diff. peak/hole / e Å^{-3}	0.33/-0.25
Flack/Hooft parameter	0.08(2)/0.09(2)

5. Comparison of the Spectra and Data of (–)-Ganocins A–C

^{1}H NMR spectra and data comparison of our synthetic, Natural[15], Zhao's synthetic[2], Li and Tang's synthetic[5], and Sugital's synthetic[8] (–)-ganocin A

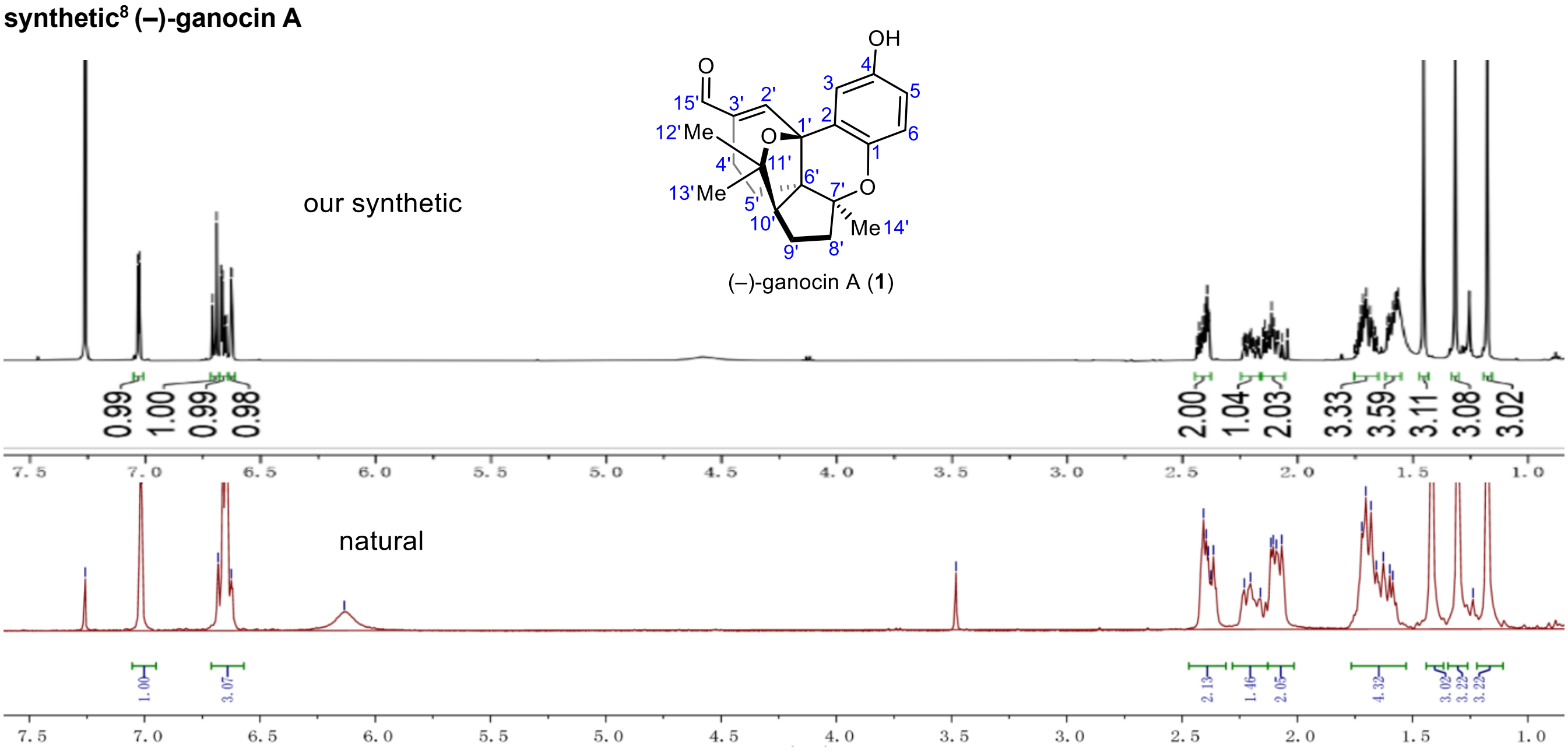

H	Our synthetic (500 MHz, $CDCl_3$)	Natural[15] (400 MHz, $CDCl_3$)	Zhao's synthetic[2] (600 MHz, $CDCl_3$)	Li and Tang's synthetic[5] (400 MHz, $CDCl_3$)	Sugital's synthetic[8] (600 MHz, $CDCl_3$)
1	-	-	-	-	-
2	-	-	-	-	-
3	7.03 (d, J = 2.8 Hz, 1H)	7.01 (d, J = 2.4 Hz, 1H)	7.03 (d, J = 2.8 Hz, 1H)	7.03 (d, J = 2.8 Hz, 1H)	7.03 (d, J =2.4 Hz, 1H)
4	-	-	-	-	4.71 (s, 1H) (Ar-OH)
5	6.66 (dd, J = 8.6, 2.9 Hz, 1H)	6.64 (m, 1H)	6.66 (dd, J = 8.6, 2.8 Hz, 1H)	6.66 (dd, J = 8.6, 2.8 Hz, 1H)	6.66 (dd, J = 7.8, 2.4 Hz, 1H)
6	6.70 (d, J = 8.5 Hz, 1H)	6.64 (m, 1H)	6.70 (d, J = 8.6 Hz, 1H)	6.70 (d, J = 8.5 Hz, 1H)	6.69 (d, J = 7.8 Hz, 1H)
1'	-	-	-	-	-
2'	6.64 – 6.61 (m, 1H)	6.62 (m, 1H)	6.63 (s, 1H)	6.63 (m, 1H)	6.63 (brs, 1H)
3'	-	-	-	-	-
4'	2.44 – 2.38 (m, 1H); 2.25 – 2.16 (m, 1H)	2.37 (m, 1H); 2.18 (m, 1H)	2.39 (m, 1H); 2.20 (m, 1H)	2.39 (m, 1H); 2.21 (m, 1H)	2.39 (m, 1H); 2.20 (m, 1H)
5'	2.16 – 2.06 (m, 1H); 1.76 – 1.64 (m, 1H)	2.07 (m, 1H); 1.66 (m, 1H)	2.11 (m, 1H); 1.67 (m, 1H)	2.12 (m, 1H); 1.67 (m, 1H)	2.11 (m, 1H); 1.69 (m, 1H)
6'	-	-	-	-	-
7'	-	-	-	-	-
8'	2.16 – 2.06 (m, 1H); 1.62 – 1.56 (m, overlap with H_2O, 1H)	2.07 (m, 1H); 1.57 (m, 1H)	2.07 (m, 1H); 1.57 (m, 1H)	2.09 (m, 1H); 1.58 (m, 1H)	2.11 (m, 1H); 1.58 (m, 1H)
9'	1.76 – 1.64 (m, 2H)	1.69 (m, 2H)	1.69 (m, 2H)	1.70 (m, 2H)	1.69 (m, 2H)
10'	2.44 – 2.38 (m, 1H)	2.39 (m, 1H)	2.41 (m, 1H)	2.42(m, 1H)	2.41 (m, 1H)
11'	-	-	-	-	-
12'	1.18 (s, 3H)	1.17 (s, 3H)	1.18 (s, 3H)	1.18 (s, 3H)	1.18 (s, 3H)
13'	1.32 (s, 3H)	1.30 (s, 3H)	1.32 (s, 3H)	1.32 (s, 3H)	1.32 (s, 3H)
14'	1.45 (s, 3H)	1.42 (s, 3H)	1.45 (s, 3H)	1.46 (s, 3H)	1.45 (s, 3H)
15'	9.44 (s, 1H)	9.40 (s, 1H)	9.44 (s, 1H)	9.44 (s, 1H)	9.44 (s, 1H)

^{13}C NMR spectra and data comparison of our synthetic, Natural[15], Zhao's synthetic[2], Li and Tang's synthetic[5], and Sugital's synthetic[8] (–)-ganocin A

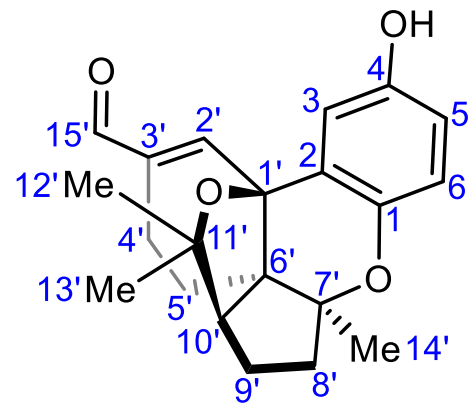

(–)-ganocin A (**1**)

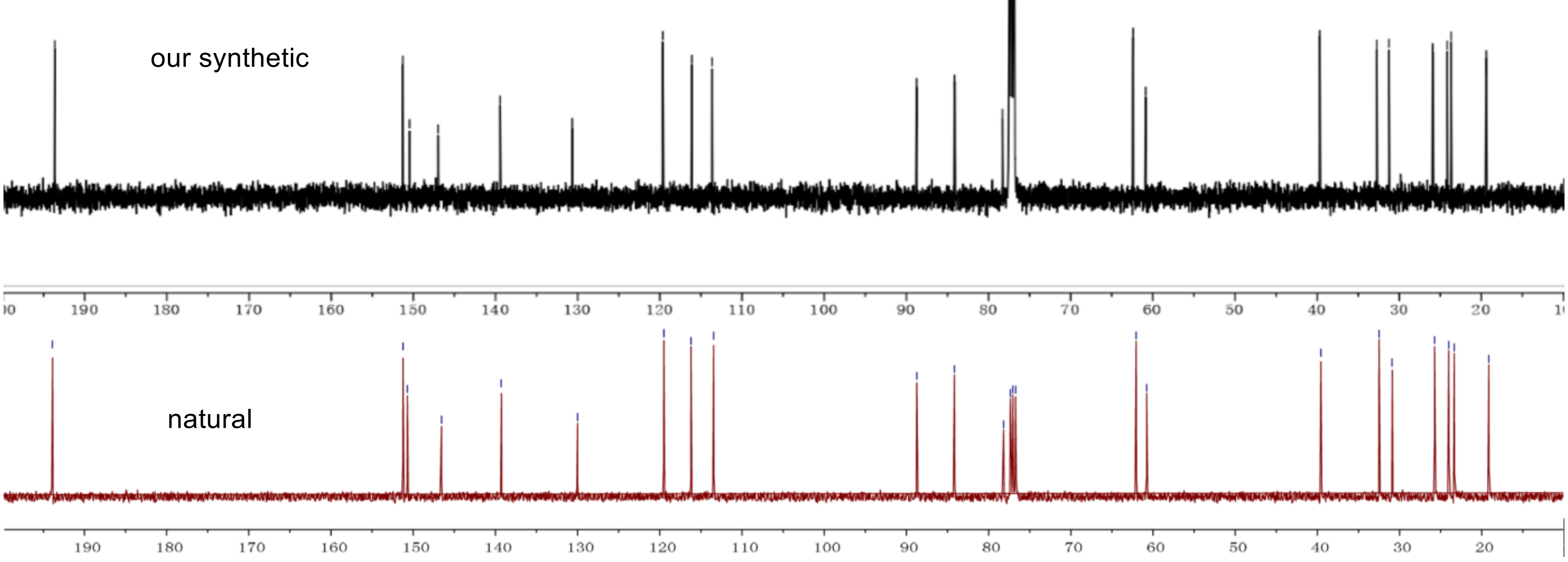

C	Our synthetic (125 MHz, $CDCl_3$)	Natural[15] (100 MHz, $CDCl_3$)	Zhao's synthetic[2] (150 MHz, $CDCl_3$)	Li and Tang's synthetic[5] (100 MHz, $CDCl_3$)	Sugital's synthetic[8] (150 MHz, $CDCl_3$)
1	146.8	146.5	146.8	146.7	146.7
2	130.5	129.9	130.5	130.3	130.2
3	113.5	113.4	113.5	113.5	113.4
4	150.3	151.2	151.2	151.3	151.1
5	116.0	116.1	116.0	116.2	116.0
6	119.5	119.5	119.5	119.6	119.5
1'	78.1	78.1	78.1	78.3	78.2
2'	151.1	150.6	150.3	150.7	150.5
3'	139.3	139.2	139.3	139.3	139.3
4'	19.3	19.1	19.3	19.3	19.2
5'	31.1	30.8	31.1	31.1	31.0
6'	60.7	60.7	60.7	60.8	60.7
7'	88.6	88.7	88.6	88.7	88.6
8'	39.5	39.5	39.5	39.6	39.5
9'	24.0	24.0	24.0	24.1	24.0
10'	62.2	62.0	62.2	62.2	62.1
11'	84.0	84.2	84.0	84.2	84.1
12'	25.8	25.8	25.8	25.9	25.7
13'	32.6	32.5	32.6	32.6	32.5
14'	23.5	23.3	23.5	23.5	23.4
15'	193.5	193.8	193.6	193.8	193.7

Note: *we revised ^{13}C assignments (C4, C2') from prior reports[2,5,8,15].*

^{1}H NMR spectra and data comparison of our synthetic and natural[15] (–)-ganocin B

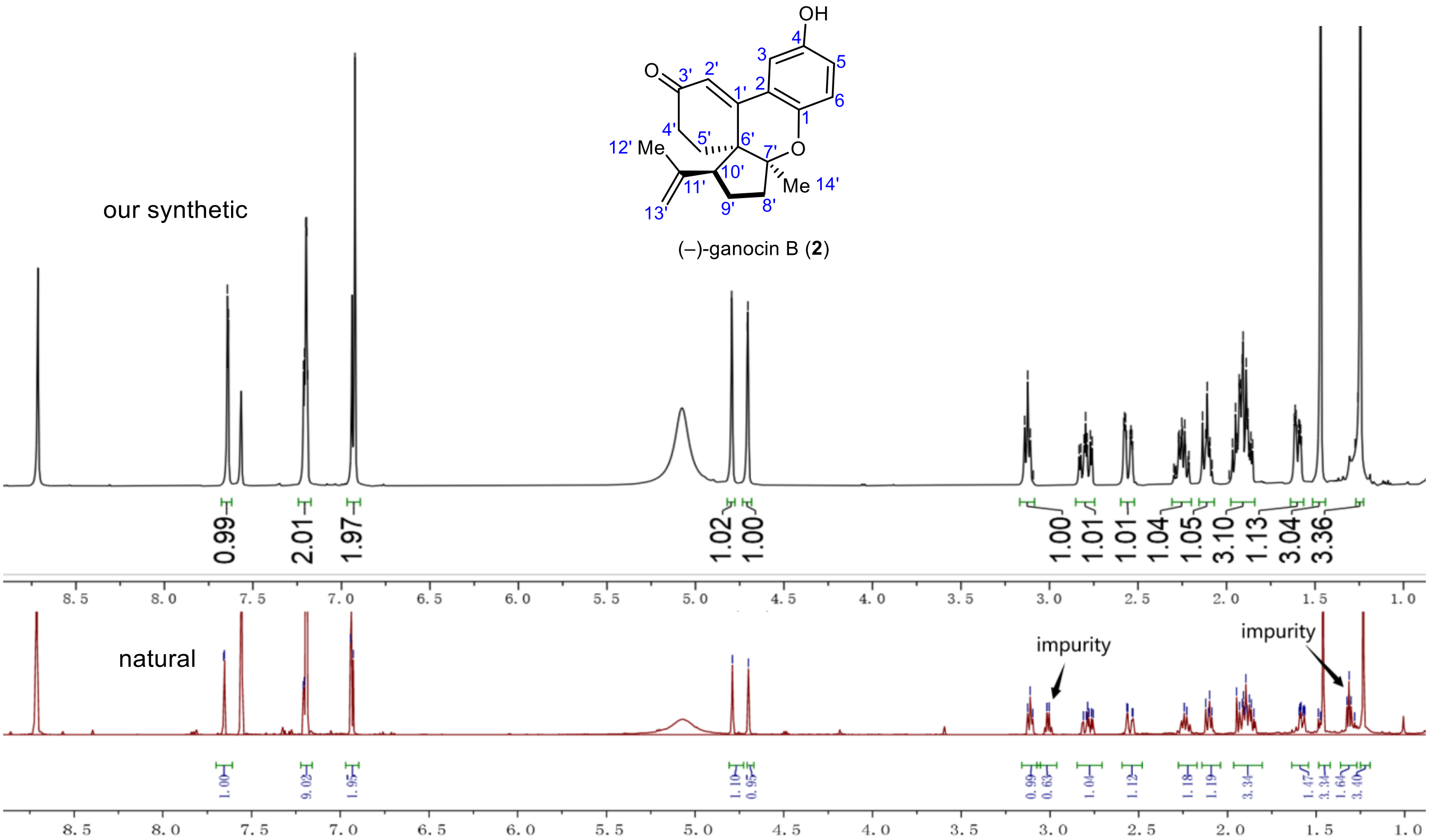

H	Our synthetic<br>(500 MHz, C_5D_5N)	Natural[15]<br>(600 MHz, C_5D_5N)	Δδ<br>(Our synthetic – Natural)
1	-	-	-
2	-	-	-
3	7.63 (d, *J* = 3.0 Hz, 1H)	7.65 (d, *J* = 3.0 Hz, 1H)	–0.02
4	-	-	-
5	7.20 (dd, *J* = 8.7, 2.8 Hz, 1H)	7.20 (dd, *J* = 9.0, 3.0 Hz, 1H)	0
6	6.92 (d, *J* = 8.7 Hz, 1H)	6.93, (d, *J* = 9.0 Hz, 1H)	–0.01
1'	-	-	-
2'	6.91 (s, 1H)	6.94 (s, 1H)	–0.03
3'	-	-	-
4'	2.79 (ddd, *J* = 18.5, 14.0, 4.9 Hz, 1H);<br>2.55 (ddd, *J* = 18.0, 4.9, 2.2 Hz, 1H)	2.78 (m, 1H);<br>2.55 (m, 1H)	+0.01;<br>0
5'	1.98 – 1.83 (m, 1H);<br>1.59 (ddd, *J* = 12.8, 4.9, 2.3 Hz, 1H)	1.86 (m, 1H);<br>1.57 (m, 1H)	-;<br>+0.02
6'	-	-	-
7'	-	-	-
8'	2.15 – 2.06 (m, 1H);<br>1.98 – 1.83 (m, 1H)	2.10 (m, 1H);<br>1.91 (m, 1H)	+0.01 (center);<br>-
9'	2.30 – 2.19 (m, 1H);<br>1.98 – 1.83 (m, 1H)	2.23 (m, 1H);<br>1.89 (m, 1H)	+0.02 (center);<br>-
10'	3.12 (t, *J* = 8.1 Hz, 1H)	3.11 (t, 1H)	+0.01
11'	-	-	-
12'	1.46 (s, 3H)	1.46 (s, 3H)	0
13'	4.79 (s, 1H);<br>4.72 – 4.68 (m, 1H)	4.79 (s, 1H);<br>4.70 (s, 1H)	0;<br>+0.01 (center)
14'	1.24 (s, 3H)	1.23 (s, 3H)	+0.01

^{13}C NMR spectra and data comparison of our synthetic and natural[15] (–)-ganocin B

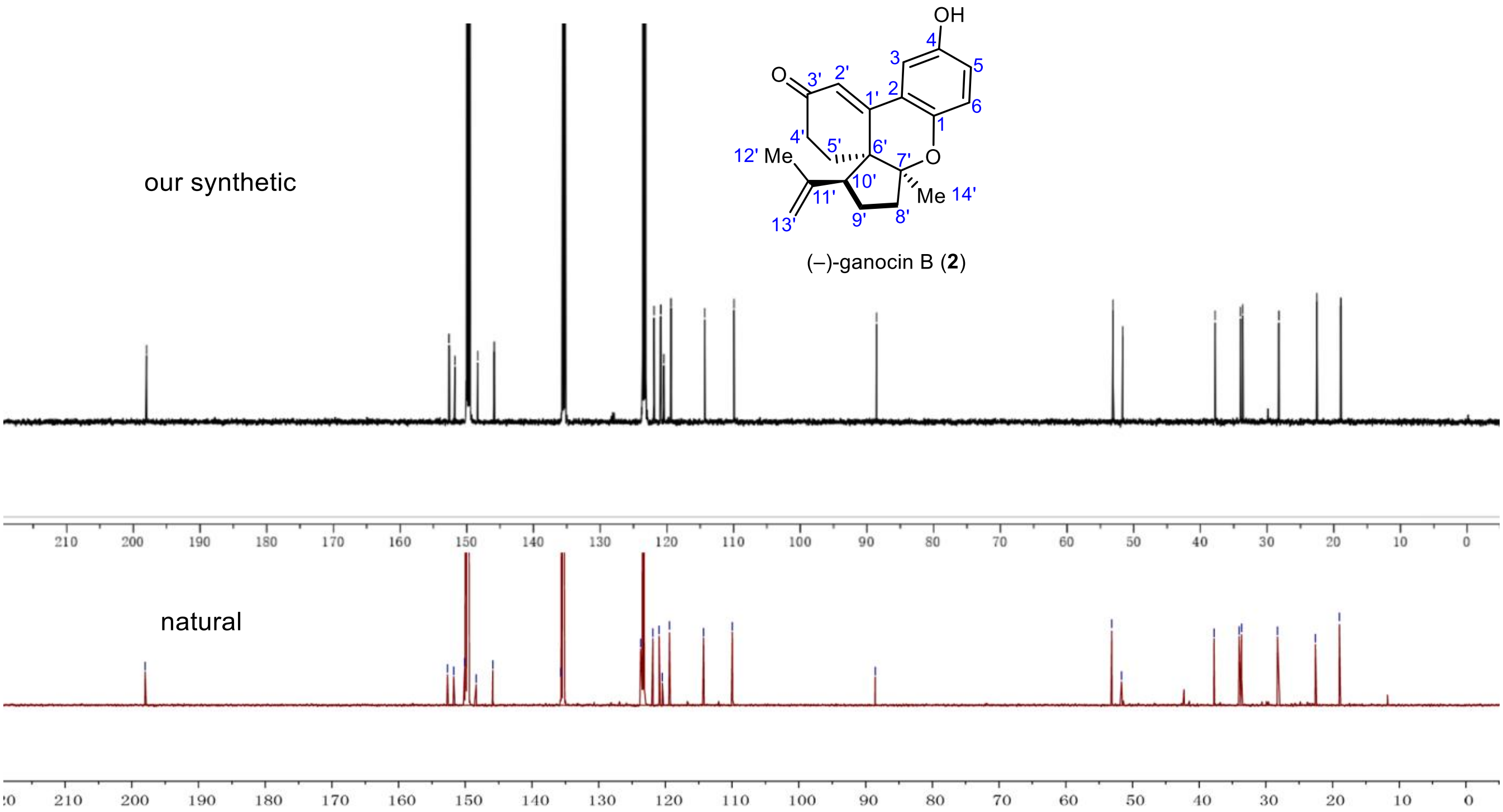

C	Our synthetic (125 MHz, C_5D_5N)	Natural[15] (150 MHz, C_5D_5N)	Δδ (Our synthetic – Natural)
1	148.4	148.4	0
2	120.5	120.4	+0.1
3	109.9	109.9	0
4	151.8	151.7	+0.1
5	121.9	121.9	0
6	119.4	119.4	0
1'	152.7	152.6	+0.1
2'	120.9	120.9	0
3'	198.0	198.0	0
4'	33.6	33.6	0
5'	34.0	33.9	+0.1
6'	51.7	51.6	+0.1
7'	88.6	88.5	+0.1
8'	37.8	37.7	+0.1
9'	28.2	28.2	0
10'	53.1	53.0	+0.1
11'	145.9	145.8	+0.1
12'	22.5	22.5	0
13'	114.3	114.3	0
14'	18.9	18.9	0

^{1}H NMR spectra and data comparison of our synthetic and natural[15] (–)-ganocin C

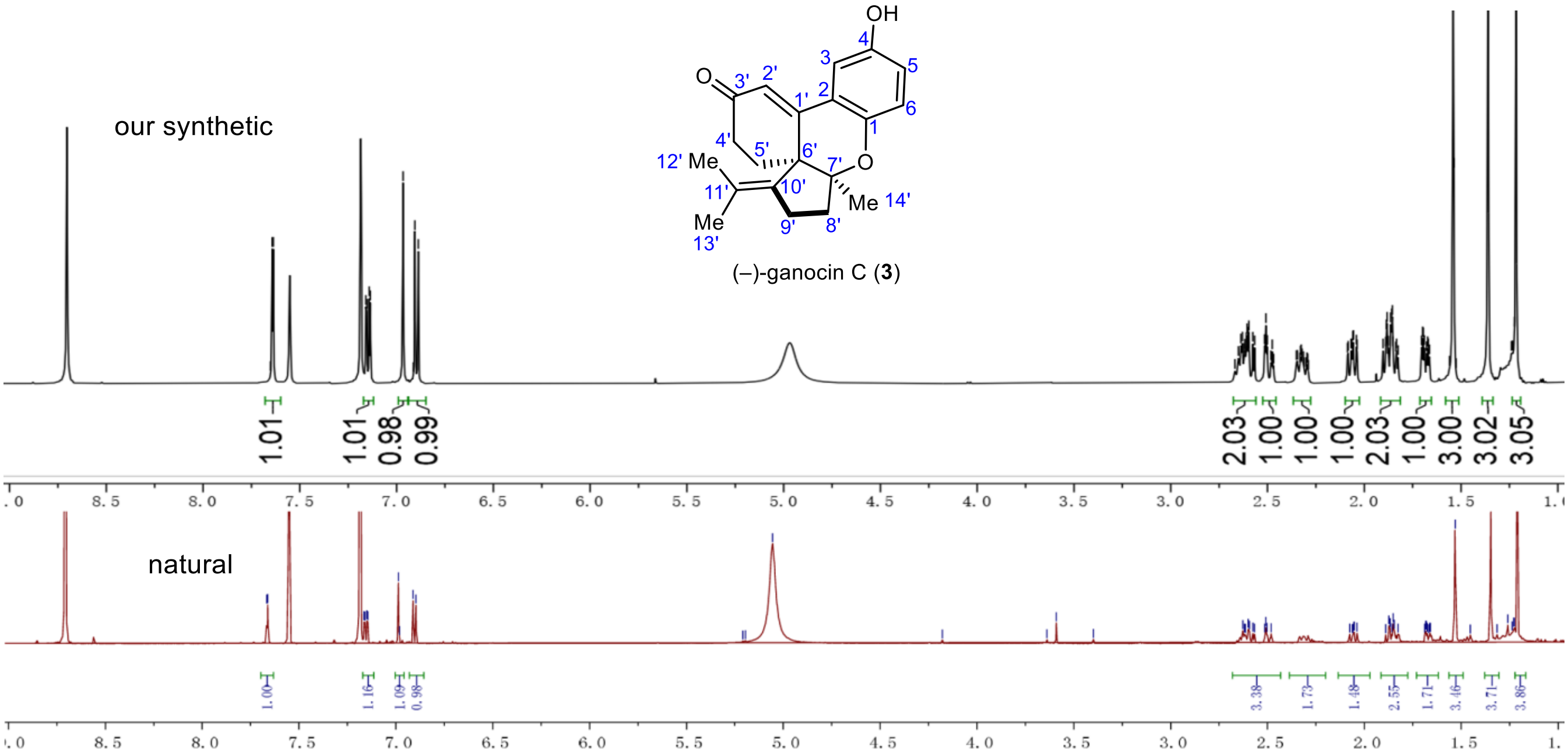

H	Our synthetic (500 MHz, C_5D_5N)	Natural[15] (600 MHz, C_5D_5N)	Δδ (Our synthetic – Natural)
1	-	-	-
2	-	-	-
3	7.64 (d, *J* = 2.9 Hz, 1H)	7.66 (d, *J* = 3.0 Hz, 1H)	–0.02
4	-	-	-
5	7.15 (dd, *J* = 8.8, 2.9 Hz, 1H)	7.16 (dd, *J* = 9.0, 3.0 Hz, 1H)	–0.01
6	6.90 (d, *J* = 8.8 Hz, 1H)	6.91 (d, *J* = 9.0 Hz, 1H)	–0.01
1'	-	-	-
2'	6.97 (s, 1H)	6.99 (s, 1H)	–0.02
3'	-	-	-
4'	2.68 – 2.56 (m, 1H);<br>2.49 (dt, *J* = 16.3, 3.5 Hz, 1H)	2.59 (m, 1H);<br>2.49 (m, 1H)	-;<br>0
5'	1.92 – 1.82 (m, 1H);<br>1.68 (ddd, *J* = 13.0, 4.7, 2.9 Hz, 1H)	1.84 (m, 1H);<br>1.66 (m, 1H)	-;<br>+0.02
6'	-	-	-
7'	-	-	-
8'	2.68 – 2.56 (m, 1H);<br>2.36 – 2.28 (m, 1H)	2.62 (m, 1H);<br>2.30 (m, 1H)	-;<br>-
9'	2.06 (ddd, *J* = 13.7, 9.3, 1.8 Hz, 1H);<br>1.92 – 1.82 (m, 1H)	2.05 (m, 1H);<br>1.85 (m, 1H)	+0.01;<br>-
10'	-	-	-
11'	-	-	-
12'	1.54 (s, 3H)	1.53 (s, 3H)	+0.01
13'	1.36 (s, 3H)	1.34 (s, 3H)	+0.02
14'	1.22 (s, 3H)	1.21 (s, 3H)	+0.01

^{13}C NMR spectra and data comparison of our synthetic and natural[15] (–)-ganocin C

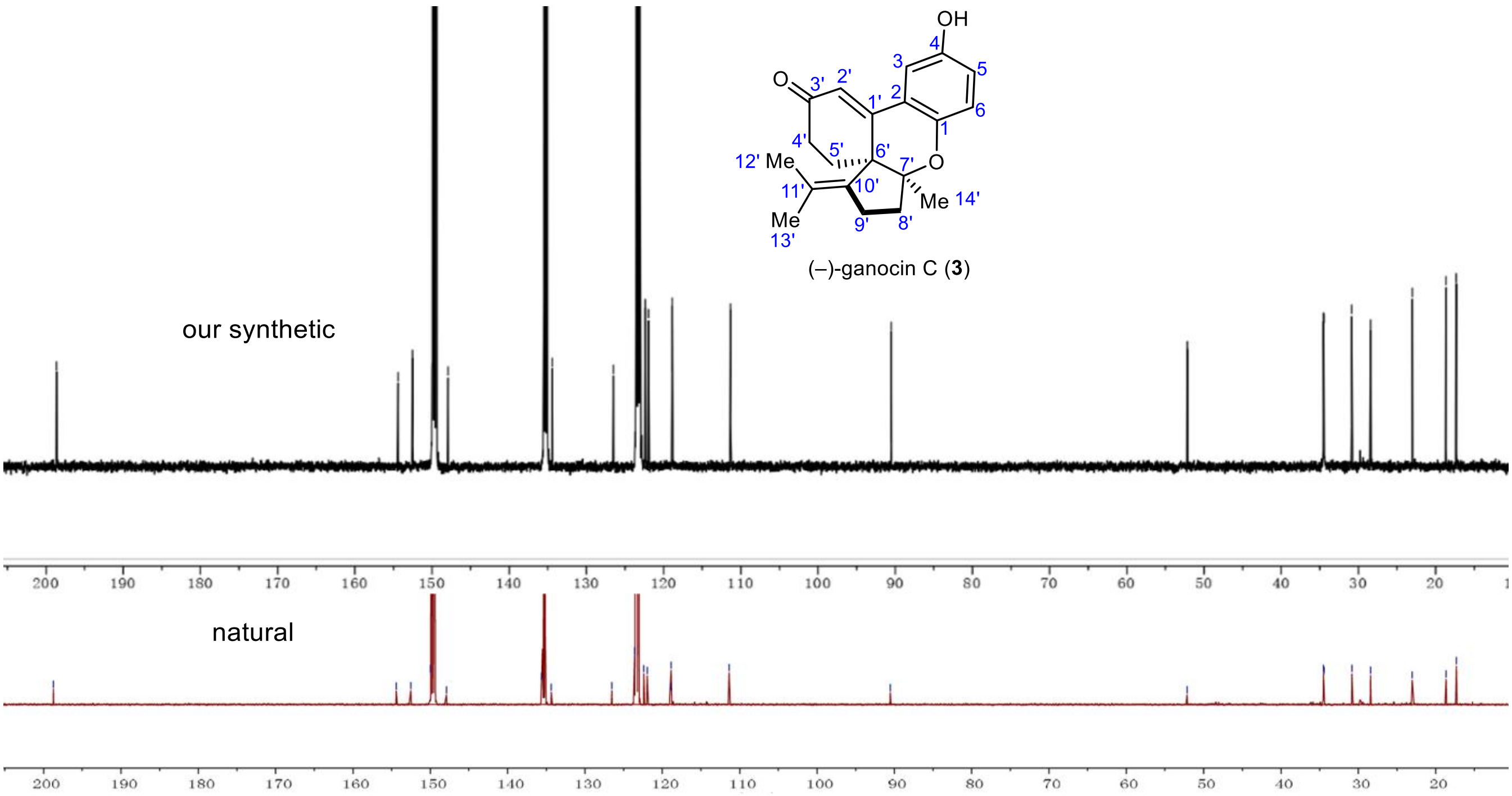

C	Our synthetic (125 MHz, C_5D_5N)	Natural[15] (150 MHz, C_5D_5N)	Δδ (Our synthetic – Natural)
1	147.9	147.9	0
2	118.9	118.9	0
3	111.3	111.4	–0.1
4	152.5	152.6	–0.1
5	122.0	122.1	–0.1
6	118.9	118.9	0
1'	154.4	154.5	–0.1
2'	122.4	122.5	–0.1
3'	198.6	198.8	–0.2
4'	34.52	34.6	–0.1
5'	30.9	30.9	0
6'	52.2	52.2	0
7'	90.5	90.6	–0.1
8'	28.4	28.5	–0.1
9'	34.46	34.5	0
10'	134.4	134.5	–0.1
11'	126.5	126.6	–0.1
12'	18.7	18.8	–0.1
13'	23.0	23.1	–0.1
14'	17.3	17.4	–0.1

6. Comparison of the Spectra and Data of (+)-Cochlearol B

1H NMR spectra and data comparison of our synthetic and natural[16] (+)-cochlearol B

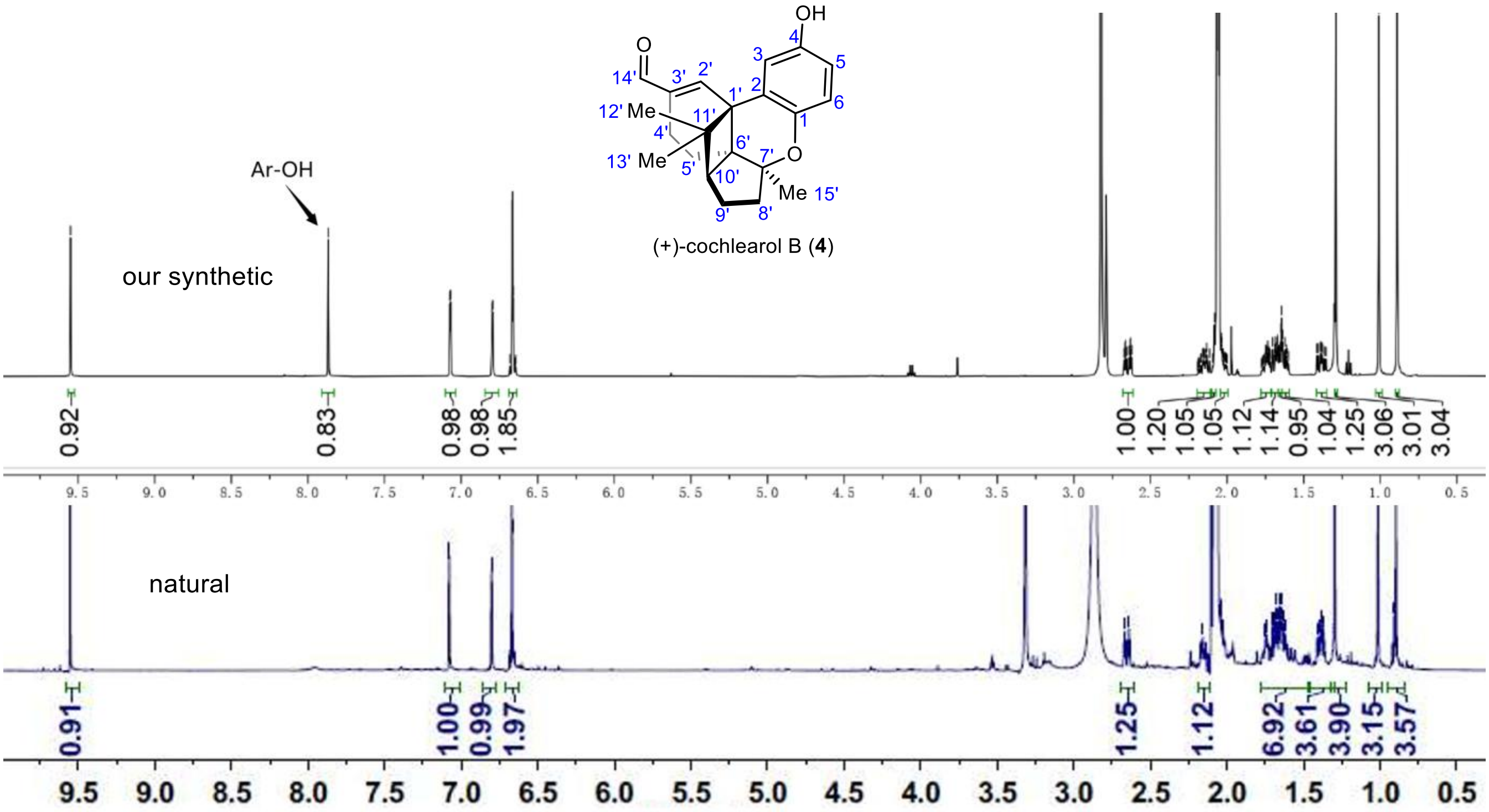

H	Our synthetic (500 MHz, $(CD_3)_2CO$)	Natural[16] (600 MHz, $(CD_3)_2CO$)	Δδ (Our synthetic – Natural)
1	-	-	-
2	-	-	-
3	6.80 (d, *J* = 1.9 Hz, 1H)	6.80 (d, *J* = 2.2 Hz, 1H)	0
Ar-OH	7.87 (s, 1H)	-	-
5	6.70 – 6.63 (m, 1H)	6.65 (dd, *J* = 8.5, 2.2 Hz, 1H)	-
6	6.70 – 6.63 (m, 1H)	6.68 (d, *J* = 8.5 Hz, 1H)	-
1'	-	-	-
2'	7.07 (d, *J* = 2.3 Hz, 1H)	7.08 (d, *J* = 2.0 Hz, 1H)	–0.01
3'	-	-	-
4'	2.64 (ddd, *J* = 16.6, 4.7, 3.6 Hz, 1H); 2.04 – 1.99 (m, 1H)	2.65 (m, 1H); 2.03 (m, 1H)	–0.01; –0.01 (center)
5'	1.71 – 1.60 (m, 1H); 1.38 (ddd, *J* = 13.4, 11.5, 4.7 Hz, 1H)	1.66 (m, 1H); 1.39 (m, 1H)	-; –0.01
6'	-	-	-
7'	-	-	-
8'	2.20 – 2.11 (m, 1H); 1.71 – 1.60 (m, 1H)	2.15 (m, 1H); 1.63 (m, 1H)	+0.01 (center); -
9'	1.78 – 1.72 (m, 1H); 1.71 – 1.60 (m, 1H)	1.75 (m, 1H); 1.69 (m, 1H)	–0.01 (center); -
10'	2.10 – 2.07 (m, overlap with H_2O, 1H)	2.09 (m, overlap with H_2O, 1H)	0 (center)
11'	-	-	-
12'	0.89 (s, 3H)	0.90 (s, 3H)	–0.01
13'	1.01 (s, 3H)	1.01 (s, 3H)	0
14'	9.55 (s, 1H)	9.55 (s, 1H)	0
15'	1.29 (s, 3H)	1.30 (s, 3H)	–0.01

^{13}C NMR spectra and data comparison of our synthetic and natural[16] (+)-cochlearol B

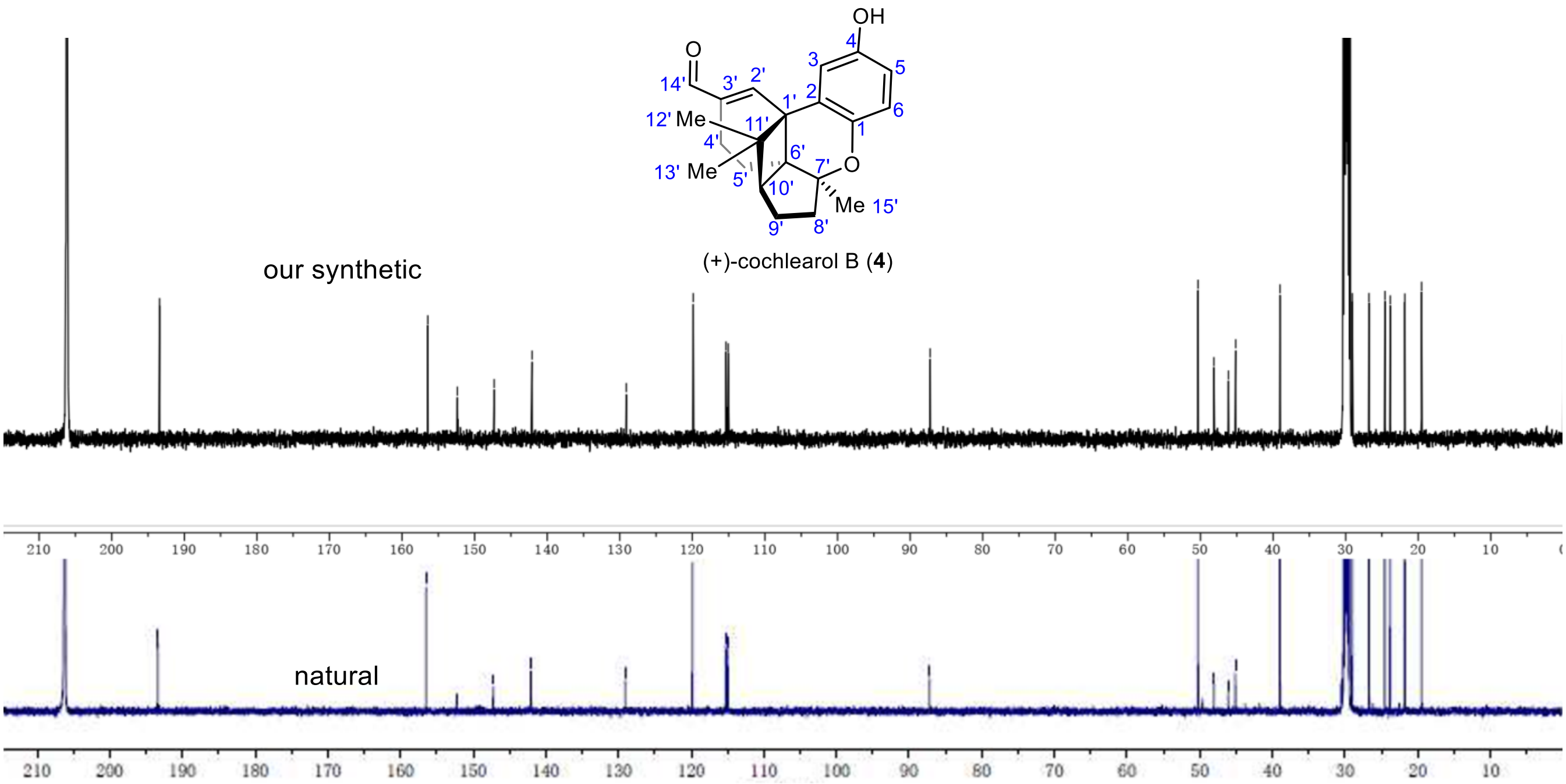

C	Our synthetic (125 MHz, $(CD_3)_2CO$)	Natural[16] (150 MHz, $(CD_3)_2CO$)	Δδ (Our synthetic – Natural)
1	152.4	156.4	-
2	129.1	129.0	+0.1
3	115.1	114.9	+0.2
4	147.3	147.2	+0.1
5	115.4	115.2	+0.2
6	119.9	119.8	+0.1
1'	46.1	46.1	0
2'	156.4	152.2	-
3'	142.1	142.0	+0.1
4'	19.5	19.5	0
5'	26.8	26.7	+0.1
6'	48.2	45.1	-
7'	87.2	87.2	0
8'	39.0	38.9	+0.1
9'	24.6	24.5	+0.1
10'	50.3	50.2	+0.1
11'	45.1	48.1	-
12'	21.8	21.8	0
13'	29.1	29.0	+0.1
14'	193.4	193.4	0
15'	23.8	23.8	0

***Note**: The ^{1}H and ^{13}C NMR spectra of our synthesized cochlearol B are in full agreement with those reported in the isolation literature[16]. We revised the ^{13}C assignments (C1, C2', C6', and C11') from the isolation literature.*

7. ^{1}H and ^{13}C NMR Spectra of the Synthetic Intermediates, Ganocins A–C and Cochlearol B

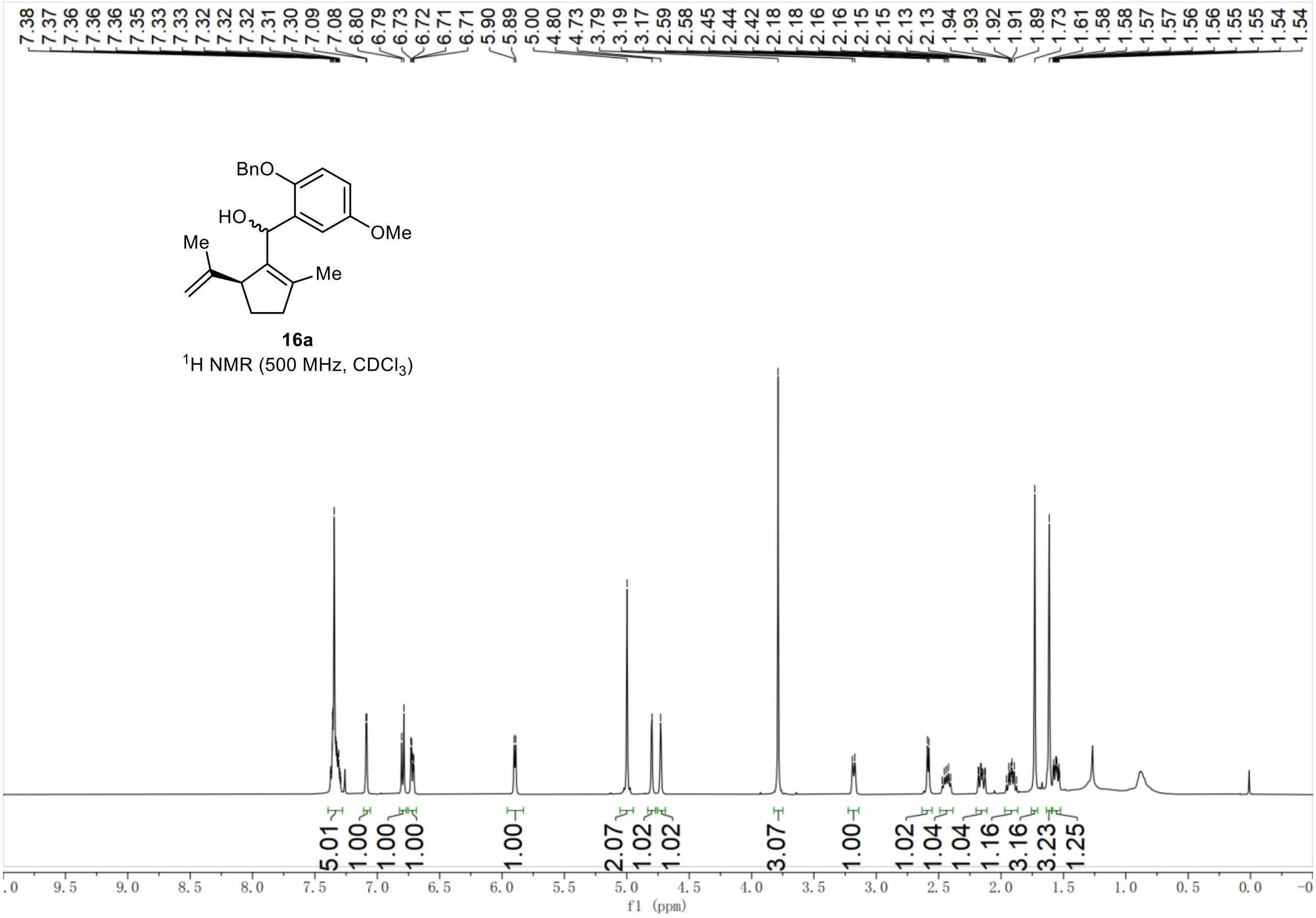

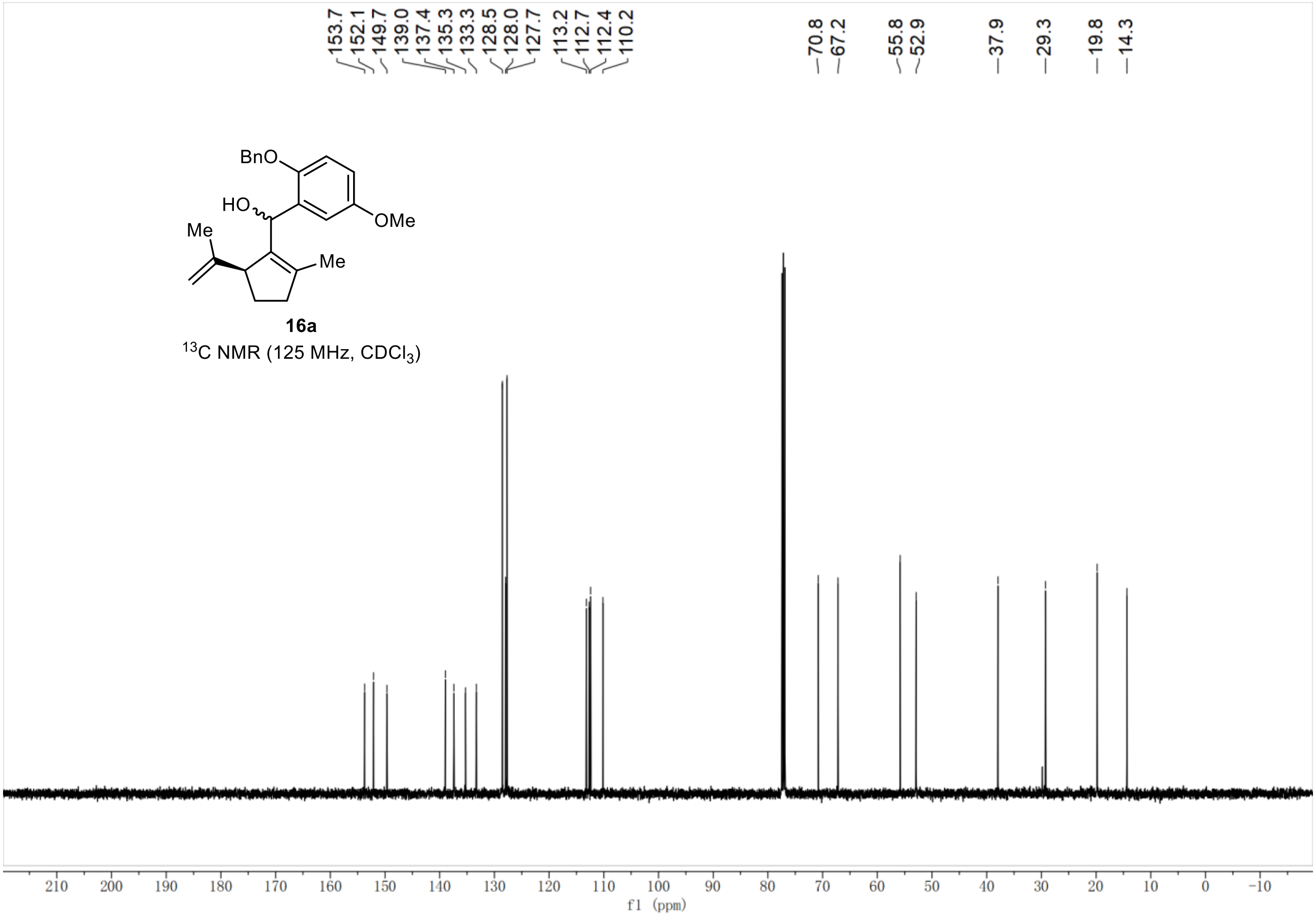

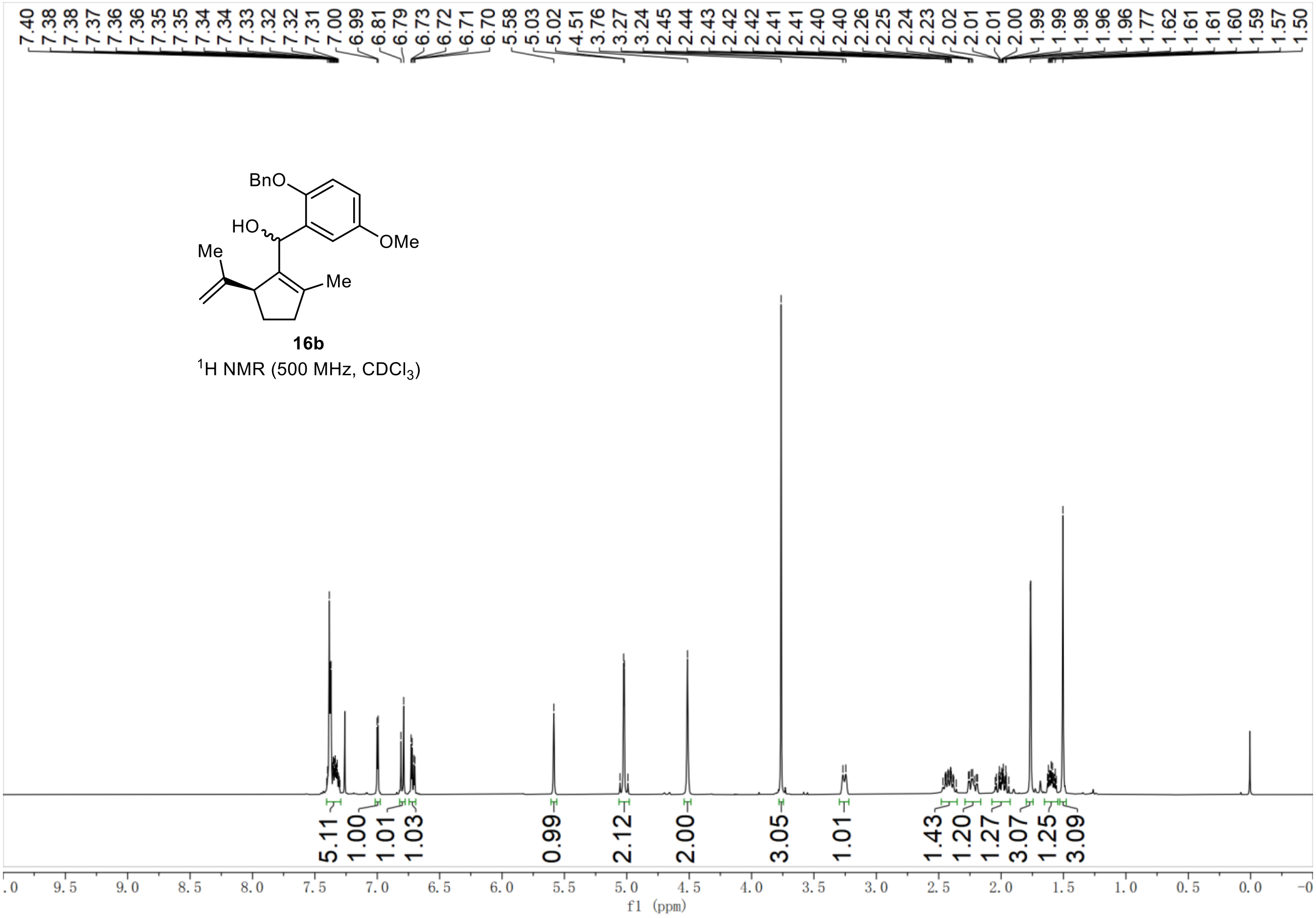
BnO
HO
OMe
Me
Me
16b
1H NMR (500 MHz, CDCl3)
f1 (ppm)

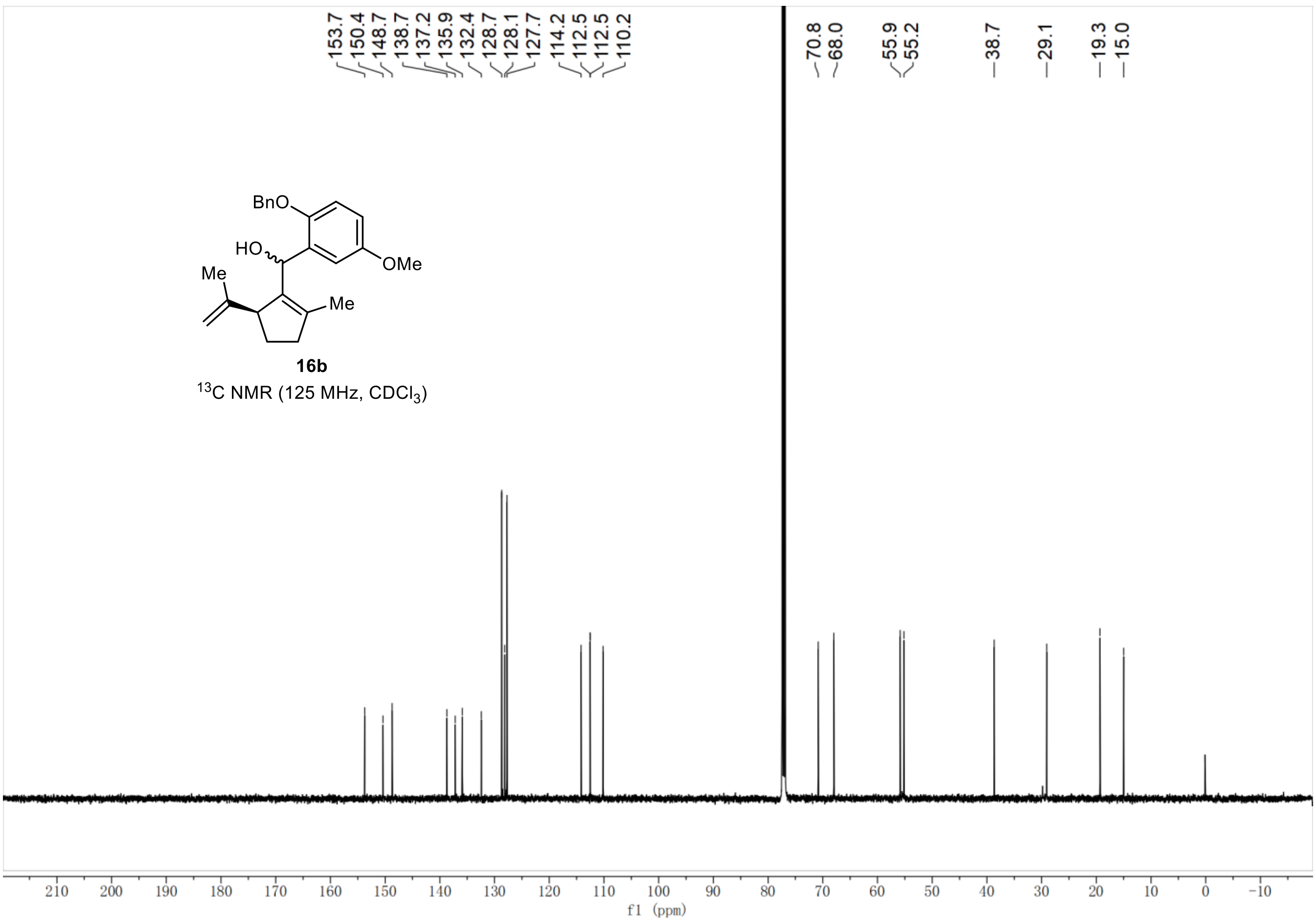
BnO
HO
OMe
Me
Me
16b
13C NMR (125 MHz, CDCl3)
f1 (ppm)

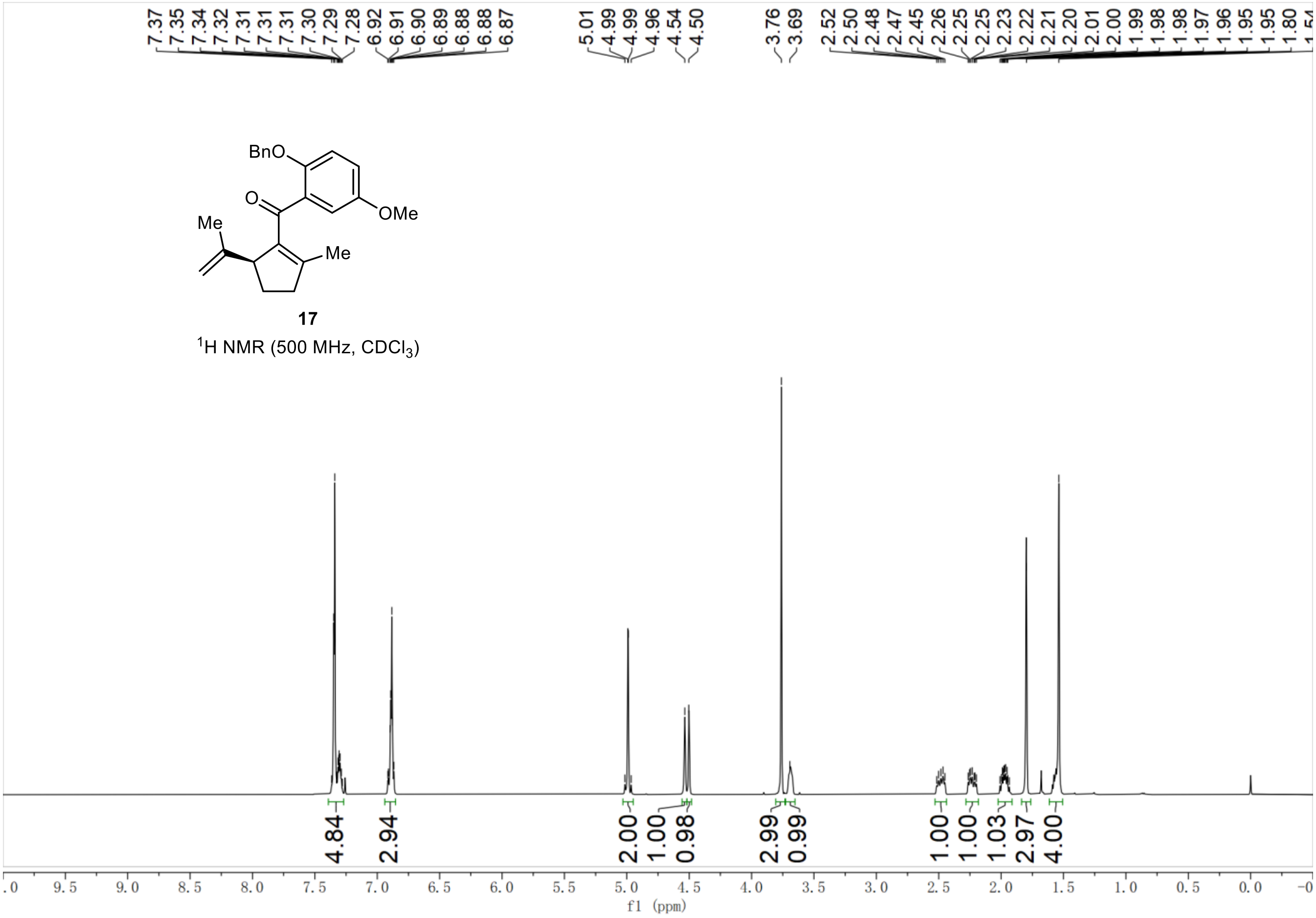
7.37 7.35 7.34 7.32 7.31 7.31 7.31 7.30 7.29 7.28 6.92 6.91 6.90 6.89 6.88 6.88 6.87
5.01 4.99 4.99 4.96 4.54 4.50
3.76 3.69
2.52 2.50 2.48 2.47 2.45 2.26 2.25 2.25 2.23 2.22 2.21 2.20 2.01 2.00 1.99 1.98 1.98 1.97 1.96 1.95 1.95 1.80
BnO
O
OMe
Me
Me
17
1H NMR (500 MHz, CDCl3)
4.84 2.94 2.00 1.00 0.98 2.99 0.99 1.00 1.00 1.03 2.97 4.00
f1 (ppm)

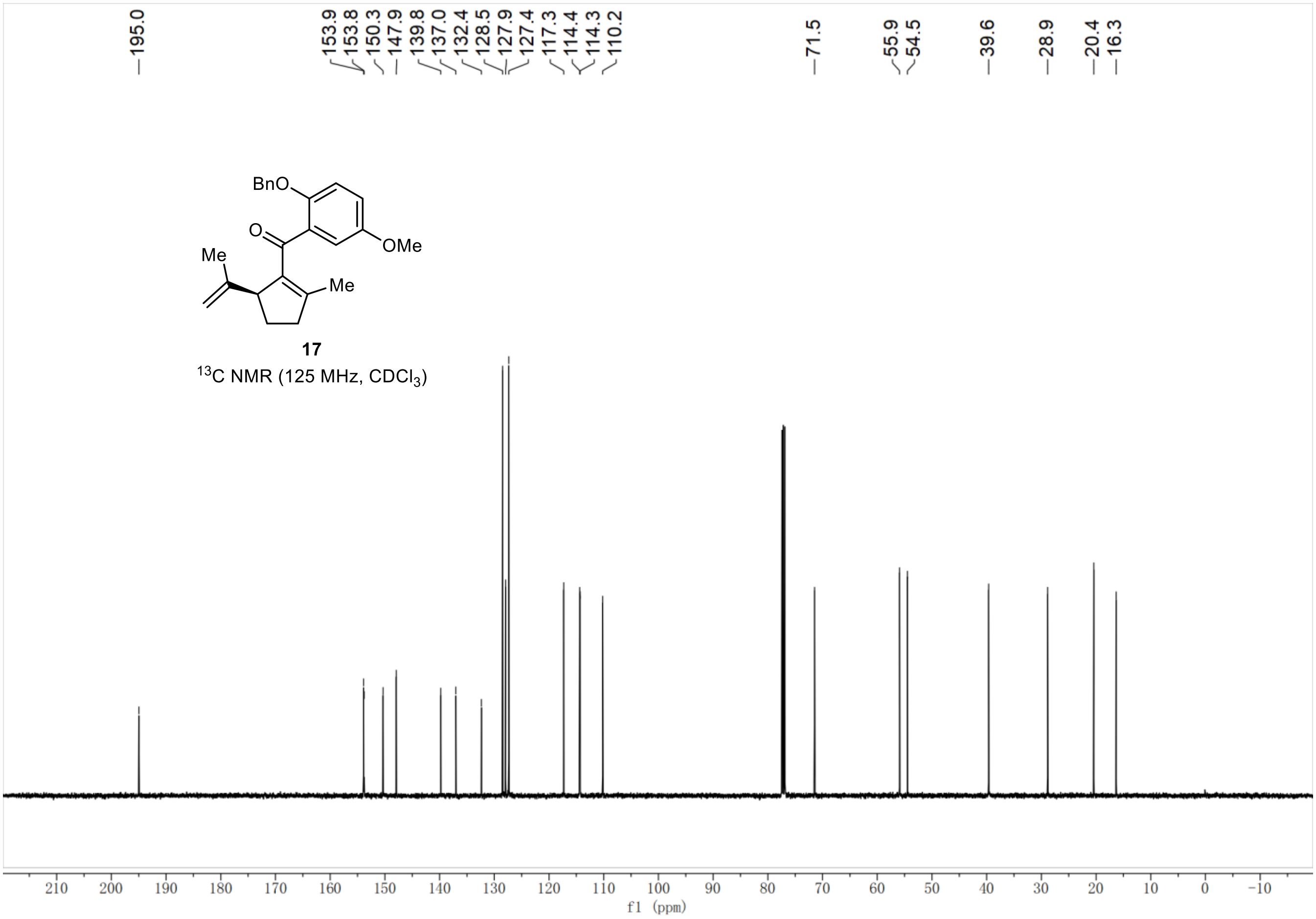
195.0
153.9 153.8 150.3 147.9 139.8 137.0 132.4 128.5 127.9 127.4 117.3 114.4 114.3 110.2
71.5
55.9 54.5
39.6
28.9
20.4 16.3
BnO
O
OMe
Me
Me
17
13C NMR (125 MHz, CDCl3)
f1 (ppm)

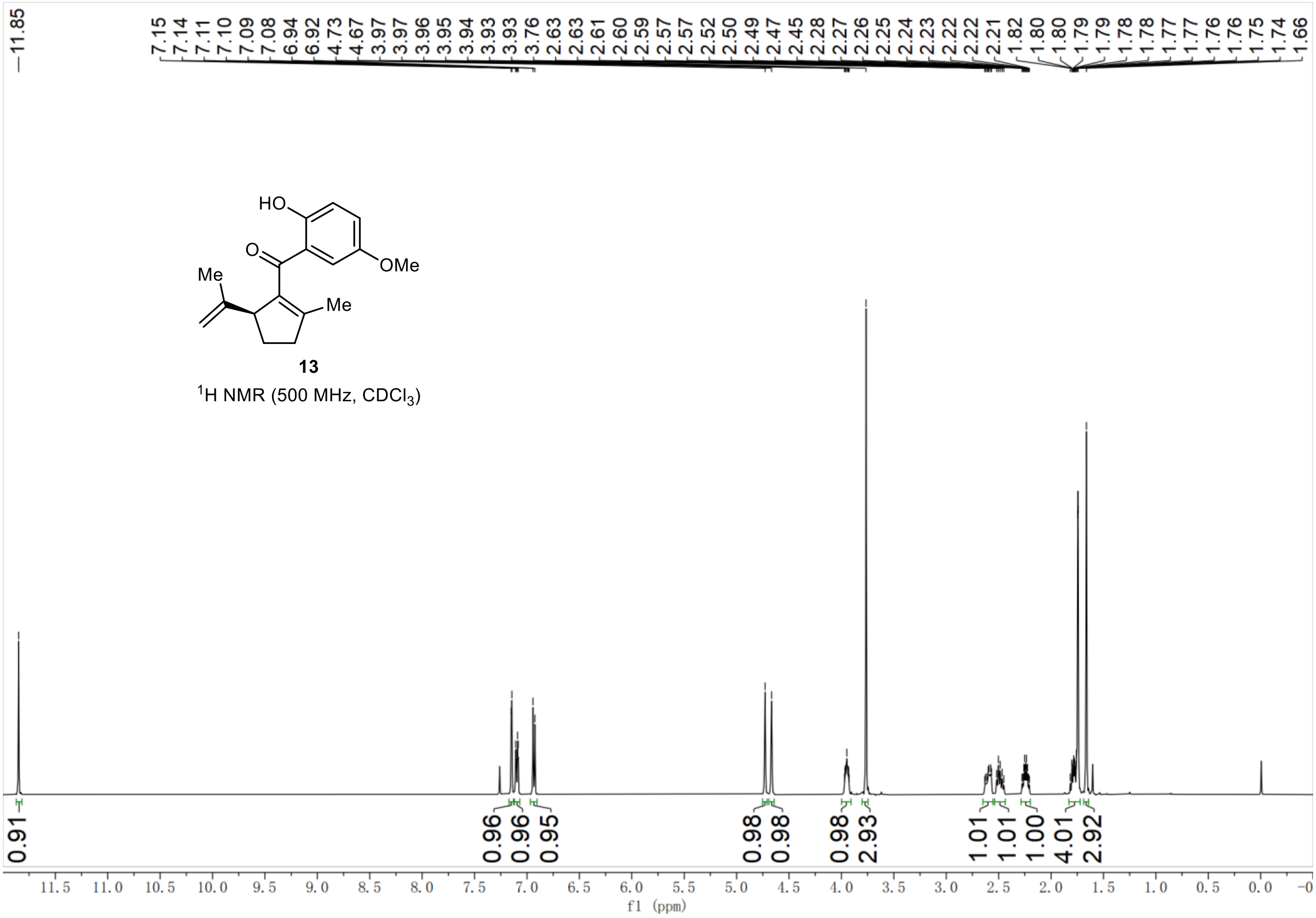
11.85
7.15
7.14
7.11
7.10
7.09
7.08
6.94
6.92
4.73
4.67
3.97
3.97
3.96
3.95
3.94
3.93
3.93
3.76
2.63
2.63
2.61
2.60
2.59
2.57
2.57
2.52
2.50
2.49
2.47
2.45
2.28
2.27
2.26
2.25
2.24
2.23
2.23
2.22
2.22
2.21
1.82
1.80
1.80
1.79
1.79
1.78
1.78
1.77
1.77
1.76
1.76
1.75
1.74
1.66
HO
O
OMe
Me
Me
13
^{1}H NMR (500 MHz, CDCl$_3$)
0.91
0.96
0.96
0.95
0.98
0.98
0.98
2.93
1.01
1.01
1.00
4.01
2.92
f1 (ppm)

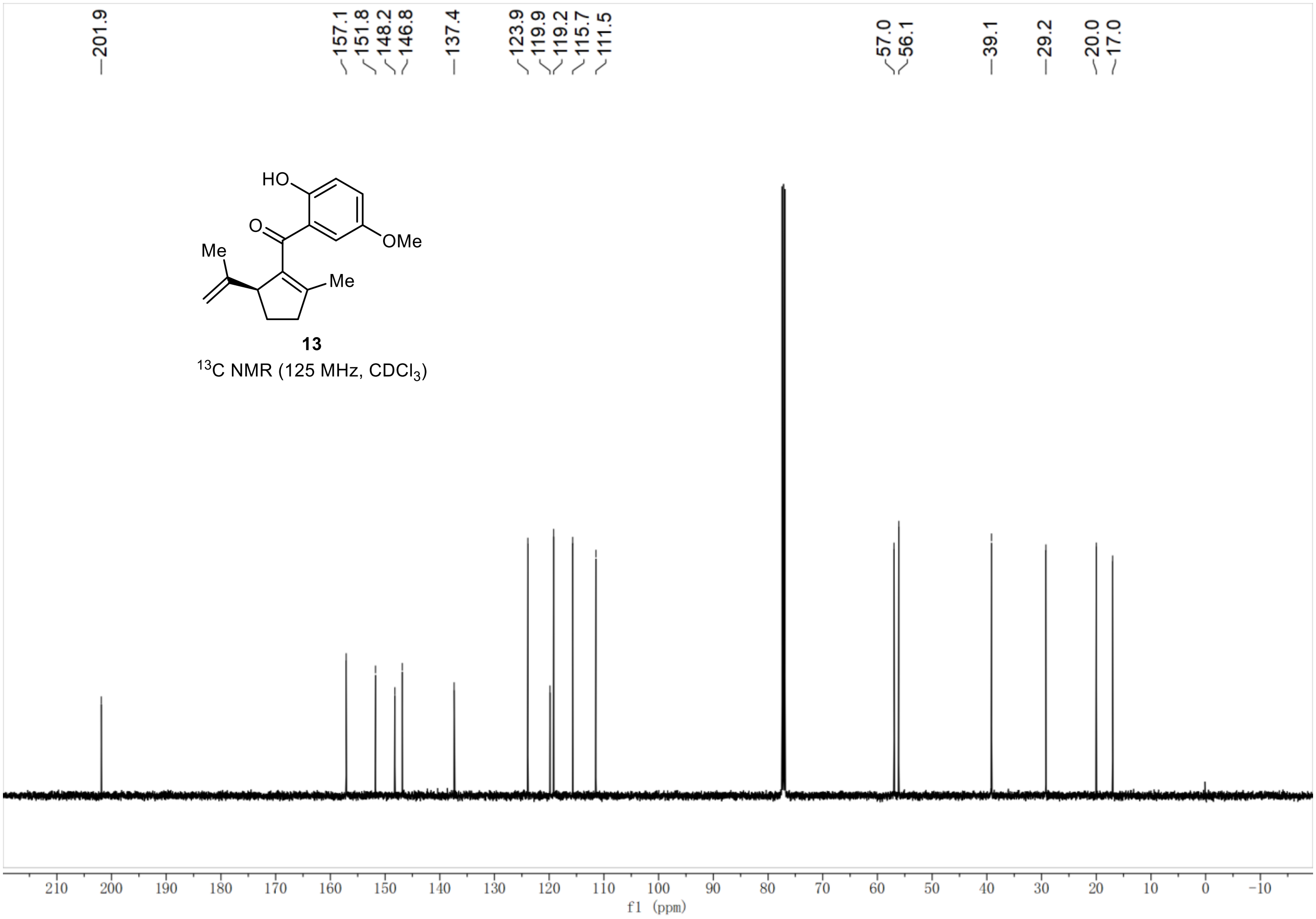
201.9
157.1
151.8
148.2
146.8
137.4
123.9
119.9
119.2
115.7
111.5
57.0
56.1
39.1
29.2
20.0
17.0
HO
O
OMe
Me
Me
13
^{13}C NMR (125 MHz, CDCl$_3$)
f1 (ppm)

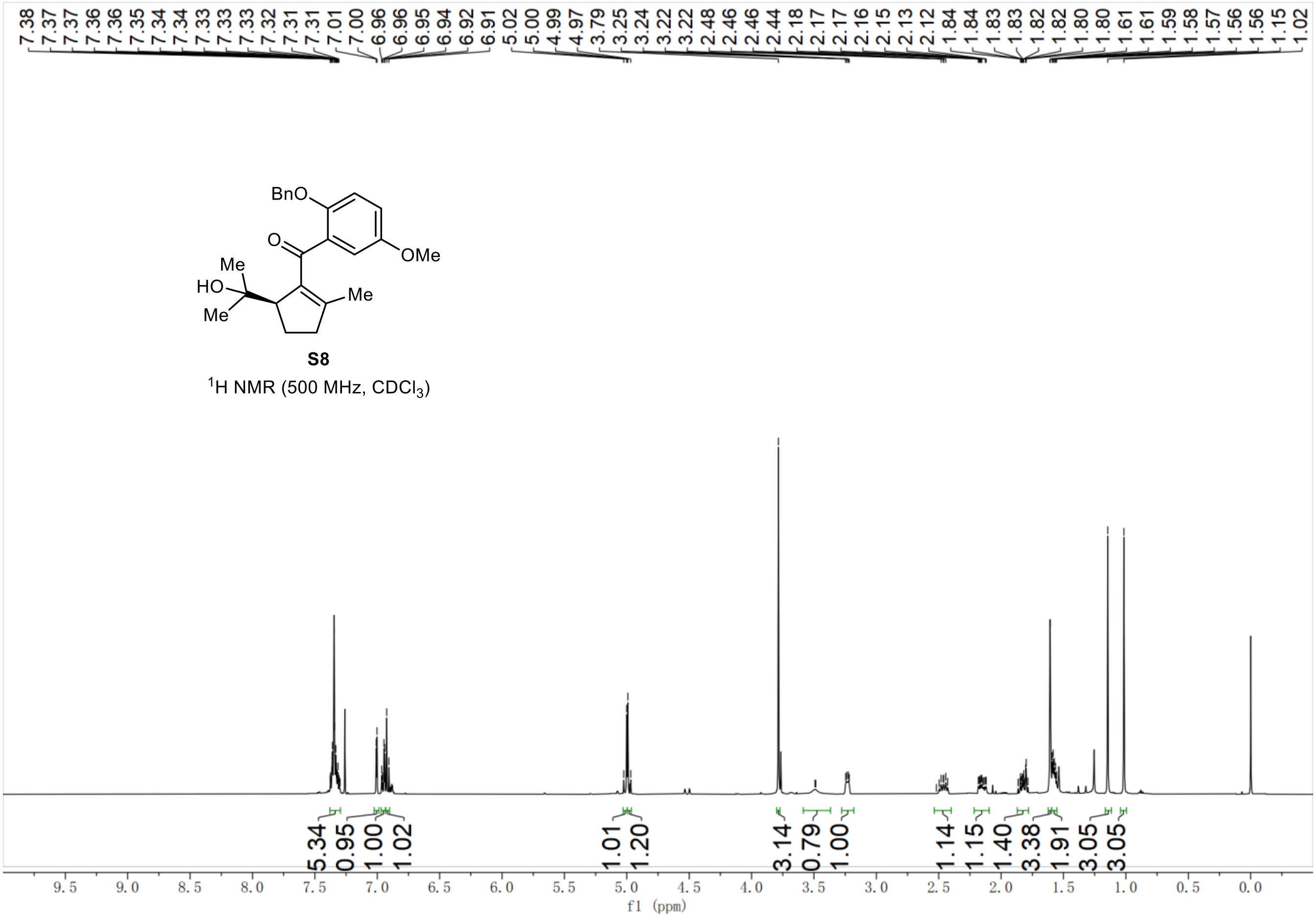
7.38 7.37 7.37 7.36 7.36 7.35 7.34 7.34 7.33 7.33 7.33 7.32 7.31 7.31 7.01 7.00 6.96 6.96 6.95 6.94 6.92 6.91 5.02 5.00 4.99 4.97 3.79 3.25 3.24 3.22 3.22 2.48 2.46 2.46 2.44 2.18 2.17 2.17 2.16 2.15 2.13 2.12 1.84 1.84 1.83 1.83 1.82 1.82 1.80 1.61 1.61 1.59 1.58 1.57 1.56 1.56 1.15 1.02
BnO
O
OMe
Me
HO
Me
Me
S8
^{1}H NMR (500 MHz, CDCl$_3$)
5.34 0.95 1.00 1.02 1.01 1.20 3.14 0.79 1.00 1.14 1.15 1.40 3.38 1.91 3.05 3.05
f1 (ppm)

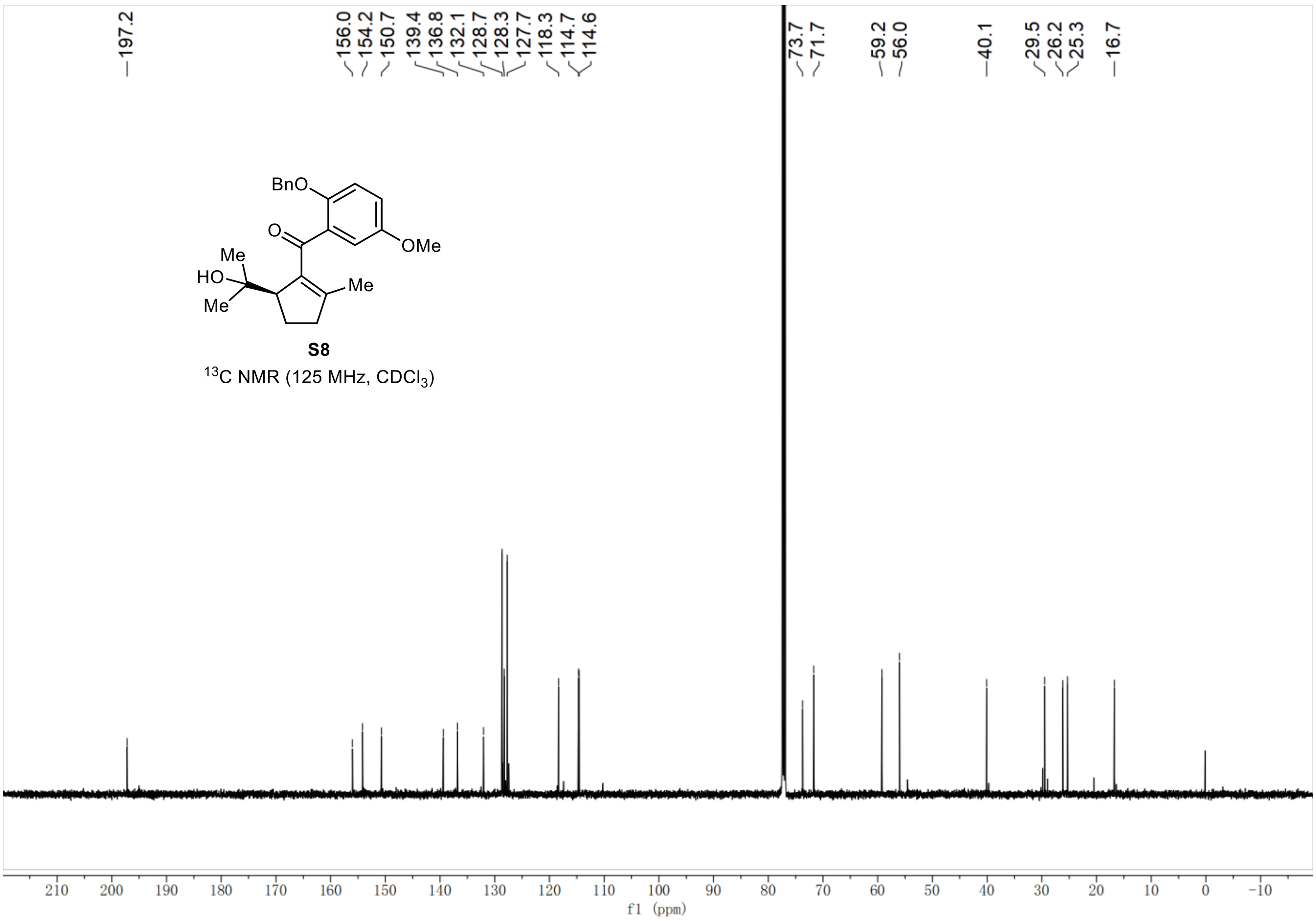
197.2
156.0 154.2 150.7 139.4 136.8 132.1 128.7 128.3 127.7 118.3 114.7 114.6
73.7 71.7 59.2 56.0 40.1 29.5 26.2 25.3 16.7
BnO
O
OMe
Me
HO
Me
Me
S8
^{13}C NMR (125 MHz, CDCl$_3$)
f1 (ppm)

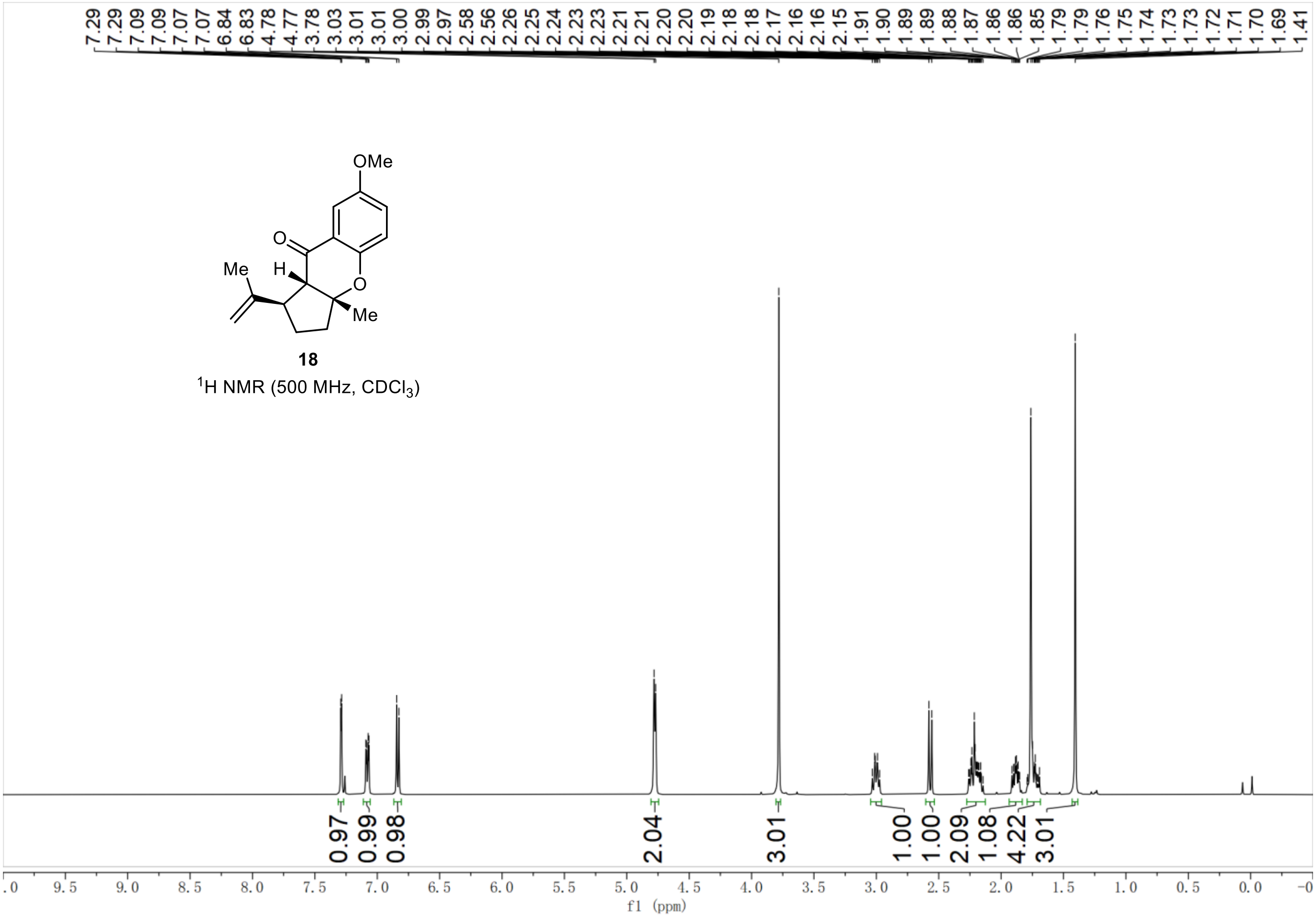
7.29 7.29 7.09 7.09 7.07 7.07 6.84 6.83 4.78 4.77 3.78 3.03 3.01 3.01 3.00 2.99 2.97 2.58 2.56 2.26 2.25 2.24 2.23 2.23 2.21 2.21 2.20 2.20 2.19 2.18 2.18 2.17 2.16 2.16 2.15 1.91 1.90 1.89 1.89 1.88 1.87 1.86 1.86 1.85 1.79 1.79 1.76 1.75 1.74 1.73 1.73 1.72 1.71 1.70 1.69 1.41
OMe
O
H
Me
O
Me
18
^{1}H NMR (500 MHz, CDCl$_3$)
0.97 0.99 0.98 2.04 3.01 1.00 1.00 2.09 1.08 4.22 3.01
f1 (ppm)

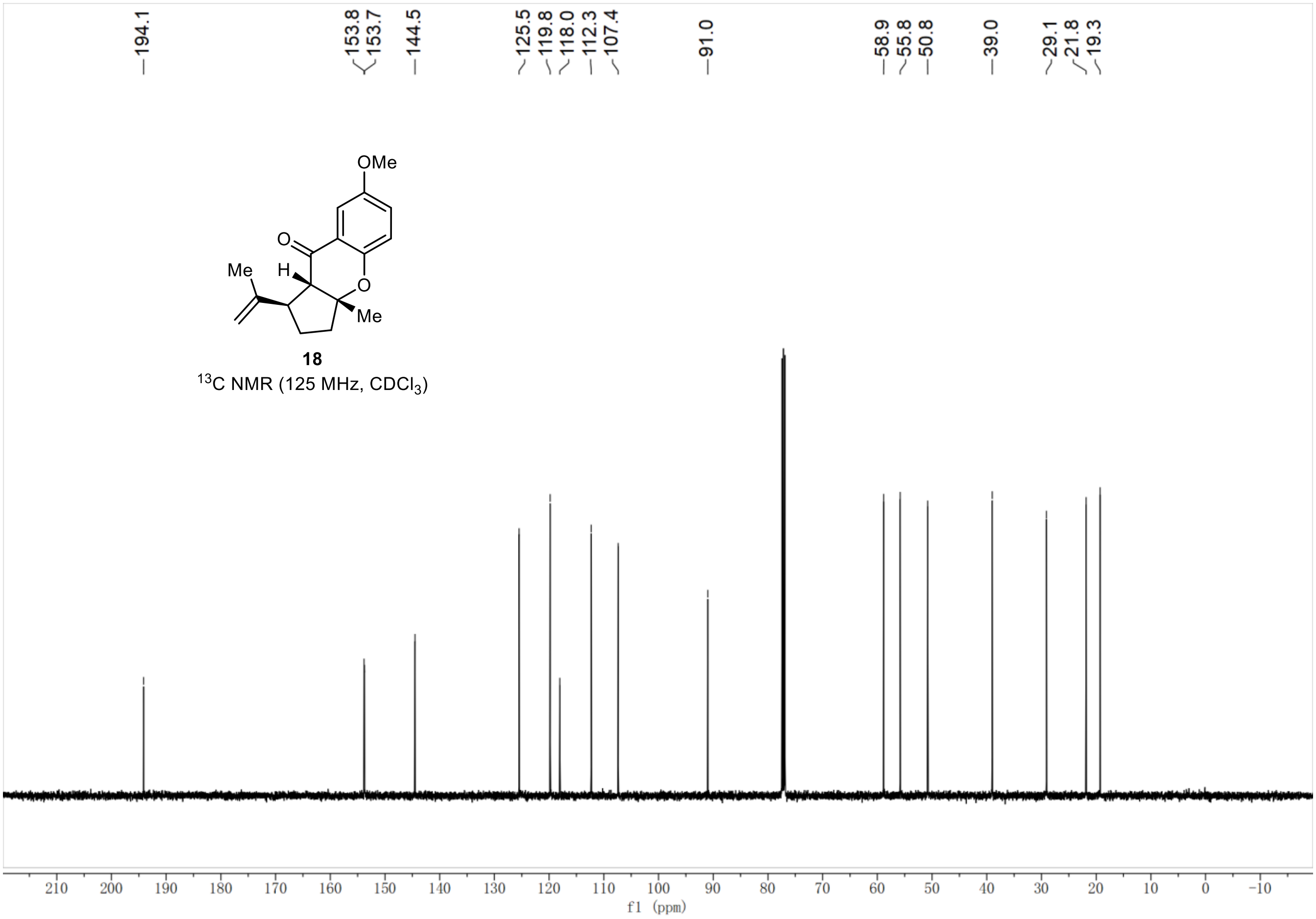
194.1 153.8 153.7 144.5 125.5 119.8 118.0 112.3 107.4 91.0 58.9 55.8 50.8 39.0 29.1 21.8 19.3
OMe
O
H
Me
O
Me
18
^{13}C NMR (125 MHz, CDCl$_3$)
f1 (ppm)

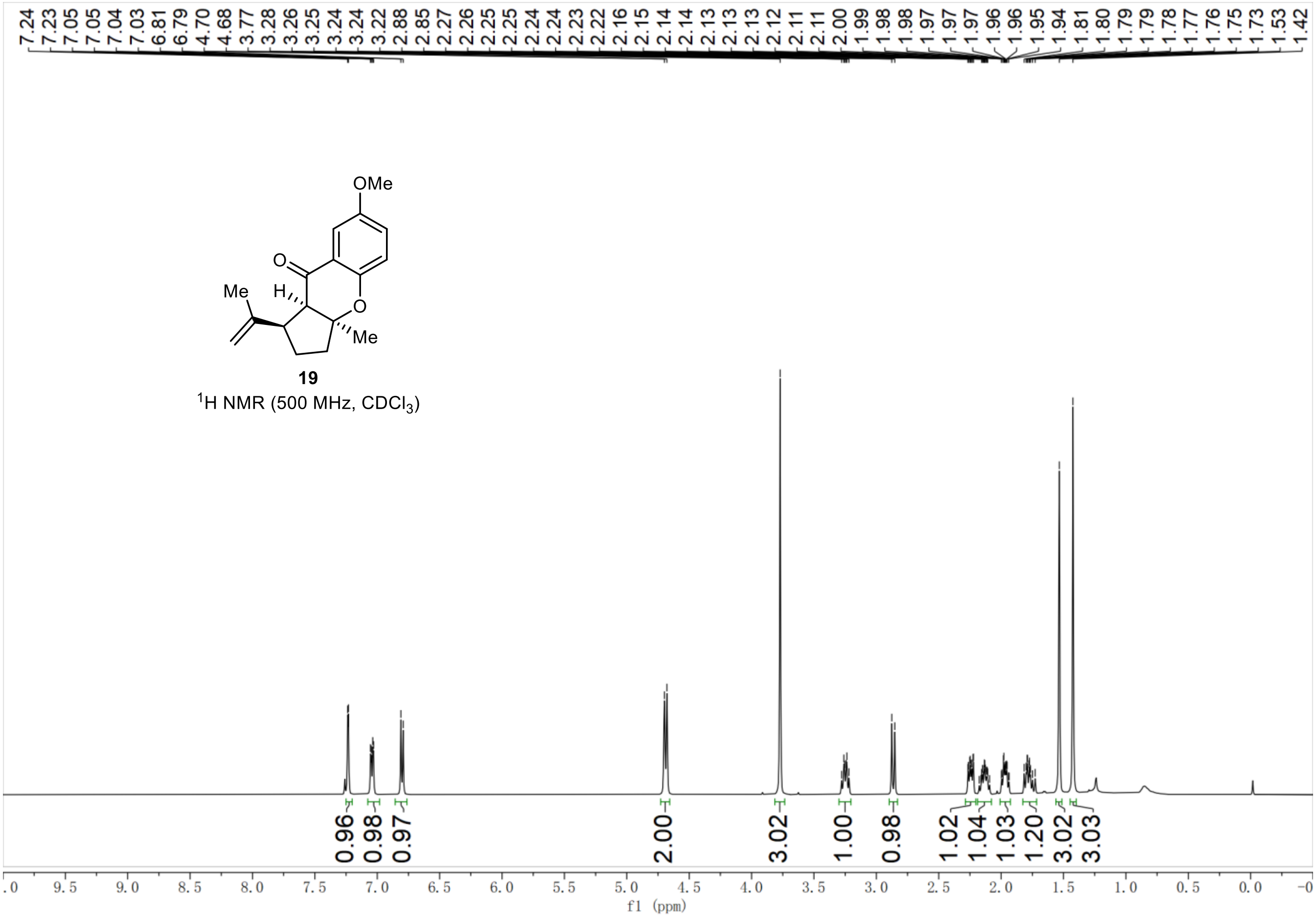
7.24 7.23 7.05 7.05 7.04 7.03 6.81 6.79 4.70 4.68 3.77 3.28 3.26 3.25 3.24 3.24 3.22 2.88 2.85 2.27 2.26 2.25 2.25 2.24 2.24 2.23 2.22 2.16 2.15 2.14 2.14 2.13 2.13 2.13 2.12 2.11 2.11 2.00 1.99 1.98 1.98 1.97 1.97 1.97 1.96 1.96 1.95 1.94 1.81 1.80 1.79 1.79 1.78 1.77 1.76 1.75 1.73 1.53 1.42
OMe
O
Me
H
O
Me
19
1H NMR (500 MHz, CDCl3)
0.96 0.98 0.97 2.00 3.02 1.00 0.98 1.02 1.04 1.03 1.20 3.02 3.03
10.0 9.5 9.0 8.5 8.0 7.5 7.0 6.5 6.0 5.5 5.0 4.5 4.0 3.5 3.0 2.5 2.0 1.5 1.0 0.5 0.0 -0
f1 (ppm)

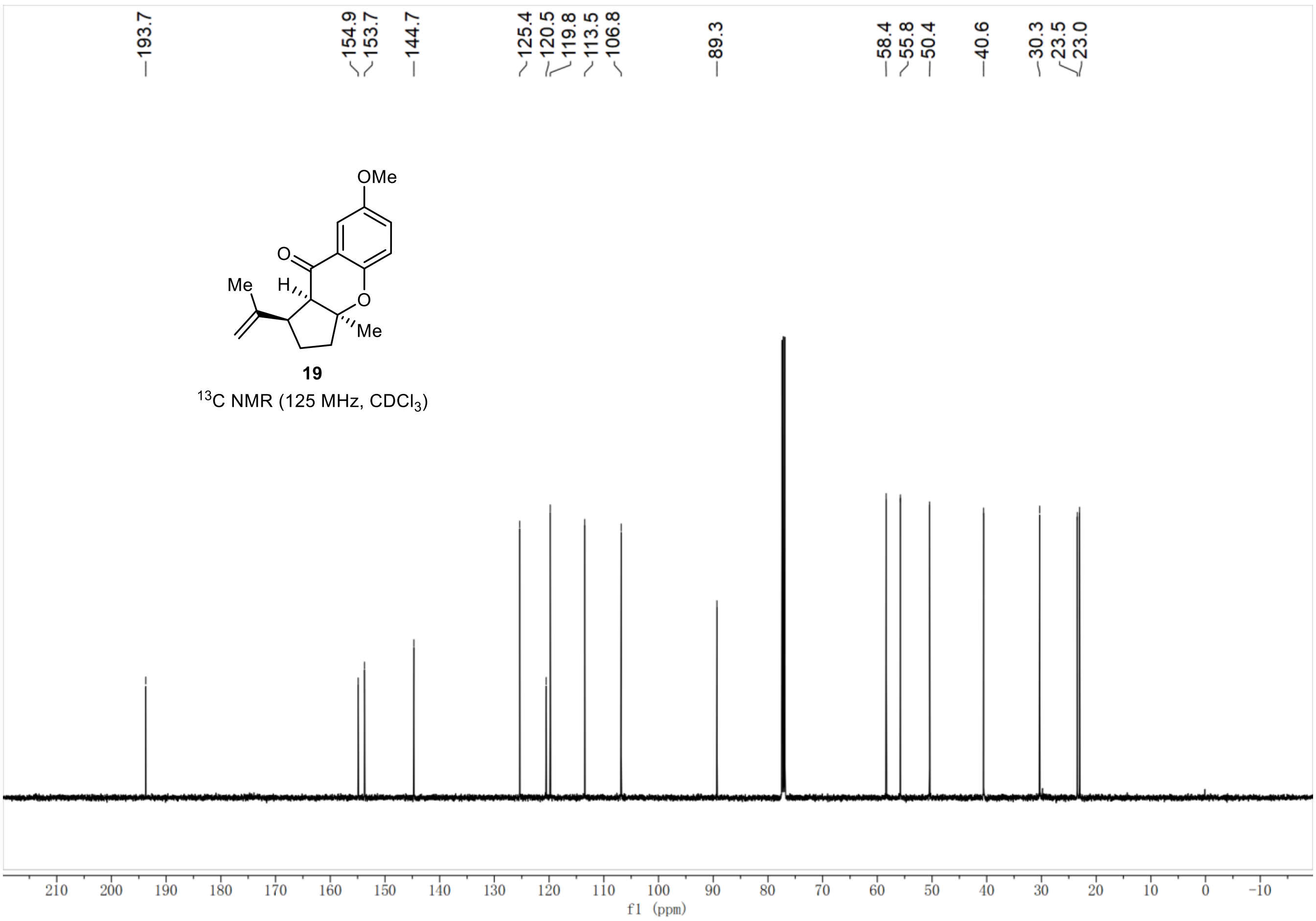
193.7 154.9 153.7 144.7 125.4 120.5 119.8 113.5 106.8 89.3 58.4 55.8 50.4 40.6 30.3 23.5 23.0
OMe
O
Me
H
O
Me
19
13C NMR (125 MHz, CDCl3)
210 200 190 180 170 160 150 140 130 120 110 100 90 80 70 60 50 40 30 20 10 0 -10
f1 (ppm)

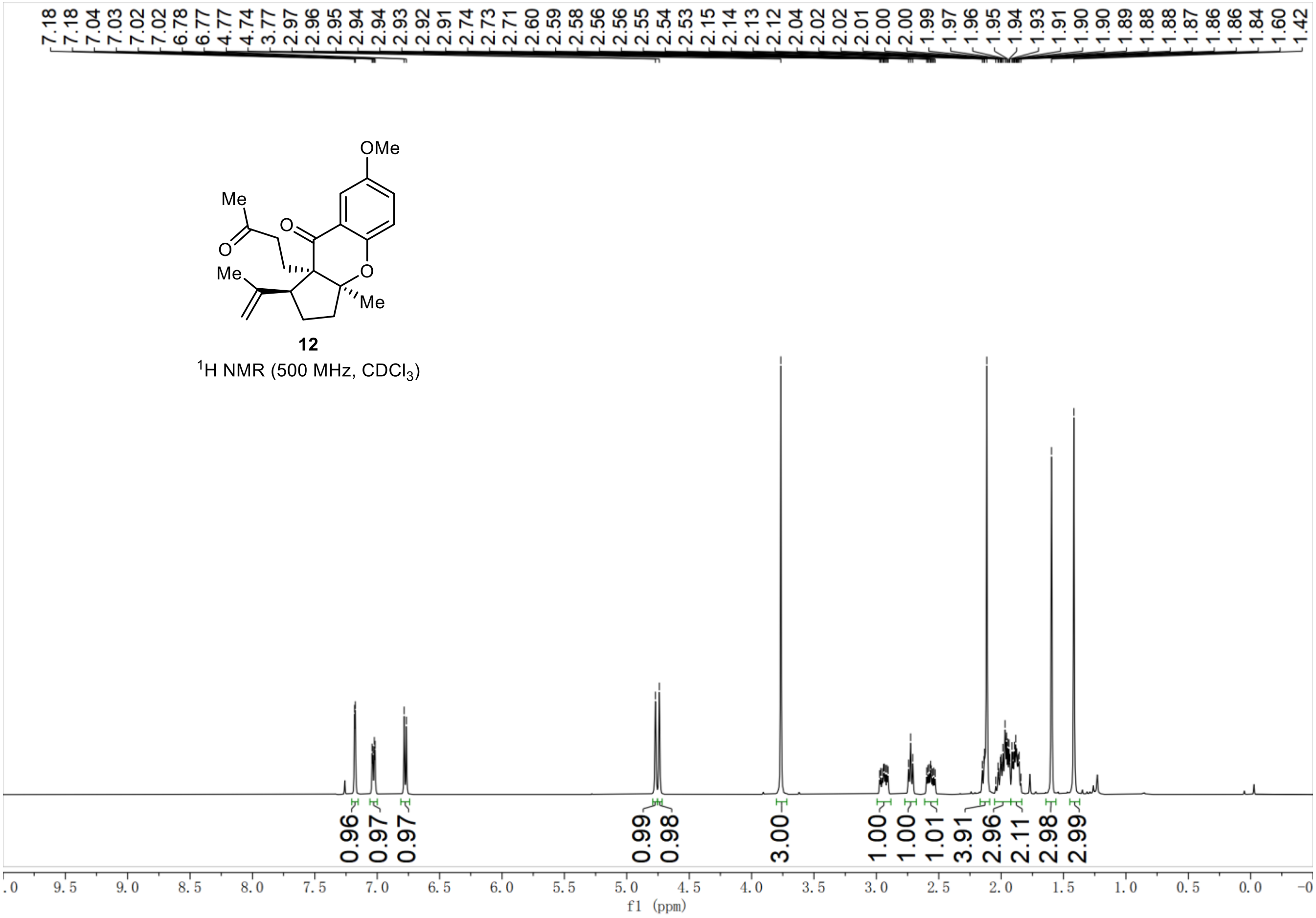

12

^{1}H NMR (500 MHz, $CDCl_3$)

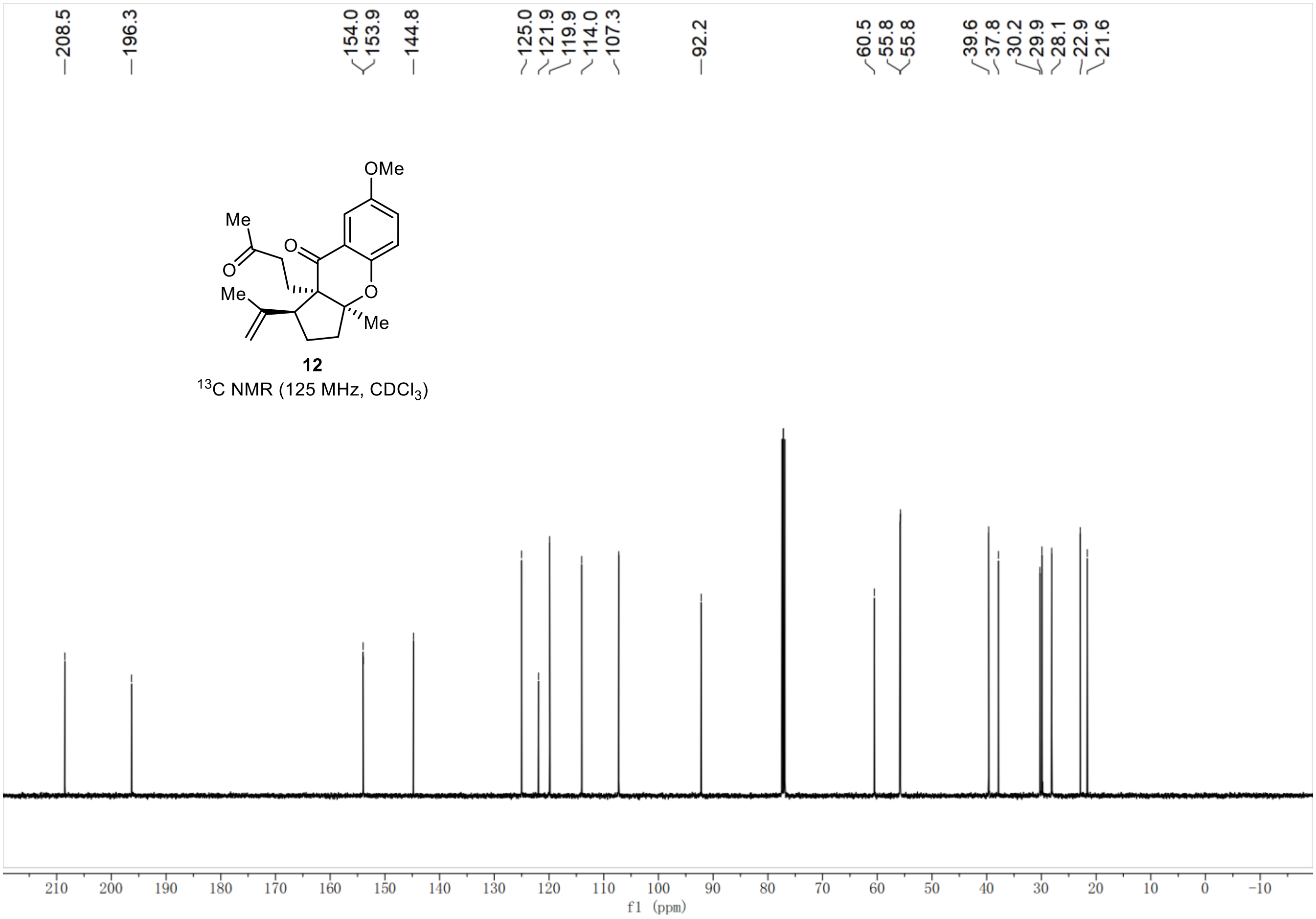

12

^{13}C NMR (125 MHz, $CDCl_3$)

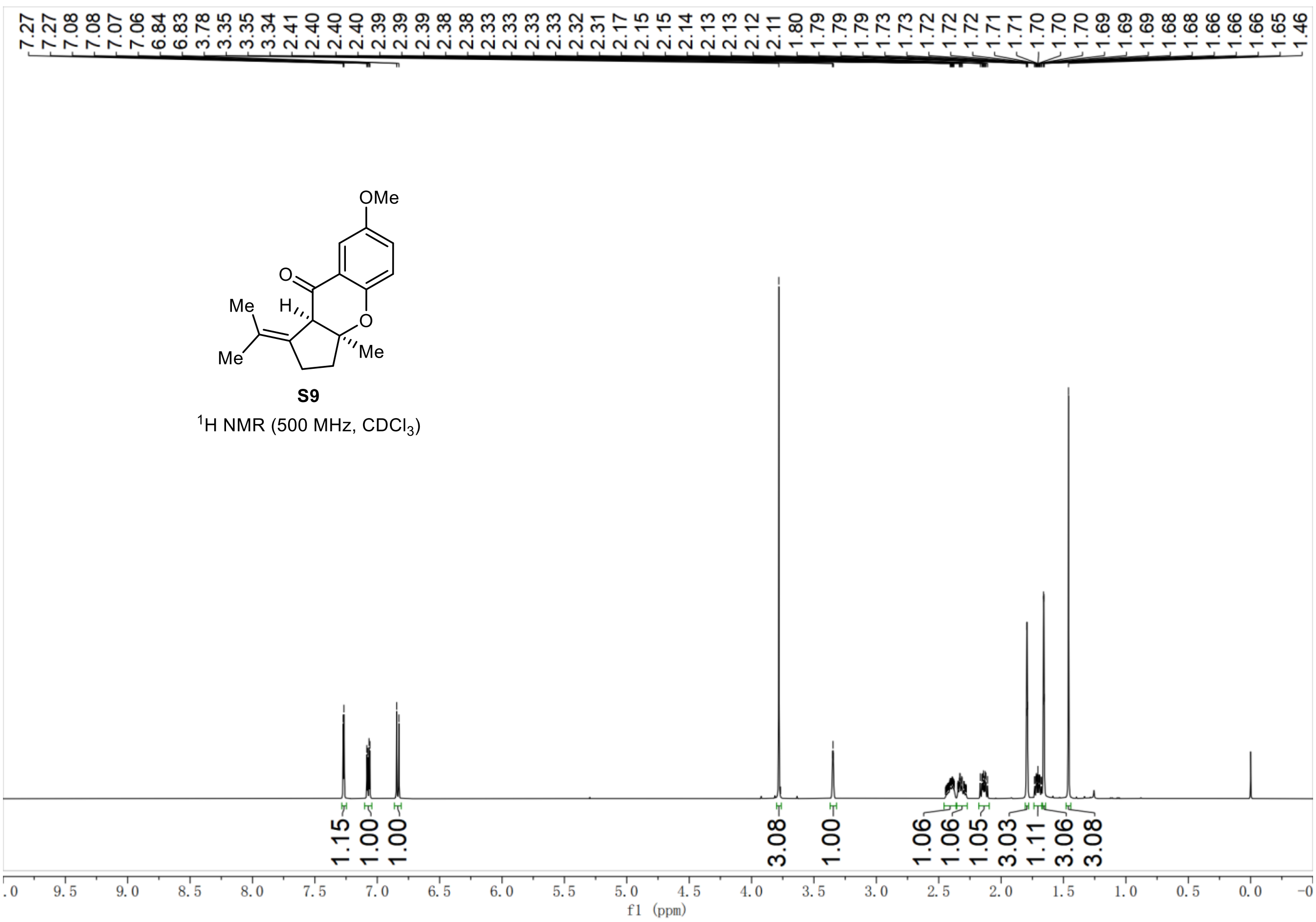
7.27 7.27 7.08 7.08 7.07 7.06 6.84 6.83 3.78 3.35 3.35 3.34 2.41 2.40 2.40 2.39 2.39 2.39 2.38 2.38 2.33 2.33 2.33 2.33 2.32 2.31 2.17 2.15 2.15 2.14 2.13 2.13 2.12 2.11 1.80 1.79 1.79 1.73 1.73 1.72 1.72 1.72 1.71 1.71 1.70 1.70 1.70 1.69 1.69 1.69 1.68 1.68 1.66 1.66 1.66 1.65 1.46
OMe
O
H
O
Me
Me
Me
S9
^{1}H NMR (500 MHz, CDCl$_3$)
1.15 1.00 1.00 3.08 1.00 1.06 1.06 1.05 3.03 1.11 3.06 3.08
f1 (ppm)

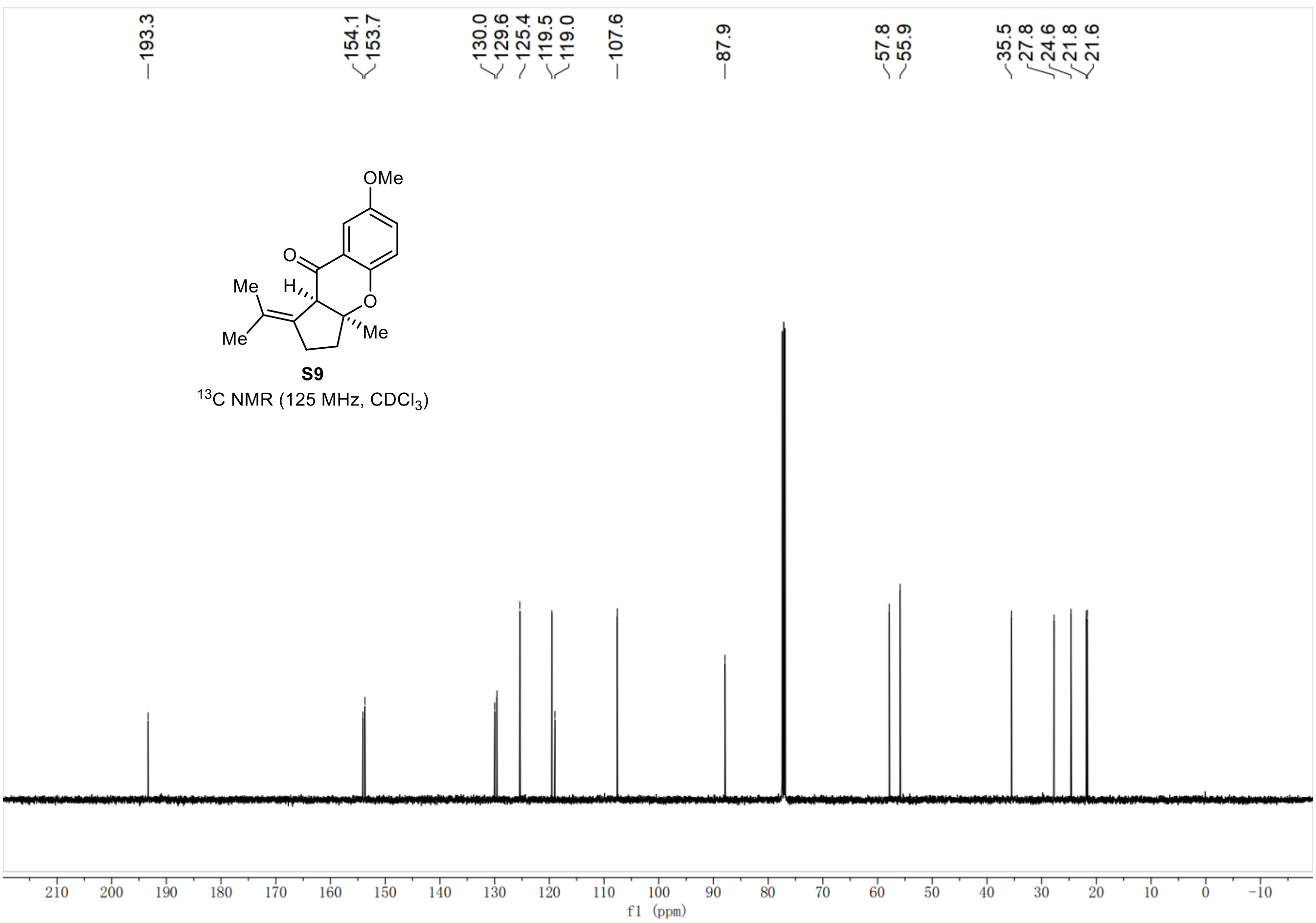
193.3
154.1 153.7
130.0 129.6 125.4 119.5 119.0
107.6
87.9
57.8 55.9
35.5 27.8 24.6 21.8 21.6
OMe
O
H
O
Me
Me
Me
S9
^{13}C NMR (125 MHz, CDCl$_3$)
f1 (ppm)

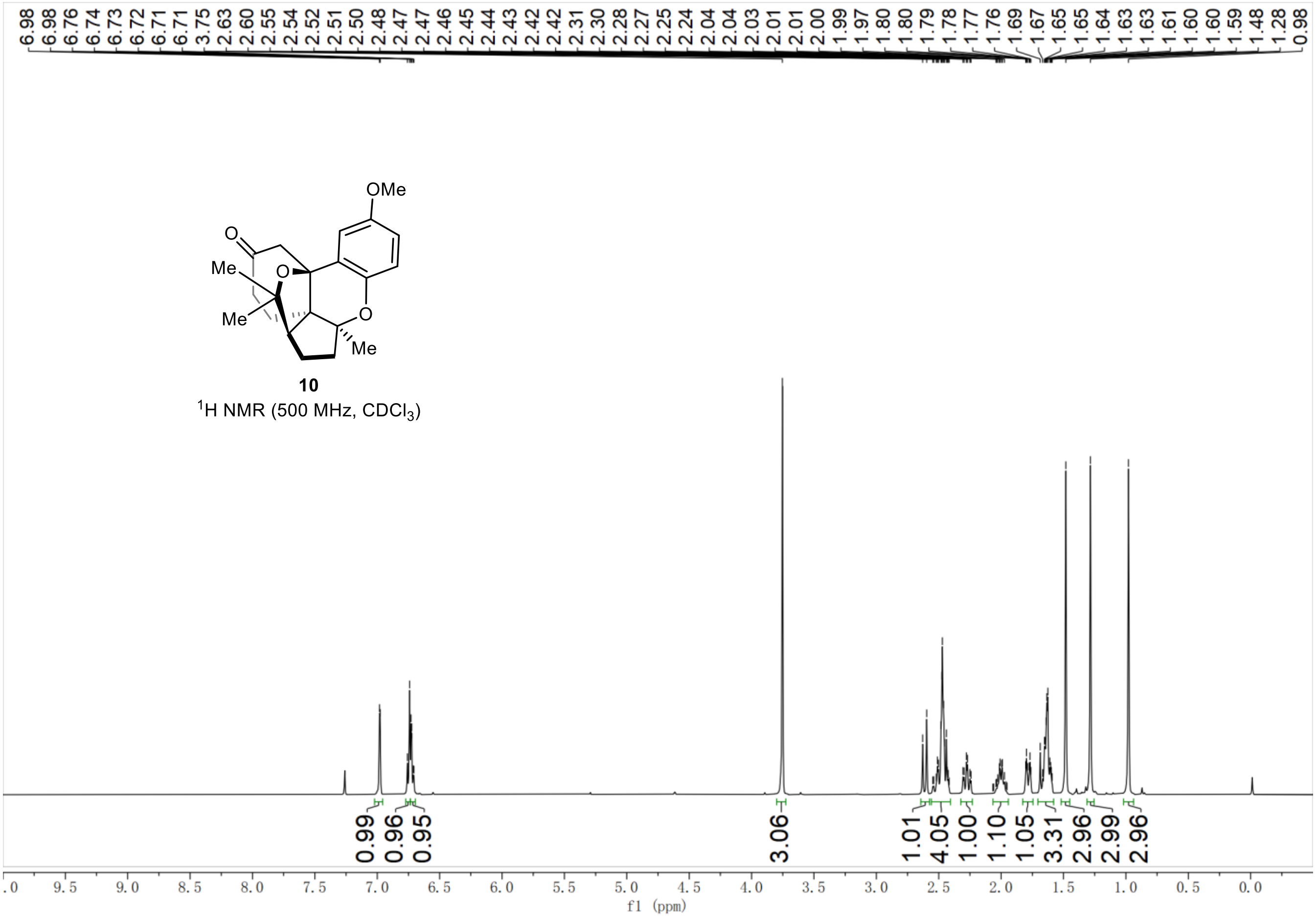

10

^{1}H NMR (500 MHz, $CDCl_3$)

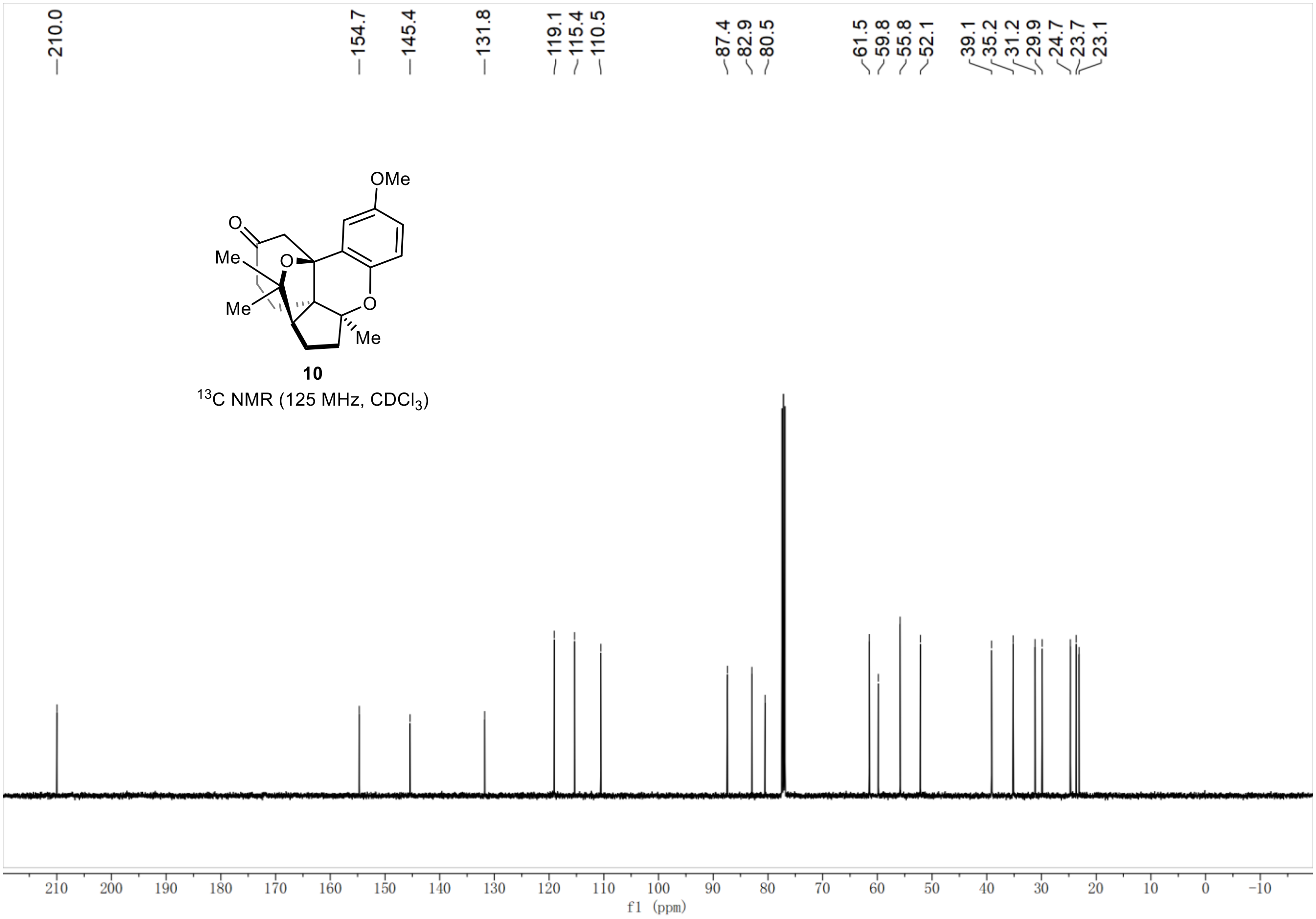

10

^{13}C NMR (125 MHz, $CDCl_3$)

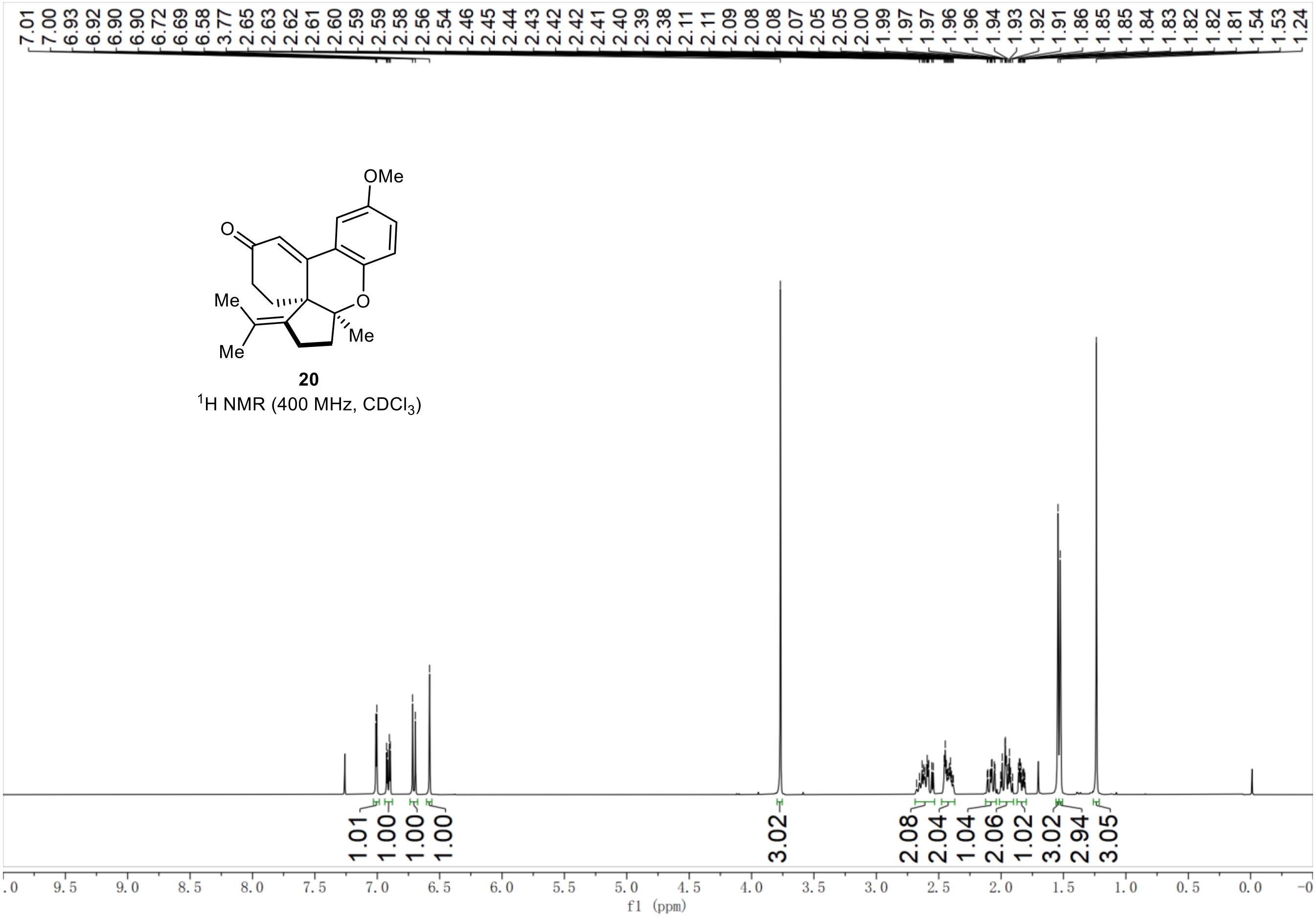

7.01
7.00
6.93
6.92
6.90
6.90
6.72
6.69
6.58
3.77
2.65
2.63
2.62
2.61
2.60
2.59
2.59
2.58
2.56
2.54
2.46
2.45
2.44
2.43
2.42
2.42
2.41
2.40
2.39
2.38
2.11
2.11
2.09
2.08
2.08
2.07
2.05
2.05
2.00
1.99
1.97
1.97
1.96
1.96
1.94
1.93
1.92
1.91
1.86
1.85
1.85
1.84
1.83
1.82
1.82
1.81
1.54
1.53
1.24
OMe
O
Me
O
Me
Me
20
^{1}H NMR (400 MHz, $CDCl_3$)
1.01
1.00
1.00
1.00
3.02
2.08
2.04
1.04
2.06
1.02
3.02
2.94
3.05
f1 (ppm)

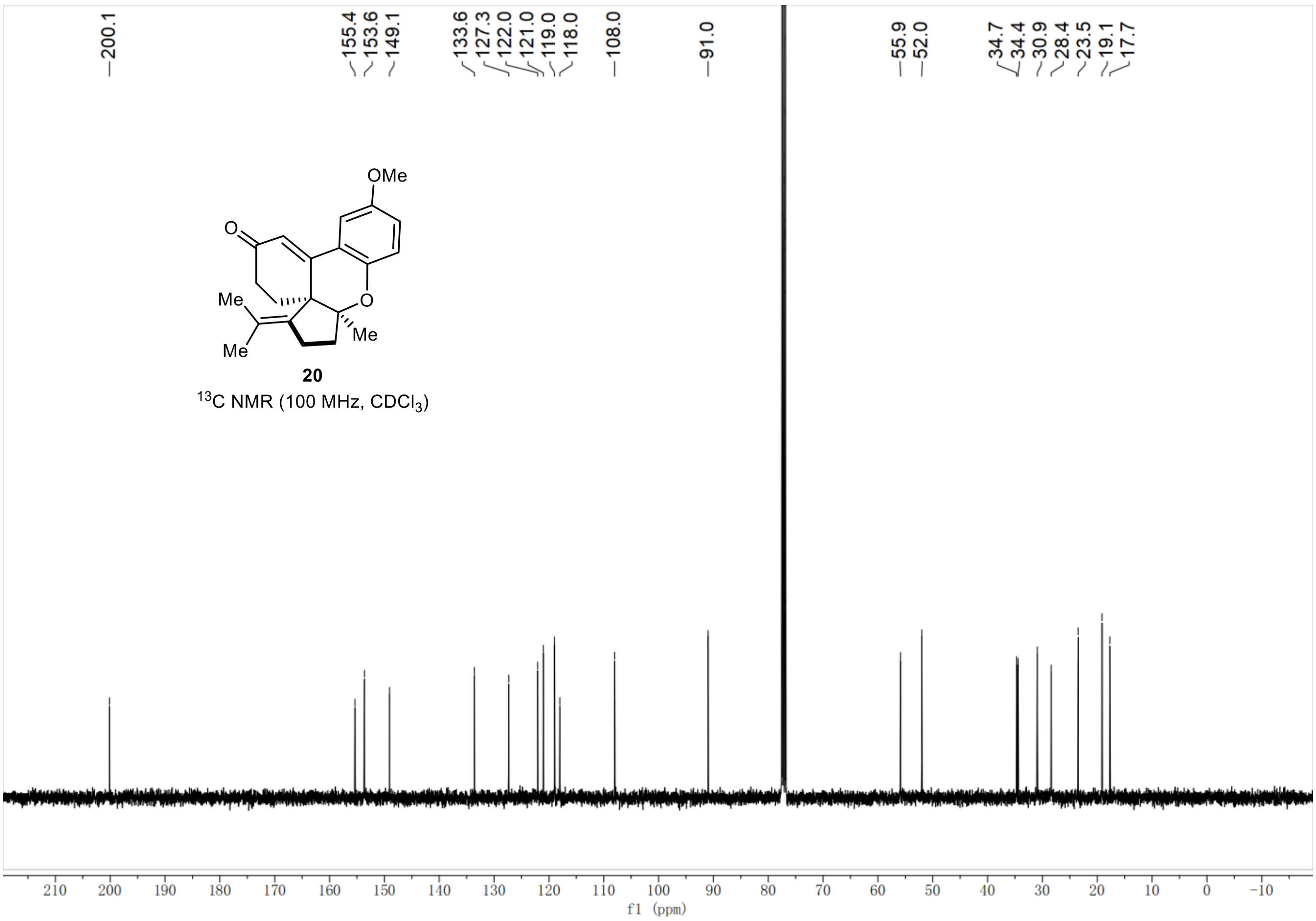

200.1
155.4
153.6
149.1
133.6
127.3
122.0
121.0
119.0
118.0
108.0
91.0
55.9
52.0
34.7
34.4
30.9
28.4
23.5
19.1
17.7
OMe
O
Me
O
Me
Me
20
^{13}C NMR (100 MHz, $CDCl_3$)
f1 (ppm)

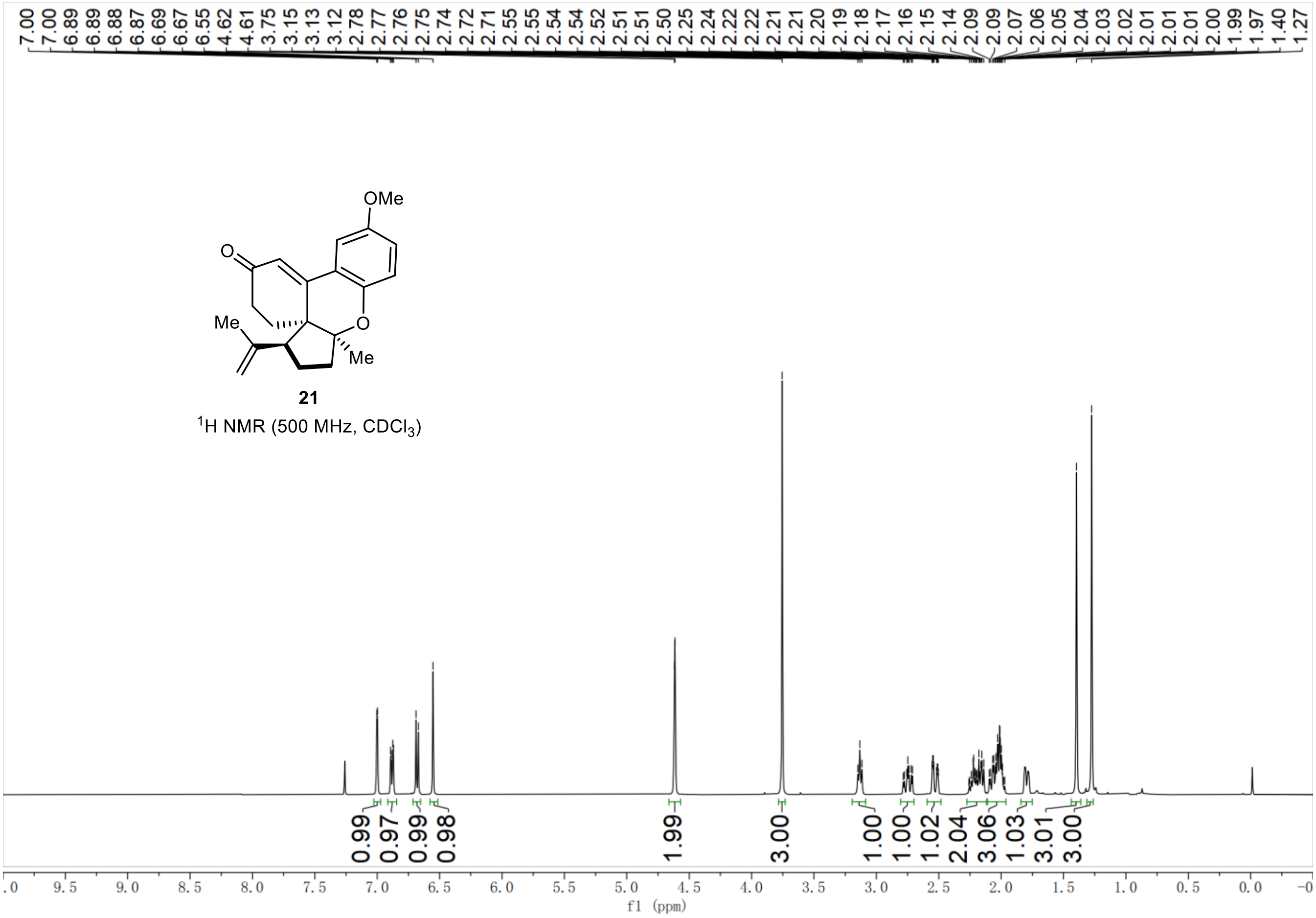
7.00 7.00 6.89 6.89 6.88 6.87 6.69 6.67 6.55 4.62 4.61 3.75 3.15 3.13 3.12 2.78 2.77 2.76 2.75 2.74 2.72 2.71 2.55 2.55 2.54 2.54 2.52 2.51 2.51 2.50 2.25 2.24 2.22 2.22 2.21 2.21 2.20 2.19 2.18 2.17 2.16 2.15 2.14 2.09 2.09 2.07 2.06 2.05 2.04 2.03 2.02 2.01 2.01 2.00 1.99 1.97 1.40 1.27
OMe
O
Me
Me
21
^{1}H NMR (500 MHz, CDCl$_3$)
0.99 0.97 0.99 0.98 1.99 3.00 1.00 1.00 1.02 2.04 3.06 1.03 3.01 3.00
f1 (ppm)

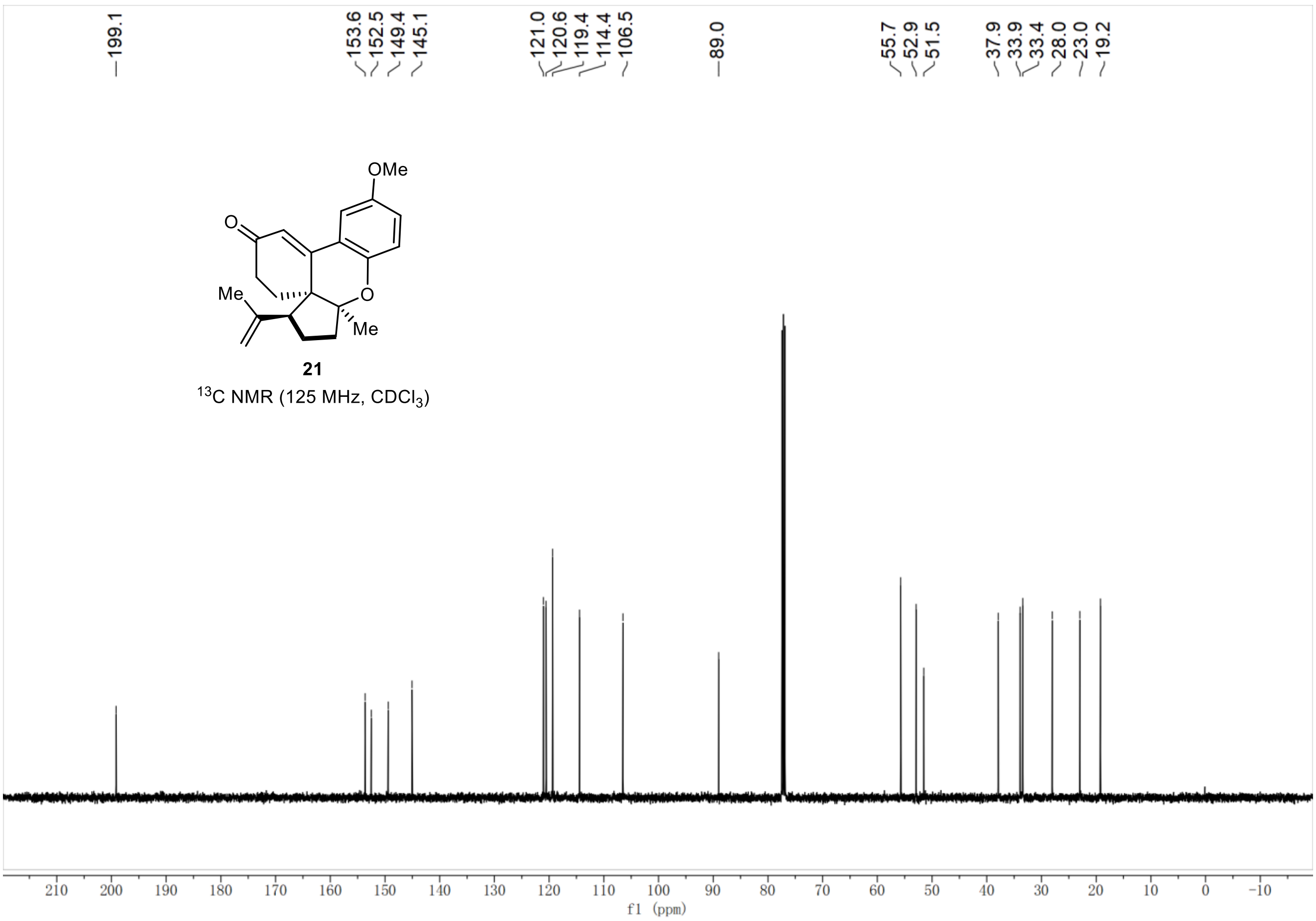
199.1
153.6 152.5 149.4 145.1
121.0 120.6 119.4 114.4 106.5
89.0
55.7 52.9 51.5
37.9 33.9 33.4 28.0 23.0 19.2
OMe
O
Me
Me
21
^{13}C NMR (125 MHz, CDCl$_3$)
f1 (ppm)

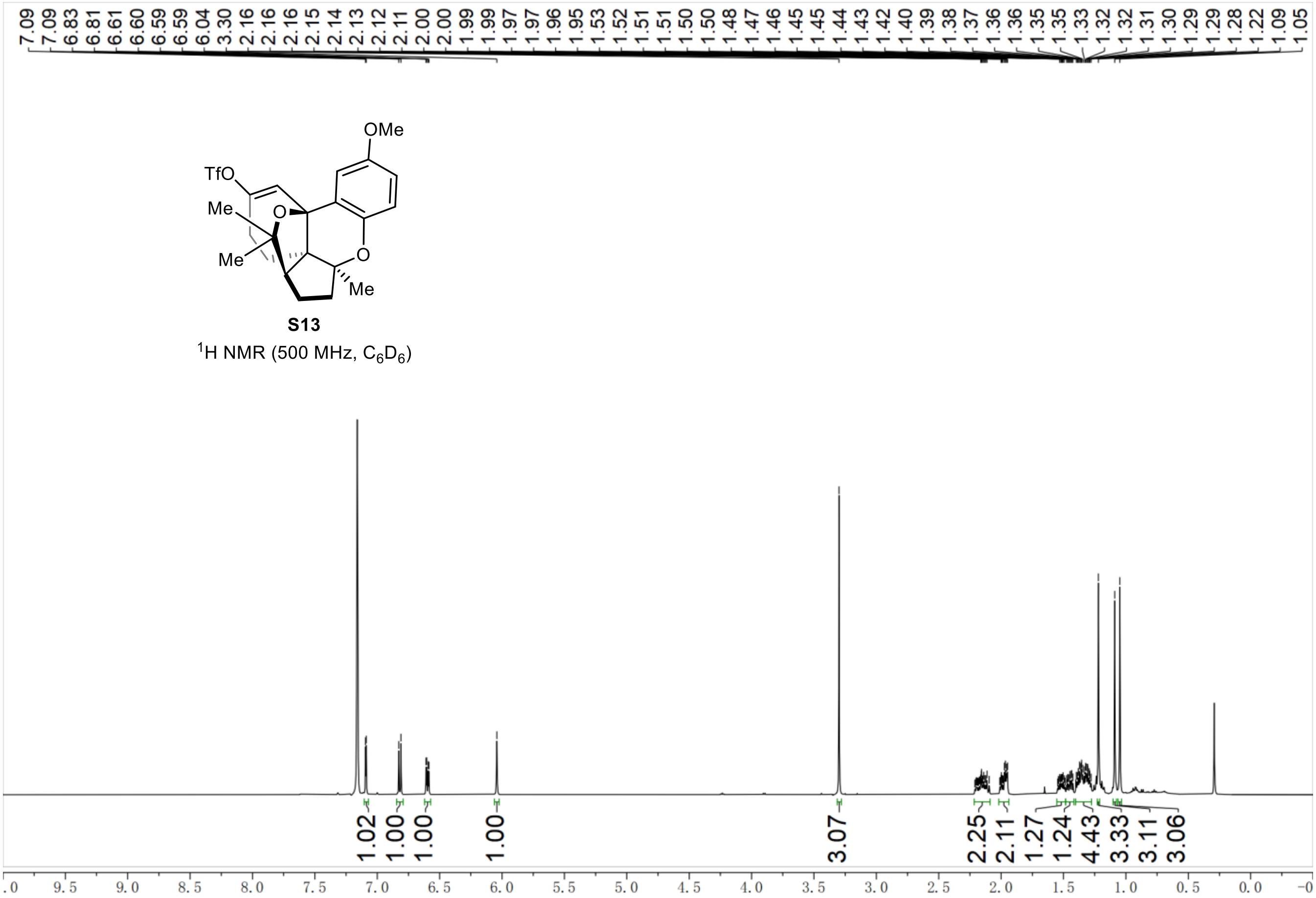
7.09 7.09 6.83 6.81 6.61 6.60 6.59 6.59 6.04 3.30 2.16 2.16 2.16 2.15 2.14 2.13 2.12 2.11 2.00 2.00 1.99 1.99 1.97 1.97 1.96 1.95 1.53 1.52 1.51 1.51 1.50 1.48 1.47 1.46 1.45 1.45 1.44 1.43 1.42 1.40 1.39 1.38 1.37 1.36 1.36 1.35 1.35 1.33 1.32 1.32 1.31 1.30 1.29 1.28 1.22 1.09 1.05
OMe
TfO
Me
O
Me
O
Me
S13
1H NMR (500 MHz, C6D6)
1.02 1.00 1.00 1.00 3.07 2.25 2.11 1.27 1.24 4.43 3.33 3.11 3.06
f1 (ppm)

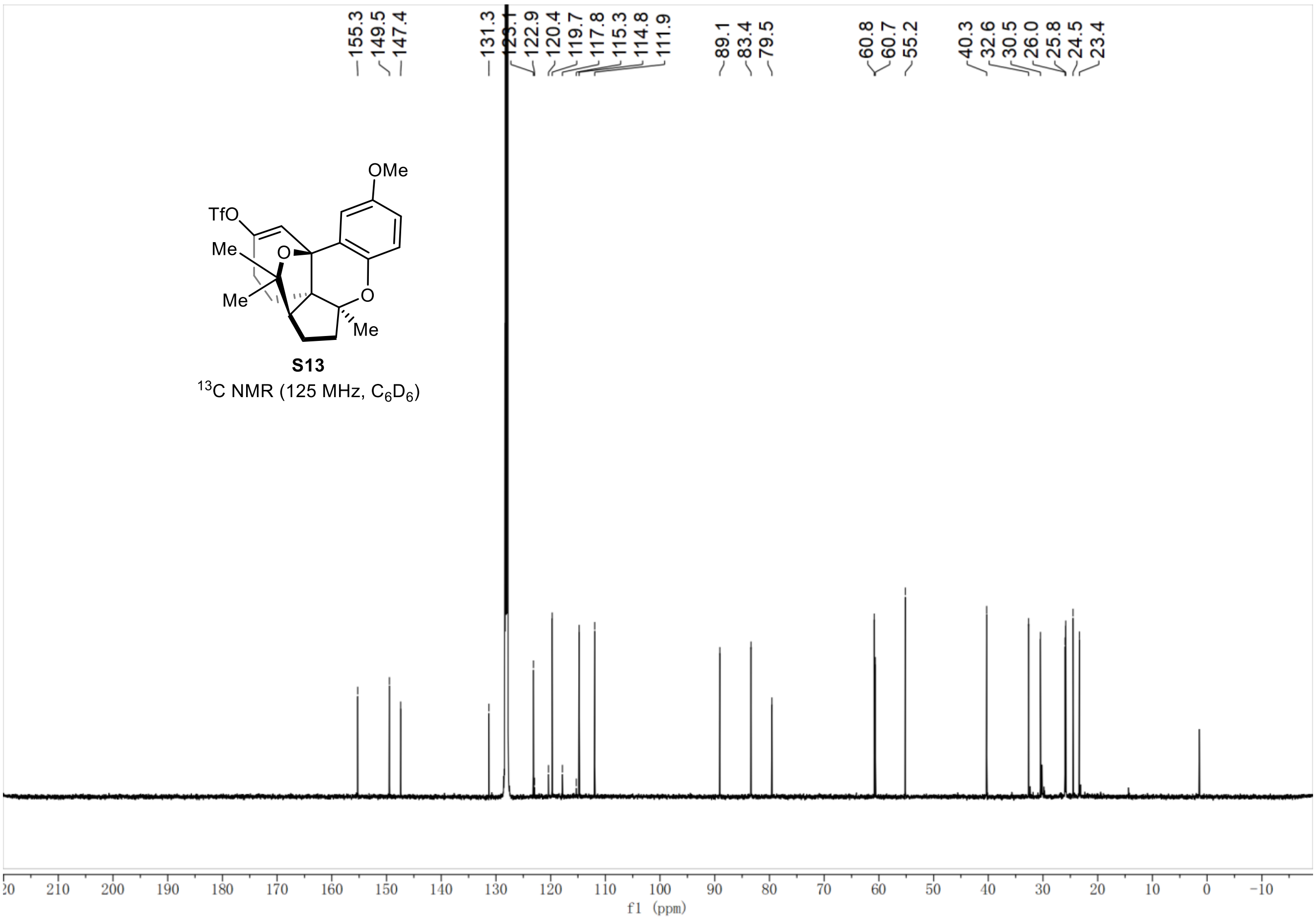
155.3 149.5 147.4 131.3 122.9 120.4 119.7 117.8 115.3 114.8 111.9 89.1 83.4 79.5 60.8 60.7 55.2 40.3 32.6 30.5 26.0 25.8 24.5 23.4
OMe
TfO
Me
O
Me
O
Me
S13
13C NMR (125 MHz, C6D6)
f1 (ppm)

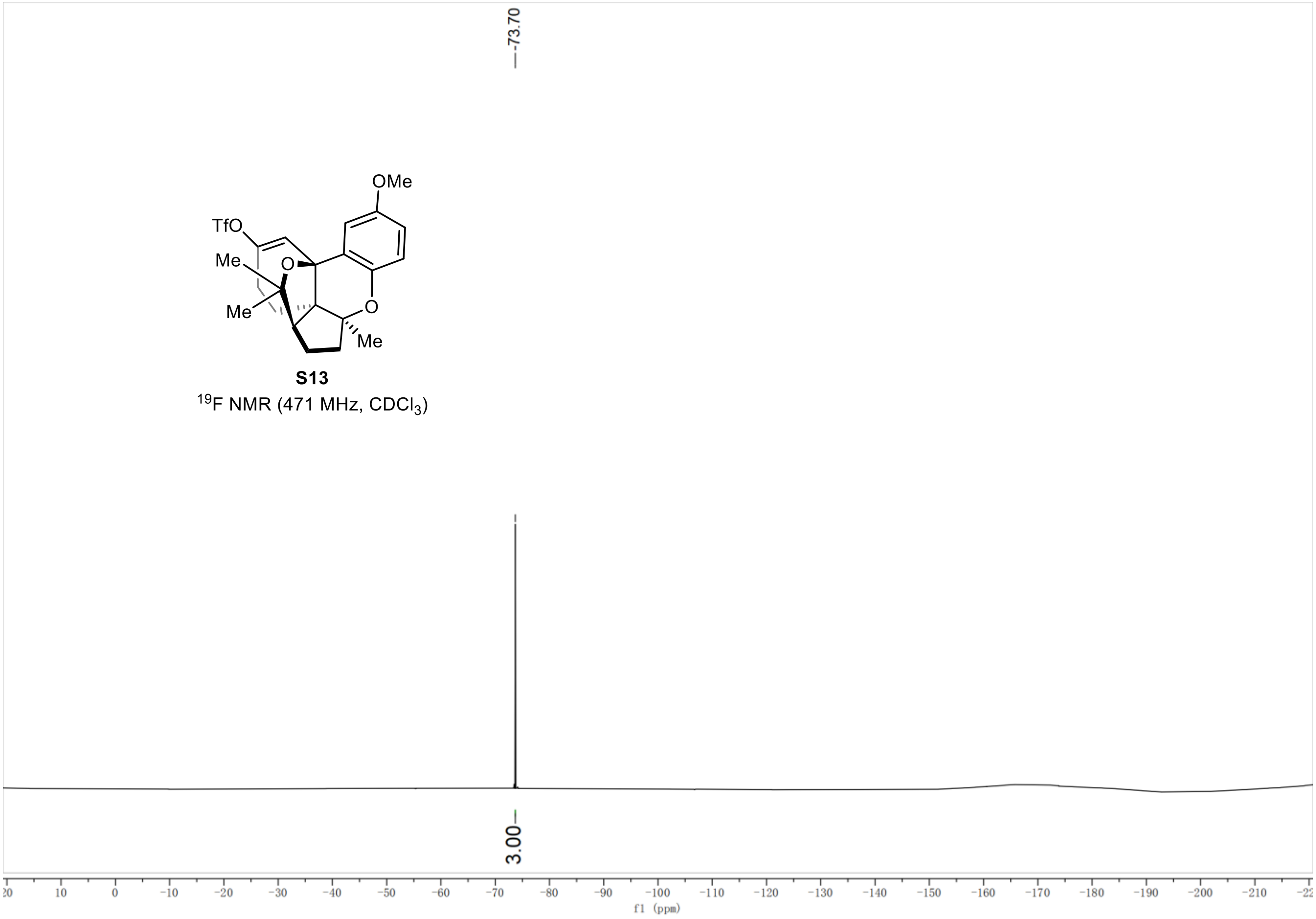

-73.70
OMe
TfO
Me
O
Me
O
Me
S13
19F NMR (471 MHz, CDCl3)
3.00
f1 (ppm)

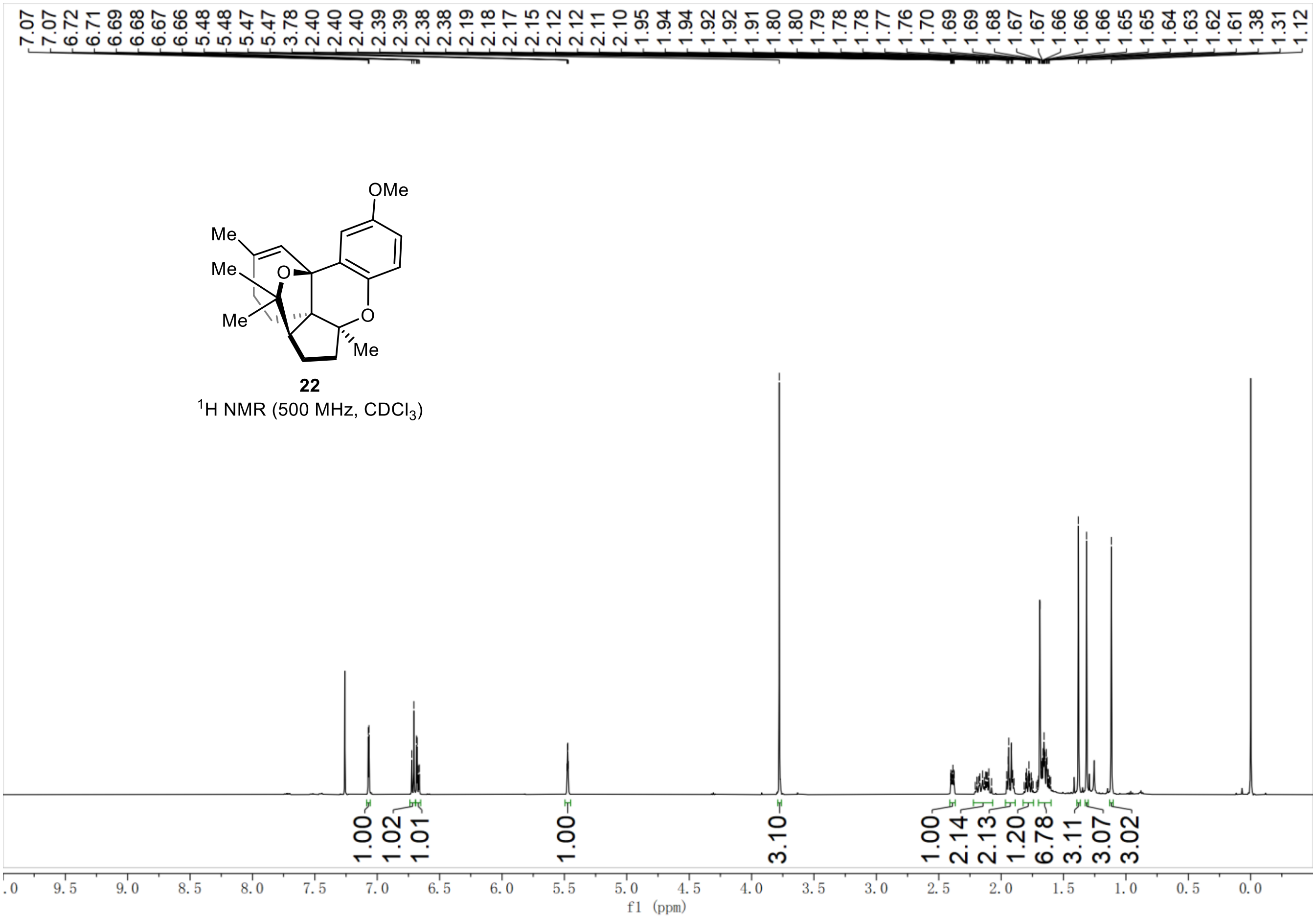

7.07 7.07 6.72 6.71 6.69 6.68 6.67 6.66 5.48 5.48 5.47 5.47 3.78 2.40 2.40 2.40 2.39 2.39 2.38 2.38 2.19 2.18 2.17 2.15 2.12 2.12 2.11 2.10 1.95 1.94 1.94 1.92 1.92 1.91 1.80 1.80 1.79 1.78 1.78 1.77 1.76 1.70 1.69 1.68 1.67 1.67 1.66 1.66 1.66 1.65 1.65 1.64 1.63 1.62 1.61 1.38 1.31 1.12
OMe
Me
Me
O
O
Me
Me
22
1H NMR (500 MHz, CDCl3)
1.00 1.02 1.01 1.00 3.10 1.00 2.14 2.13 1.20 6.78 3.11 3.07 3.02
f1 (ppm)

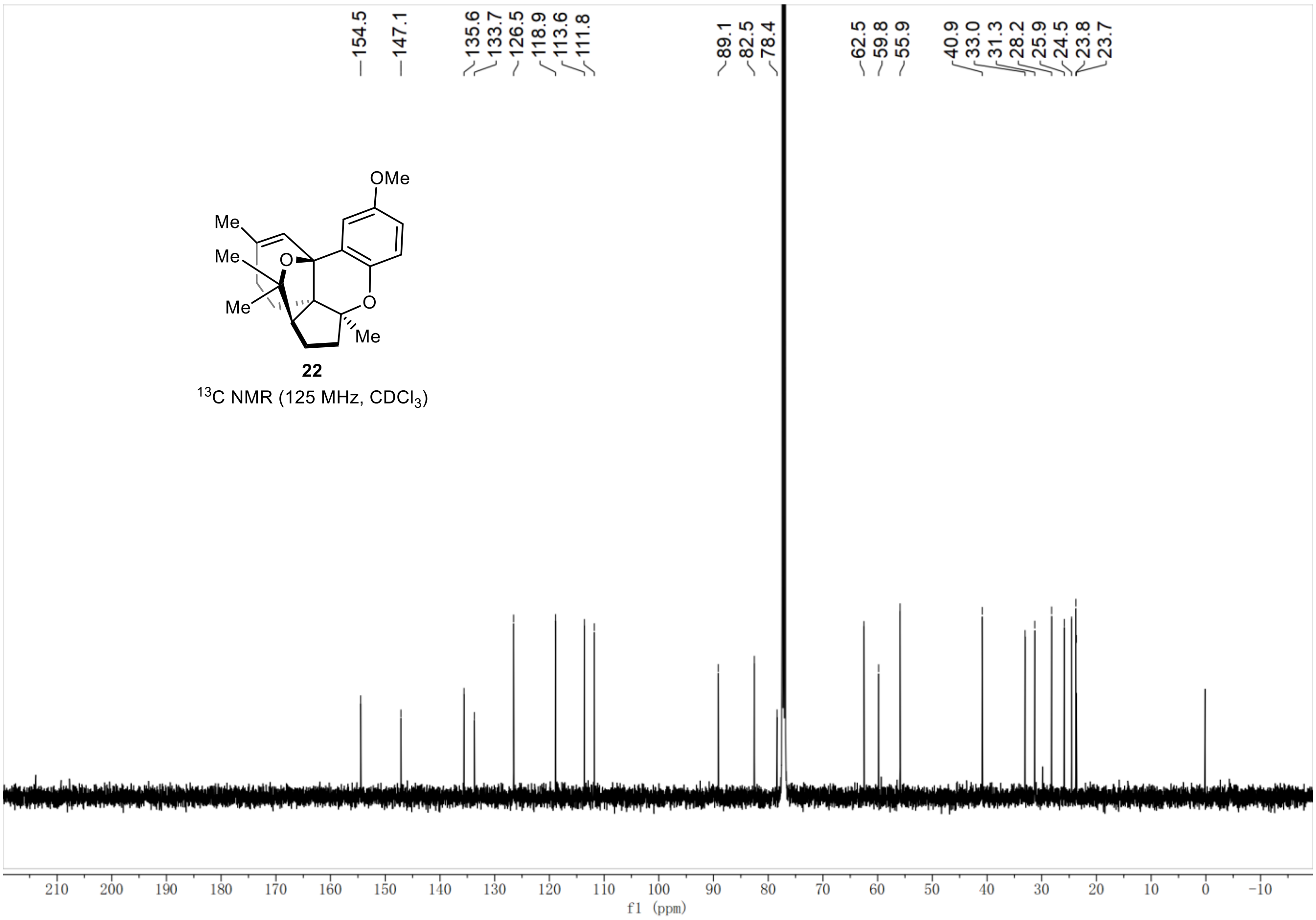
154.5
147.1
135.6
133.7
126.5
118.9
113.6
111.8
89.1
82.5
78.4
62.5
59.8
55.9
40.9
33.0
31.3
28.2
25.9
24.5
23.8
23.7
OMe
Me
Me
O
Me
O
Me
22
13C NMR (125 MHz, CDCl3)
f1 (ppm)

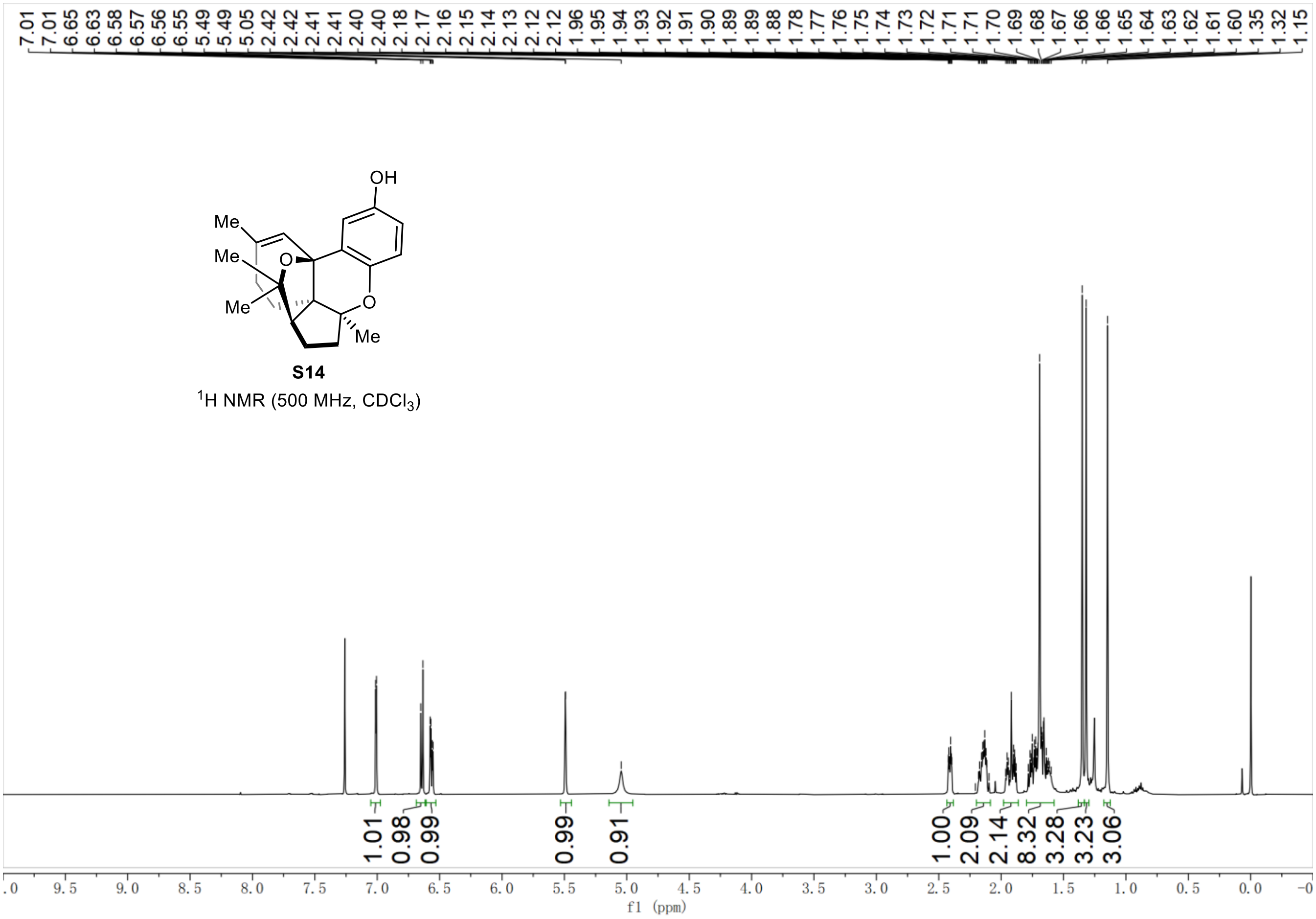
7.01
7.01
6.65
6.63
6.58
6.57
6.56
6.55
5.49
5.05
2.42
2.42
2.41
2.41
2.40
2.40
2.18
2.17
2.16
2.15
2.14
2.13
2.12
2.12
1.96
1.95
1.94
1.93
1.92
1.91
1.90
1.89
1.89
1.88
1.78
1.76
1.75
1.74
1.73
1.72
1.71
1.71
1.70
1.69
1.68
1.67
1.66
1.66
1.65
1.64
1.63
1.62
1.61
1.60
1.35
1.32
1.15
OH
Me
Me
O
Me
O
Me
S14
1H NMR (500 MHz, CDCl3)
1.01
0.98
0.99
0.99
0.91
1.00
2.09
2.14
8.32
3.28
3.23
3.06
f1 (ppm)

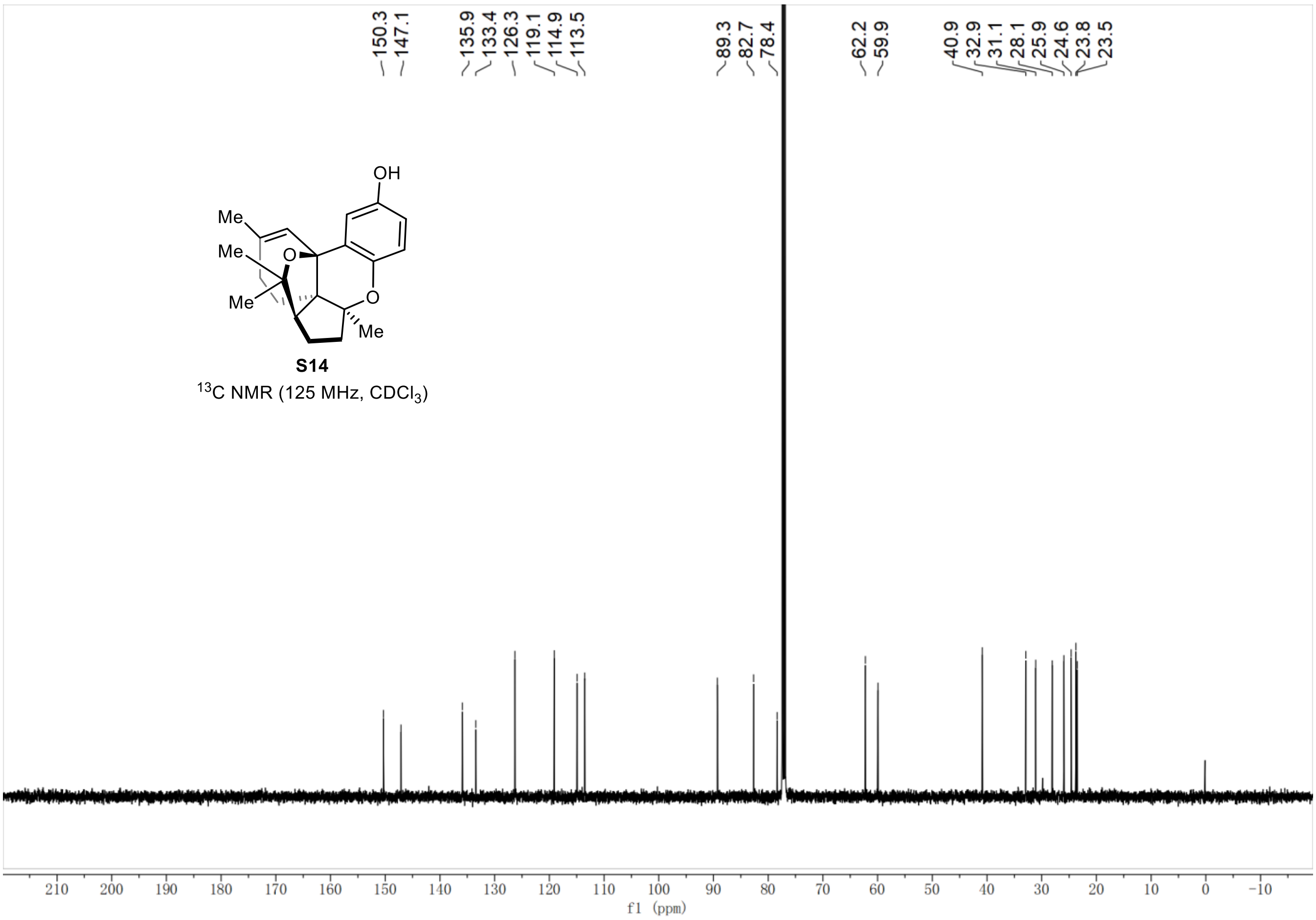
150.3
147.1
135.9
133.4
126.3
119.1
114.9
113.5
89.3
82.7
78.4
62.2
59.9
40.9
32.9
31.1
28.1
25.9
24.6
23.8
23.5
OH
Me
Me
O
Me
O
Me
S14
^{13}C NMR (125 MHz, $CDCl_3$)
210 200 190 180 170 160 150 140 130 120 110 100 90 80 70 60 50 40 30 20 10 0 -10
f1 (ppm)

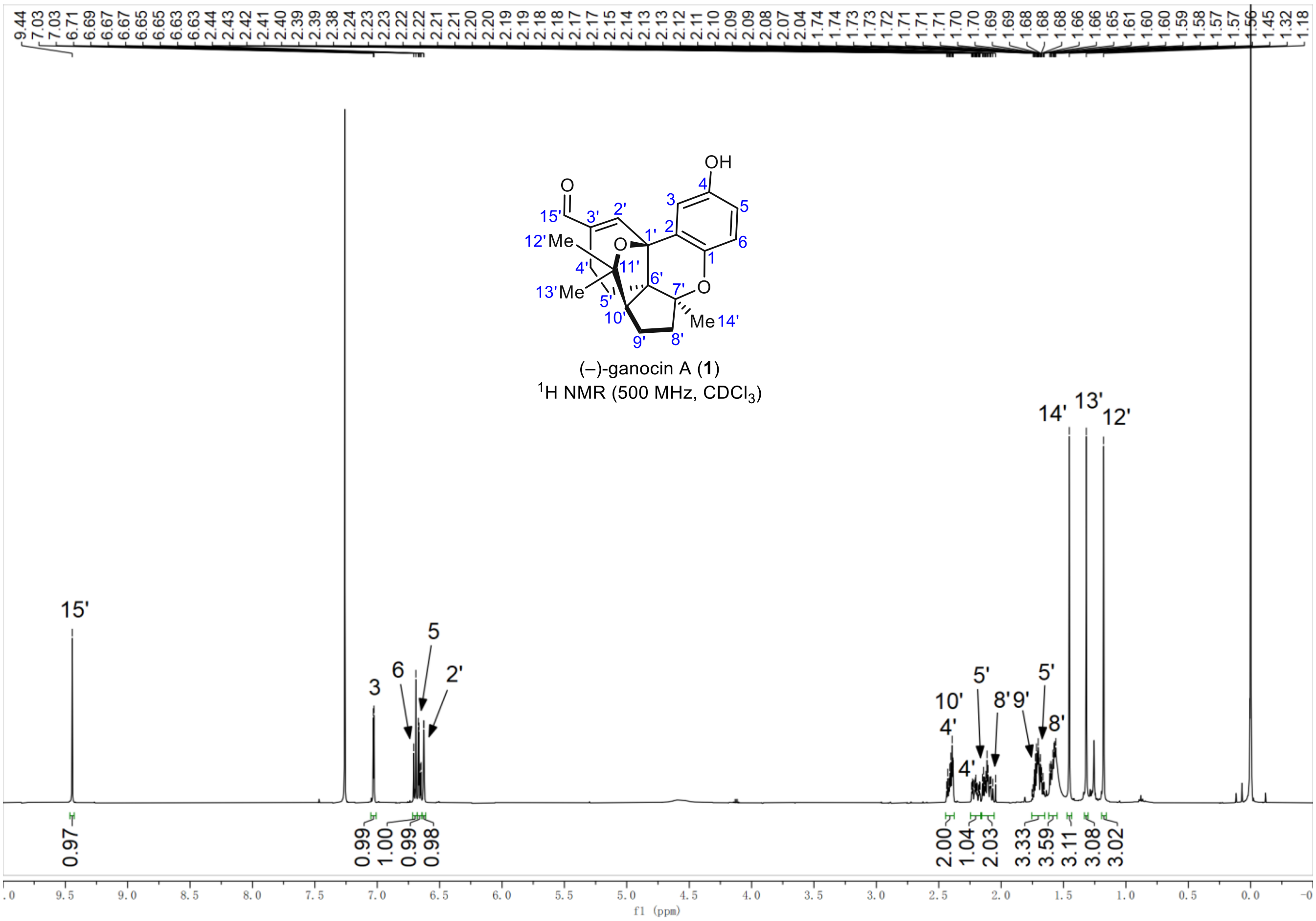
9.44 7.03 7.03 6.71 6.69 6.67 6.67 6.65 6.65 6.63 6.63 2.44 2.43 2.42 2.41 2.40 2.39 2.39 2.38 2.24 2.23 2.23 2.22 2.22 2.21 2.21 2.20 2.20 2.19 2.19 2.18 2.18 2.17 2.17 2.15 2.14 2.13 2.13 2.12 2.11 2.10 2.09 2.09 2.08 2.07 2.04 1.74 1.74 1.73 1.73 1.72 1.71 1.71 1.71 1.70 1.70 1.69 1.69 1.68 1.68 1.68 1.66 1.66 1.65 1.61 1.60 1.60 1.59 1.58 1.57 1.57 1.56 1.45 1.32 1.18
OH
(–)-ganocin A (**1**)
^{1}H NMR (500 MHz, $CDCl_3$)
15'
3
6
5
2'
10'
4'
4'
5'
8'
9'
5'
8'
14'
13'
12'
0.97
0.99
1.00
0.99
0.98
2.00
1.04
2.03
3.33
3.59
3.11
3.08
3.02
f1 (ppm)

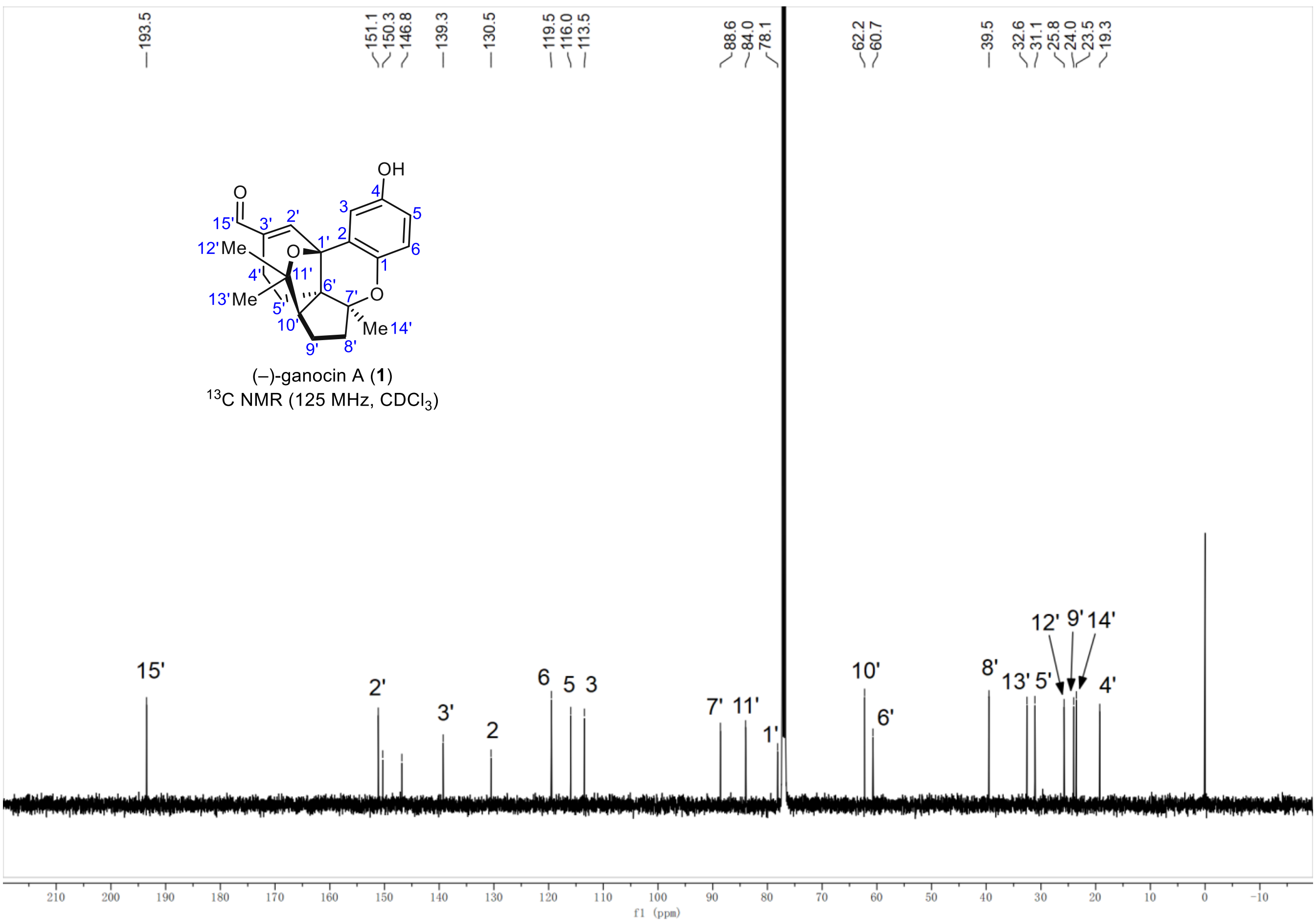
193.5
151.1
150.3
146.8
139.3
130.5
119.5
116.0
113.5
88.6
84.0
78.1
62.2
60.7
39.5
32.6
31.1
25.8
24.0
23.5
19.3
(–)-ganocin A (1)
13C NMR (125 MHz, CDCl3)
15' 2' 3' 2 6 5 3 7' 11' 1' 10' 6' 8' 13' 5' 12' 9' 14' 4'
f1 (ppm)

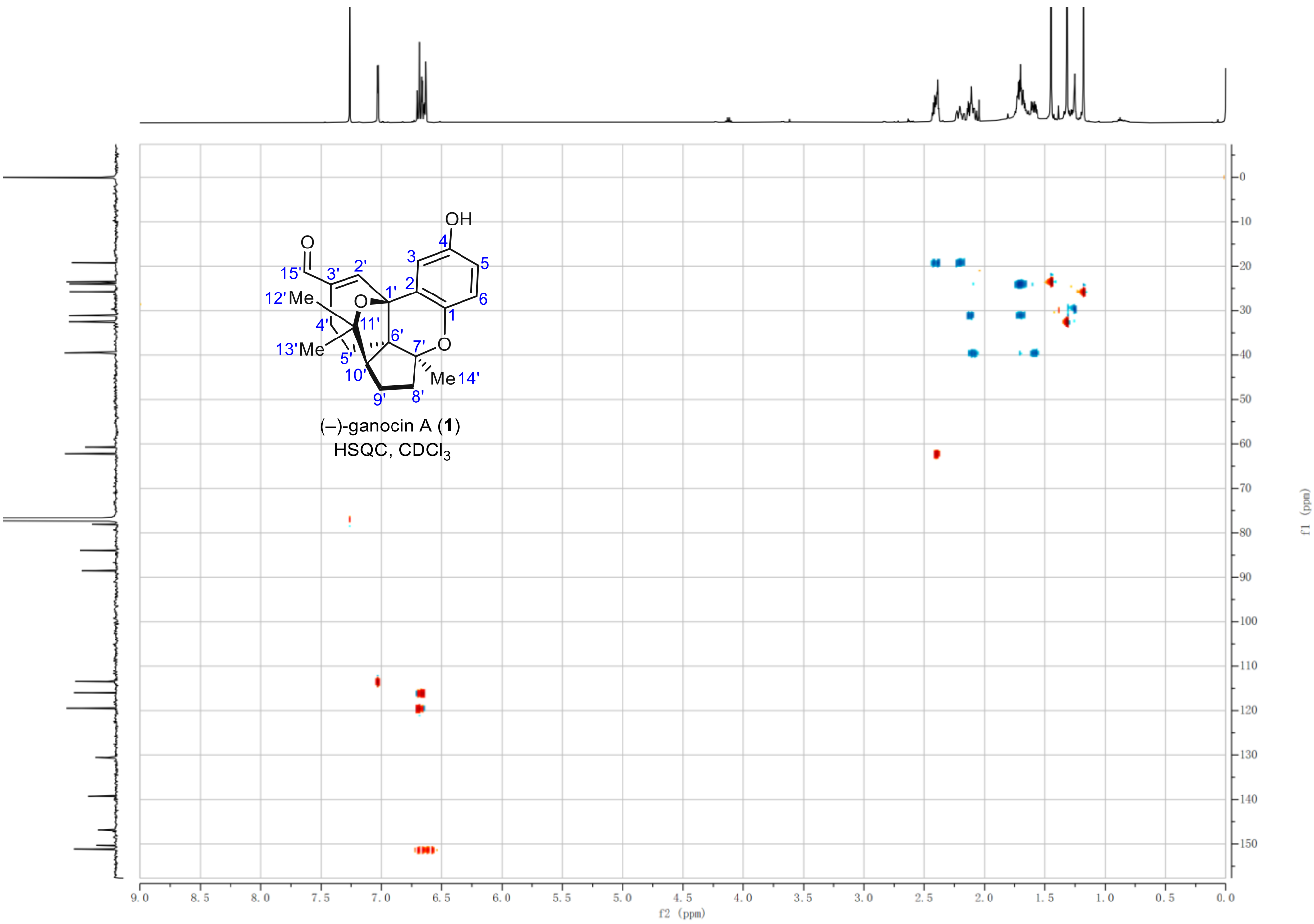
(–)-ganocin A (1)
HSQC, CDCl3
f2 (ppm)
f1 (ppm)

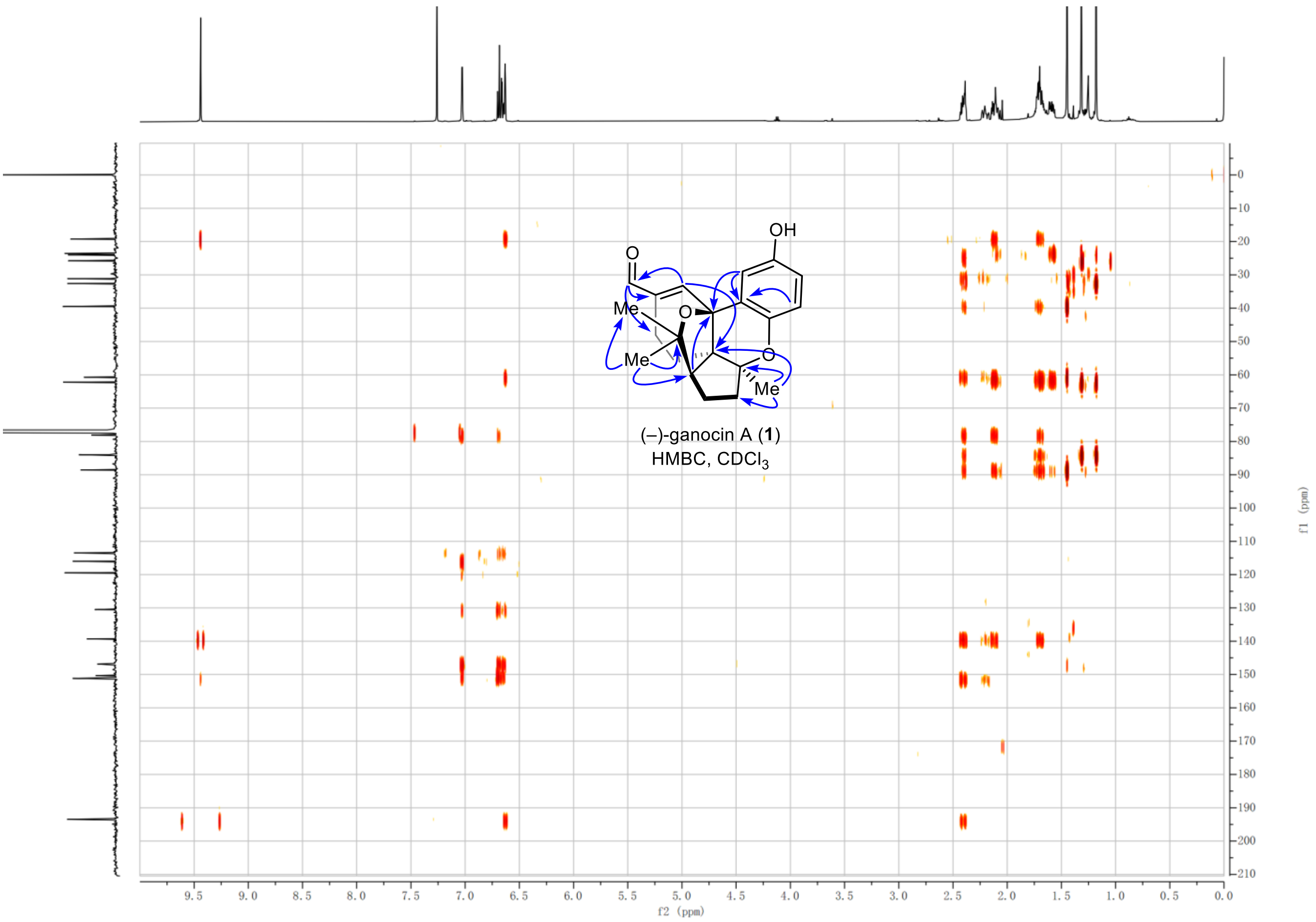
(–)-ganocin A (1)
HMBC, CDCl3
f2 (ppm)
f1 (ppm)

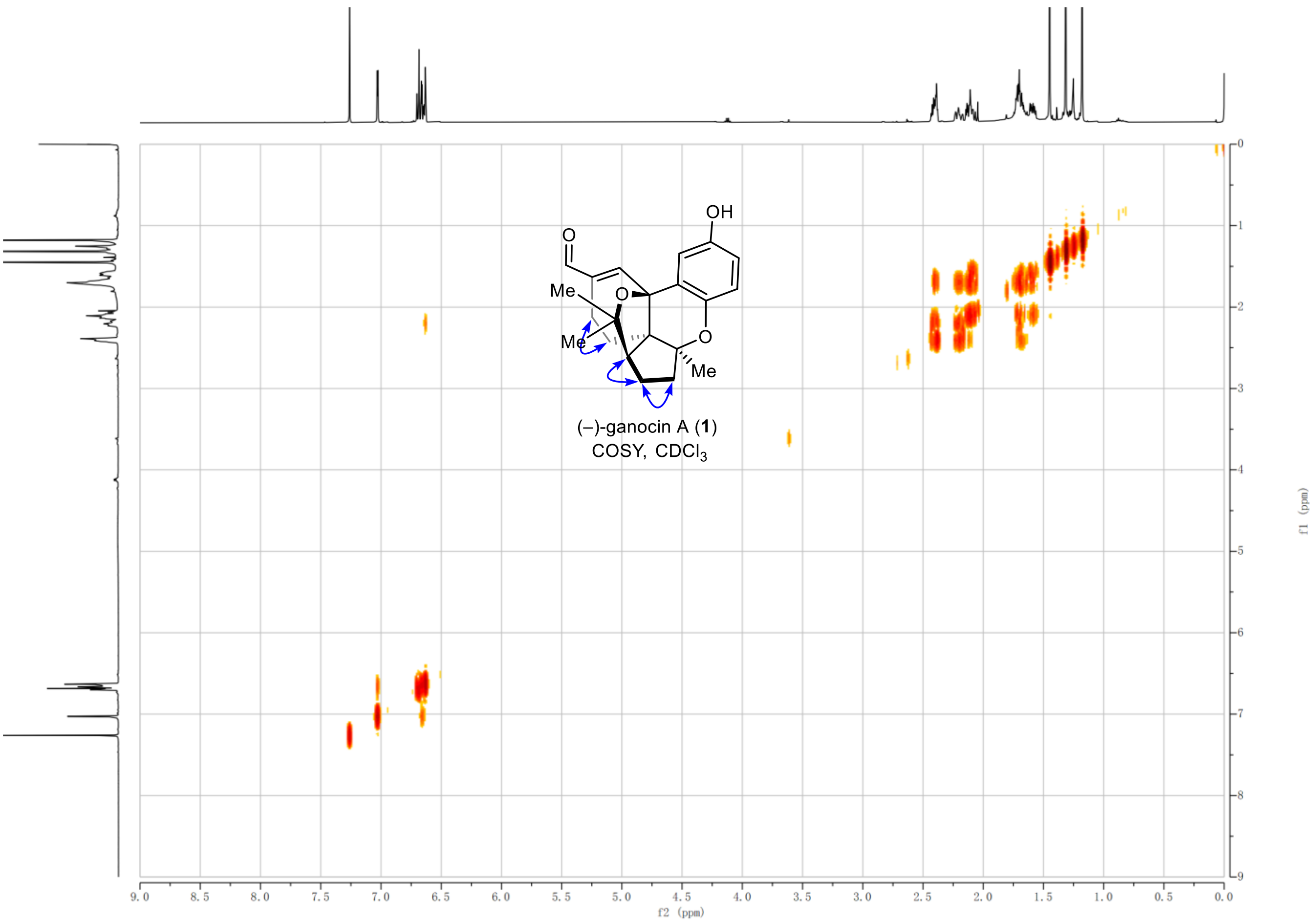
(–)-ganocin A (1)
COSY, CDCl3
f2 (ppm)
f1 (ppm)

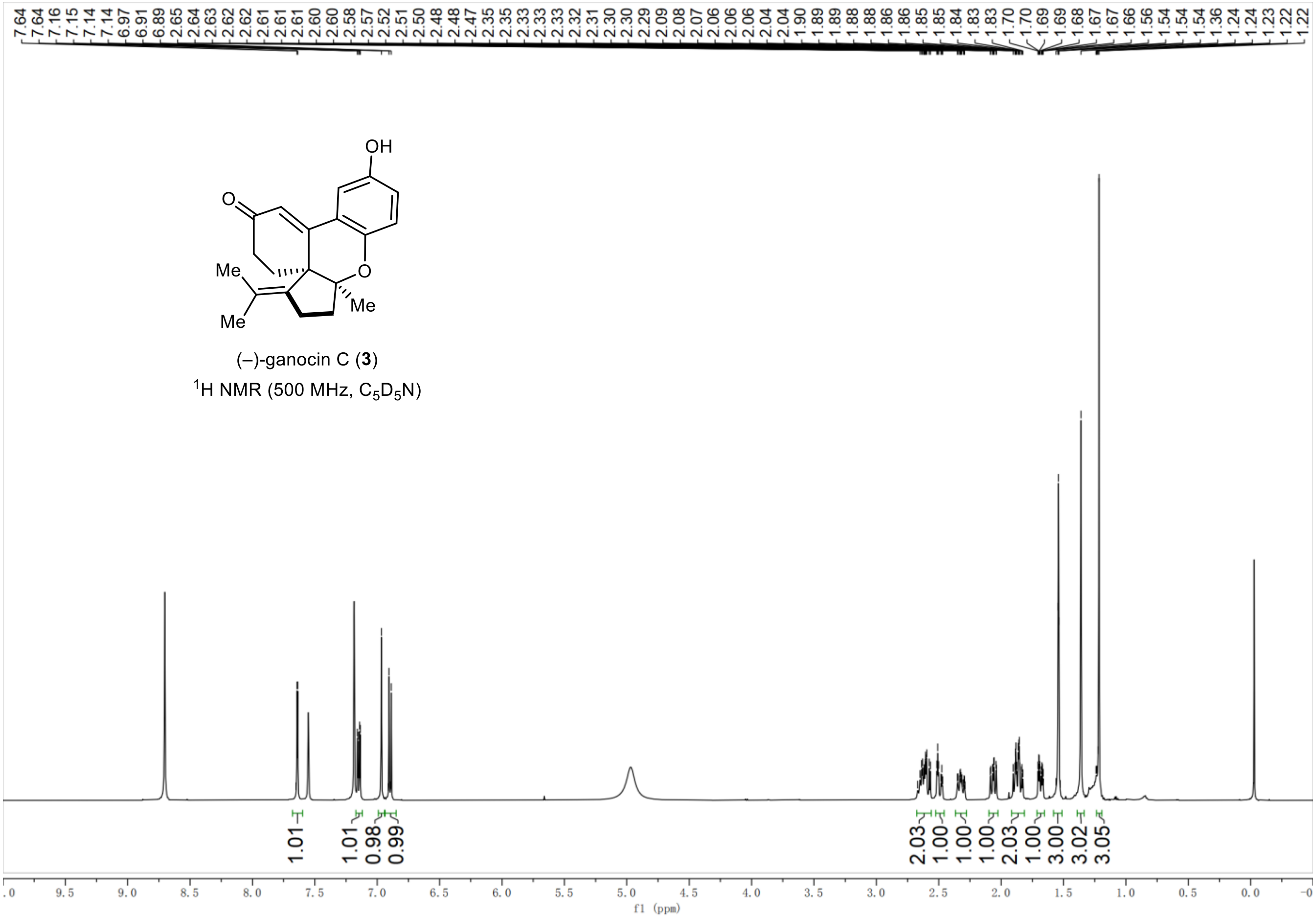
7.64 7.64 7.16 7.15 7.14 7.14 6.97 6.91 6.89 2.65 2.64 2.63 2.62 2.62 2.61 2.61 2.61 2.60 2.60 2.58 2.57 2.52 2.51 2.50 2.48 2.48 2.47 2.35 2.35 2.33 2.33 2.33 2.32 2.31 2.30 2.30 2.29 2.09 2.08 2.07 2.06 2.06 2.06 2.04 2.04 1.90 1.89 1.89 1.88 1.88 1.86 1.86 1.85 1.85 1.84 1.83 1.83 1.70 1.69 1.69 1.68 1.67 1.67 1.66 1.56 1.54 1.54 1.54 1.36 1.24 1.24 1.23 1.22 1.22
OH
O
Me
O
Me
Me
(–)-ganocin C (3)
1H NMR (500 MHz, C5D5N)
1.01
1.01
0.98
0.99
2.03
1.00
1.00
1.00
2.03
1.00
3.00
3.02
3.05
f1 (ppm)

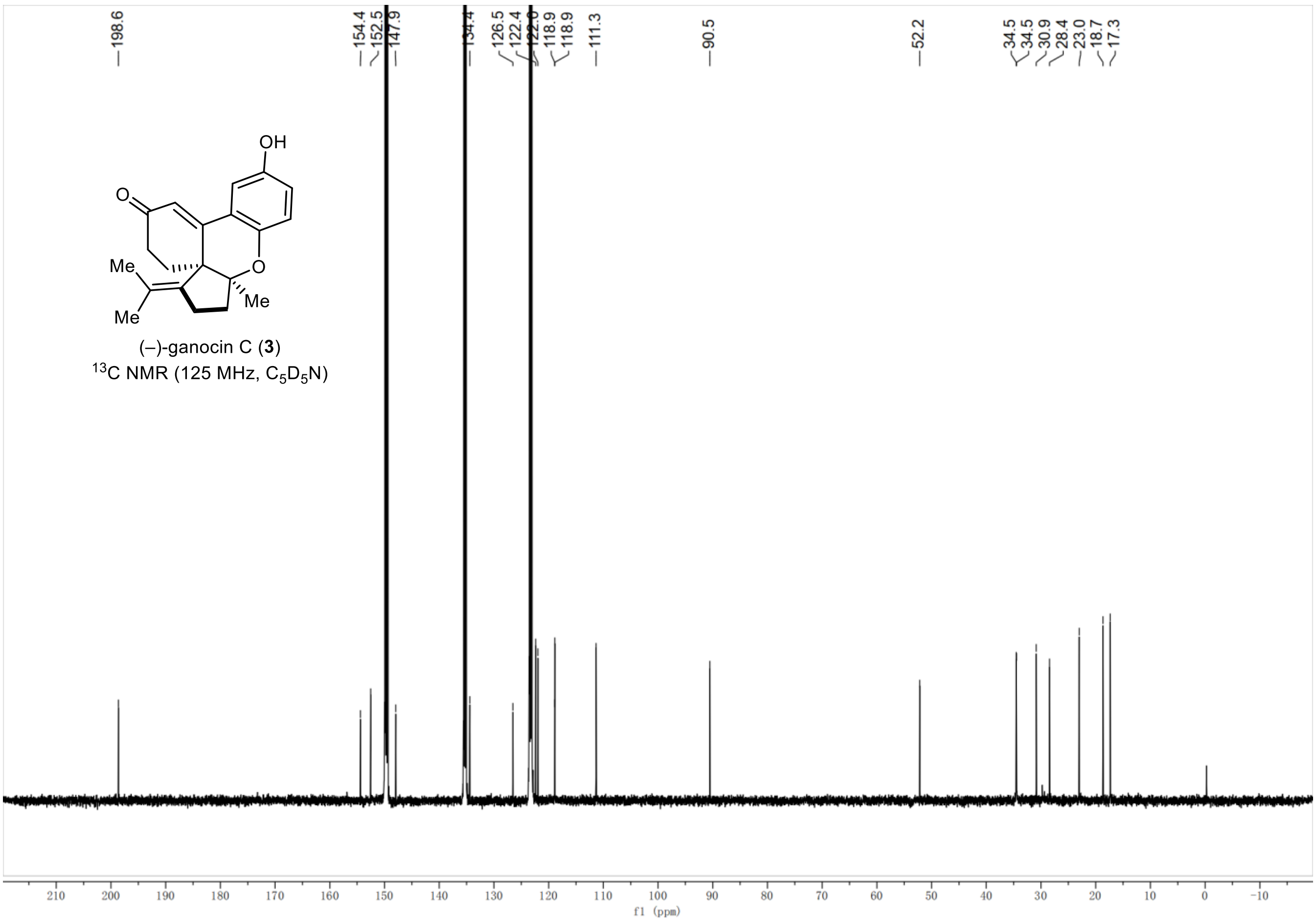
198.6
154.4
152.5
147.9
134.4
126.5
122.4
122.0
118.9
118.9
111.3
90.5
52.2
34.5
34.5
30.9
28.4
23.0
18.7
17.3
OH
O
Me
O
Me
Me
(–)-ganocin C (3)
13C NMR (125 MHz, C5D5N)
f1 (ppm)

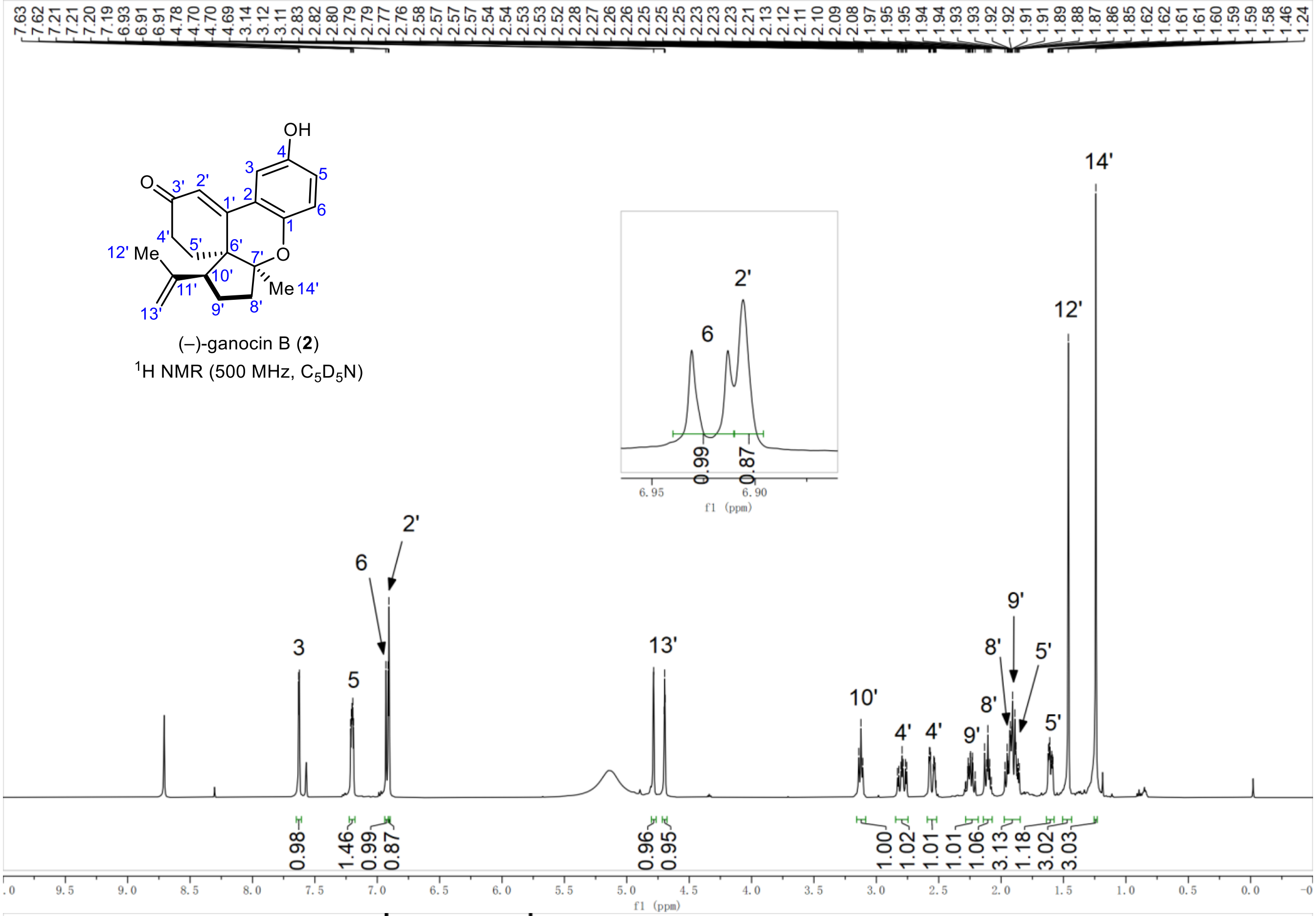
7.63 7.62 7.21 7.21 7.20 7.19 6.93 6.91 6.91 4.78 4.70 4.70 4.69 3.14 3.12 3.11 2.83 2.82 2.80 2.79 2.79 2.77 2.76 2.58 2.57 2.57 2.57 2.54 2.54 2.53 2.53 2.52 2.28 2.27 2.26 2.26 2.25 2.25 2.25 2.23 2.23 2.23 2.21 2.13 2.12 2.11 2.10 2.09 2.08 1.97 1.95 1.95 1.94 1.94 1.93 1.93 1.93 1.92 1.92 1.91 1.91 1.89 1.88 1.87 1.86 1.85 1.62 1.61 1.61 1.60 1.59 1.59 1.58 1.46 1.24
(–)-ganocin B (**2**)
^{1}H NMR (500 MHz, C_5D_5N)
f1 (ppm)

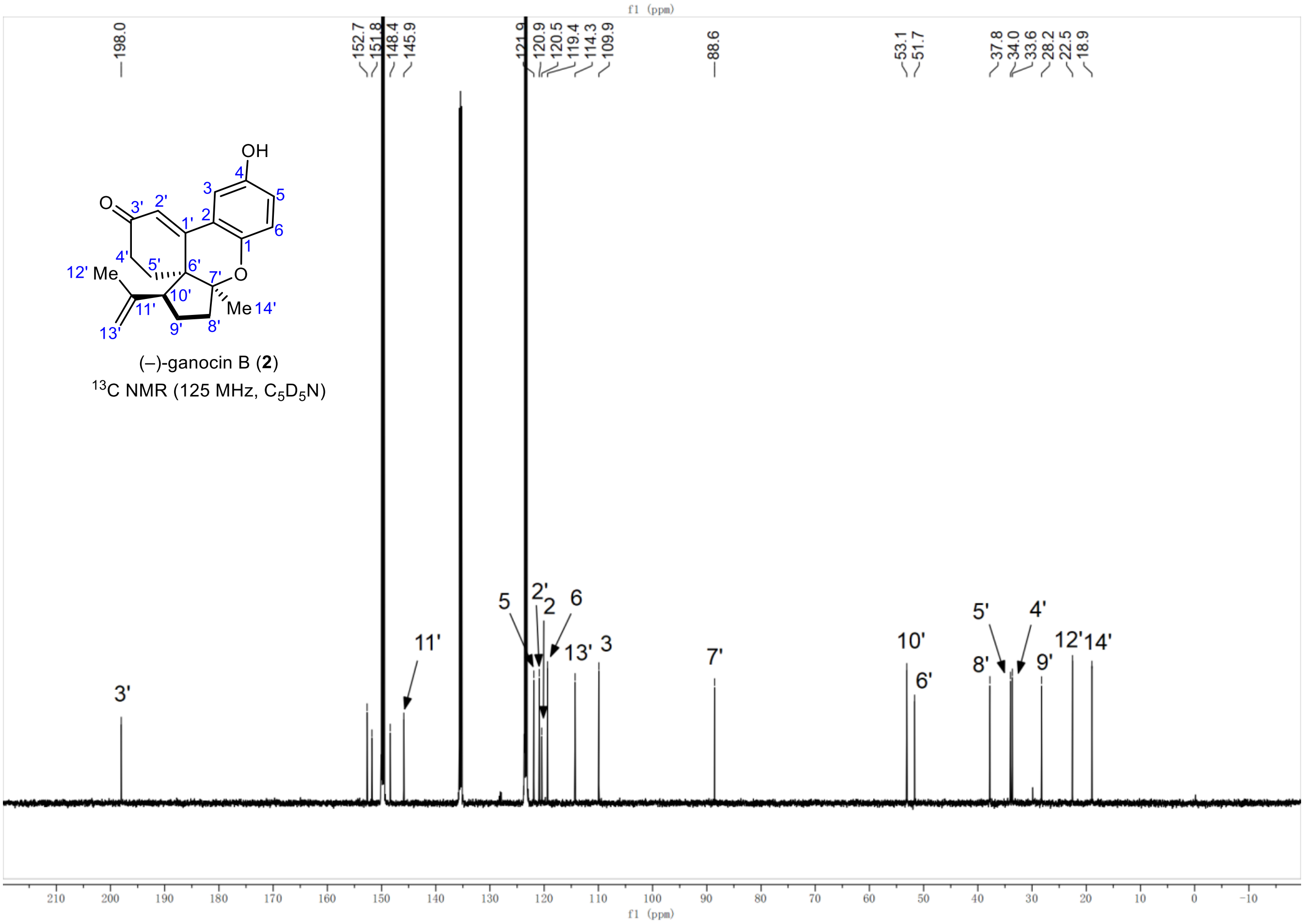
198.0
152.7 151.8 148.4 145.9
121.9 120.9 120.5 119.4 114.3 109.9
88.6
53.1 51.7
37.8 34.0 33.6 28.2 22.5 18.9
(–)-ganocin B (**2**)
^{13}C NMR (125 MHz, C_5D_5N)
f1 (ppm)

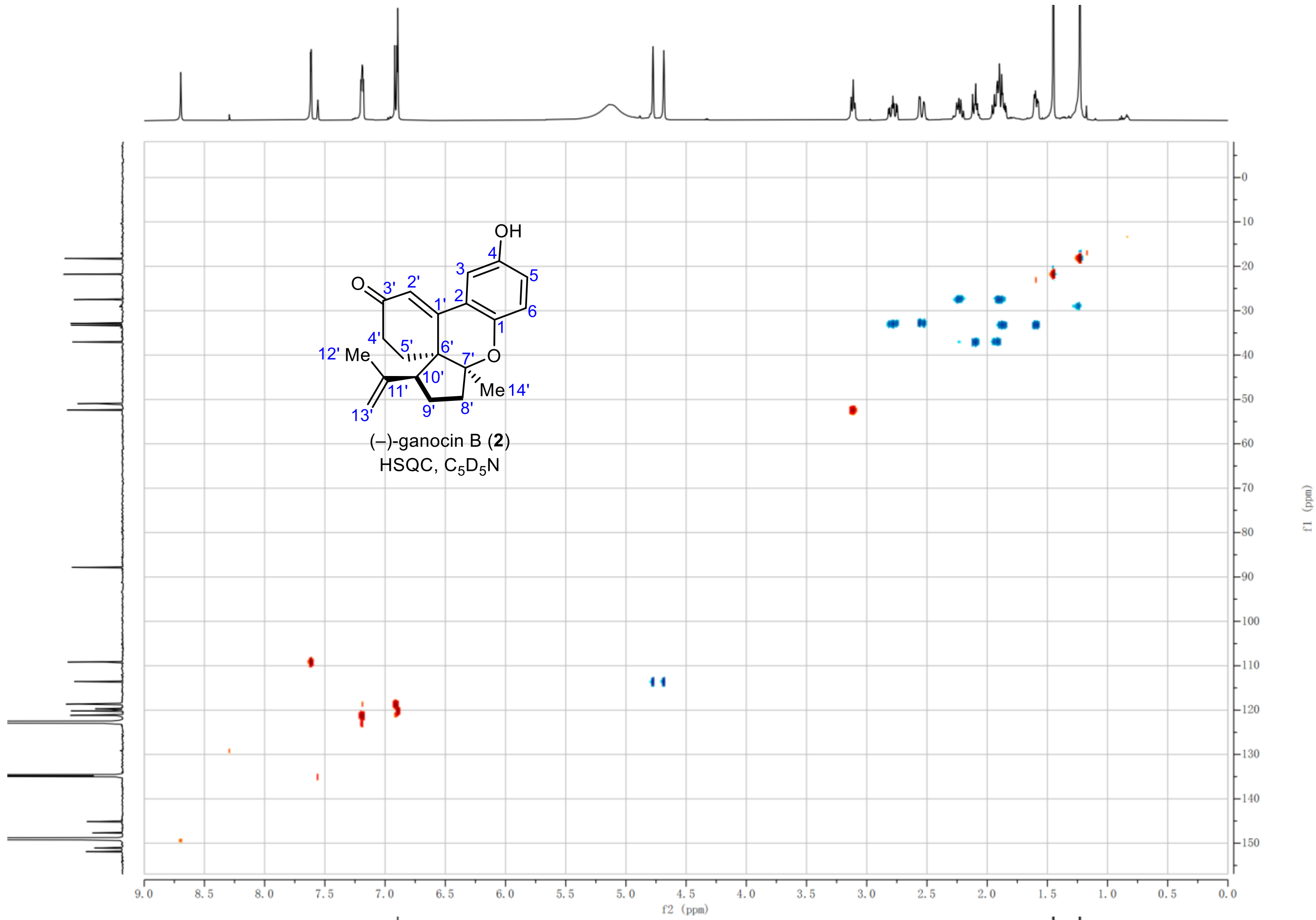

OH
(–)-ganocin B (2)
HSQC, C_5D_5N
f2 (ppm)
f1 (ppm)

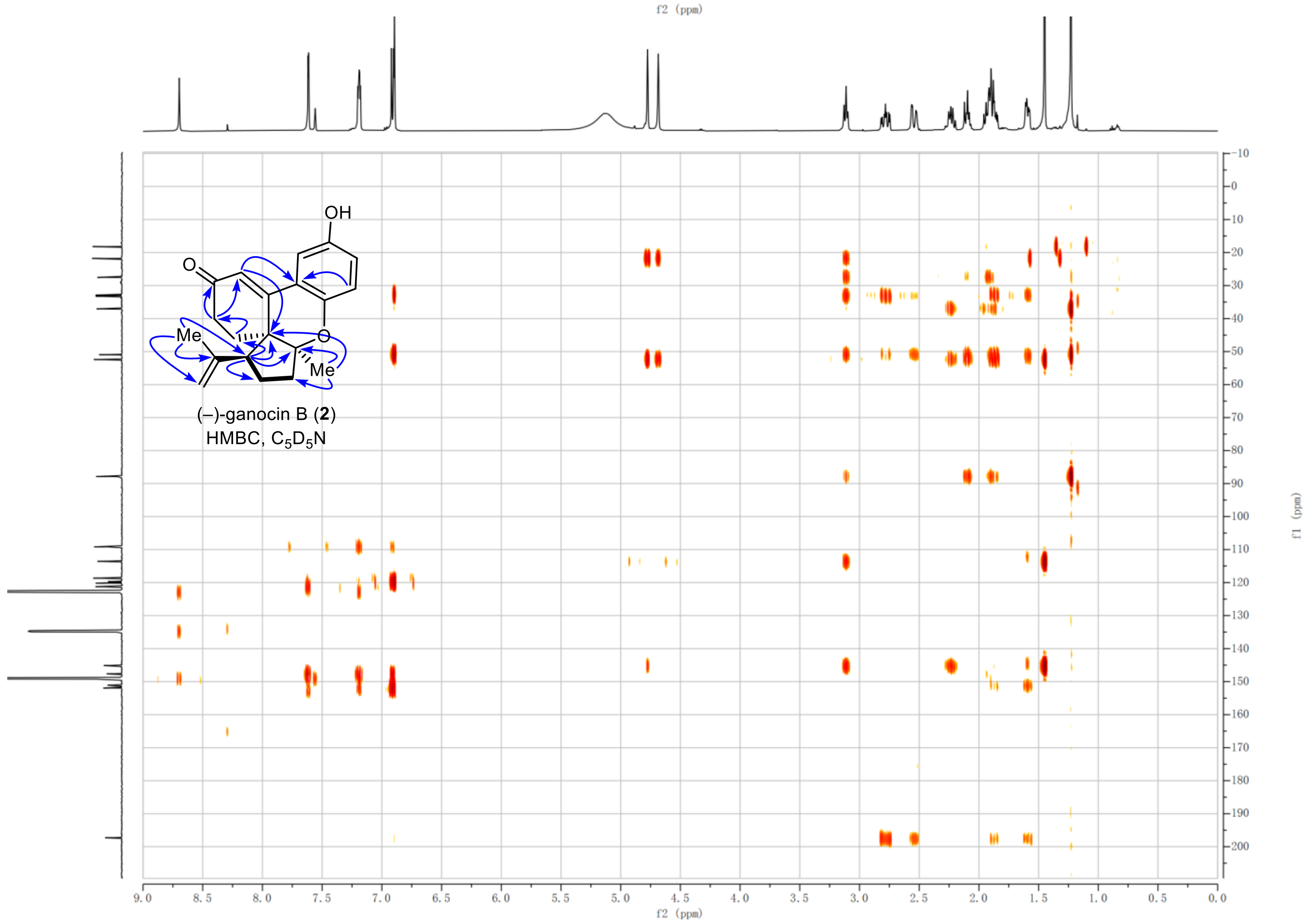

OH
O
Me
Me
(–)-ganocin B (2)
HMBC, C_5D_5N
f2 (ppm)
f1 (ppm)

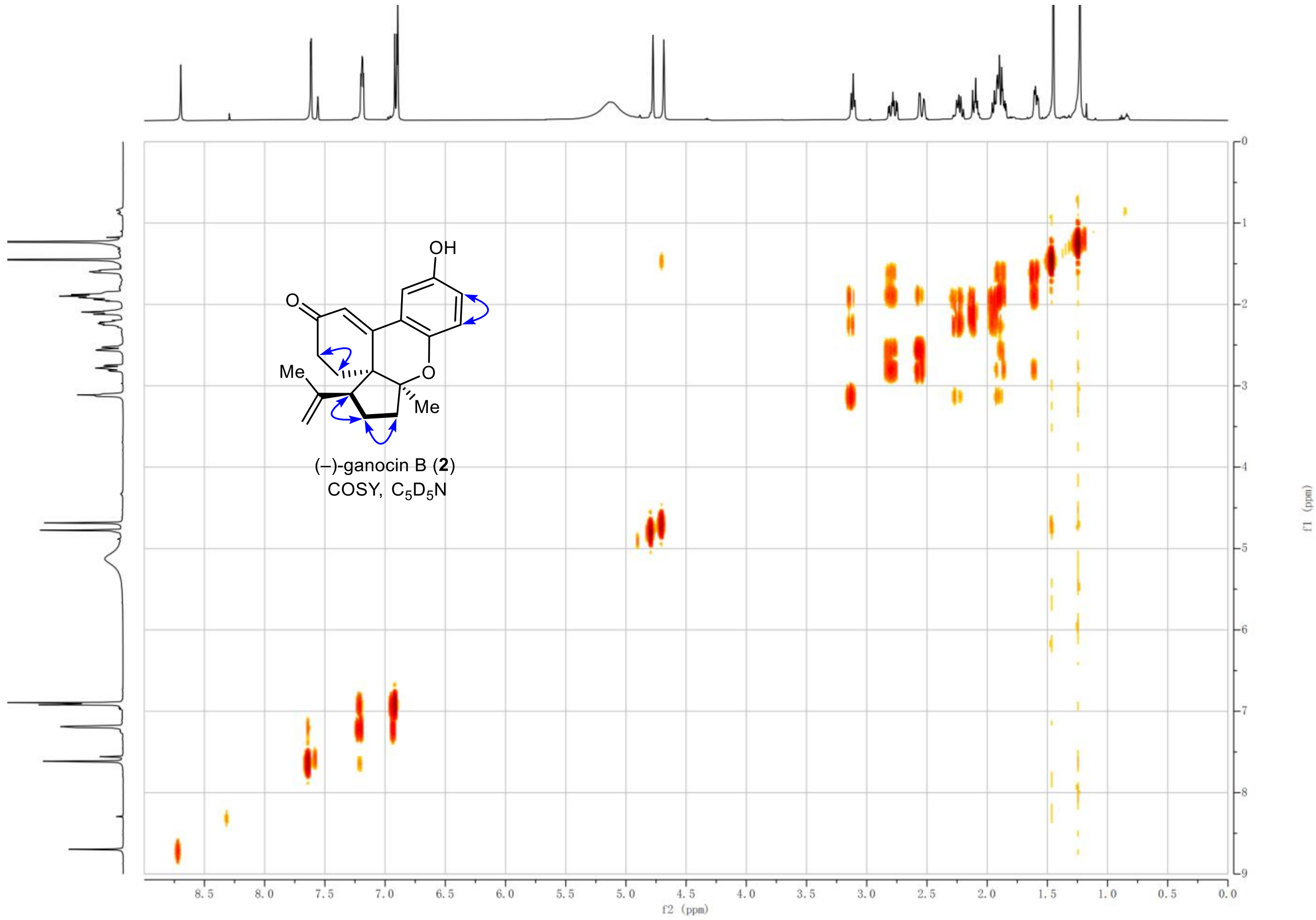

OH
O
Me
O
Me
(–)-ganocin B (2)
COSY, C5D5N
f2 (ppm)
f1 (ppm)

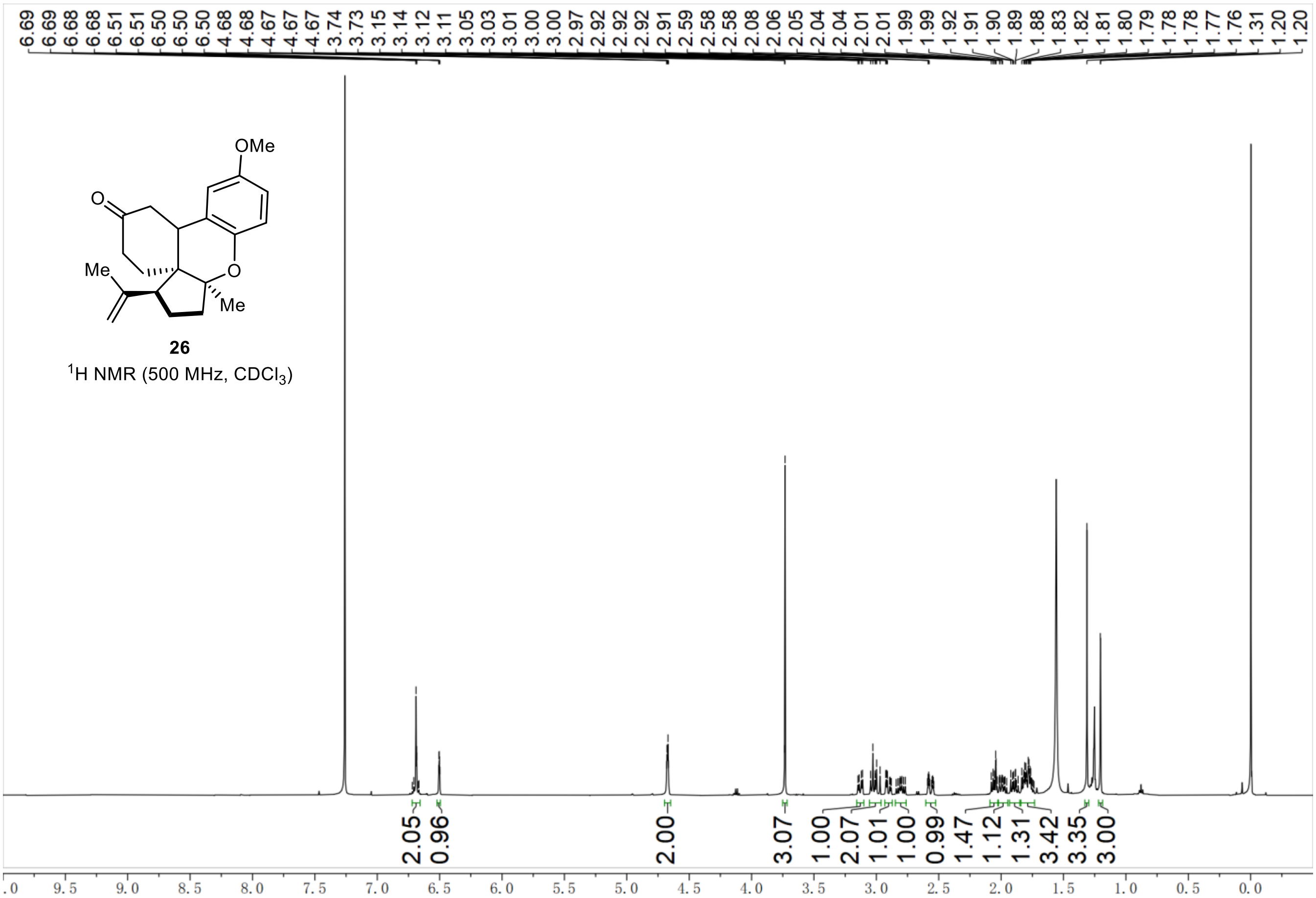

OMe
O
Me
O
Me
26
1H NMR (500 MHz, CDCl3)
f1 (ppm)

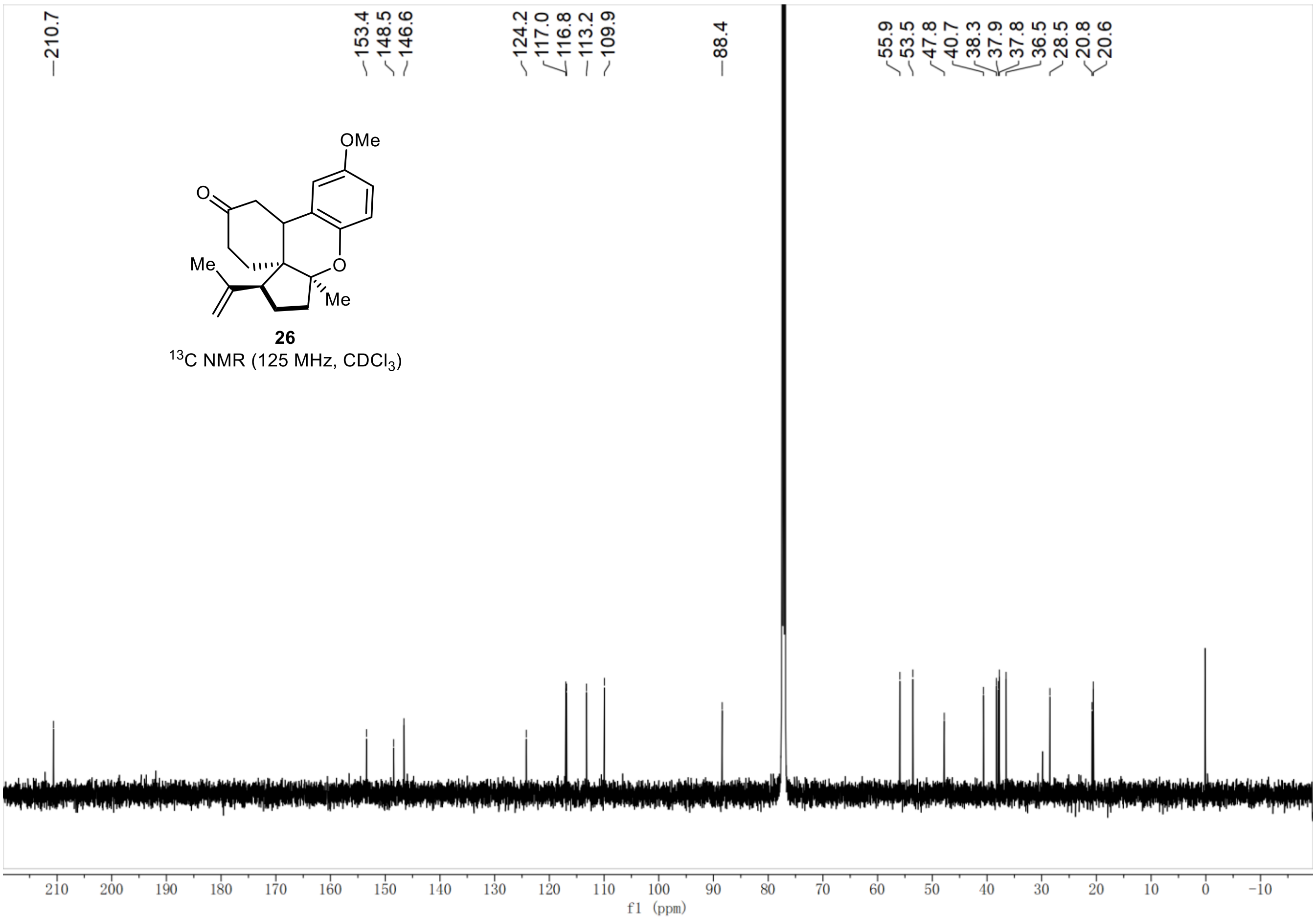

26

^{13}C NMR (125 MHz, $CDCl_3$)

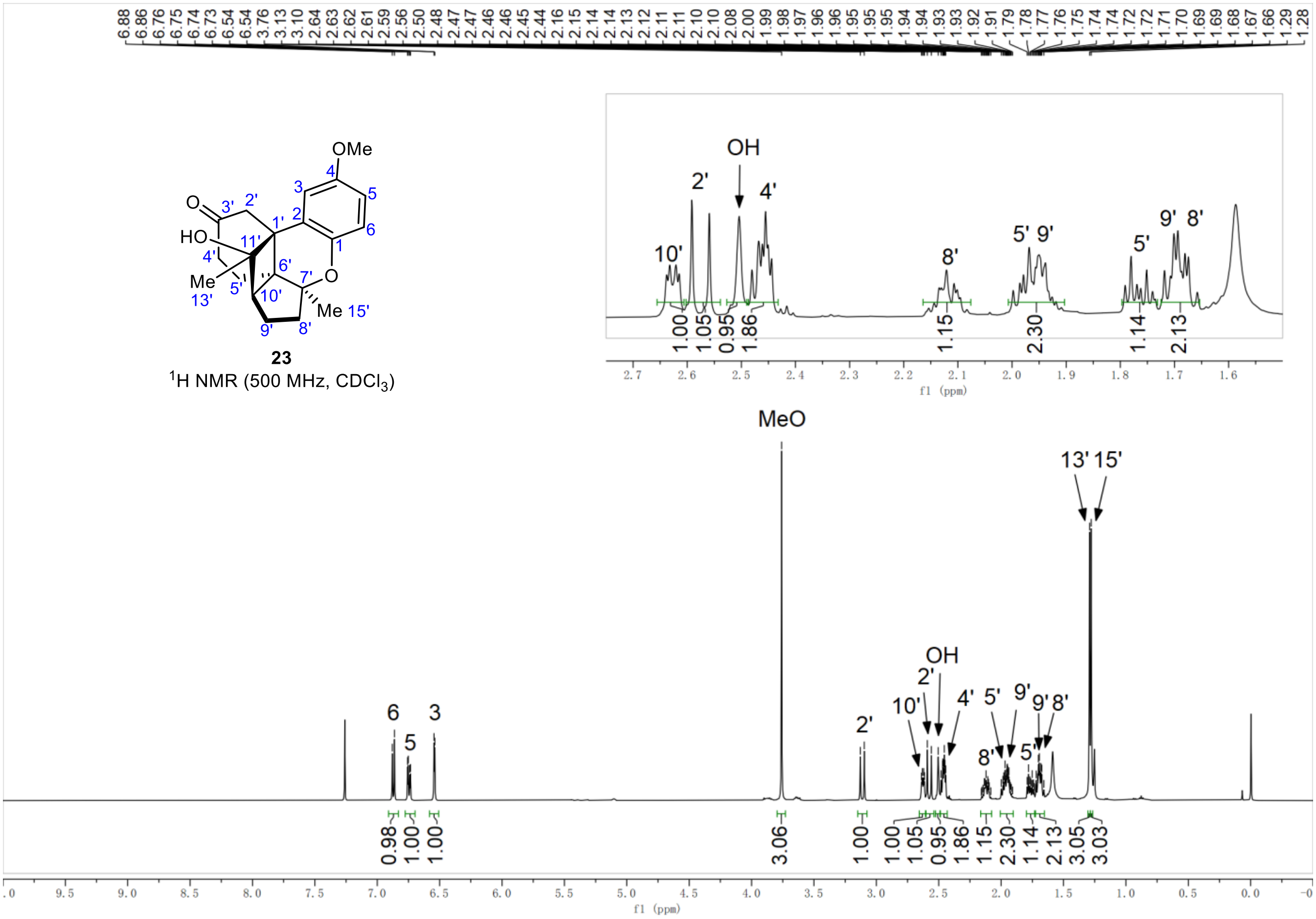

23

^{1}H NMR (500 MHz, $CDCl_3$)

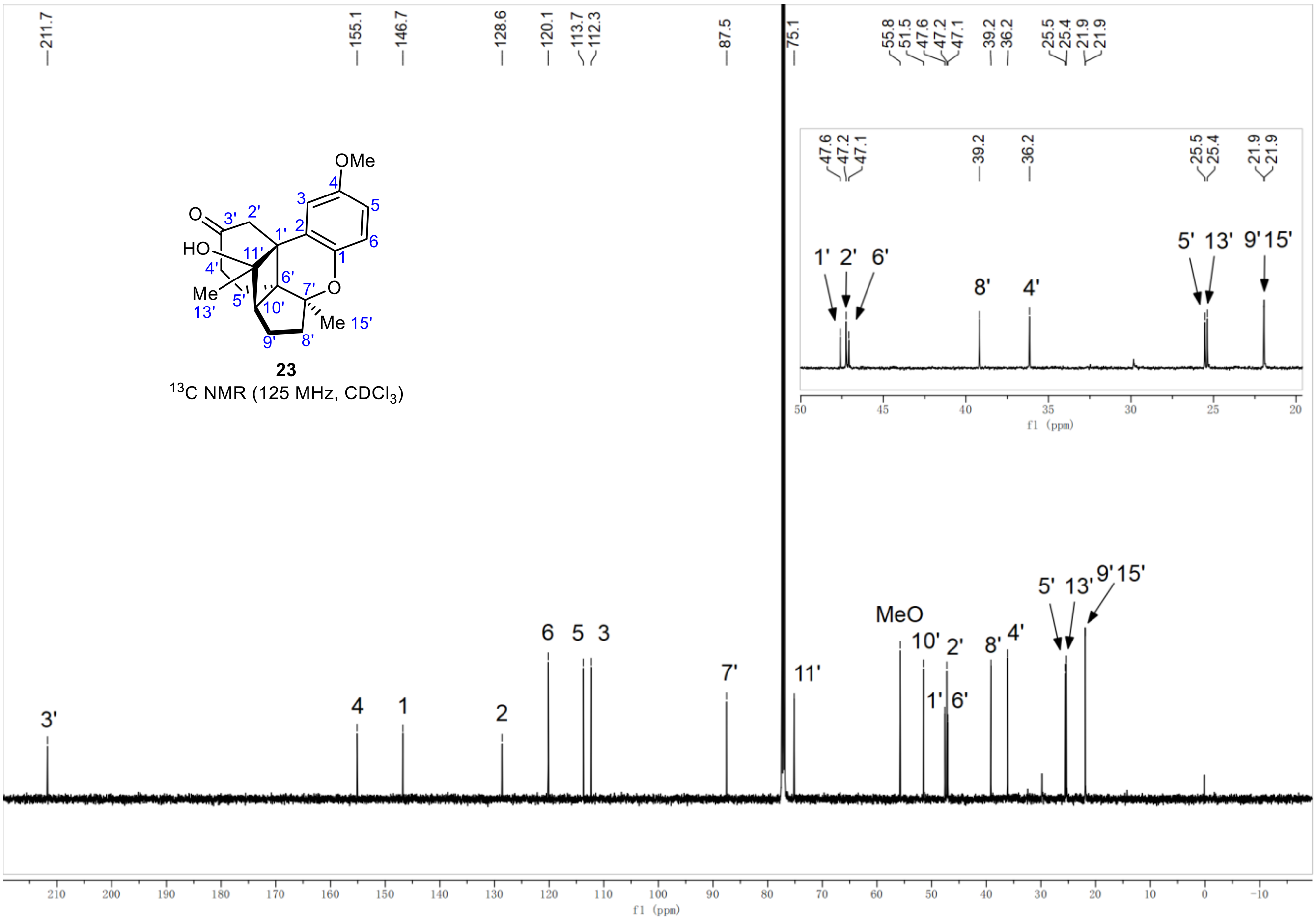
211.7
155.1
146.7
128.6
120.1
113.7
112.3
87.5
75.1
55.8
51.5
47.6
47.2
47.1
39.2
36.2
25.5
25.4
21.9
21.9
OMe
23
^{13}C NMR (125 MHz, $CDCl_3$)
1' 2' 6'
8'
4'
5' 13' 9' 15'
3'
4
1
2
6 5 3
7'
11'
MeO
10'
2'
1' 6'
8'
4'
f1 (ppm)

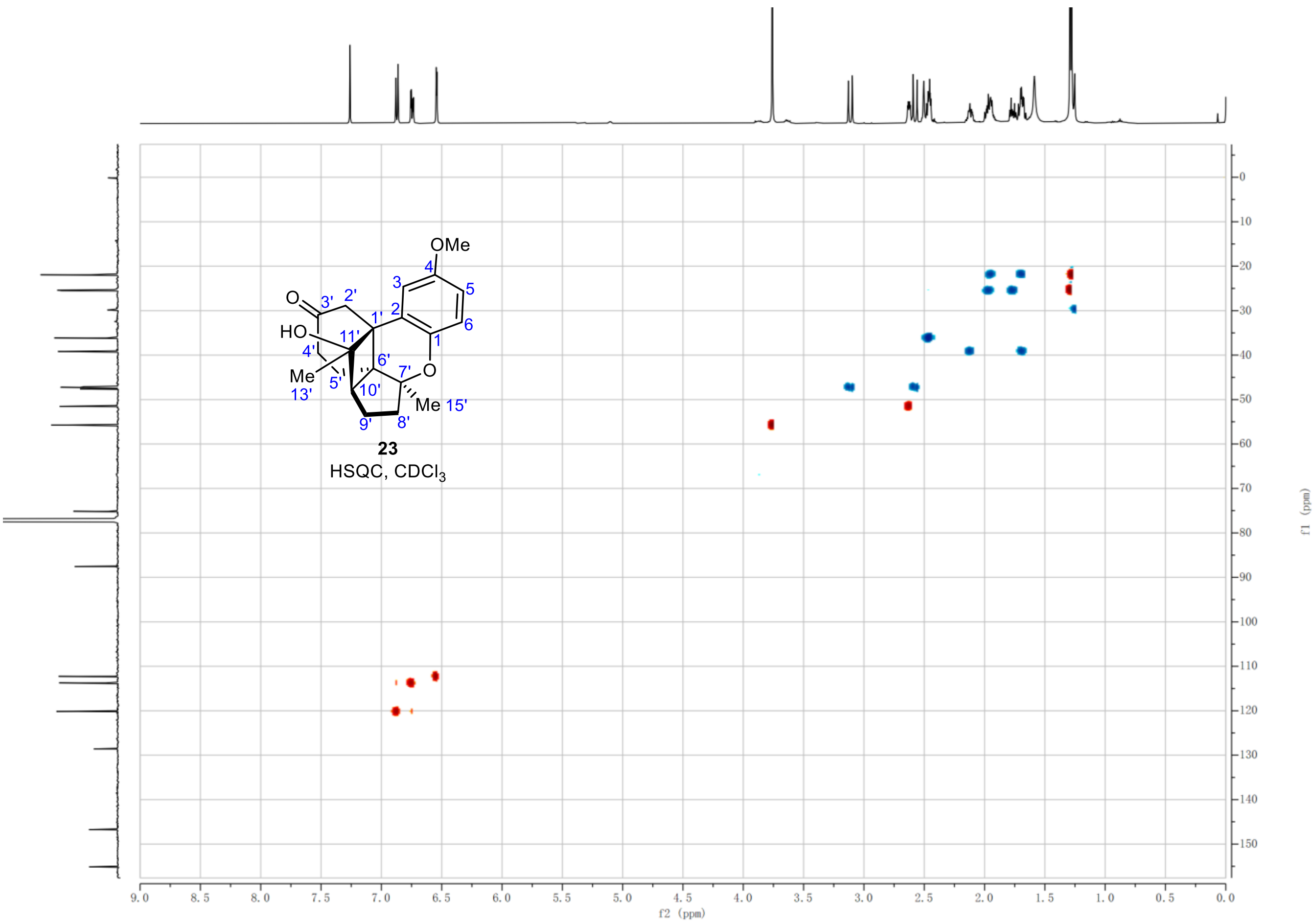
OMe
23
HSQC, $CDCl_3$
f2 (ppm)
f1 (ppm)

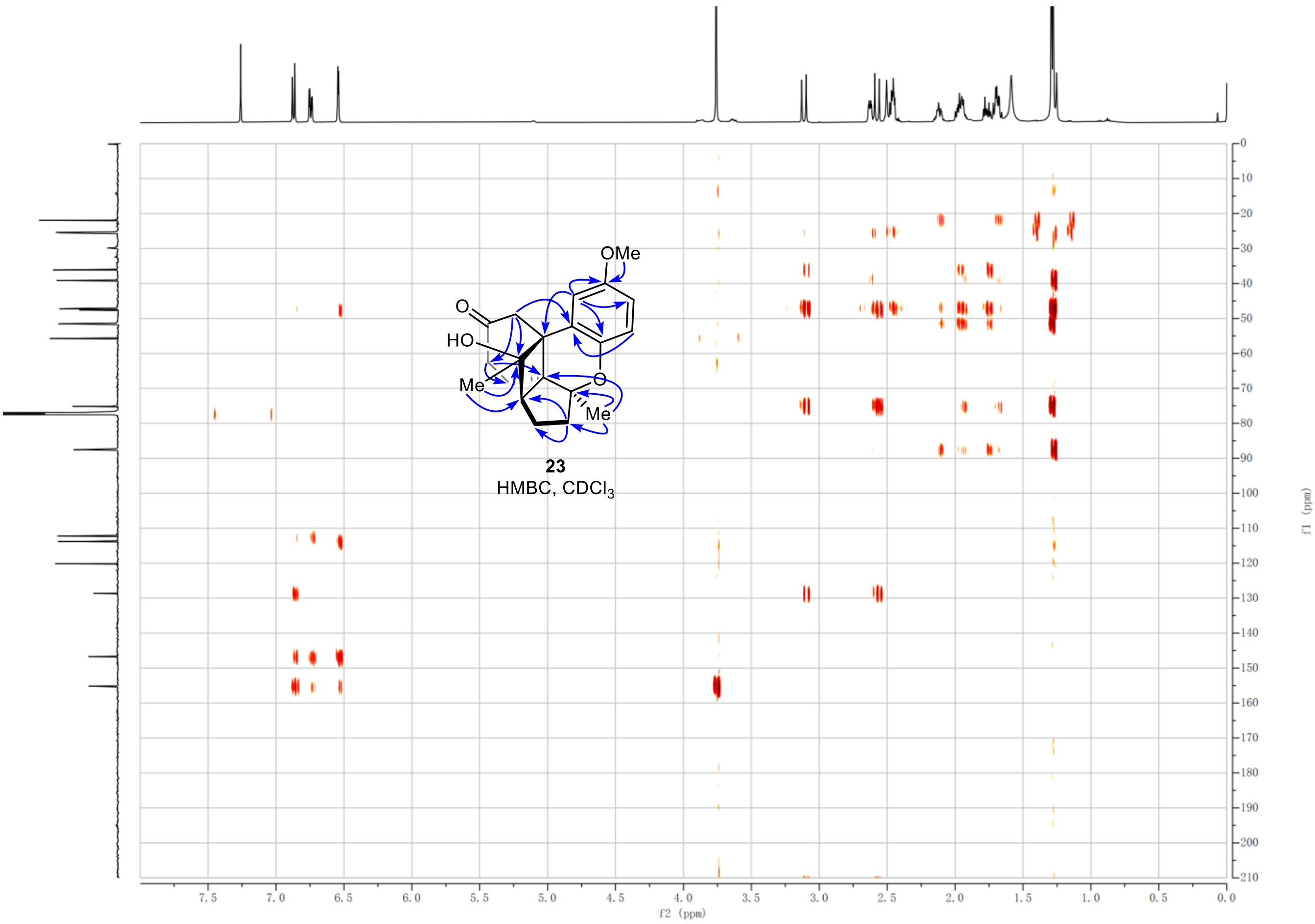
OMe
O
HO
Me
O
Me
23
HMBC, CDCl3
f2 (ppm)
f1 (ppm)

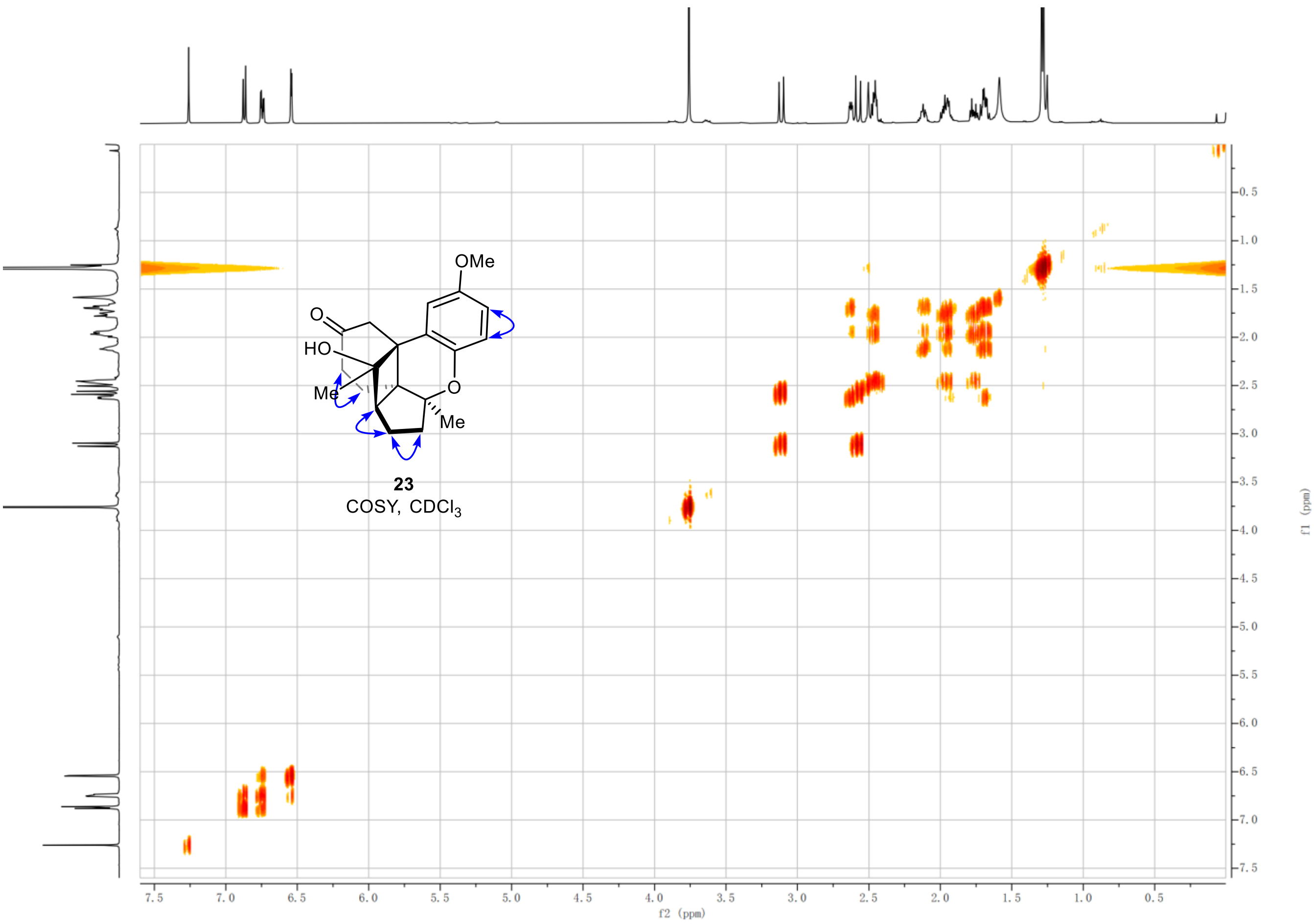
OMe
O
HO
Me
O
Me
23
COSY, CDCl3
f2 (ppm)
f1 (ppm)

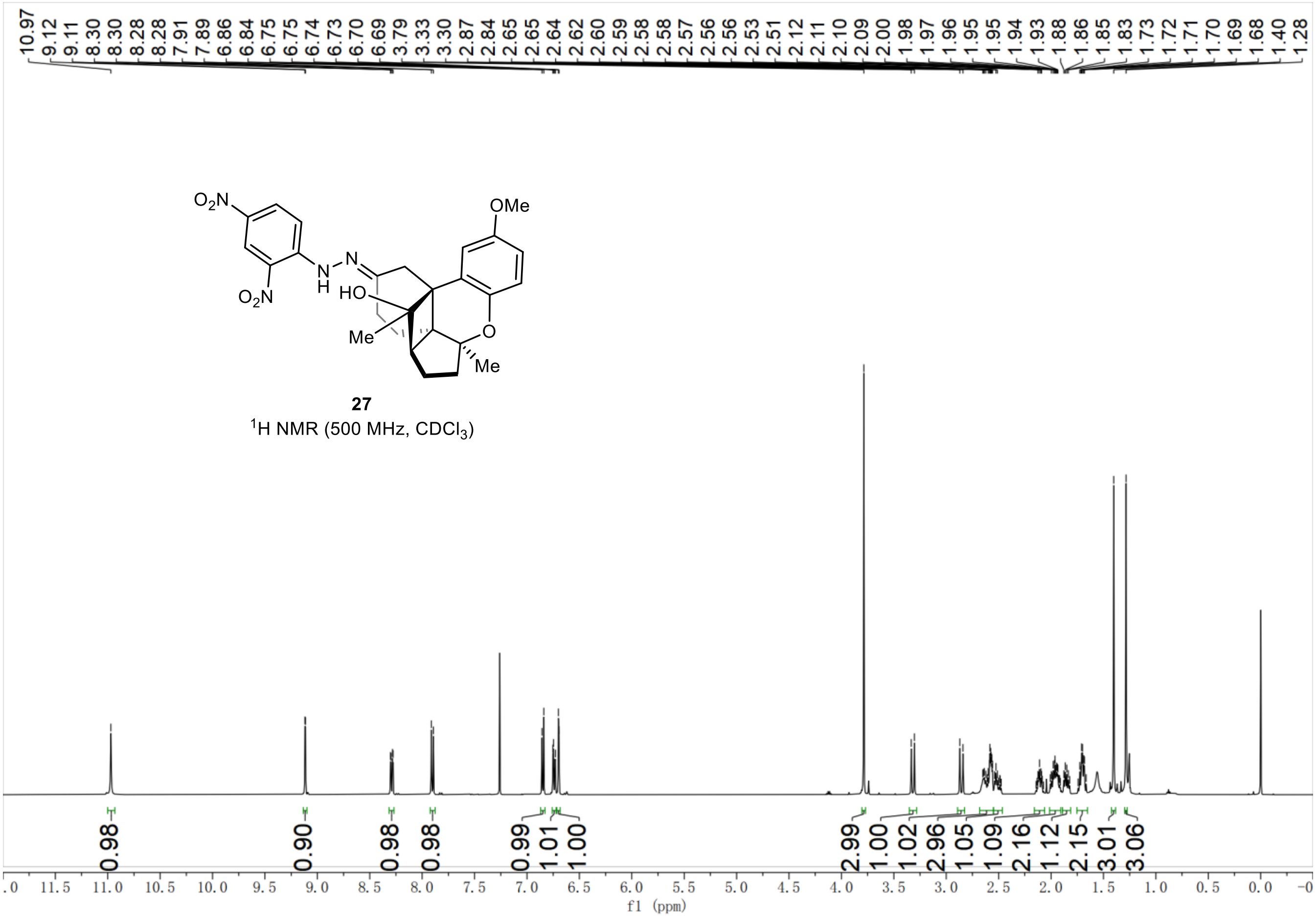
27
^{1}H NMR (500 MHz, CDCl$_3$)

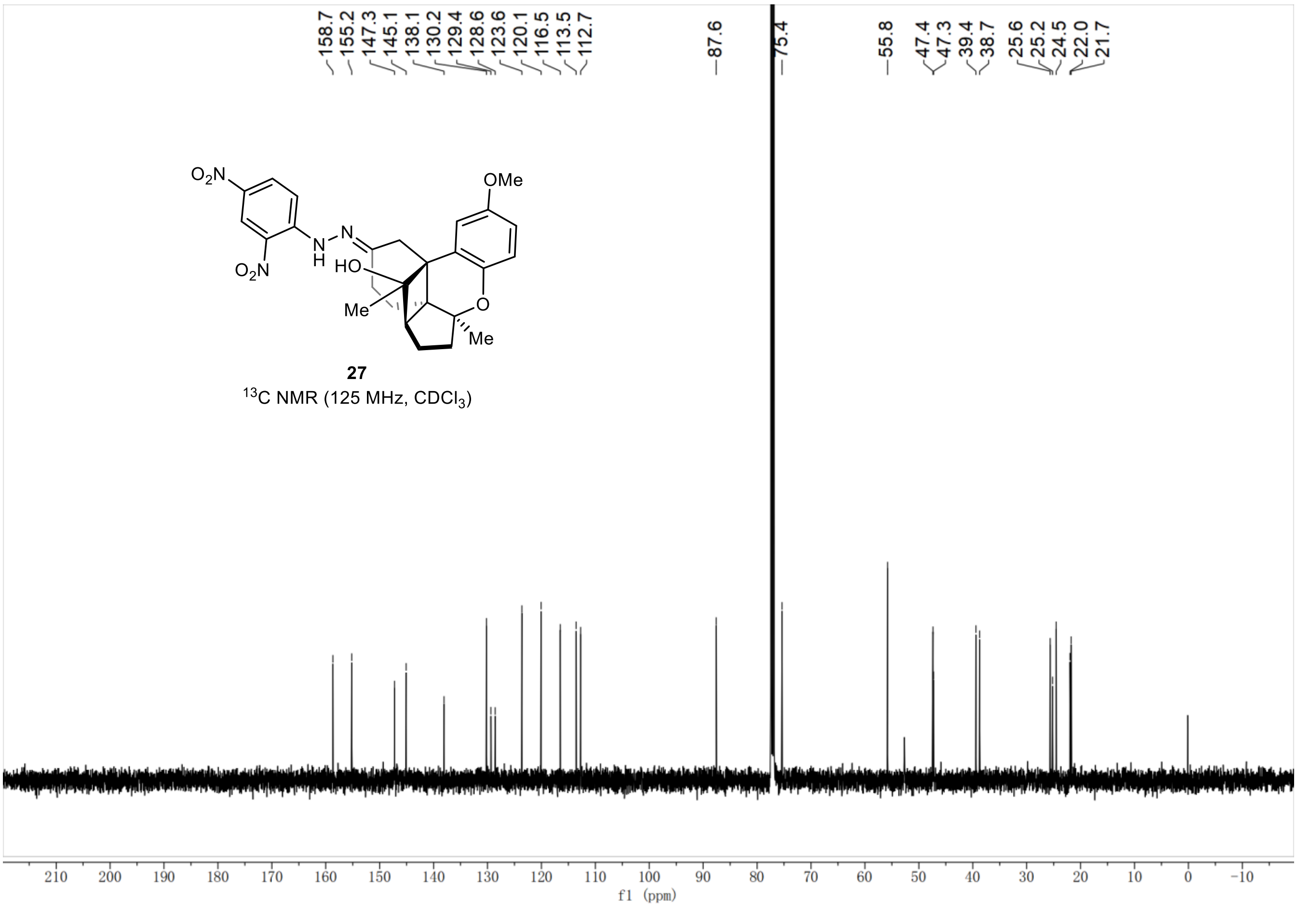
27
^{13}C NMR (125 MHz, CDCl$_3$)

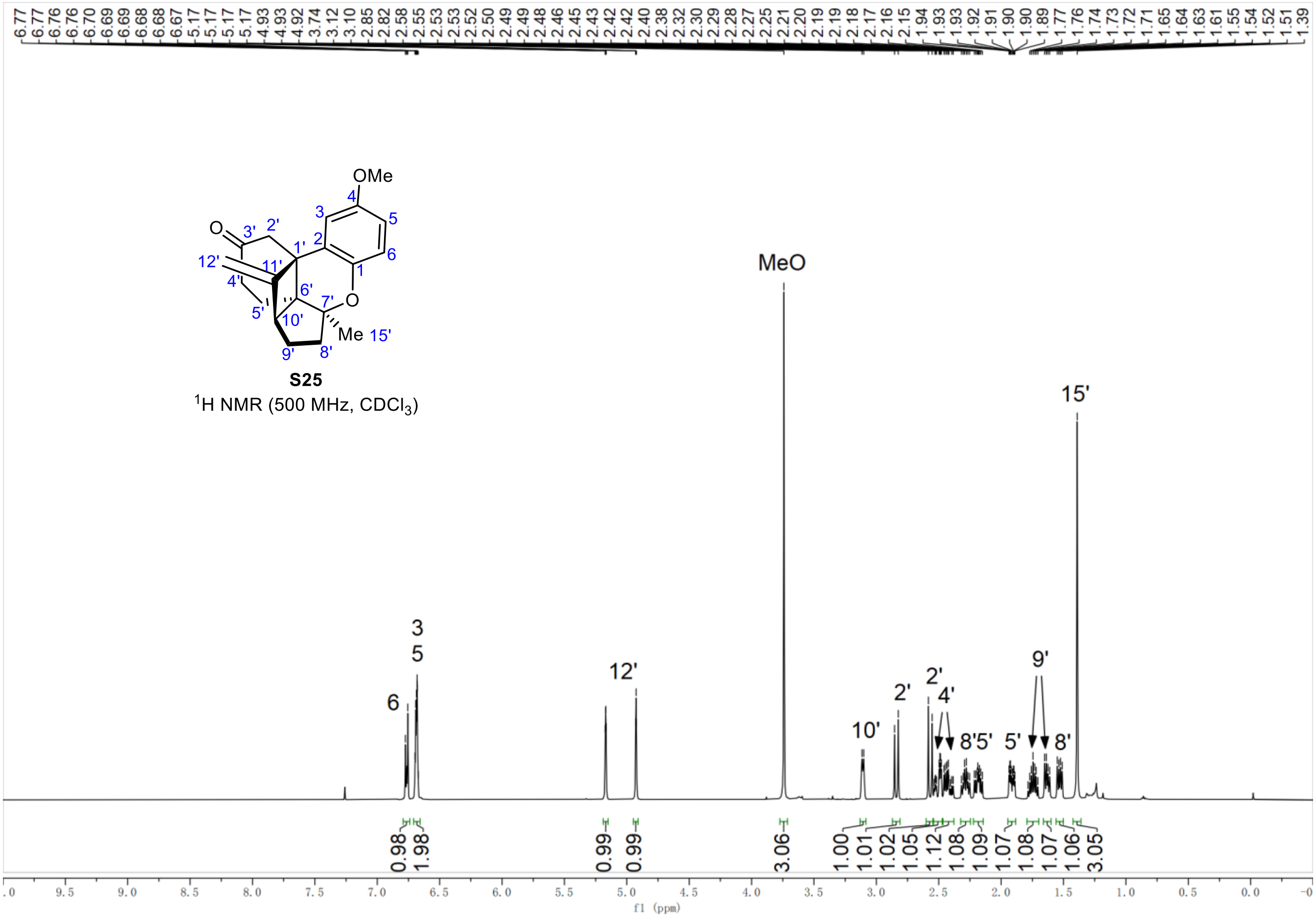
6.77 6.77 6.76 6.76 6.70 6.69 6.69 6.68 6.68 6.67 5.17 5.17 5.17 5.17 4.93 4.93 4.92 3.74 3.12 3.10 2.85 2.82 2.58 2.55 2.53 2.53 2.52 2.50 2.49 2.49 2.48 2.46 2.45 2.43 2.42 2.42 2.40 2.38 2.32 2.30 2.29 2.28 2.27 2.25 2.21 2.20 2.19 2.19 2.18 2.17 2.16 2.15 1.94 1.93 1.93 1.92 1.91 1.90 1.90 1.89 1.77 1.76 1.74 1.73 1.72 1.71 1.65 1.64 1.63 1.61 1.55 1.54 1.52 1.51 1.39
OMe
S25
1H NMR (500 MHz, CDCl3)
MeO
15'
3
5
6
12'
10'
2'
2'
4'
8'5'
5'
9'
8'
0.98 1.98 0.99 0.99 3.06 1.00 1.01 1.02 1.05 1.12 1.08 1.09 1.07 1.08 1.07 1.06 3.05
f1 (ppm)

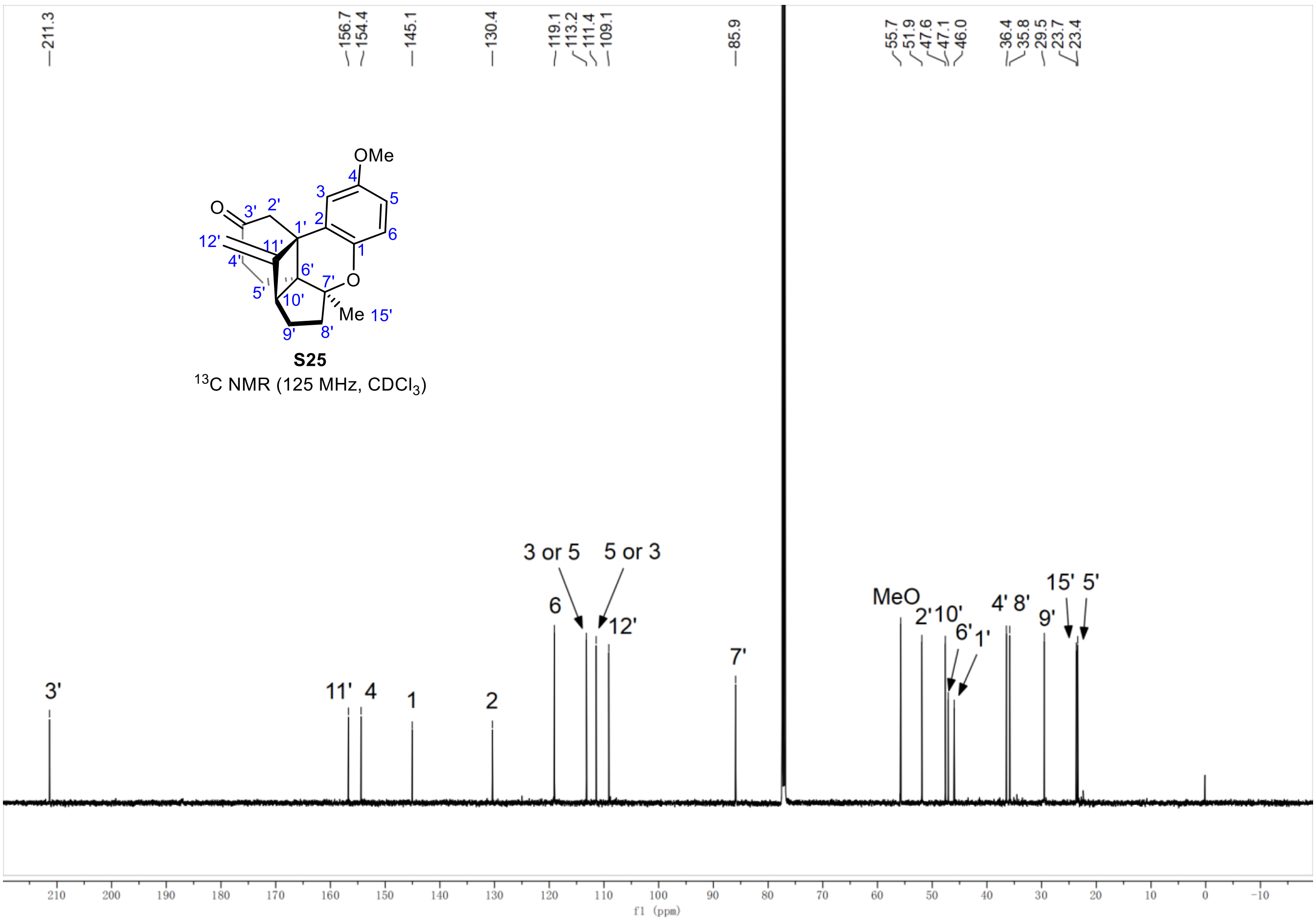
211.3 156.7 154.4 145.1 130.4 119.1 113.2 111.4 109.1 85.9 55.7 51.9 47.6 47.1 46.0 36.4 35.8 29.5 23.7 23.4
OMe
S25
13C NMR (125 MHz, CDCl3)
3 or 5
5 or 3
6
12'
3'
11'
4
1
2
7'
MeO
2'
10'
6'
1'
4'
8'
9'
15'
5'
f1 (ppm)

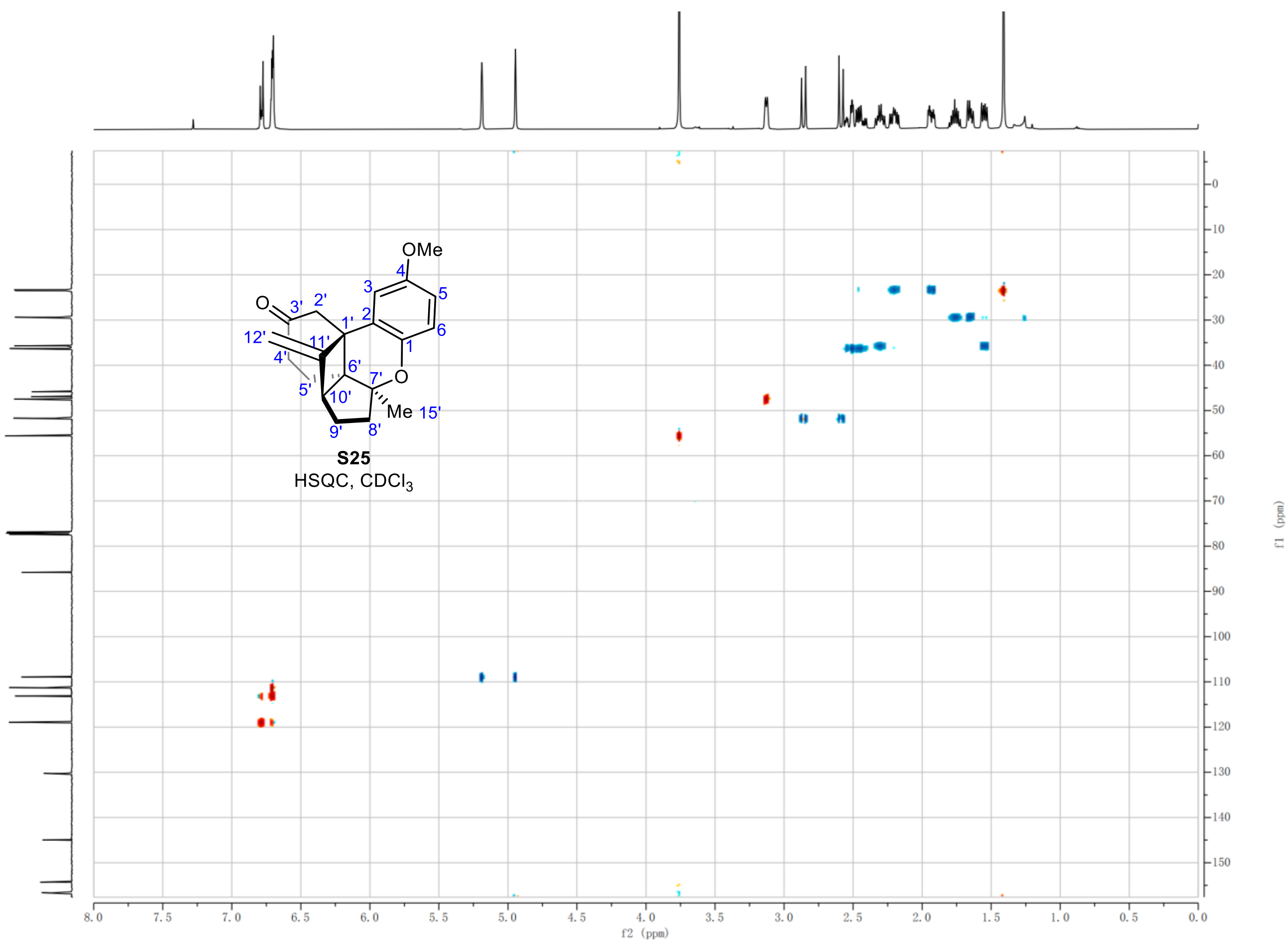
OMe
S25
HSQC, CDCl3
f2 (ppm)
f1 (ppm)

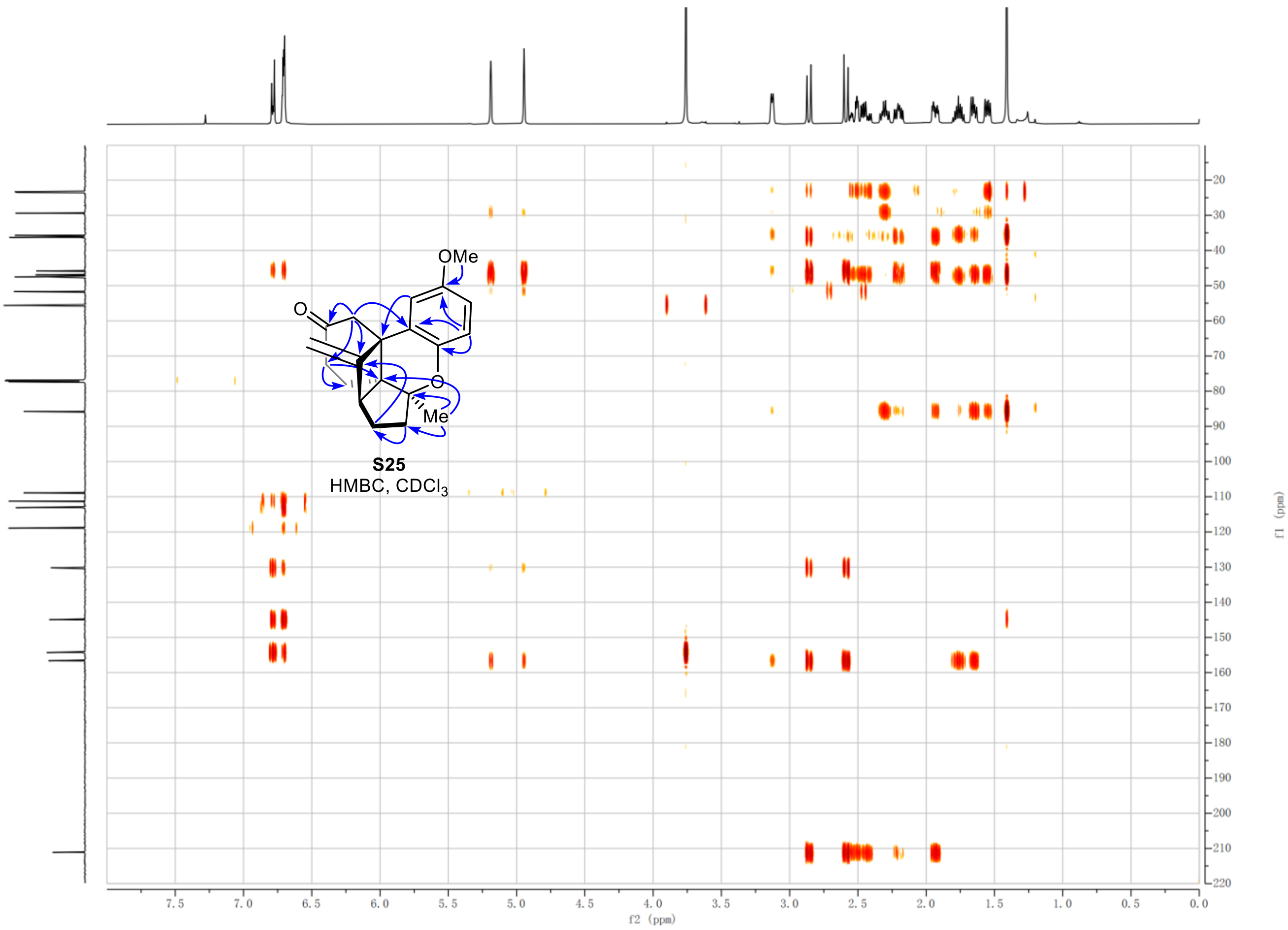
OMe
S25
HMBC, CDCl3
f2 (ppm)
f1 (ppm)

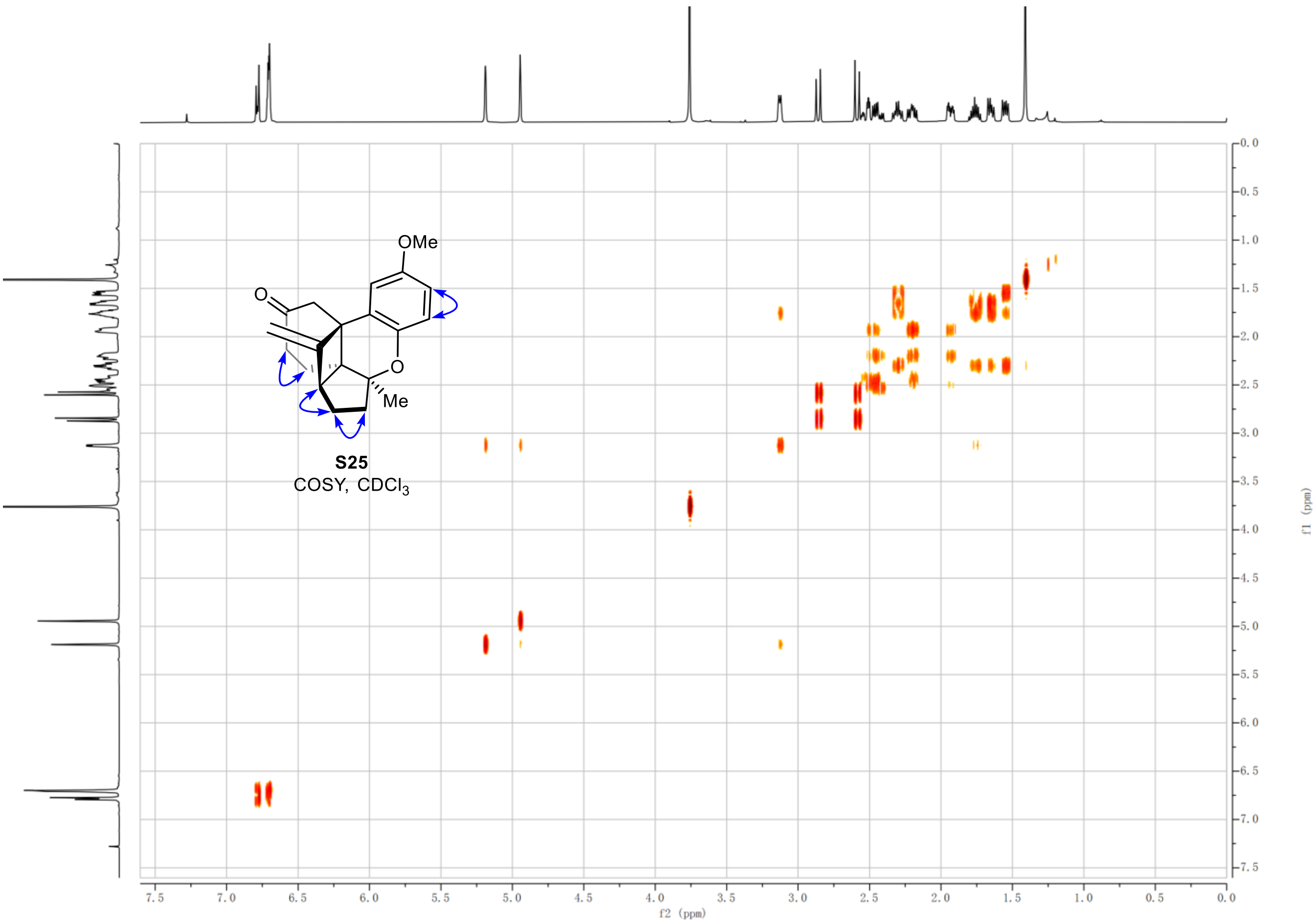
OMe
O
Me
S25
COSY, $CDCl_3$
f2 (ppm)
f1 (ppm)

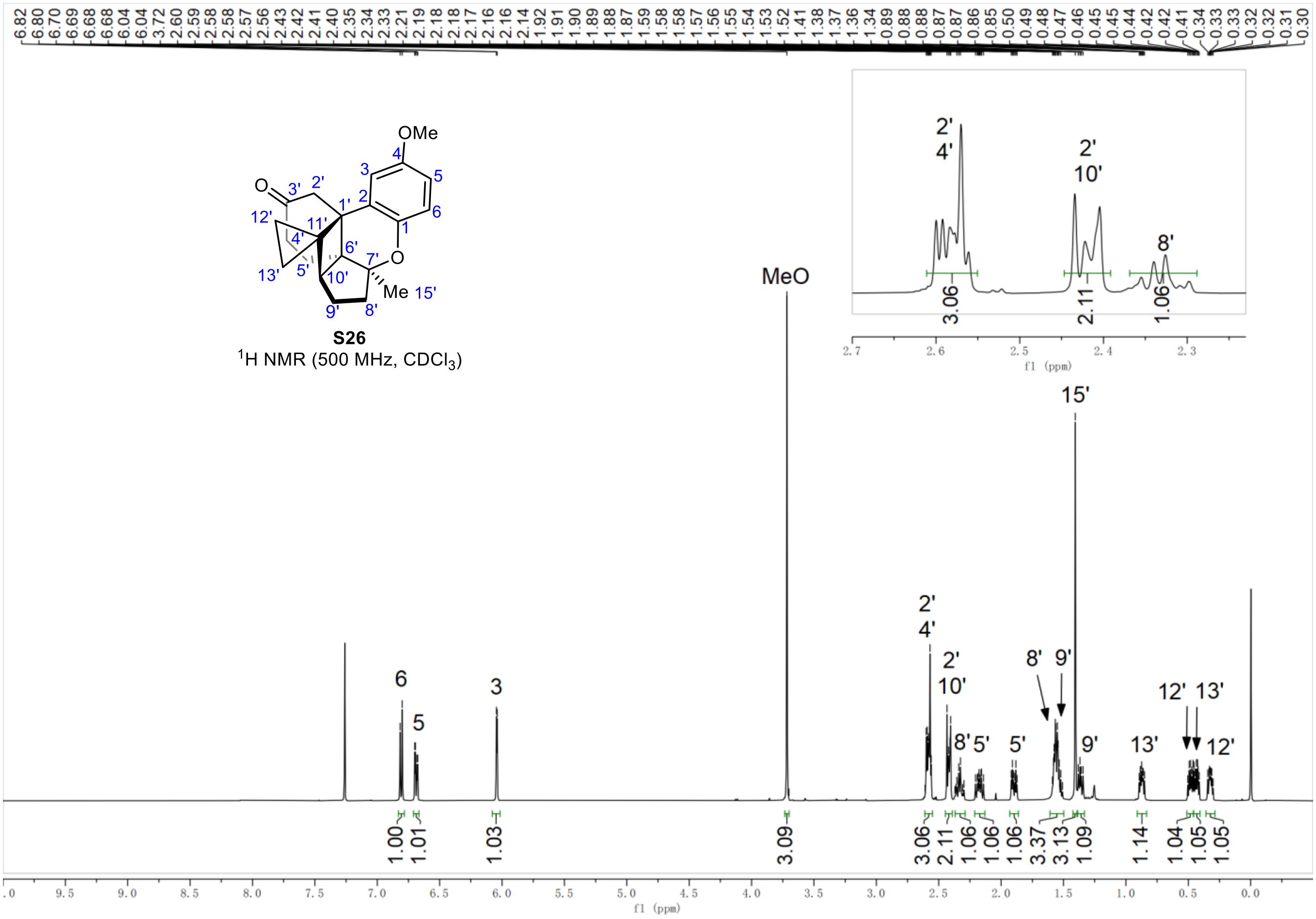
OMe
O
Me 15'
S26
1H NMR (500 MHz, $CDCl_3$)
MeO
15'
6
5
3
2'
4'
2'
10'
8'
5'
5'
8'
9'
9'
13'
12'
13'
12'
f1 (ppm)

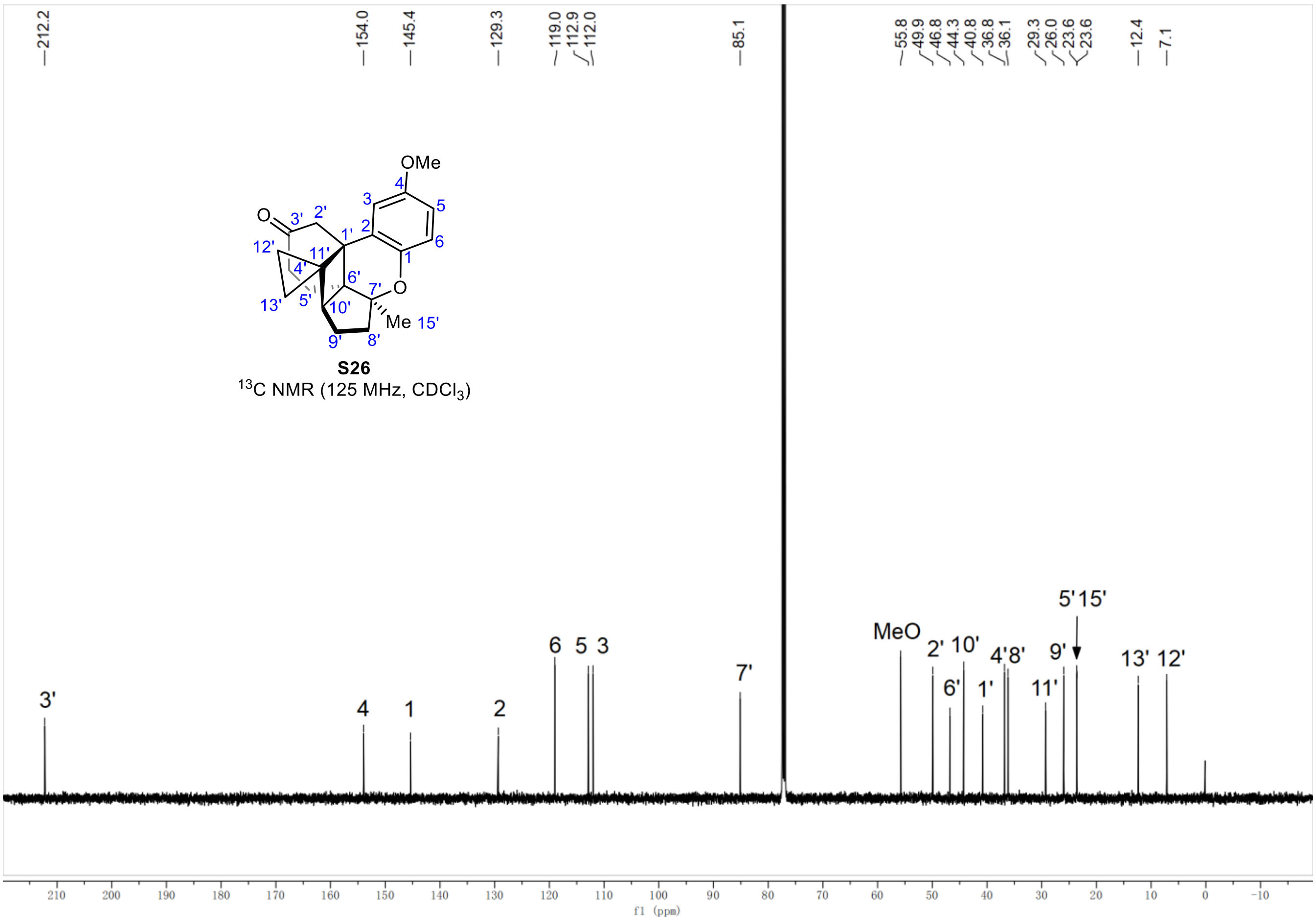

212.2
154.0
145.4
129.3
119.0
112.9
112.0
85.1
55.8
49.9
46.8
44.3
40.8
36.8
36.1
29.3
26.0
23.6
23.6
12.4
7.1
S26
^{13}C NMR (125 MHz, $CDCl_3$)
3'
4
1
2
6 5 3
7'
MeO
2' 10'
6'
1'
4'8'
11'
9'
5'15'
13' 12'
f1 (ppm)

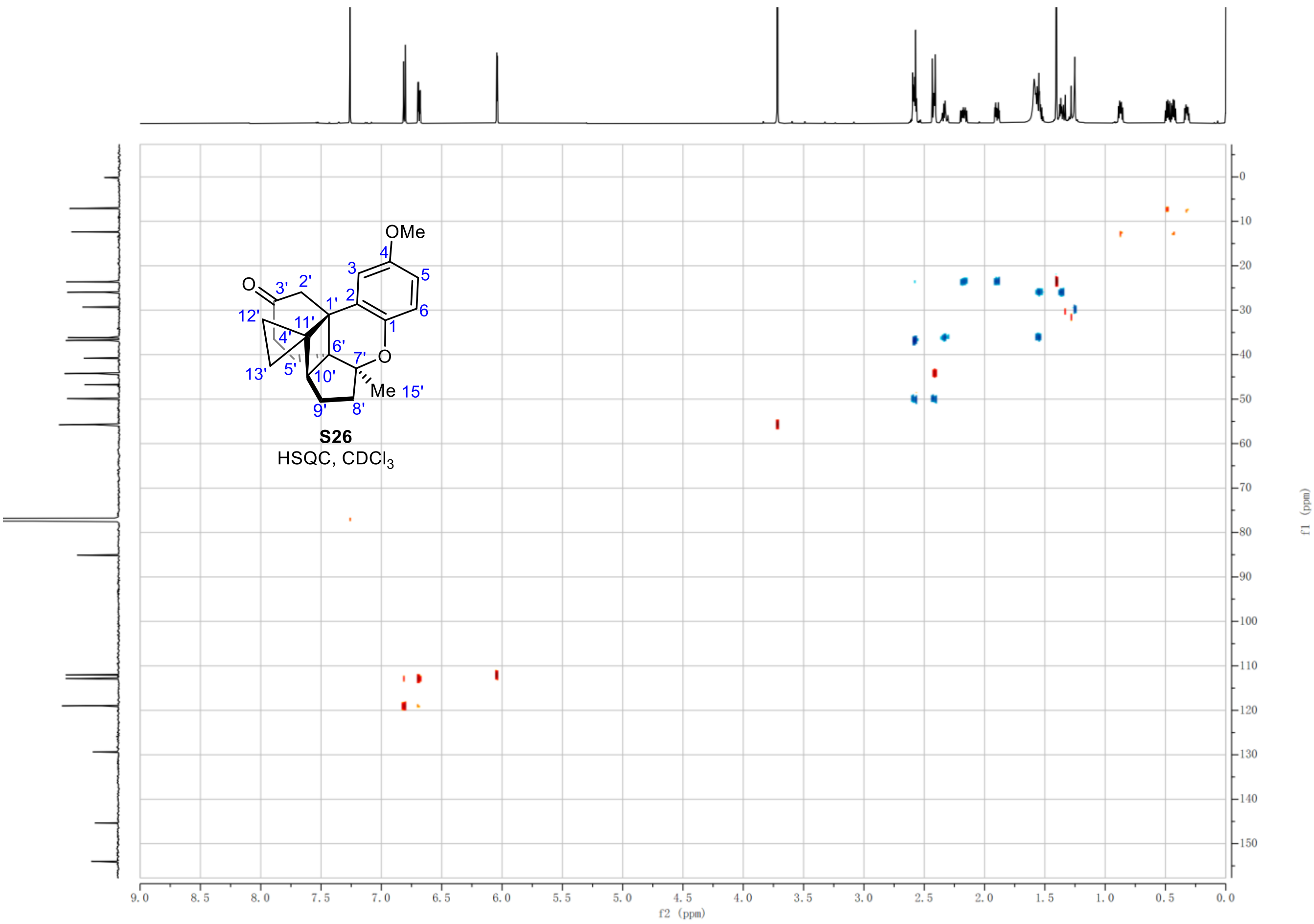

S26
HSQC, $CDCl_3$
f2 (ppm)
f1 (ppm)

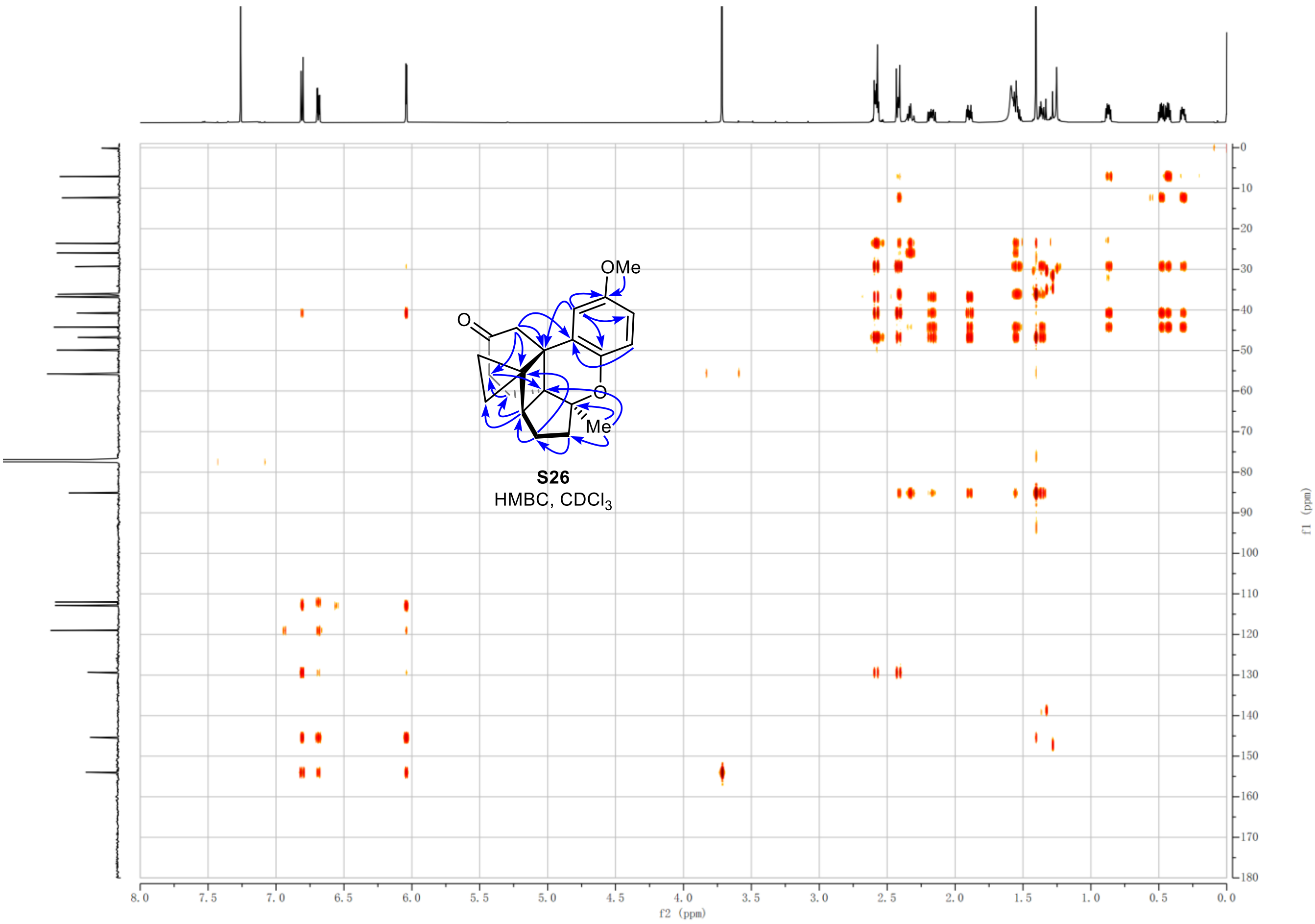
OMe
O
O
Me
S26
HMBC, CDCl3
f2 (ppm)
f1 (ppm)

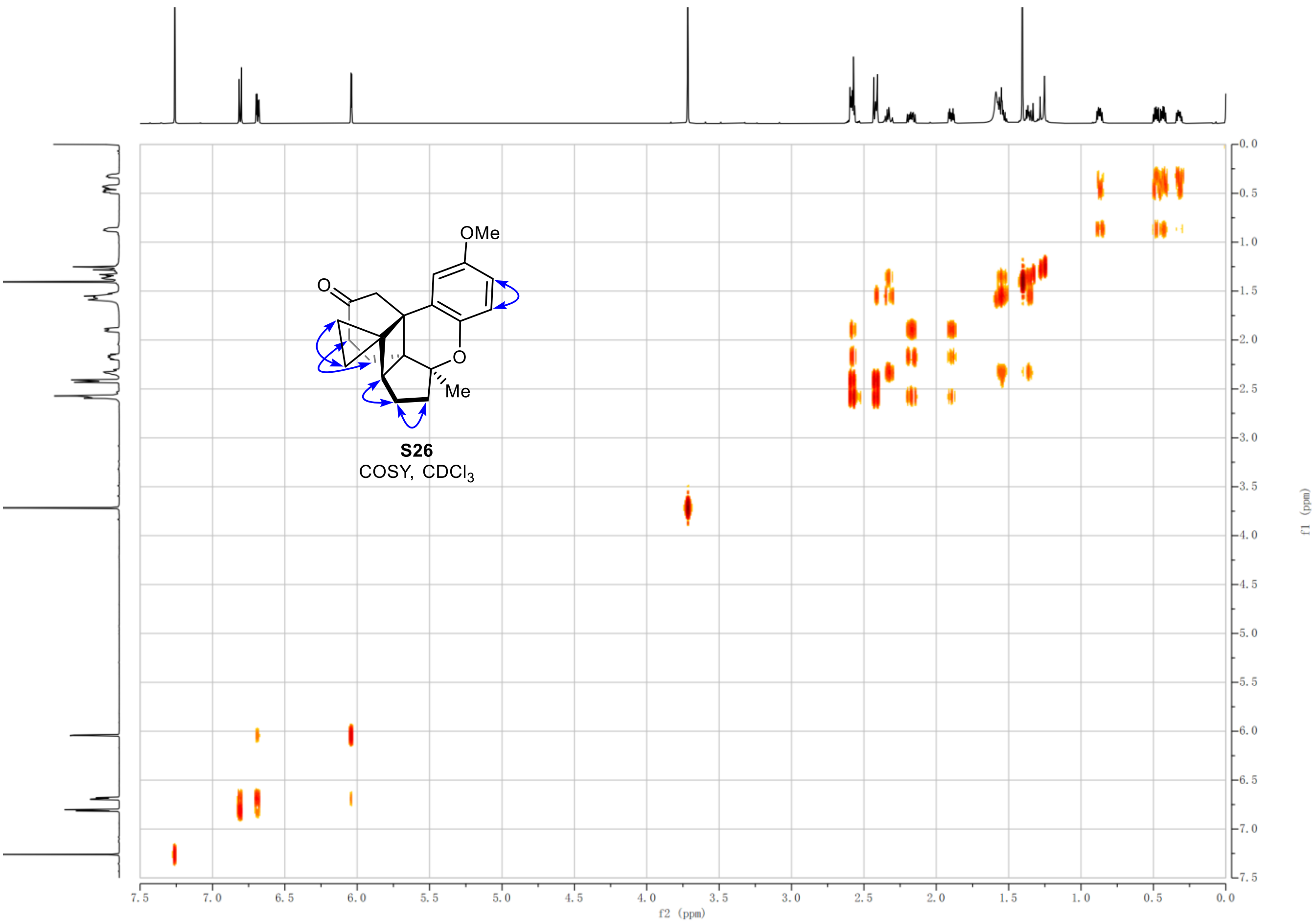
OMe
O
O
Me
S26
COSY, CDCl3
f2 (ppm)
f1 (ppm)

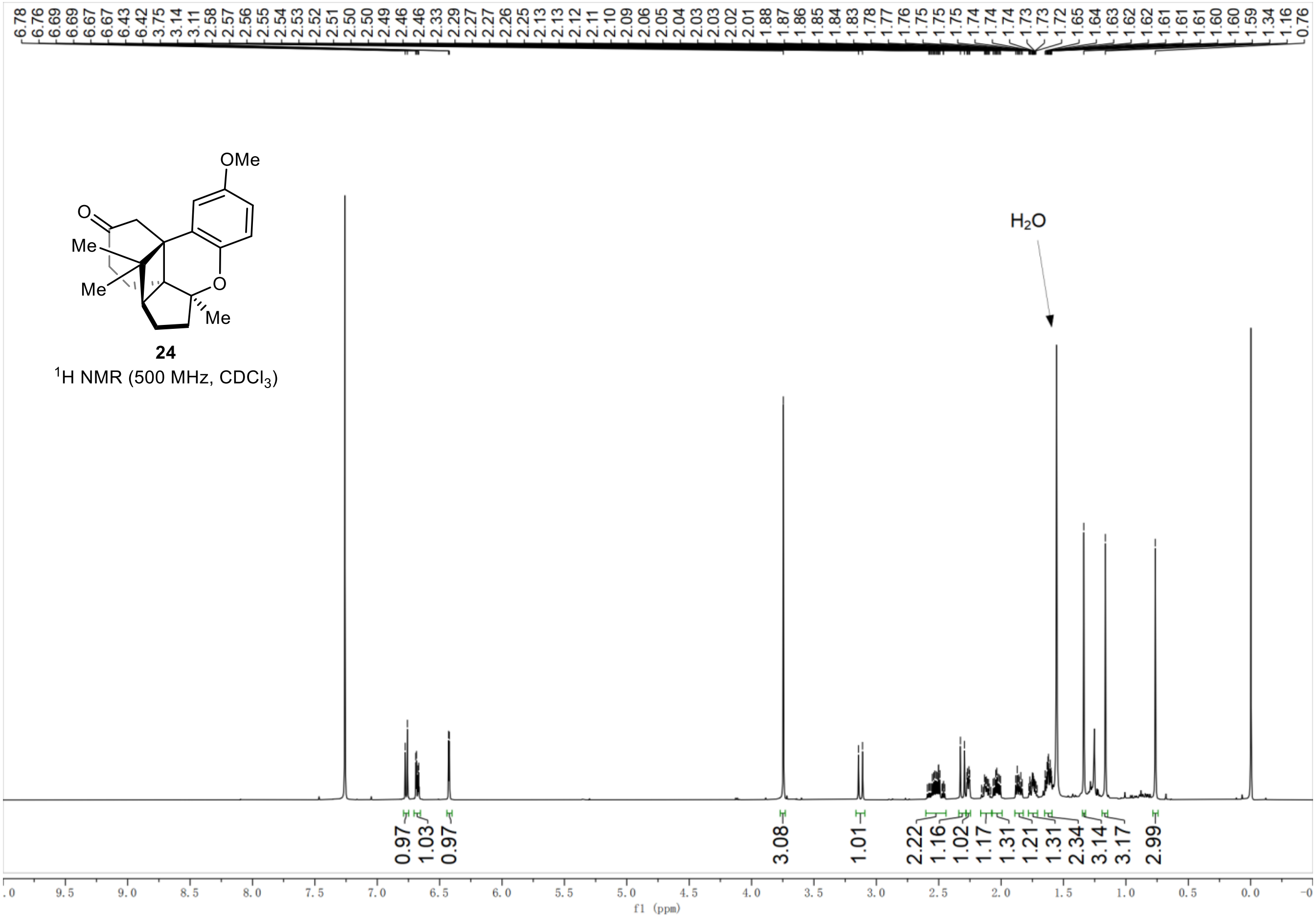
6.78 6.76 6.69 6.69 6.67 6.67 6.43 6.42 3.75 3.14 3.11 2.58 2.57 2.56 2.55 2.54 2.53 2.52 2.51 2.50 2.50 2.49 2.46 2.46 2.33 2.29 2.27 2.27 2.26 2.25 2.13 2.13 2.12 2.11 2.10 2.09 2.06 2.05 2.04 2.03 2.03 2.02 2.01 1.88 1.87 1.86 1.85 1.84 1.83 1.78 1.77 1.76 1.75 1.75 1.75 1.74 1.74 1.74 1.73 1.73 1.72 1.65 1.64 1.63 1.62 1.62 1.61 1.61 1.61 1.60 1.59 1.34 1.16 0.76
OMe
O
Me
Me
O
Me
24
1H NMR (500 MHz, CDCl3)
H2O
0.97 1.03 0.97 3.08 1.01 2.22 1.16 1.02 1.17 1.31 1.21 1.31 2.34 3.14 3.17 2.99
f1 (ppm)

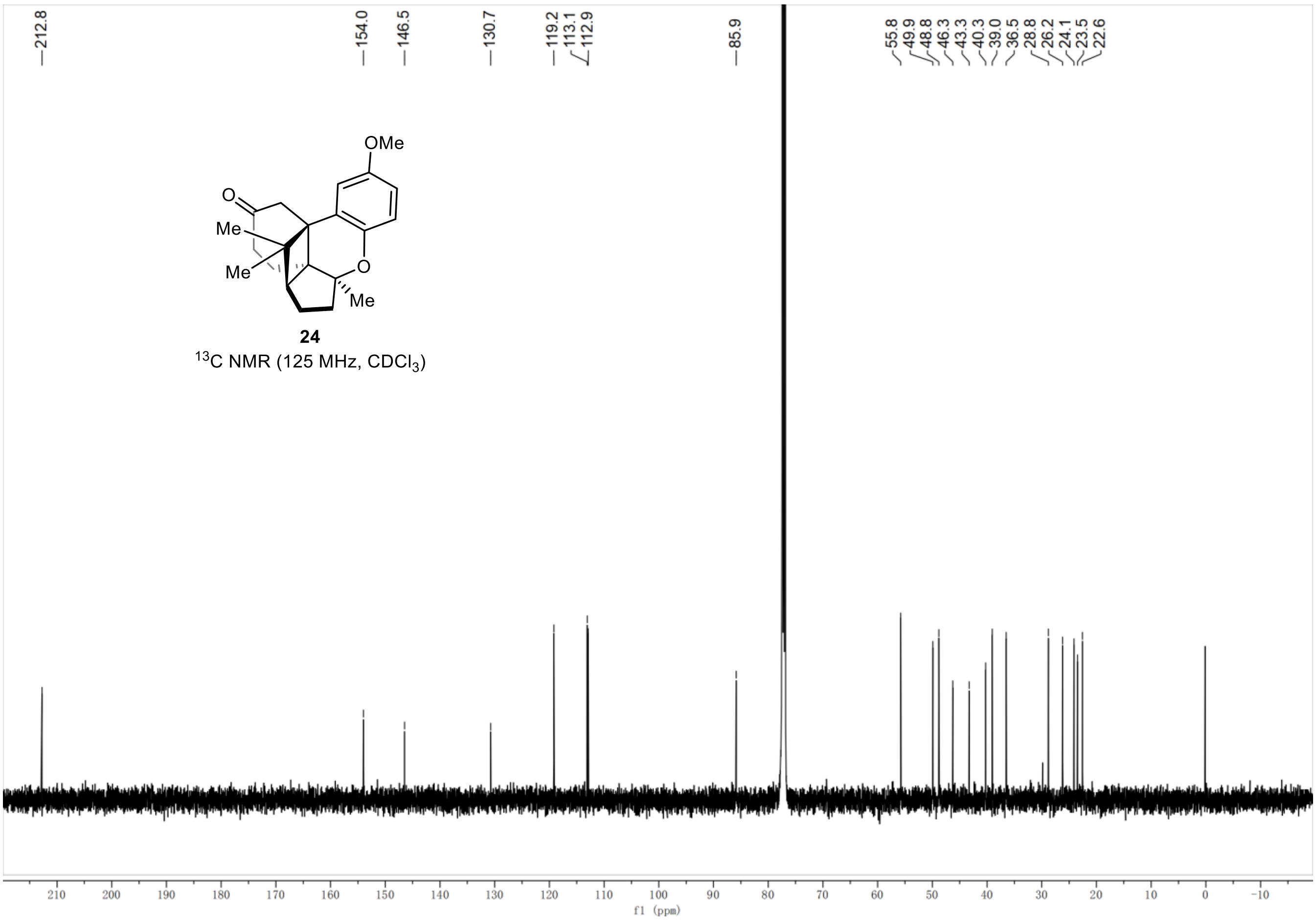
212.8 154.0 146.5 130.7 119.2 113.1 112.9 85.9 55.8 49.9 48.8 46.3 43.3 40.3 39.0 36.5 28.8 26.2 24.1 23.5 22.6
OMe
O
Me
Me
O
Me
24
13C NMR (125 MHz, CDCl3)
f1 (ppm)

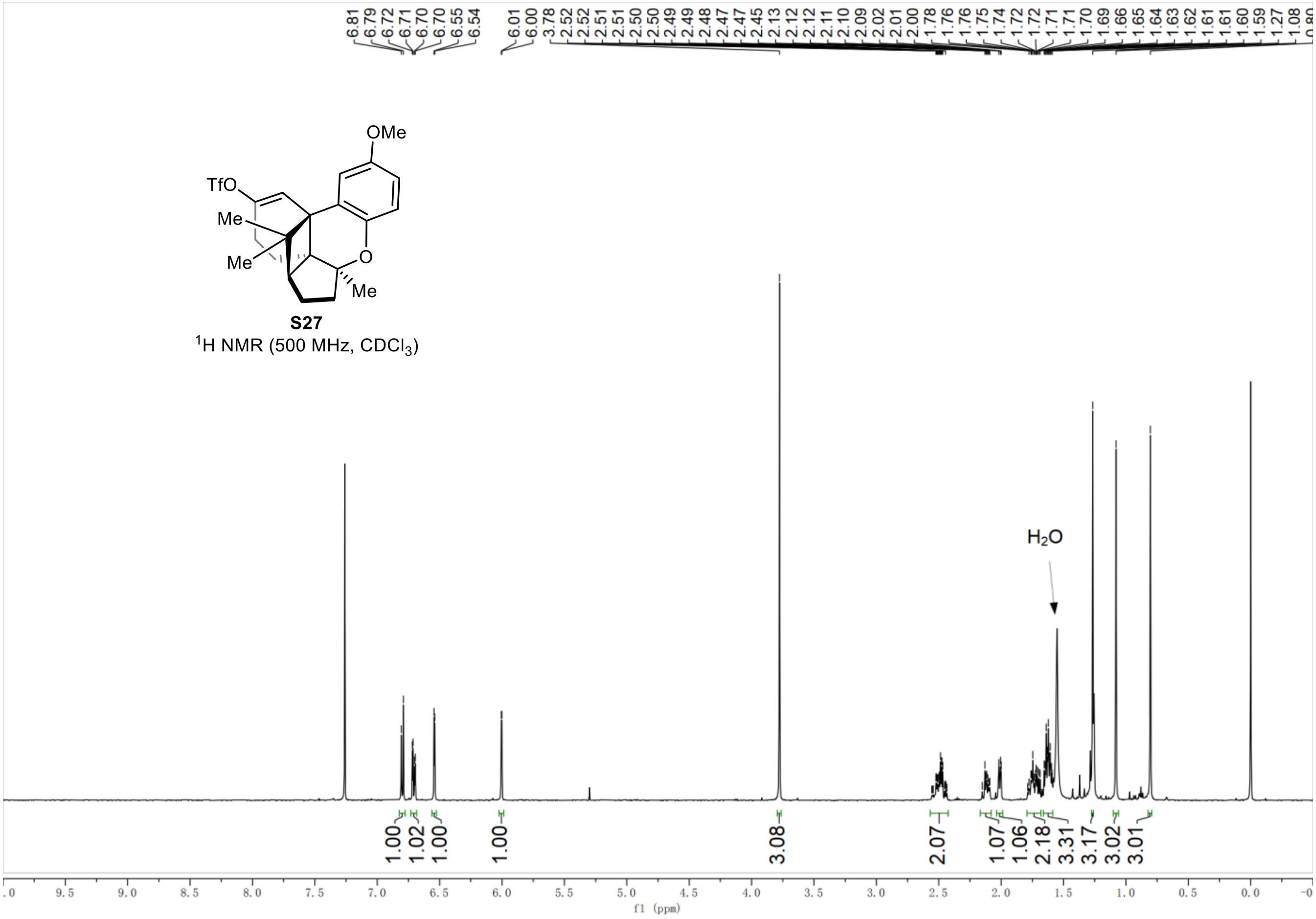
6.81 6.79 6.72 6.71 6.70 6.70 6.55 6.54 6.01 6.00 3.78 2.52 2.52 2.51 2.50 2.50 2.49 2.49 2.48 2.47 2.47 2.45 2.13 2.12 2.12 2.11 2.10 2.09 2.02 2.01 2.00 1.78 1.76 1.76 1.75 1.74 1.72 1.72 1.71 1.71 1.70 1.69 1.66 1.65 1.64 1.63 1.62 1.61 1.61 1.60 1.59 1.27 1.08
OMe
TfO
Me
Me
O
Me
S27
^{1}H NMR (500 MHz, $CDCl_3$)
H_2O
1.00 1.02 1.00 1.00 3.08 2.07 1.07 1.06 2.18 3.31 3.17 3.02 3.01
f1 (ppm)

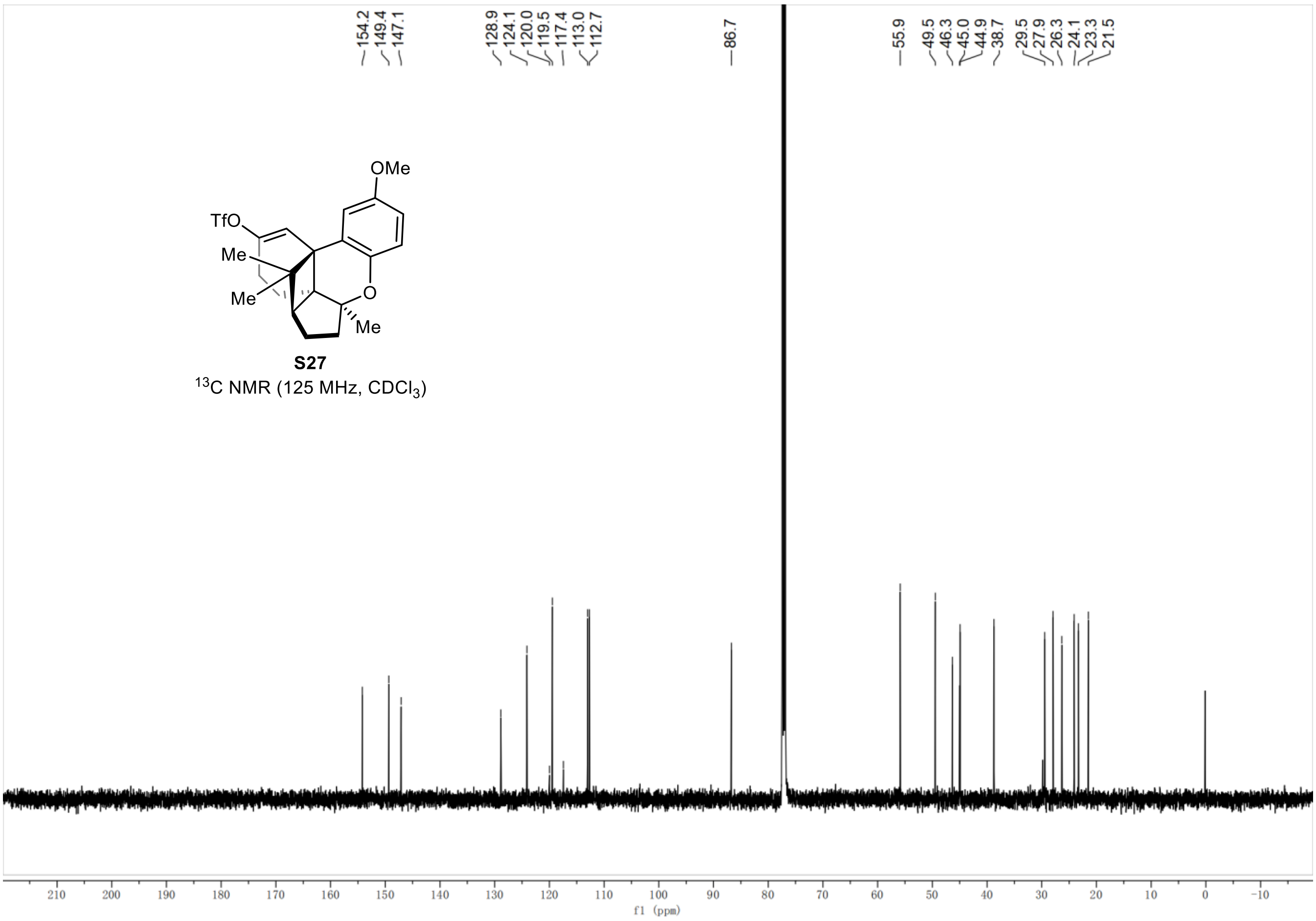
154.2 149.4 147.1 128.9 124.1 120.0 119.5 117.4 113.0 112.7 86.7 55.9 49.5 46.3 45.0 44.9 38.7 29.5 27.9 26.3 24.1 23.3 21.5
OMe
TfO
Me
Me
O
Me
S27
^{13}C NMR (125 MHz, $CDCl_3$)
f1 (ppm)

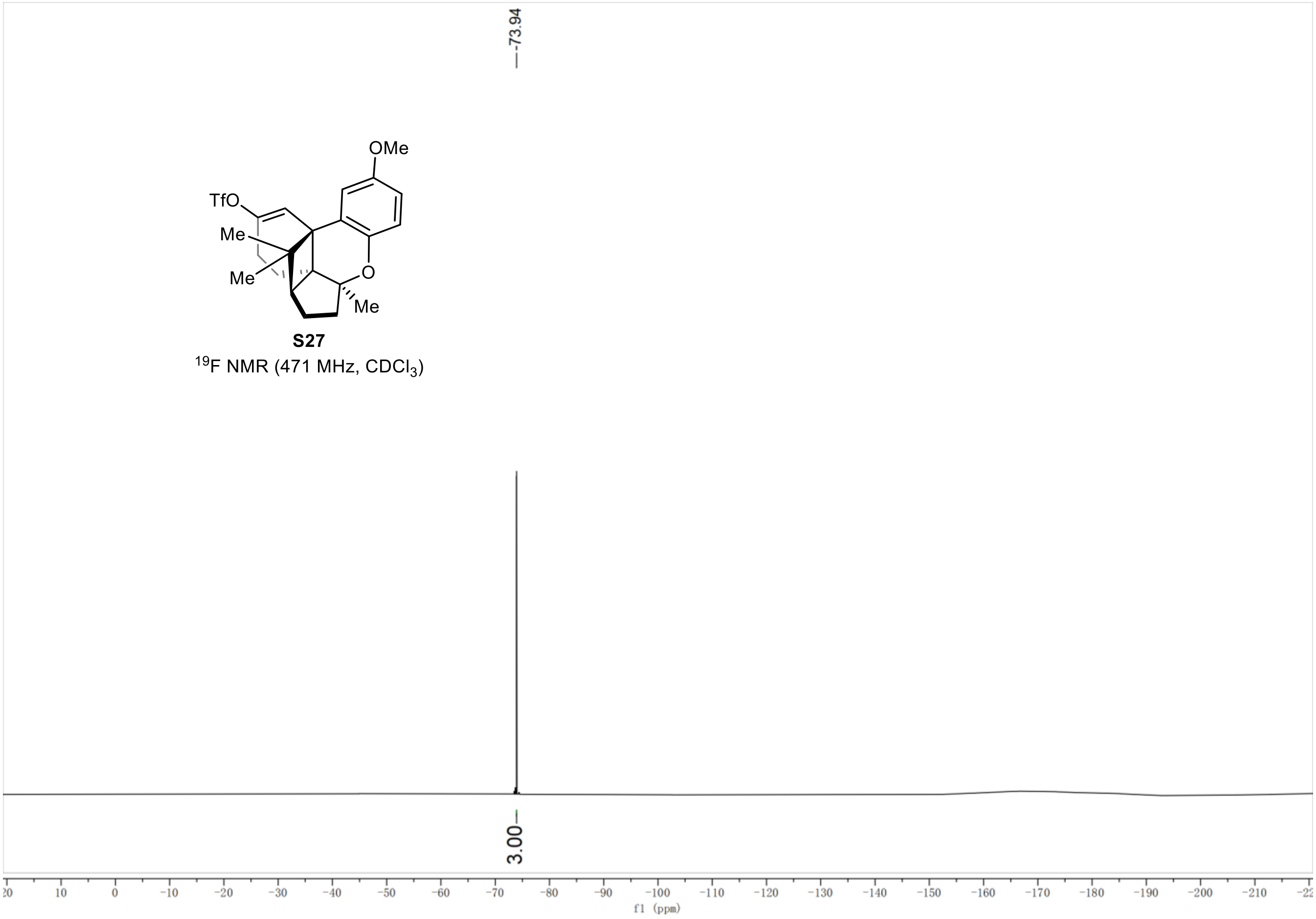
-73.94
OMe
TfO
Me
Me
O
Me
S27
^{19}F NMR (471 MHz, CDCl$_3$)
3.00
f1 (ppm)

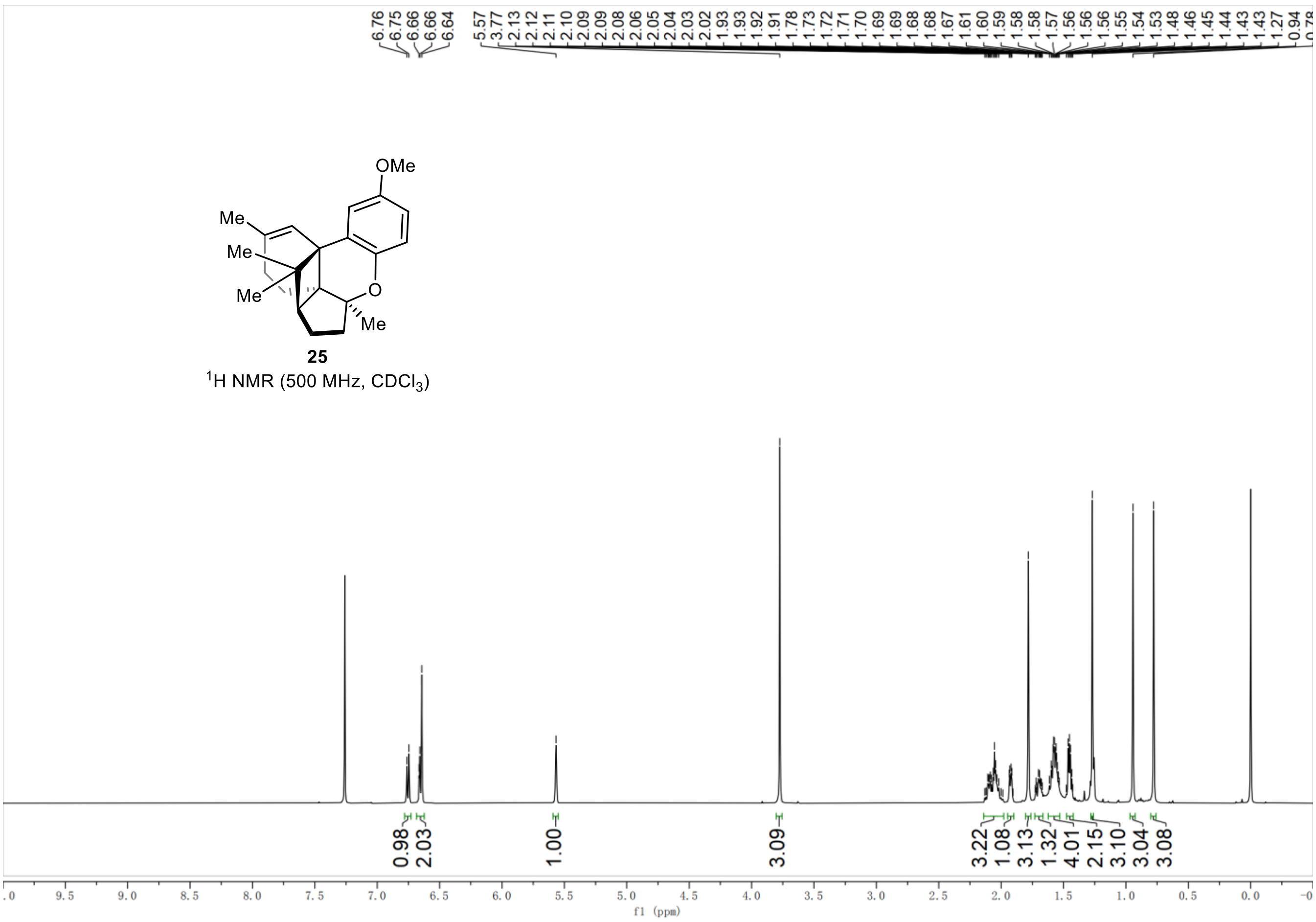
6.76
6.75
6.66
6.66
6.64
5.57
3.77
2.13
2.12
2.11
2.10
2.09
2.08
2.06
2.05
2.04
2.03
2.02
1.93
1.92
1.91
1.78
1.73
1.72
1.71
1.70
1.69
1.68
1.68
1.67
1.61
1.60
1.59
1.58
1.58
1.57
1.56
1.56
1.56
1.55
1.54
1.53
1.48
1.46
1.45
1.44
1.43
1.43
1.27
0.94
OMe
Me
Me
Me
O
Me
25
^{1}H NMR (500 MHz, CDCl$_3$)
0.98
2.03
1.00
3.09
3.22
1.08
3.13
1.32
4.01
2.15
3.10
3.04
3.08
f1 (ppm)

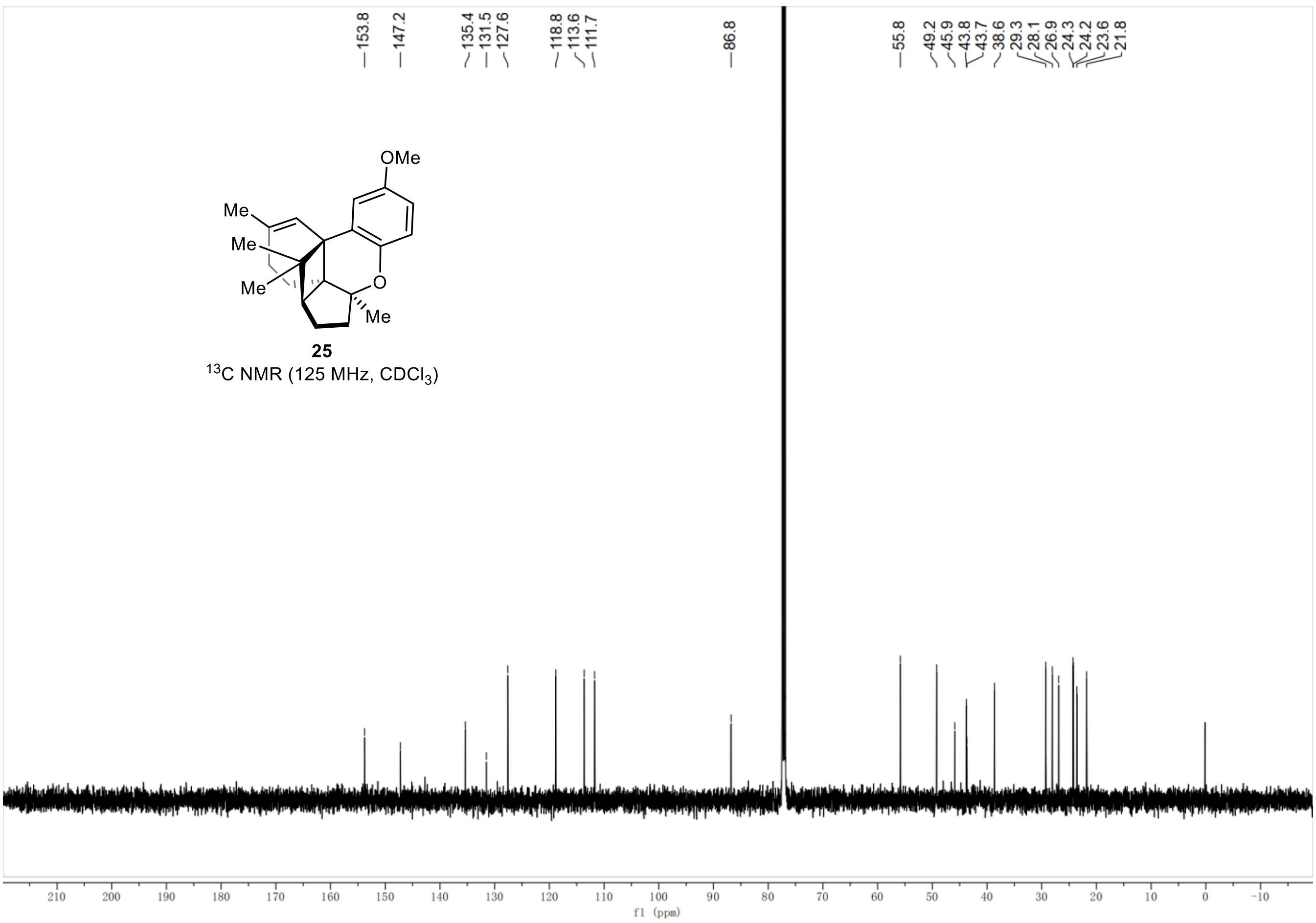

25

^{13}C NMR (125 MHz, $CDCl_3$)

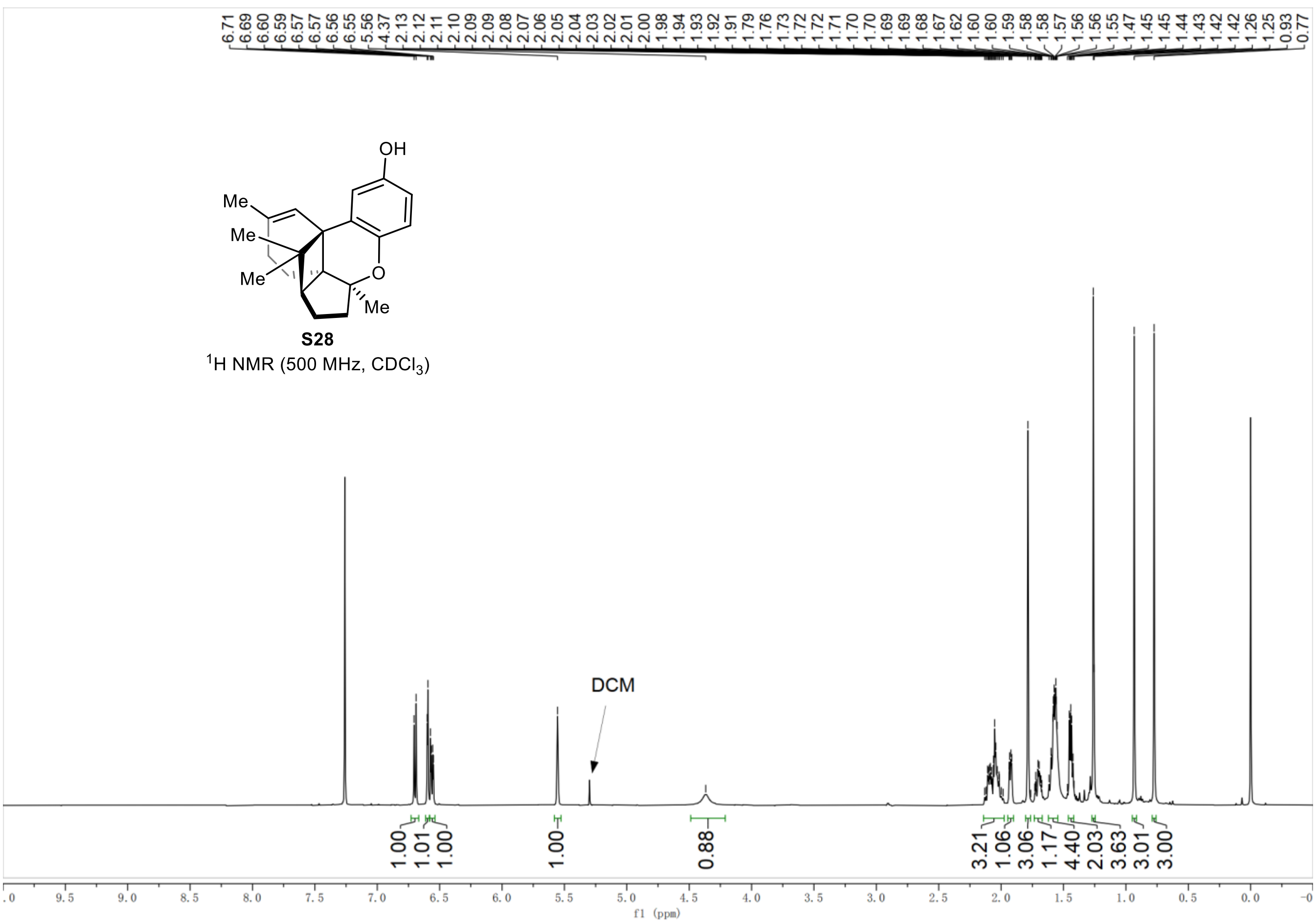

S28

^{1}H NMR (500 MHz, $CDCl_3$)

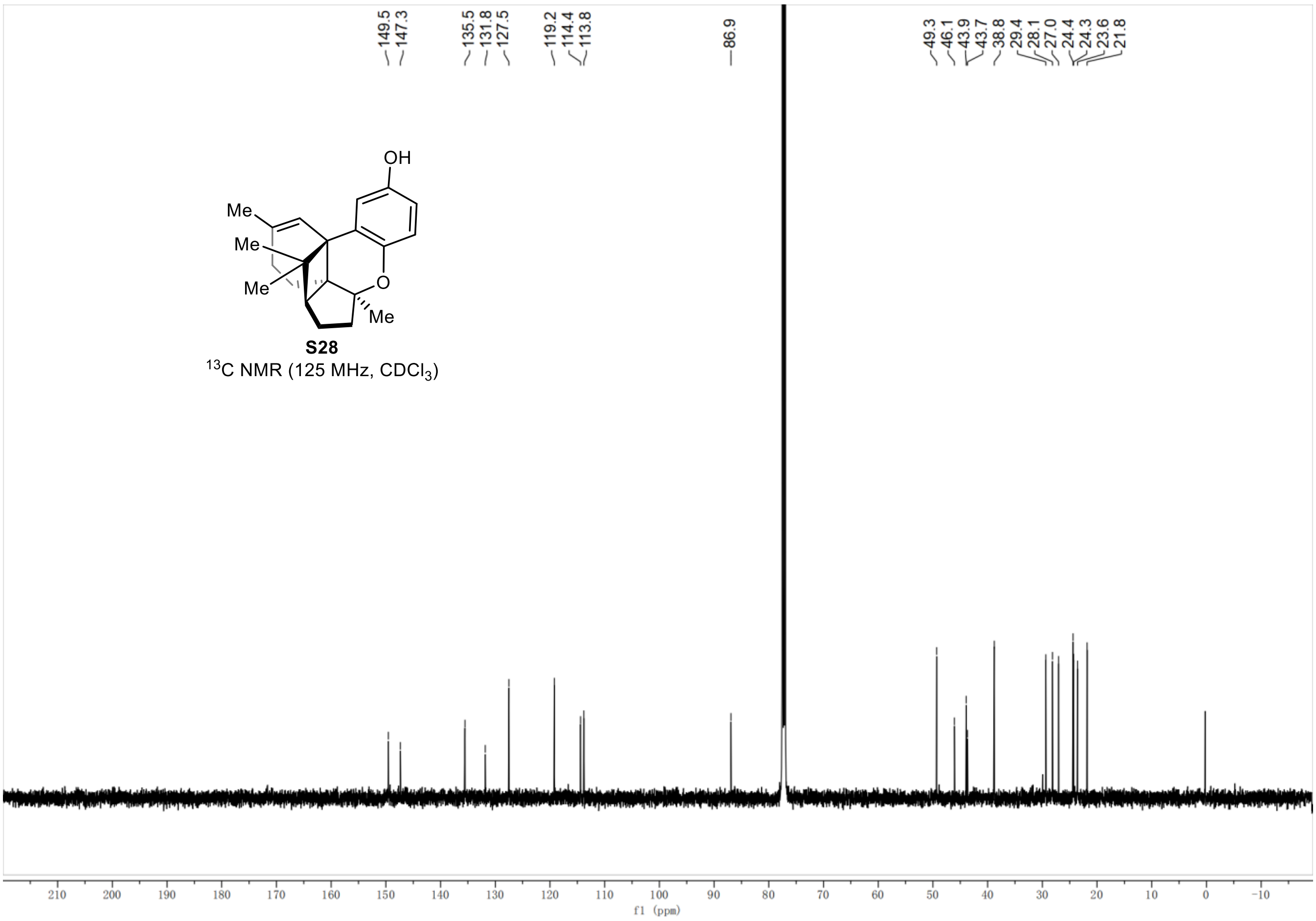

S28

^{13}C NMR (125 MHz, $CDCl_3$)

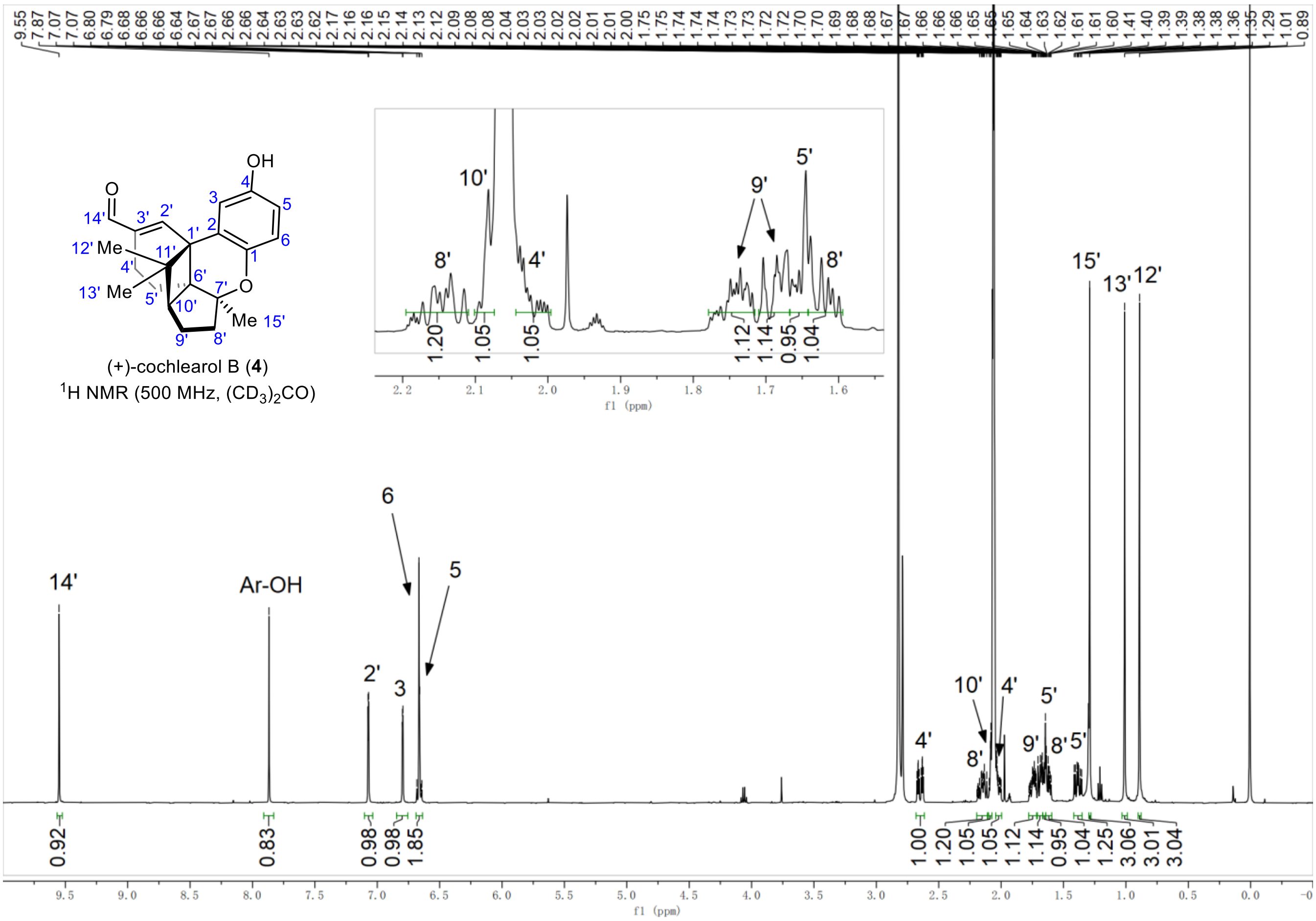

(+)-cochlearol B (**4**)

^{1}H NMR (500 MHz, $(CD_3)_2CO$)

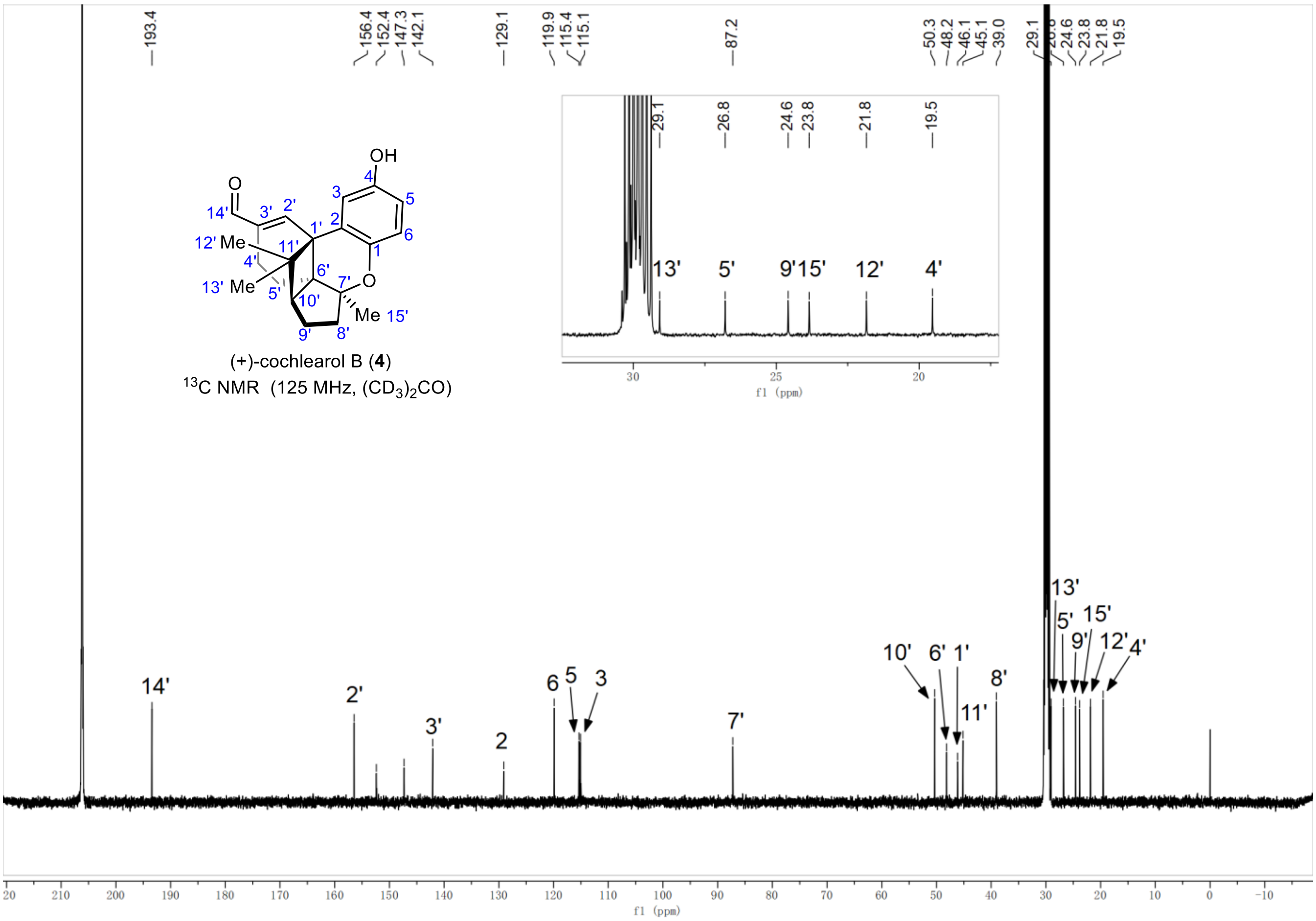
193.4
156.4
152.4
147.3
142.1
129.1
119.9
115.4
115.1
87.2
50.3
48.2
46.1
45.1
39.0
29.1
26.8
24.6
23.8
21.8
19.5
(+)-cochlearol B (4)
^{13}C NMR (125 MHz, $(CD_3)_2CO$)
f1 (ppm)

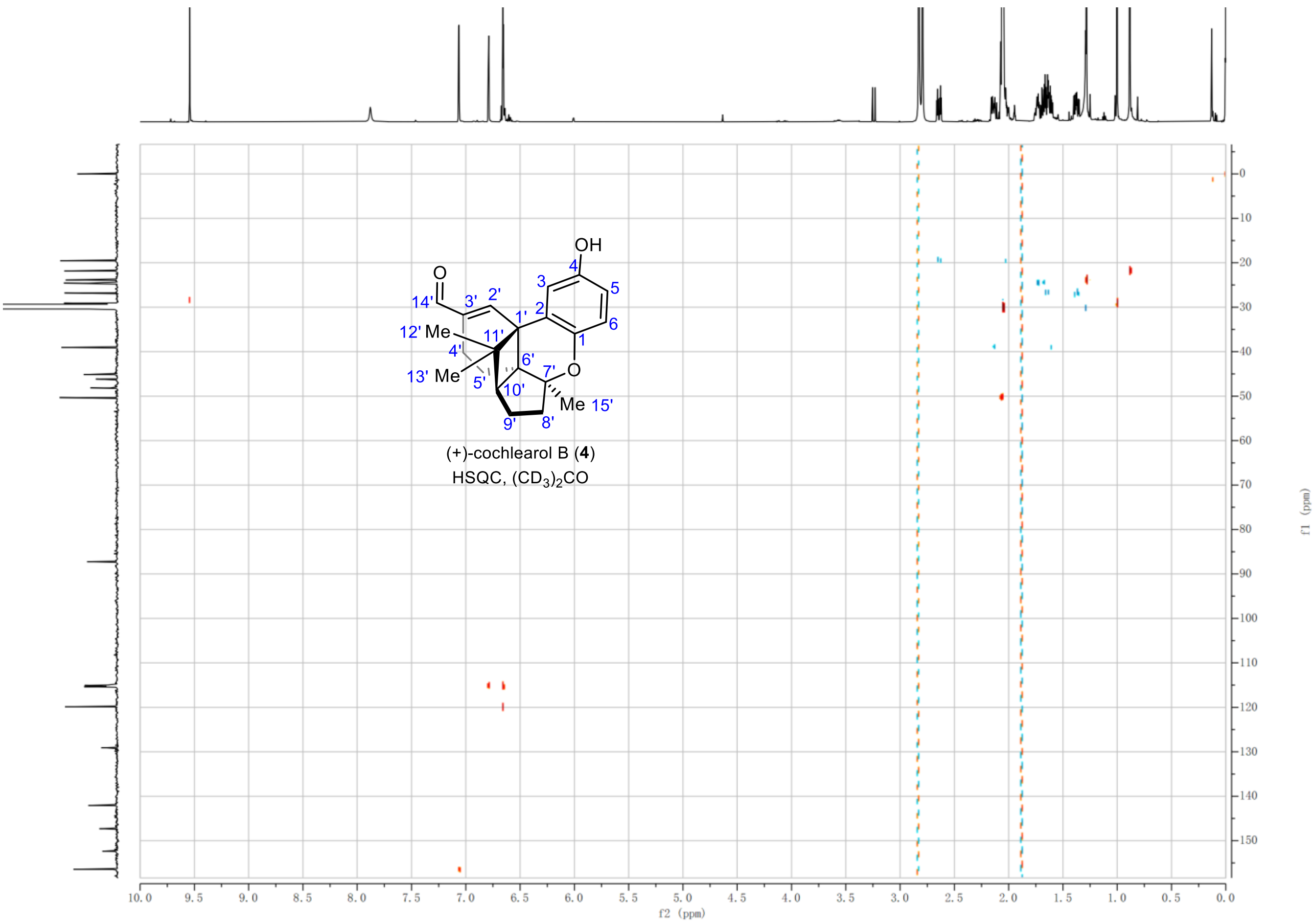
(+)-cochlearol B (4)
HSQC, $(CD_3)_2CO$
f2 (ppm)
f1 (ppm)

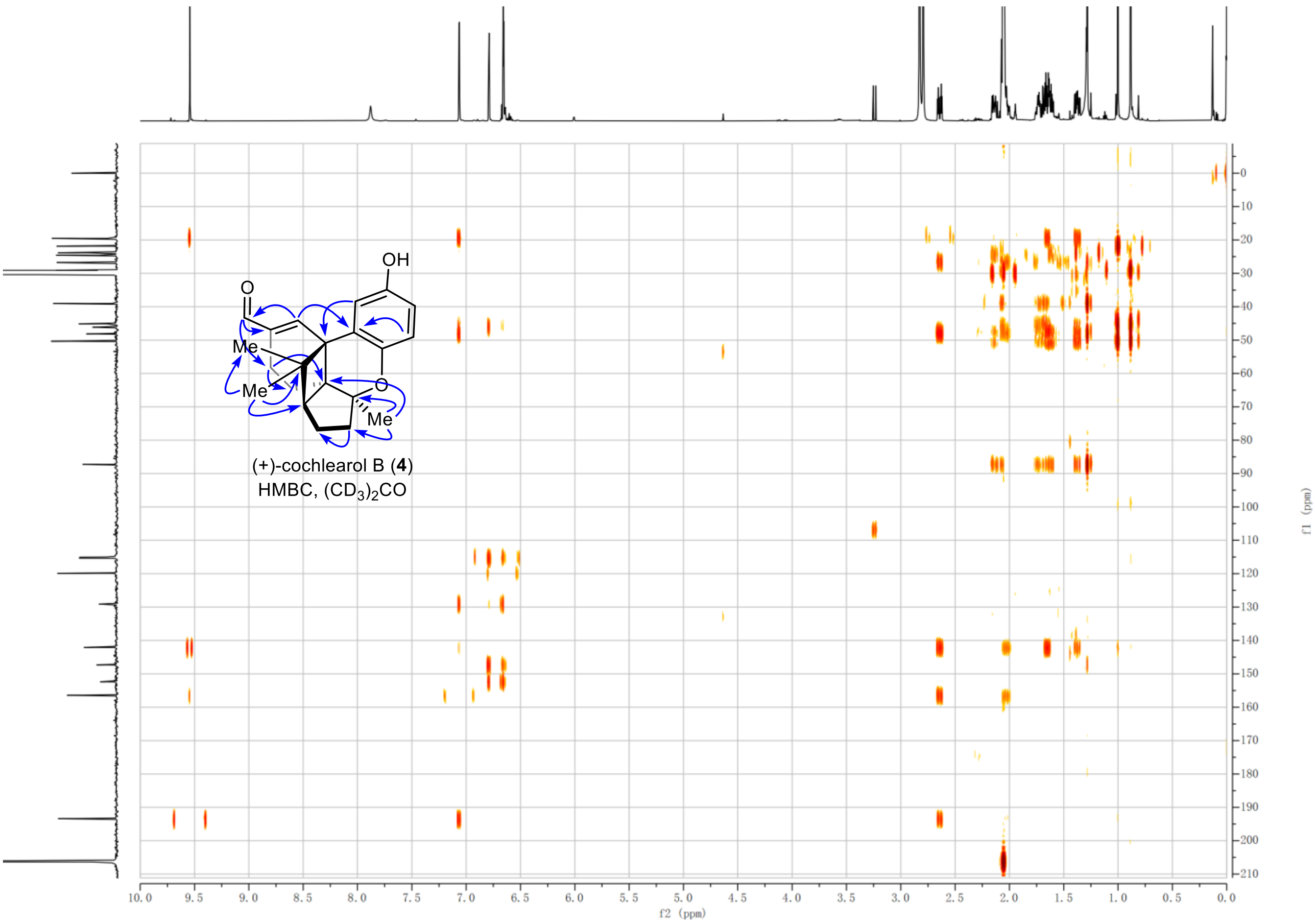
OH
O
Me
Me
O
Me
(+)-cochlearol B (4)
HMBC, $(CD_3)_2CO$
f1 (ppm)
f2 (ppm)

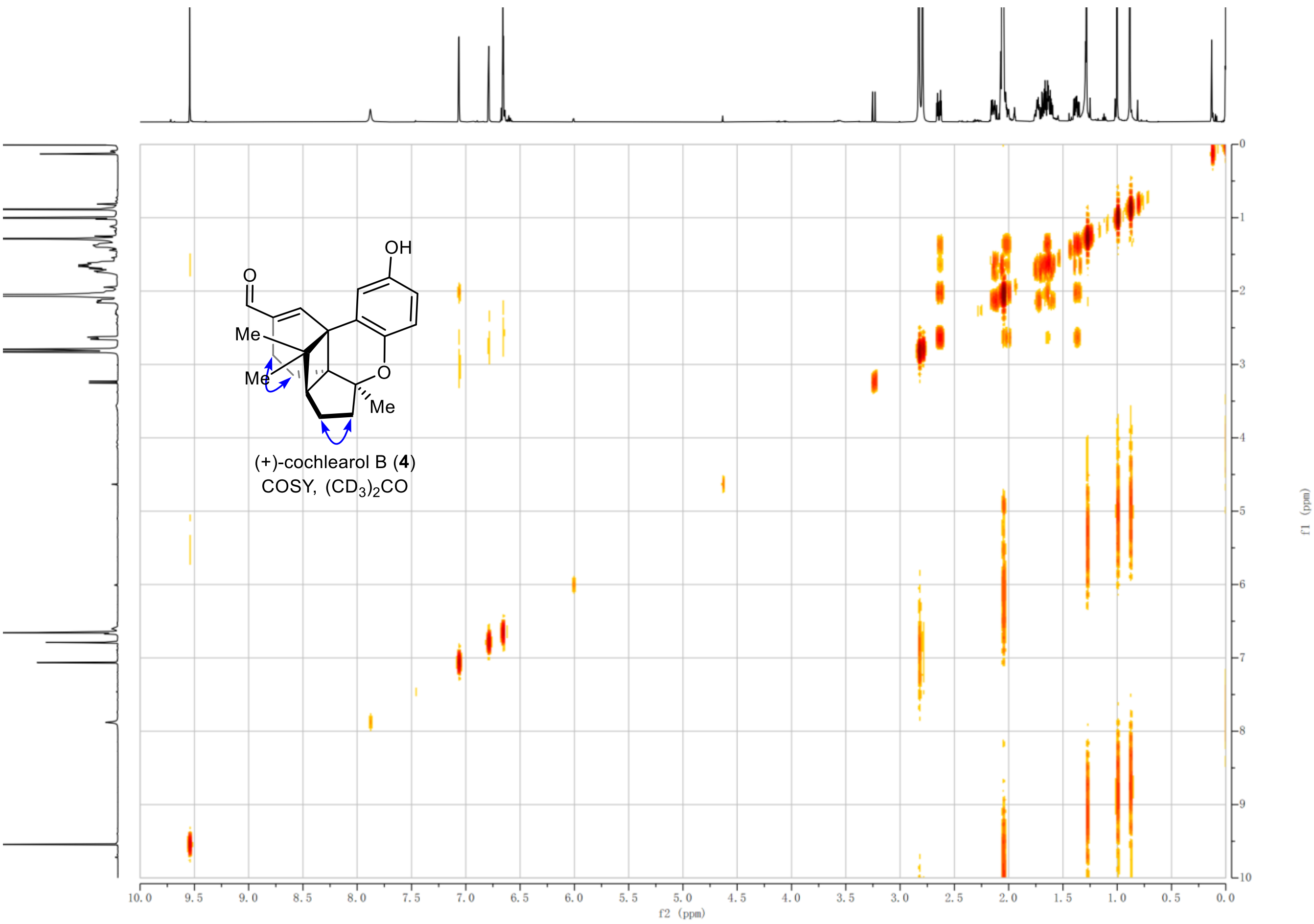
OH
O
Me
Me
O
Me
(+)-cochlearol B (4)
COSY, $(CD_3)_2CO$
f1 (ppm)
f2 (ppm)

8. Supplementary References

(1) Liu, Y., Zhou, C.-J., Li, Q. & Wang, H. Total synthesis of (±)-ganocins B and C. *Org. Biomol. Chem.* **14,** 10362−10365 (2016).

(2) Shao, H., Gao, X., Wang, Z.-T., Gao, Z. & Zhao, Y.-M. Divergent biomimetic total syntheses of ganocins A–C, ganocochlearins C and D, and cochlearol T. *Angew. Chem. Int. Ed.* **59,** 7419−7424 (2020).

(3) Mashiko, T. et al. Total synthesis of cochlearol B via intramolecular [2+2] photocycloaddition. *Angew. Chem. Int. Ed.* **60,** 24484−24487 (2021).

(4) Gao, Z. et al. A unified and bio-inspired total syntheses of cochlearol B and ganocins A-C via a cyclobutane as overbred intermediate. Preprint at https://doi.org/10.26434/chemrxiv-2022-xxmgl (2022).

(5) Wang, Z. et al. Bioinspired synthesis of cochlearol B and ganocin A. *Chin. Chem. Lett.* **35,** 109247 (2024).

(6) Zhou, Q. et al. Total synthesis of *Ganoderma* meroterpenoids cochlearol B and its congeners driven by structural similarity and biological homology. *Chem. Eur. J.* **30,** e202400084 (2024).

(7) Richardson, A. D., Vogel, T. R., Traficante, E. F., Glover, K. J. & Schindler, C. S. Total synthesis of (+)-cochlearol B by an approach based on a catellani reaction and visible-light-enabled [2+2] cycloaddition. *Angew. Chem. Int. Ed.* **61,** e2022012 (2022).

(8) Mashiko, T. et al. Enantioselective total syntheses of (+)-ganocin A and (–)-cochlearol B. *Org. Lett.* **25,** 8382−8386 (2023).

(9) Wang, Y.-Q., Xu, K., Min, L. & Li, C.-C. Asymmetric total syntheses of hypoestin A, albolic acid, and ceroplastol II. *J. Am. Chem. Soc.* **144**, 10162−10167 (2022).

(10) Liu, L. et al. Total synthesis of (–)-8-*epi*-chromazonarol enabled by a unique $N_2H_4 \cdot H_2O$ promoted intramolecular oxa-Michael cyclization reaction. *Org. Chem. Front.* **5**, 3013−3017 (2018).

(11) Wong, M. L. J., Sterling, A. J., Mousseau, J. J., Duarte, F. & Anderson, E. A. Direct catalytic asymmetric synthesis of *α*-chiral bicyclo[1.1.1]pentanes. *Nat. Commun.* **12**, 1644 (2021).

(12) Palomo, C. et al. Water-compatible iminium activation: organocatalytic Michael reactions of carbon-centered nucleophiles with enals. *Angew. Chem. Int. Ed.* **46**, 8431−8435 (2007).

(13) Epplin, R. C. et al. [2]-Ladderanes as isosteres for *meta*-substituted aromatic rings and rigidified cyclohexanes. *Nat. Commun.* **13**, 6056 (2022).

(14) Shi, D. et al. Low trap-state density and long carrier diffusion in organolead trihalide perovskite single crystals. *Science* **347**, 519−522 (2015).

(15) Peng, X.-R. et al. Four new polycyclic meroterpenoids from *Ganoderma cochlear*. *Org. Lett.* **16**, 5262−5265 (2014).

(16) Dou, M. et al. Cochlearols A and B, polycyclic meroterpenoids from the fungus *Ganoderma cochlear* that have renoprotective activities. *Org. Lett.* **16**, 6064−6067 (2014).
