# Supplementary material for: Divergent asymmetric synthesis of (–)-ganocins A–C and (+)-cochlearol B via a non-biomimetic strategy
Source: Nat Commun. 2026 Oct 15;17:11650. doi: 10.1038/s41467-026-78287-1 (PMC13759510; doi:10.1038/s41467-026-78287-1)
Supplement: Supplementary file 2 [file 41467_2026_78287_MOESM2_ESM.pdf]

nature portfolio

Peer Review File

Divergent Asymmetric Synthesis of (–)-Ganocins A–C and (+)-Cochlearol B via a Non-biomimetic Strategy

Corresponding Author: Professor Zhiqiang Ma

This file contains all reviewer reports in order by version, followed by all author rebuttals in order by version.

Version 0:

Reviewer comments:

Reviewer #1

(Remarks to the Author)
Summary:
The study by Xu and co-workers report asymmetric syntheses of (-)-ganocins A-C and (+)-cochlearol B. The crux of the study is the preparation of a versatile intermediate (12 in the paper) which can be diverted to access the different scaffold of the natural products. In turn, compound 12 was accessed in a relatively short sequence from (R)-limonene (chiral pool) and commercial 2-bromo-4-methoxyphenol. Some transformations are notable: (1) a sequential organocatalytic oxo-michael-Michael reaction to construct 12; (2) the differentiated acid-mediated transformations to guide 12 to intermediates 10, 20, 21; (3) The SmI2-mediated ketone-none cross-coupling to construct the cyclobutane-system of cochlearol.

Assessment:
Overall, this is a nice piece of work and the authors have clearly provided an improved route to access these natural products. The results, however, appears primarily of interest to specialists within the specific class of molecules which has already seen substantial interest from other groups. To match with the scope and intended impact of Nature Commun, the study would, in my opinion, need to demonstrate broader implications (broader conceptual advance, new method, biological insights/tools) beyond the immediate synthetic target(s). For this reason, while technically strong and carefully executed, the study, in its current form, is better suited for a specialised journal.

Reviewer #2

(Remarks to the Author)
In this manuscript, Ma and co-workers report the first divergent asymmetric total syntheses of Ganocins A–B and Cochlearol B, utilizing (R)-limonene as the chiral pool. The authors' key achievements include the intramolecular oxa-Michael reaction to construct the common chromanone unit present in the target molecules; the intermolecular Michael addition employing methyl vinyl ketone (MVK) as the acceptor to access a common synthetic intermediate; intramolecular aldol or aldol/etherification cascade reactions to assemble the cyclohexenone or oxa-6-5 bicyclic frameworks, respectively; and a samarium(II) diiodide-promoted intramolecular alkoxyl radical cyclization to form the highly substituted cyclobutane unit in cochlearol B. Both the manuscript and the supporting information are well organized and clearly presented, which is commendable. However, considering prior synthetic reports on these target molecules—encompassing both racemic and asymmetric approaches—this reviewer finds it difficult to conclude that the current work demonstrates clear superiority in terms of synthetic strategy novelty or overall synthetic efficiency. In fact, some of the key reactions, such as the MVK-involved intermolecular Michael addition and intramolecular aldol reactions, bear a strong resemblance to previously reported procedures, which notably diminishes the novelty of this study. Moreover, the yields and selectivities of several crucial reactions throughout the synthetic sequence are suboptimal, resulting in only moderate overall efficiency.
In summary, while the synthesis is structurally comprehensive and rich in detail, this reviewer considers the novelty insufficient and questions the suitability of this work for publication in a top-tier journal such as Nature Commun.

Reviewer #3

(Remarks to the Author)
In this report, the authors present an asymmetric, divergent total synthesis of four Ganoderma meroterpenoids from a

common intermediate, which is accessed from a chiral starting material. The synthetic route features several key transformations, including a diastereoselective oxa-Michael addition, a condition-controlled divergent aldol/etherification cascade, and an SmI_2-mediated radical cyclization to generate a highly strained cyclobutane ring.
Overall, the chemistry is elegant and interesting. The authors clearly demonstrate their efforts through extensive optimization studies and exploration of synthetic detours.

Comments on the manuscript
1. Line 24: "…displays biological activities, such as antitumor, …, and antimicrobial."
The phrasing is awkward and should be revised for clarity.
2. Line 30: p-Smads
It would be helpful to briefly define this abbreviation or provide a short explanation.
3. Line 233 (Methods): (rt, approximately 33 °C)
This appears inconsistent, as room temperature is typically not 33 °C. Please clarify.

Comments on the Supporting Information (SI)
1. Page S3 (Note for compound 16):
Correct a word separation error.
2. Page S5 (optimization table, and similar tables):
Instead of unclear descriptions, consider using "complex mixture" for clarity.
3. Page S8 (procedure for 19):
Use "59 h" instead of "59.0 h" for consistency.
4. Tables in general (see S9 as an example):
Temperature ranges such as "rt–60 °C" or "0 °C–rt" are inconsistently formatted. Consider replacing "rt" with a defined value (e.g., 23 °C) or standardizing notation throughout.
5. General wording in procedures (see S10):
Replace phrases like "the reaction was added a solution of…" with "a solution of … was added to the reaction mixture."
6. General phrasing (see S10):
Replace "after stirred" with "after stirring."
7. Ganocin A:
In compound S7, the phenolic OH is included in the characterization but omitted in the natural product depiction, leading to an incorrect proton count.
Additionally, although the ^{1}H NMR data match previous literature, inclusion of 2D NMR spectra would be beneficial—especially where signals overlap with water and affect integration.
8. Ganocin B:
The signal at 7.20 ppm (dd, J = 8.7, 2.9 Hz) is reported as 1H, but appears integrated as 2H in the spectrum. Additionally, signals at 6.93 and 6.92 ppm are listed separately in the data but appear combined in the spectrum. Clarification is needed, and 2D NMR data would again be helpful.
9. Compound 23:
No 2D NMR data are provided.
10. Compound 27:
Reported as a red oil, yet X-ray data are provided. Please clarify if this is correct.
11. Compounds S18 and S19:
Signals overlap with water; inclusion of 2D NMR spectra would strengthen the assignments.
12. Compound 25:
HRMS data are missing.
13. Cochlearol B:
No 2D NMR data are provided. The signal at 2.10–2.07 ppm overlaps with water, making assignment unclear.
After addressing these minor revisions, the manuscript will be suitable for publication. The chemistry and overall approach are innovative and distinct from previous syntheses. In my opinion, this work is a valuable contribution and merits publication.

Version 1:

Reviewer comments:

Reviewer #3

(Remarks to the Author)
Everything good now. Thanks for the revisions.

Open Access This Peer Review File is licensed under a Creative Commons Attribution 4.0 International License, which permits use, sharing, adaptation, distribution and reproduction in any medium or format, as long as you give appropriate credit to the original author(s) and the source, provide a link to the Creative Commons license, and indicate if changes were made.
In cases where reviewers are anonymous, credit should be given to 'Anonymous Referee' and the source.
The images or other third party material in this Peer Review File are included in the article's Creative Commons license, unless indicated otherwise in a credit line to the material. If material is not included in the article's Creative Commons license and your intended use is not permitted by statutory regulation or exceeds the permitted use, you will need to obtain permission directly from the copyright holder.
To view a copy of this license, visit https://creativecommons.org/licenses/by/4.0/

Zhiqiang Ma, Ph.D.
Key Lab of Functional Molecular Engineering of Guangdong Province
School of Chemistry & Chemical Engineering, South China University of Technology
Wushan Road-381, Guangzhou 510641, People's Republic of China
Phone: 020-87111098; Email: cezqma@scut.edu.cn

Title: Divergent Asymmetric Synthesis of (–)-Ganocins A–C and (+)-Cochlearol B

Manuscript ID: NCOMMS-26-008186

Dear Reviewers,

We sincerely thank you for your valuable time and constructive comments on our manuscript. We have revised the manuscript accordingly, and the changes are highlighted in yellow. The point-by-point response to the comments is as follows:

Responses to Reviewer 1:

The study by Xu and co-workers report asymmetric syntheses of (–)-ganocins A–C and (+)-cochlearol B. The crux of the study is the preparation of a versatile intermediate (**12** in the paper) which can be diverted to access the different scaffold of the natural products. In turn, compound **12** was accessed in a relatively short sequence from (*R*)-limonene (chiral pool) and commercial 2-bromo-4-methoxyphenol. Some transformations are notable: (1) a sequential organocatalytic *oxo*-michael-Michael reaction to construct **12**; (2) the differentiated acid-mediated transformations to guide **12** to intermediates **10**, **20**, **21**; (3) The SmI_2-mediated ketone-none cross-coupling to construct the cyclobutane-system of cochlearol.

Overall, this is a nice piece of work and the authors have clearly provided an improved route to access these natural products. The results, however, appears primarily of interest to specialists within the specific class of molecules which has already seen substantial interest from other groups. To match with the scope and intended impact of *Nature Commun*, the study would, in my opinion, need to demonstrate broader implications (broader conceptual advance, new method, biological insights/tools) beyond the immediate synthetic target(s). For this reason, while technically strong and carefully executed, the study, in its current form, is better suited for a specialised journal.

◆ We sincerely appreciate the reviewer's constructive comments and fully agree that the broader implications of our work should be further elaborated. In response, we have incorporated the following two aspects into the revised manuscript.

(1) Value of skeletally diverse natural products in drug discovery: Natural products have historically served as a vital source for the discovery of novel therapeutic agents. Between January 2014 and June 2025, a total of 58 natural product-related drugs received approval globally, amply demonstrating their central role in modern drug development. The three-dimensional structures of natural products enable an exceptional degree of complementarity with target proteins through a sophisticated interplay of precise structural recognition and modulation of protein conformation. This relies on a multimodal combination of hydrogen bonds, hydrophobic contacts, electrostatic interactions, and van der Waals interactions,

collectively ensuring high affinity and specificity. Furthermore, skeletal diversity primarily determines the three-dimensional shape diversity of a compound library. Consequently, the rapid and efficient construction of a library with diverse molecular skeletons substantially increases the odds of drug discovery compared with a single-scaffold library.

(2) Non-biomimetic strategy: In the biogenetic hypothesis for ganocins A–C and cochlearol B, the racemic chromane **6**, which is generated via a HDA reaction, define the branch point of biosynthesis (see Fig. 1B in the manuscript). For the synthesis of multiple congeners within a natural product family, the most effective approach generally relies on a versatile late-stage intermediate, whose diversification enables access to the entire collection. In this context, mimicking biosynthetic pathways has proven particularly effective for constructing complex natural products. To date, eight elegant total syntheses have been reported, most of which focus on individual natural products; six of these are racemic, and the majority are biomimetic. In the current biomimetic syntheses of ganocins A–C and cochlearol B, the key chromane moieties were typically constructed via a biomimetic HDA reaction. Since this HDA reaction inherently generates racemic intermediates, the biomimetic chemical synthesis ultimately affords the target natural products as racemates. The scarcity of asymmetric and divergent synthetic approaches has substantially hindered rigorous biological evaluation of these natural products. To overcome this long-standing challenge, the identification of a non-biomimetic intermediate for asymmetric synthesis is required.

Given the importance of rapidly accessing skeletally diverse natural products and the non-biomimetic strategy employed, we have revised the Title and expanded the Abstract and Introduction as follows (with newly added or revised portions highlighted in yellow):

Title: "*Divergent asymmetric synthesis of (–)-ganocins A–C and (+)-cochlearol B via a non-biomimetic strategy*"

Abstract: "*Natural products are of substantial value in drug discovery screening campaigns. Their three-dimensional structures exhibit high complementarity with target proteins, via a multimodal combination of hydrogen bonds, hydrophobic contacts, electrostatic interactions, and van der Waals interactions. Skeletal diversity directly determines the three-dimensional shape diversity of compound libraries. Therefore, the rapid access to distinct molecular skeletons significantly increases the odds of drug discovery. Ganocins A–C and cochlearol B, Ganoderma meroterpenoids, feature distinct skeletons and diverse bioactivities. Despite tremendous efforts by synthetic chemists, a unified asymmetric synthetic strategy towards all four members has remained elusive. Here, we report the first unified, asymmetric total synthesis of (–)-ganocins A–C and (+)-cochlearol B via a non-biomimetic strategy, featuring diastereoselective oxa-Michael addition, acid-controlled divergent cascade aldol/etherification cyclization or aldol/dehydration, and SmI_2-mediated 4-exo-trig reductive cross-coupling. This meticulously designed synthetic strategy establishes a general solution to a problem previously deemed intractable by biomimetic approaches.*"

Introduction: "*Natural products have long been invaluable sources for the discovery of novel therapeutic drugs*[1,2]*. Fifty-eight natural product-related drugs were approved globally from January 2014 to June 2025*[3]*. The three-dimensional structures of natural products*

enable an exceptional degree of complementarity with target proteins through a sophisticated interplay of precise structural recognition and modulation of protein conformation[4,5]*. This relies on a multimodal combination of hydrogen bonds, hydrophobic contacts, electrostatic interactions, and van der Waals interactions, ensuring high affinity and specificity. Skeletal diversity primarily determines the three-dimensional shape diversity of a compound library*[6,7]*. Thus, the rapid and efficient construction of distinct molecular skeletons substantially increases the odds of drug discovery compared with a single-scaffold library.*

Ganoderma, a medicinal fungus used for centuries to treat various diseases, has been studied extensively in China, Korea, Japan, and the United States of America[8]*. To date, a variety of natural products, including meroterpenoids, triterpenoids, polysaccharides, sesquiterpenoids, steroids and alkaloids, have been isolated from the genus Ganoderma*[9]*. Among these, Ganoderma meroterpenoids are structurally characterized by a unique 1,2,4-trisubstituted phenyl group and the unsaturated terpene subunit, exhibiting diverse biological activities, such as antitumor, antifibrotic, antiallergic, antioxidant, and antimicrobial activities*[10–13]*. Their distinct molecular skeletons and diverse biological activities have attracted considerable attention from both pharmacological and synthetic communities. Ganocins A–C (***1−3***) are three unprecedented Ganoderma meroterpenoids that were isolated from the fruiting bodies of Ganoderma cochlear in racemic form by Qiu and co-workers in 2014 (Fig. 1A)*[14]*. In the same year, cochlearol B (***4***) was also isolated from Ganoderma cochlear in racemic form by Cheng and co-workers*[15]*. Notably, the enantiomers (+)- and (–)-cochlearol B (***4*** and ent-***4***), obtained via chiral HPLC separation, exhibited significantly distinct inhibitory effects against phosphorylated Smad2 and Smad3 (p-Smads). Therefore, developing asymmetric synthetic strategies for this class of Ganoderma meroterpenoids is of critical importance.*

*Structurally, ganocins A–C (***1−3***) and cochlearol B (***4***) share a unique 5/6/6/6 tetracyclic scaffold incorporating a spiro[4.5]decane moiety (Fig. 1A). Specifically, ganocin A (***1***) features a heptasubstituted tetrahydrofuran ring (highlighted in red) and four contiguous stereocenters, including three quaternary stereocenters—two embedded within the tetrahydrofuran ring. Cochlearol B (***4***) is characterized by a gem-dimethylcyclobutane core (highlighted in red) bearing two contiguous all-carbon quaternary stereocenters.*

*Biosynthetically, Qiu et al. proposed that (±)-ganocins A–C (***1−3***) are derived from fornicin C (***5***)*[14]* (Fig. 1B). In their pathway, a hetero-Diels−Alder (HDA) reaction of* **5** *initially affords racemic chromane* **6***. Subsequent radical cyclization yields (±)-ganocin A (***1***). Alternatively, dehydration and decarboxylation of intermediate* **6** *lead to enone* **7***, whose radical cyclization furnishes both (±)-ganocins B (***2***) and C (***3***). Meanwhile, on the basis of structural similarities between (±)-ganocins A–C (***1−3***) and (±)-cochlearol B (***4***), an alternative biosynthetic pathway has been proposed by Hao's group*[16]* and by Li and Tang's group*[17]*. Their hypothesis begins with an intramolecular [2+2] photocycloaddition of chromene* **8** *or* **9,** *affording (±)-cochlearol B (***4***). Subsequent oxidative cleavage of the cyclobutane ring or epoxide rearrangement directly affords (±)-ganocin A (***1***). This intermediate then undergoes sequential decarboxylation, β-elimination, and dehydration to furnish (±)-ganocins B (***2***) and C (***3***). In the biogenetic hypothesis for ganocins A–C and cochlearol B, the racemic chromane* **6***, which is generated via a HDA reaction, define the branch point of biosynthesis. When multiple*

congeners of a natural product family are targeted for chemical synthesis, the most efficient strategy is typically the preparation of a versatile late-stage intermediate, whose diversification enables access to the entire collection. In this context, mimicking biosynthetic pathways has proven to be an effective strategy for constructing complex natural products.

To date, eight elegant total syntheses have been reported[16–23]*, most of which focus on individual natural products; six of these are racemic, and the majority are biomimetic (see Supplementary Figure 1 for details). In the current biomimetic syntheses of ganocins A–C and cochlearol B, the key chromane moieties were typically constructed via a biomimetic HDA reaction. Since this HDA reaction inherently generates racemic intermediates, the biomimetic chemical synthesis ultimately affords the target natural products as racemates. The scarcity of asymmetric and divergent synthetic approaches has substantially hindered rigorous biological evaluation of these natural products. To overcome this long-standing challenge, the identification of a non-biomimetic intermediate for asymmetric synthesis is required. Herein, we report that chiral chromanone* ***12****—a versatile non-biomimetic intermediate—serves as a branch point in our chemical synthesis (Fig. 1C). Leveraging this intermediate, we have accomplished the first divergent asymmetric total synthesis of (–)-ganocins A–C (****1−3****) and (+)-cochlearol B (****4****) from (R)-limonene in 11, 8, 8, and 15 steps, respectively. Notably, the 11-step synthesis of (–)-ganocin A (****1****) represents the shortest asymmetric synthetic route to date—a significant reduction from the previously reported 17-step route. Furthermore, the present work constitutes the first asymmetric total synthesis of (–)-ganocins B and C (****2*** *and* ***3****). This streamlined synthetic platform provides a foundation for further rigorous biological evaluation.*"

We believe these revisions significantly broaden the implications of our work and will be of great interest to the broad readership of *Nature Communications*.

Responses to Reviewer 2:

In this manuscript, Ma and co-workers report the first divergent asymmetric total syntheses of Ganocins A–B and Cochlearol B, utilizing (*R*)-limonene as the chiral pool. The authors' key achievements include the intramolecular *oxa*-Michael reaction to construct the common chromanone unit present in the target molecules; the intermolecular Michael addition employing methyl vinyl ketone (MVK) as the acceptor to access a common synthetic intermediate; intramolecular aldol or aldol/etherification cascade reactions to assemble the cyclohexenone or *oxa*-6-5 bicyclic frameworks, respectively; and a samarium(II) diiodide-promoted intramolecular alkoxyl radical cyclization to form the highly substituted cyclobutane unit in cochlearol B. Both the manuscript and the supporting information are well organized and clearly presented, which is commendable. However, considering prior synthetic reports on these target molecules—encompassing both racemic and asymmetric approaches—this reviewer finds it difficult to conclude that the current work demonstrates clear superiority in terms of synthetic strategy novelty or overall synthetic efficiency. In fact, some of the key reactions, such as the MVK-involved intermolecular Michael addition and intramolecular aldol reactions, bear a strong resemblance to previously reported procedures, which notably diminishes the novelty of this study. Moreover, the yields and selectivities of

several crucial reactions throughout the synthetic sequence are suboptimal, resulting in only moderate overall efficiency.

In summary, while the synthesis is structurally comprehensive and rich in detail, this reviewer considers the novelty insufficient and questions the suitability of this work for publication in a top-tier journal such as *Nature Commun*.

◆We sincerely appreciate the reviewer' s thoughtful evaluation of our work, and would like to take this opportunity to further clarify the novelty and efficiency of our work. To facilitate clearer comparison, we have added a comparative summary of prior synthetic reports and our work in Supplementary Figure 1 of the Supplementary Information.

Groups	stereochemical outcome	ganocin A (**1**)	ganocin B (**2**)	ganocin C (**3**)	cochlearol B (**4**)	chromane/chromene	tetrahydrofuran ring	cyclobutane ring
Wang (2016)	*racemic*	–	11 steps (6%)	11 steps (6%)	–	gold-catalyzed cascade cyclization	–	–
Zhao (2020)	*racemic*	13 steps (0.3%)	9 steps (2%)	9 steps (2%)	–	hetero-Diels–Alder	*oxa*-Michael addition	–
Sugita (2021)	*racemic*	–	–	–	16 steps (9%)	oxidative cyclization	–	All five research groups employed [2+2] photo-cycloaddition to synthesize cyclobutane.
Hao (2022)	*racemic*	10 steps (2%)	12 steps (0.3%)	12 steps (0.7%)	8 steps (4%)	hetero-Diels–Alder	oxidative cleavage of cyclobutane ring	
Li/Tang (2024)	*racemic*	8 steps (7%)	–	–	7 steps (11%)	hetero-Diels–Alder	oxidative cleavage of cyclobutane ring	
Zhao (2024)	*racemic*	–	–	–	11 steps (7%)	Pd-catalyzed cascade reaction	–	
Schindler (2022)	*asymmetric (chiral resolution)*	–	–	–	14 steps (0.9%)	Kabbe condensation	–	
Sugita (2023)	*asymmetric*	17 steps (9%)	–	–	16 steps (9%)	oxidative cyclization	oxidative cyclization (create a ring in a step)	
this work	*asymmetric*	11 steps (11%) shortest asymmetric total synthesis	8 steps (13%) first asymmetric total synthesis	8 steps (22%)	15 steps (1.5%)	*amino acid* catalyzed *oxa*-Michael addition	cascade aldol/ etherification cyclization (create two ring in a step)	SmI_2-mediated carbonyl–alkene coupling

(1) Synthetic strategy novelty

As shown in the figure above, the prior eight syntheses, reported by six independent groups (Wang, Zhao, Sugita, Hao, Li/Tang, and Schindler), mainly targeted individual members; six of these are racemic, and the majority are biomimetic. In the current biomimetic syntheses of ganocins A–C and cochlearol B, the key chromane moieties are typically constructed via a biomimetic hetero-Diels–Alder (HDA) reaction. Since this HDA reaction inherently generates racemic intermediates, and the subsequent transformations necessarily proceed via a [2+2] photocycloaddition, these biomimetic syntheses ultimately afford the target natural products as racemates. The scarcity of asymmetric and divergent synthetic approaches has substantially hindered rigorous biological evaluation of these natural products. To overcome this long-standing challenge, the identification of a non-biomimetic intermediate for asymmetric synthesis is required. We adopted a non-biomimetic strategy and consequently obtained a distinctly different intermediate via a diastereoselective, amino acid-catalyzed *oxa*-Michael reaction, serving as a branch point in our chemical synthesis. This intermediate has enabled the rapid and asymmetric synthesis of all four family members.

In addition, as shown in the figure, previous syntheses mainly relied on biomimetic HAD reaction for constructing the racemic chromane/chromene core. In contrast, We employed a diastereoselective, amino acid-catalyzed *oxa*-Michael reaction to generate a chiral intermediate. For the assembly of the tetrahydrofuran ring, previous approaches typically constructed it via *oxa*-Michael addition, oxidative cleavage of cyclobutanes, or oxidative

cyclization—all of which require stepwise ring construction (typically 1 ring/step). Conversely, we developed an unprecedented aldol/etherification cascade cyclization that enables the formation of two rings in a single operation. For the construction of the cyclobutane ring, all previous syntheses have relied exclusively on biomimetic [2+2] photocycloaddition. In stark contrast, we adopted a SmI_2-mediated 4-*exo*-trig reductive cross-coupling reaction of ketone and cyclic enone motifs to rapidly forge the highly strained cyclobutane ring.

While the Michael addition and aldol reaction bear resemblance to previously reported, they are merely conventional transformations. We employed a non-biomimetic strategy and adopted innovative ring-system construction methods. Specifically, a diastereoselective, amino acid-catalyzed *oxa*-Michael reaction to construct the chromane core; an unprecedented aldol/etherification cascade cyclization to assemble the tetrahydrofuran ring; and a SmI_2-mediated 4-*exo*-trig reductive cross-coupling reaction to build the cyclobutane ring. As acknowledged by Reviewer 3: "*The synthetic route features several key transformations, including a diastereoselective oxa-Michael addition, a condition-controlled divergent aldol/etherification cascade, and an SmI_2-mediated radical cyclization to generate a highly strained cyclobutane ring.*"

Therefore, while most previous syntheses focused on single targets or delivered racemic products of all family members, we have accomplished the first unified asymmetric synthesis of all four family members via a non-biomimetic strategy, which clearly demonstrates the novelty of our approach.

(2) Overall synthetic efficiency

As shown in the figure above, although our synthesis of (+)-cochlearol B led to comparable step counts (our 15 steps vs. previous 14 or 16 steps) and a lower overall yield (our 1.5% vs. previous 0.9% or 9%) compared with the previous asymmetric synthesis, our syntheses of (–)-ganocins A–C provided significantly higher efficiency compared with all previous syntheses. (1) Our synthesis of (–)-ganocin A achieved a 6-step reduction (our 11 steps vs. previous 17 steps) and comparable overall yield (our 11% vs. previous 9%) compared with the previous asymmetric synthesis. (2) We achieved the first asymmetric total syntheses of (–)-ganocins B and C. Even compared with previous racemic routes, our asymmetric synthesis of (–)-ganocin B proceeded via shorter route (our 8 steps vs. previous 9, 11, or 12 steps) and afforded the highest overall yield (our 13% vs. previous 0.3%, 2%, or 6%). Likewise, our asymmetric synthesis of (–)-ganocin C was completed in shorter route (our 8 steps vs. previous 9, 11, or 12 steps) and afforded the highest overall yield (our 22% vs. previous 0.7%, 2%, or 6%).

Therefore, although our synthesis of (+)-cochlearol B showed room for improvement in efficiency, the superior step economy and overall yields achieved in the syntheses of (–)-ganocins A–C, representing the most efficient routes reported to date, unambiguously validate the overall efficiency advantage of our synthetic strategy.

In addition, during the revision, we have systematically optimized the Michael addition between chromanone **19** and MVK, including MVK addition mode, equivalents, and solvent,

and improved the yield to 58%. The detailed experimental data are included in Supplementary Table 4 of the Supplementary Information.

Supplementary Table 4. Optimization of the Michael addition of chromanone 19 with MVK

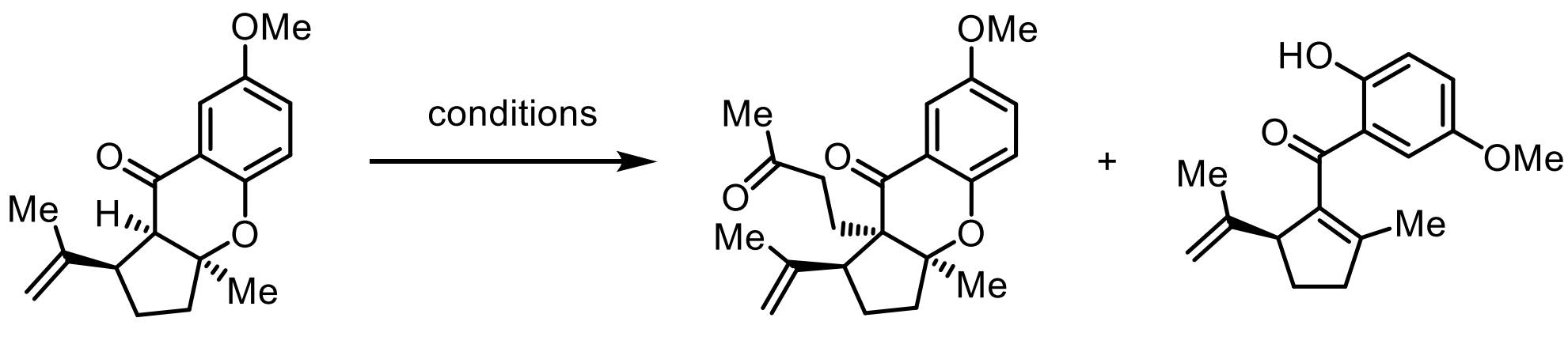

Entry	Conditions[a]	**12** (%)[b]	**13** (%)[b]	**19** (%)[b]
1[c]	MVK (1.85 M in THF, 1.0 equiv), NaOH (0.5 equiv), THF (0.1 M), 5 °C, 1 h	38	12	23
2[d]	MVK (0.5 M in THF, 1.0 equiv), NaOH (0.5 equiv), THF (0.1 M), 5 °C, 1 h (*Add dropwise via syringe pump over 20 min.*)	26	12	35
3[d]	MVK (0.5 M in THF, 1.0 equiv), NaOH (0.5 equiv), THF (0.1 M), 5 °C, 1 h (*Add dropwise via syringe pump over 40 min.*)	30	12	30
4[d]	MVK (1.2 equiv), NaOH (0.5 equiv), THF (0.1 M), 5 °C, 2 h (*Add dropwise via syringe pump over 36 min.*)	33	23	11
5[d]	MVK (1.85 M in THF, 1.0 equiv), NaOH (0.5 equiv), THF (0.1 M), 5 °C, 1 h (*Add dropwise via syringe pump over 30 min.*)	34	12	37
6[e]	MVK (1.85 M in THF, 1.0 equiv), NaOH (0.5 equiv), THF (0.1 M), 5 °C, 1 h (*Add dropwise one portion via a syringe pump every 5 min.*)	35	11	40
7[e]	MVK (1.85 M in THF, 1.0 equiv), NaOH (0.5 equiv), THF (0.1 M), 5 °C, 1 h (*Add dropwise one portion via a syringe pump every 20 min.*)	37	12	36
8[e]	MVK (1.85 M in THF, 1.0 equiv), NaOH (0.5 equiv), THF (0.05 M), 5 °C, 1 h	32	8	48
9[e]	MVK (1.85 M in THF, 1.0 equiv), NaOH (1.0 equiv), THF (0.05 M), 5 °C, 1 h	34	8	47
10[e,f,g]	MVK (1.85 M in THF, 1.0 equiv), NaOH (1.0 equiv), THF (0.05 M), 5 °C, 1 h	35 (58)	7 (15)	45 (7)

11[e]	MVK (1.85 M in THF, 1.0 equiv), NaOH (1.0 equiv), THF (0.05 M), 5 °C, 2 h	36	15	33
12[e]	MVK (1.85 M in THF, 1.0 equiv), NaOH (2.0 equiv), THF (0.05 M), 5 °C, 1 h	35	10	42
13[e]	MVK (1.85 M in THF, 1.5 equiv), NaOH (1.0 equiv), THF (0.05 M), 5 °C, 1 h	38	9	27
14[e]	MVK (1.85 M in THF, 2.0 equiv), NaOH (1.0 equiv), THF (0.05 M), 5 °C, 40 min	35	7	20
15[e]	MVK (1.85 M in THF, 1.0 equiv), NaOH (1.0 equiv), THF/PhMe (0.05 M), 5 °C, 1 h	31	4	51
16[e]	MVK (1.85 M in THF, 1.0 equiv), NaOH (1.0 equiv), THF/PhCl (0.05 M), 5 °C, 1 h	31	6	52
17[e]	MVK (1.85 M in THF, 1.0 equiv), KOH (1.0 equiv), THF/PhCl (0.05 M), 5 °C, 4 h	33	14	39
18[e]	MVK (1.85 M in THF, 1.0 equiv), KOH (1.0 equiv), THF (0.05 M), 5 °C, 1 h	42	19	24

[a]all reactions were performed on a 165 mg scale in 6.1 mL solvent except as otherwise indicated. [b]Isolated yield. [c]General procedure A. [d]General procedure B. [e]General procedure C. [f]**19** (1.0 g). [g]Yield of three cycles (in parentheses).

Note: General procedures are described on page S13 of the Supplementary Information.

Responses to Reviewer 3:

In this report, the authors present an asymmetric, divergent total synthesis of four *Ganoderma* meroterpenoids from a common intermediate, which is accessed from a chiral starting material. The synthetic route features several key transformations, including a diastereoselective *oxa*-Michael addition, a condition-controlled divergent aldol/etherification cascade, and an SmI_2-mediated radical cyclization to generate a highly strained cyclobutane ring.

Overall, the chemistry is elegant and interesting. The authors clearly demonstrate their efforts through extensive optimization studies and exploration of synthetic detours.

……

After addressing these minor revisions, the manuscript will be suitable for publication. The chemistry and overall approach are innovative and distinct from previous syntheses. In my opinion, this work is a valuable contribution and merits publication.

1. Line 24: “This class of natural products displays diverse biological activities, such as antitumor, anti-renal fibrosis, anti-allergic, anti-oxidant, anti-Acetyl cholinesterase (AChE), and antimicrobial.” The phrasing is awkward and should be revised for clarity.

◆ Thanks for the suggestion. The sentence has been revised to: “*Among these, Ganoderma meroterpenoids are structurally characterized by a unique 1,2,4-trisubstituted phenyl group and the unsaturated terpene subunit, exhibiting diverse biological activities, such as antitumor, antifibrotic, antiallergic, antioxidant, and antimicrobial activities*[10–13].”

2. Line 30: p-Smads. It would be helpful to briefly define this abbreviation or provide a short explanation.

◆ Thanks for the suggestion. We have added the definition of "*p-Smads*" as "*phosphorylated Smad2 and Smad3*" in the revised manuscript.

3. Line 233 (Methods): (rt, approximately 33 °C). This appears inconsistent, as room temperature is typically not 33 °C. Please clarify.

◆Thanks for the suggestion. We have replaced "*rt*" with "*33 °C*" throughout the revised manuscript and the Supplementary Information.

4. Page S3 (Note for compound **16**): Correct a word separation error.

◆Thanks for the suggestion. We have corrected this word separation error in the revised Supplementary Information (see revised page S5).

5. Page S5 (optimization table, and similar tables): Instead of unclear descriptions, consider using "complex mixture" for clarity.

◆Thanks for the suggestion. We have replaced "*messy*" with "*complex mixture*" throughout the revised Supplementary Information (see revised pages S7, S11 and S15).

6. Page S8 (procedure for **19**): Use "59 h" instead of "59.0 h" for consistency.

◆Thanks for the suggestion. We have corrected this accordingly (see revised page S10).

7. Tables in general (see S9 as an example): Temperature ranges such as "rt–60 °C" or "0 °C–rt" are inconsistently formatted. Consider replacing "rt" with a defined value (e.g., 23 °C) or standardizing notation throughout.

◆Thanks for the suggestion. In the revised manuscript and Supplementary Information, we have replaced "*rt*" with "*33 °C*" and standardized the notation throughout, for example, changing "*rt–60 °C*" to "*33 to 60 °C*".

8. General wording in procedures (see S10): Replace phrases like "the reaction was added a solution of…" with "a solution of … was added to the reaction mixture."

◆Thanks for the suggestion. We have corrected this accordingly. The specific revisions are as follows:

On page S13: "*A solution of MVK (1.85 M in THF, 662 μL, 1.23 mmol, 0.33 equiv) was added via syringe pump to the reaction mixture over 5 min.*"

On page S18: "*A solution of Comins' reagent (68.8 mg, 0.175 mmol, 1.2 equiv) in THF (0.75 mL) was added to the reaction mixture at –78 °C.*"

On page S22: "*SmI_2 (0.1 M in THF, 2.5 mL, 0.247 mmol, 2.0 equiv) was added to the reaction mixture at 0 °C.*"

On page S28: "*A solution of Comins' reagent (54.0 mg, 0.138 mmol, 5.0 equiv) in THF (0.2 mL) was added to the reaction mixture at 0 °C.*"

9. General phrasing (see S10): Replace "after stirred" with "after stirring."

◆Thanks for the suggestion. We have changed "*after stirred*" to "*after stirring*" throughout the revised Supplementary Information.

10. Ganocin A: In compound **S7**, the phenolic OH is included in the characterization but omitted in the natural product depiction, leading to an incorrect proton count. Additionally, although the ^{1}H NMR data match previous literature, inclusion of 2D NMR spectra would be beneficial—especially where signals overlap with water and affect integration.

◆ Thanks for your valuable suggestions. We have provided the 2D NMR spectra of (–)-ganocin A, with almost all ^{1}H and ^{13}C signals assigned and clearly labeled. The 2D NMR spectra indicate the absence of a phenolic OH in the ^{1}H NMR spectrum. The HSQC and HMBC correlations confirm that the signal at 1.62–1.56 ppm (m, overlap with H_2O, 1H) corresponds to a single proton (H-8′), and the integration value of 3.59 is attributable to interference from residual water. All spectra have been incorporated into the revised Supplementary Information (see revised page S59).

Note: we revised the ^{13}C assignments (C2') from prior reports (see page S37 of the revised Supplementary Information).

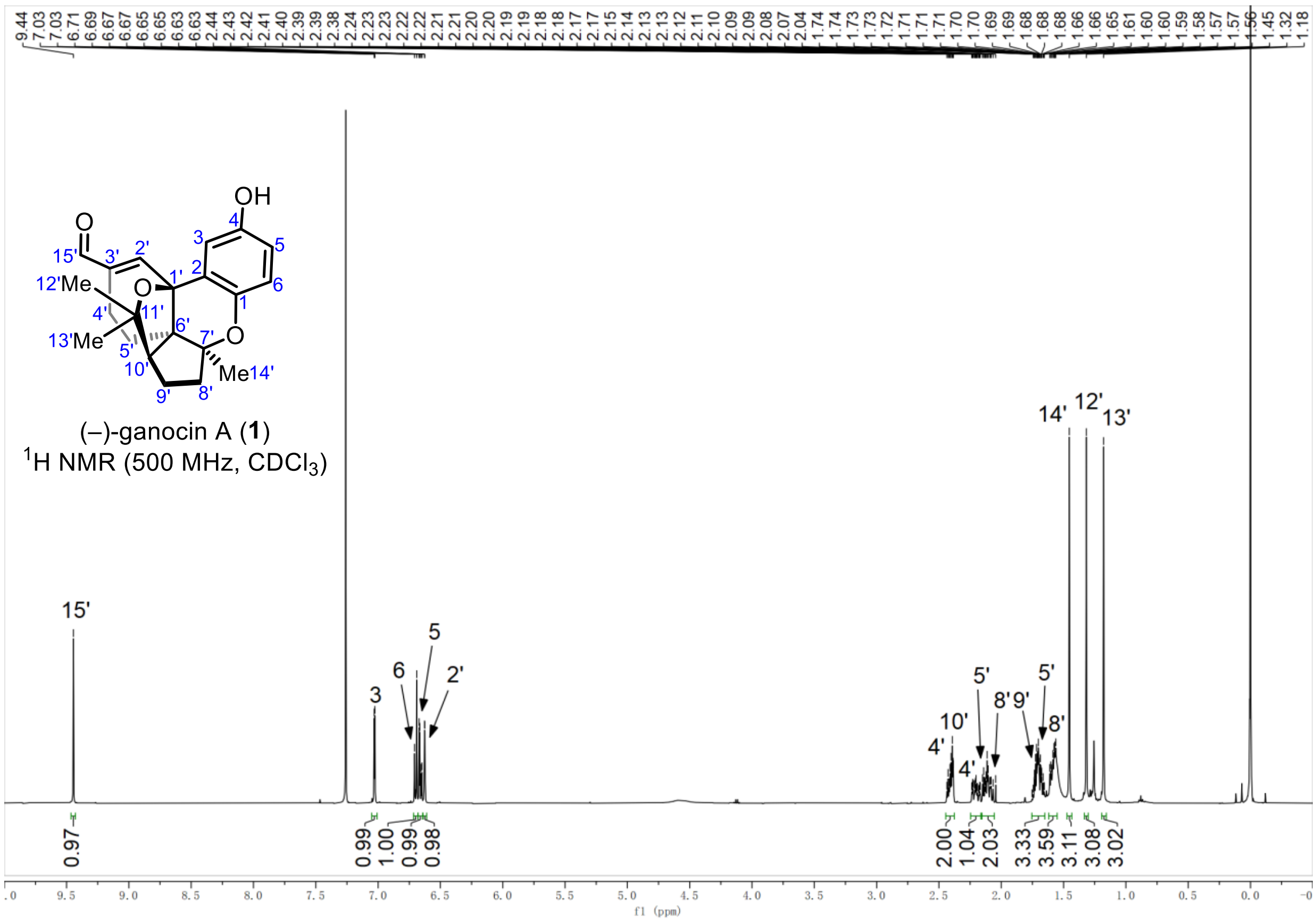
9.44 7.03 7.03 6.71 6.69 6.67 6.67 6.65 6.65 6.63 6.63 2.44 2.43 2.43 2.42 2.41 2.40 2.39 2.39 2.38 2.24 2.23 2.23 2.22 2.22 2.22 2.21 2.21 2.20 2.20 2.19 2.19 2.18 2.18 2.17 2.17 2.15 2.14 2.13 2.13 2.12 2.11 2.10 2.09 2.09 2.08 2.07 2.04 1.74 1.74 1.73 1.73 1.72 1.71 1.71 1.71 1.70 1.70 1.69 1.69 1.68 1.68 1.68 1.66 1.66 1.65 1.61 1.60 1.60 1.59 1.58 1.57 1.57 1.56 1.45 1.32 1.18
OH
O
(–)-ganocin A (1)
1H NMR (500 MHz, CDCl3)
15'
3
6
5
2'
4'
10'
4'
5'
8'
9'
5'
8'
14'
12'
13'
0.97
0.99
1.00
0.99
0.98
2.00
1.04
2.03
3.33
3.59
3.11
3.08
3.02
f1 (ppm)

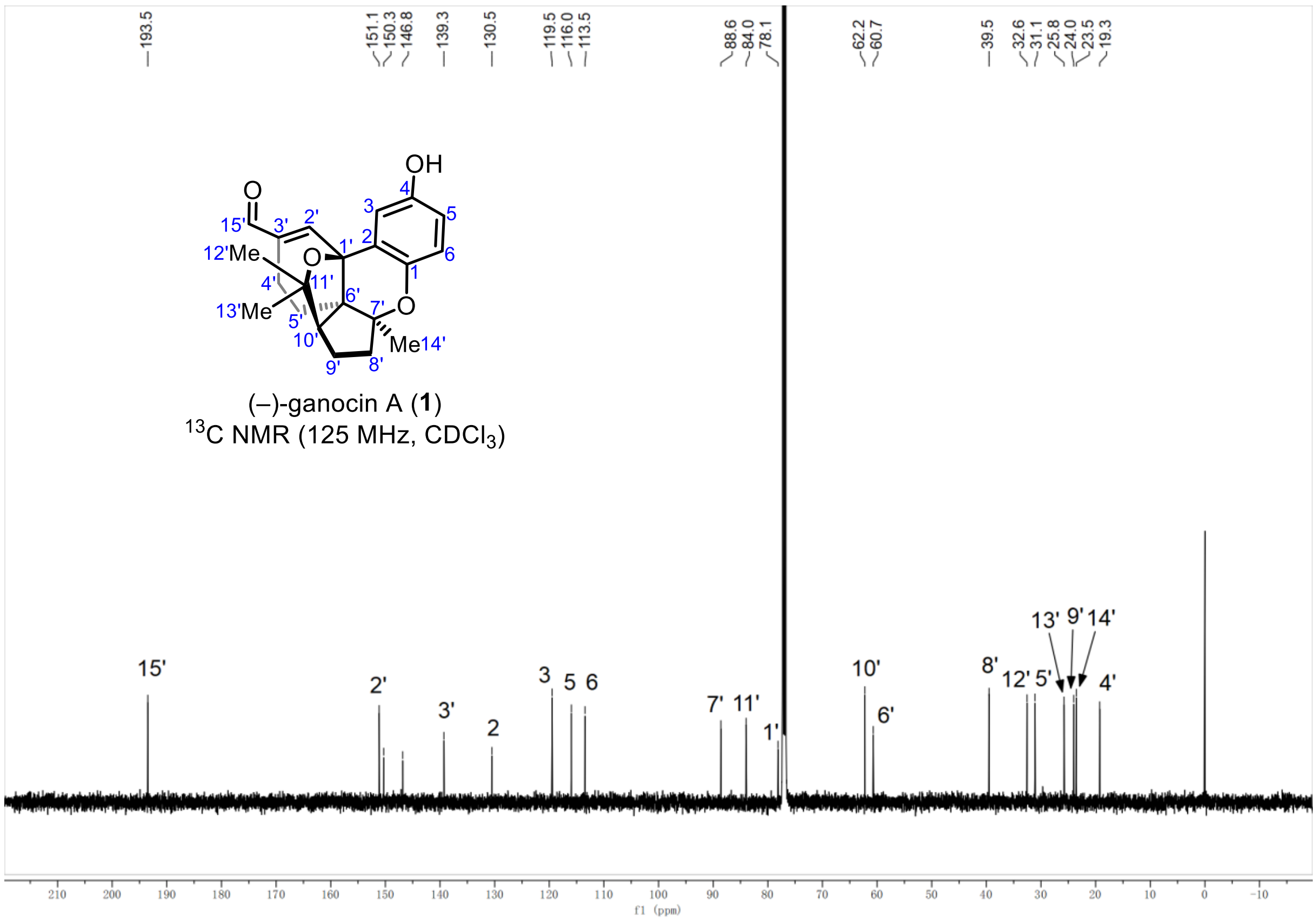
193.5
151.1
150.3
146.8
139.3
130.5
119.5
116.0
113.5
88.6
84.0
78.1
62.2
60.7
39.5
32.6
31.1
25.8
24.0
23.5
19.3
OH
O
(–)-ganocin A (1)
13C NMR (125 MHz, CDCl3)
15'
2'
3'
2
3
5
6
7'
11'
1'
10'
6'
8'
12'
5'
13'
9'
14'
4'
f1 (ppm)

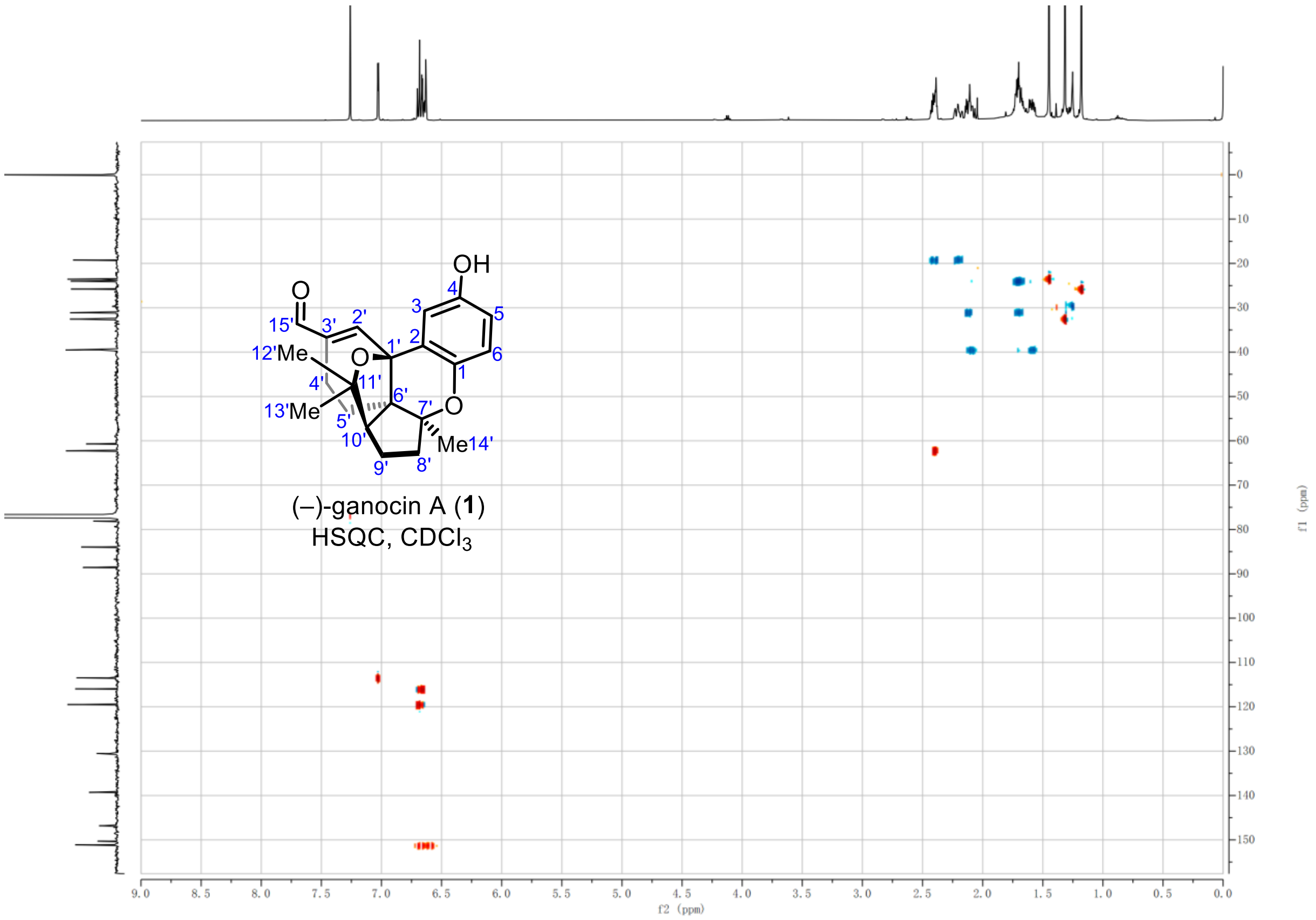
OH
O
15'
3'
2'
3
4
5
6
1
2
1'
12'Me
4'
11'
13'Me
5'
6'
7'
O
10'
9'
8'
Me14'
(–)-ganocin A (1)
HSQC, CDCl3
f2 (ppm)
f1 (ppm)

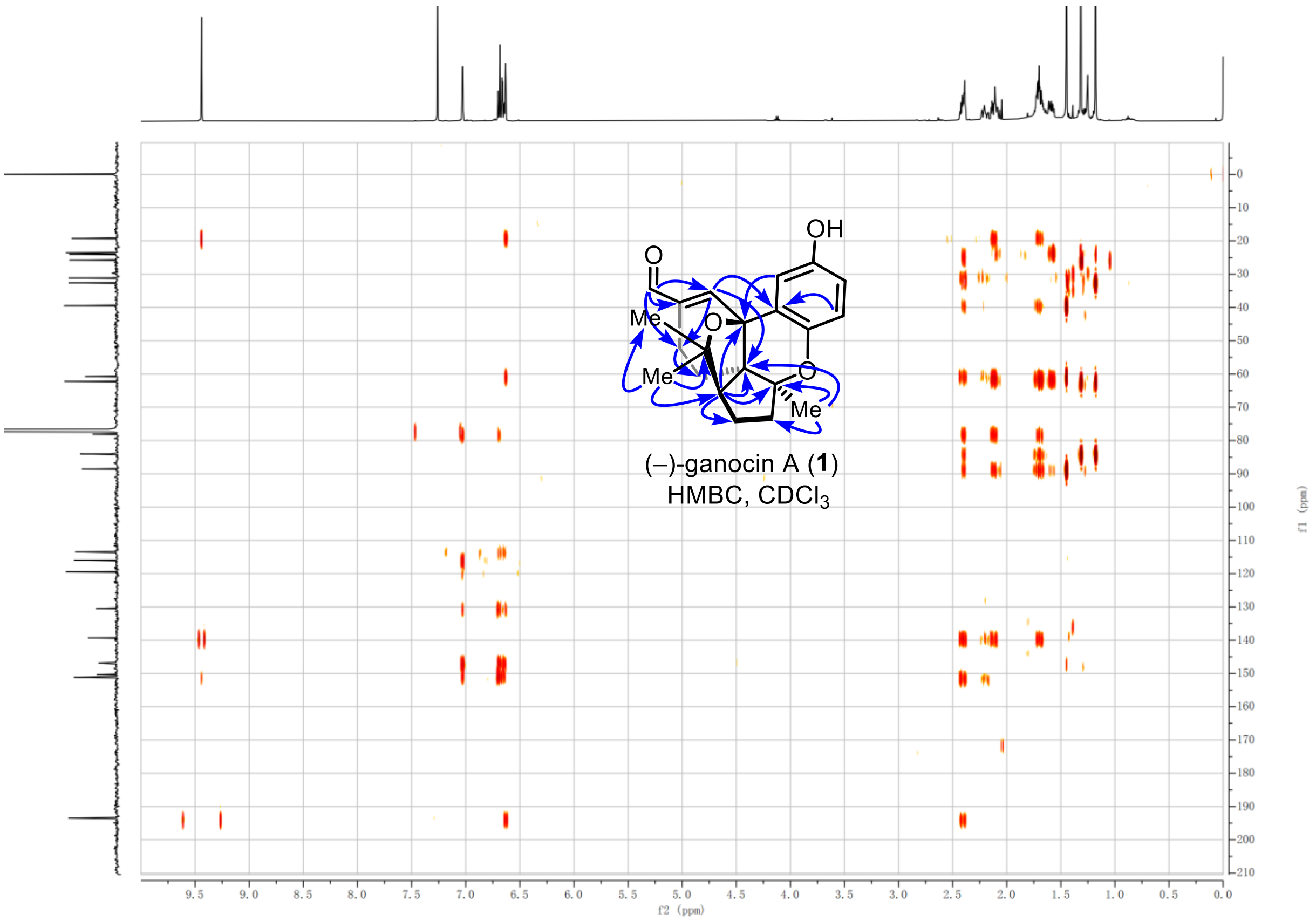
OH
O
Me
O
Me
O
Me
(–)-ganocin A (1)
HMBC, CDCl3
f2 (ppm)
f1 (ppm)

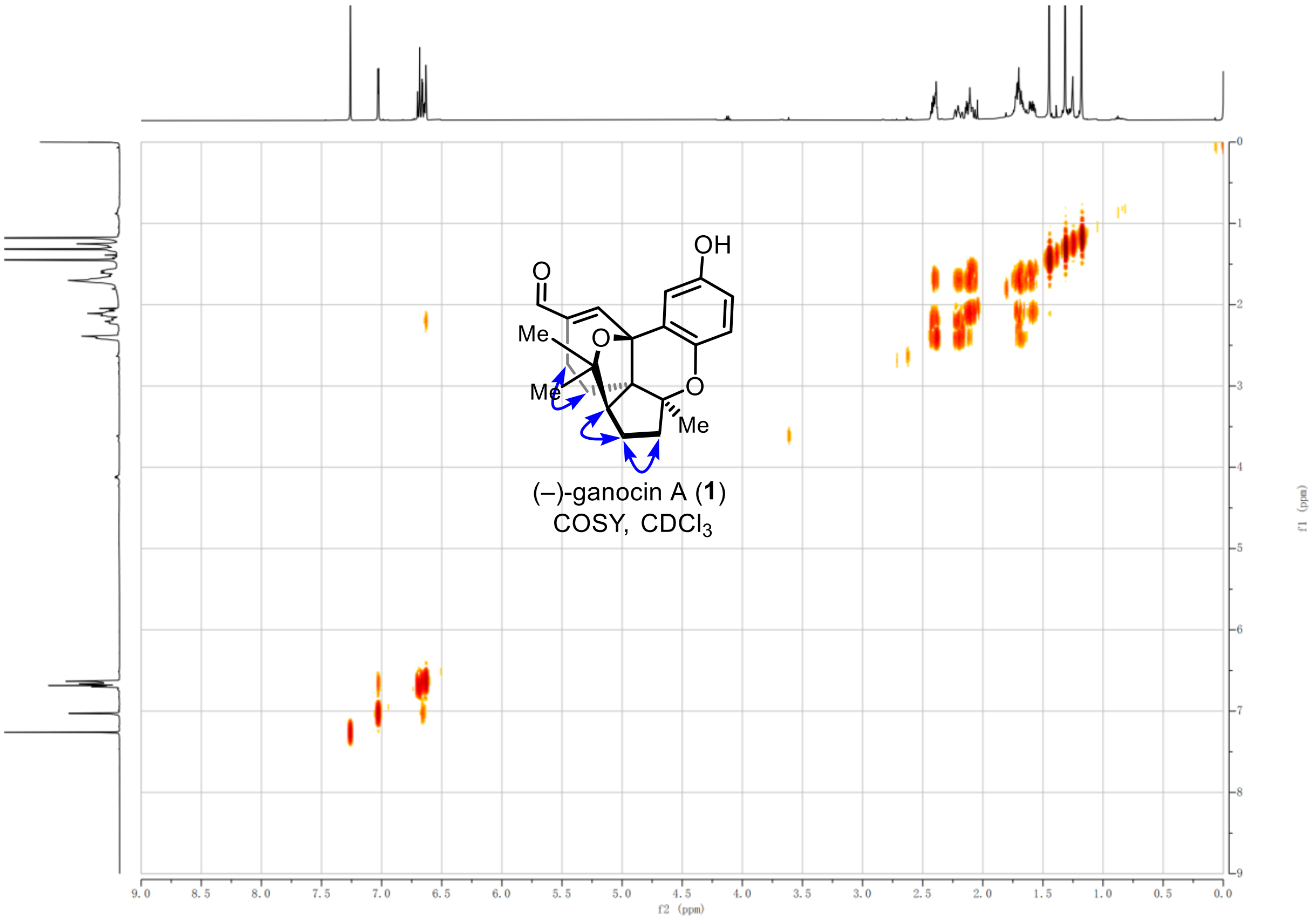
OH
O
Me
O
Me
O
Me
(–)-ganocin A (1)
COSY, CDCl3
f2 (ppm)
f1 (ppm)

11. Ganocin B: The signal at 7.20 ppm (dd, J = 8.7, 2.9 Hz) is reported as 1H, but appears integrated as 2H in the spectrum. Additionally, signals at 6.93 and 6.92 ppm are listed separately in the data but appear combined in the spectrum. Clarification is needed, and 2D NMR data would again be helpful.

◆Thanks for your valuable suggestions. We have provided the 2D NMR spectra of (–)-ganocin B, with almost all ^{1}H and ^{13}C signals assigned and clearly labeled. We have retested the ^{1}H NMR spectrum of (–)-ganocin B. The 2D NMR spectra confirm that the signal at 7.20 ppm corresponds to a single proton (H-5), and the integration value of 1.46 is attributable to interference from the C_5D_5N solvent. In addition, the signals at 6.93 and 6.92 ppm are now well resolved. The HSQC and HMBC correlations confirm that the signal at 6.93 ppm is a doublet (H-6), and the signal at 6.92 ppm is a singlet (H-2′). All spectra have been incorporated into the revised Supplementary Information (see revised page S63).

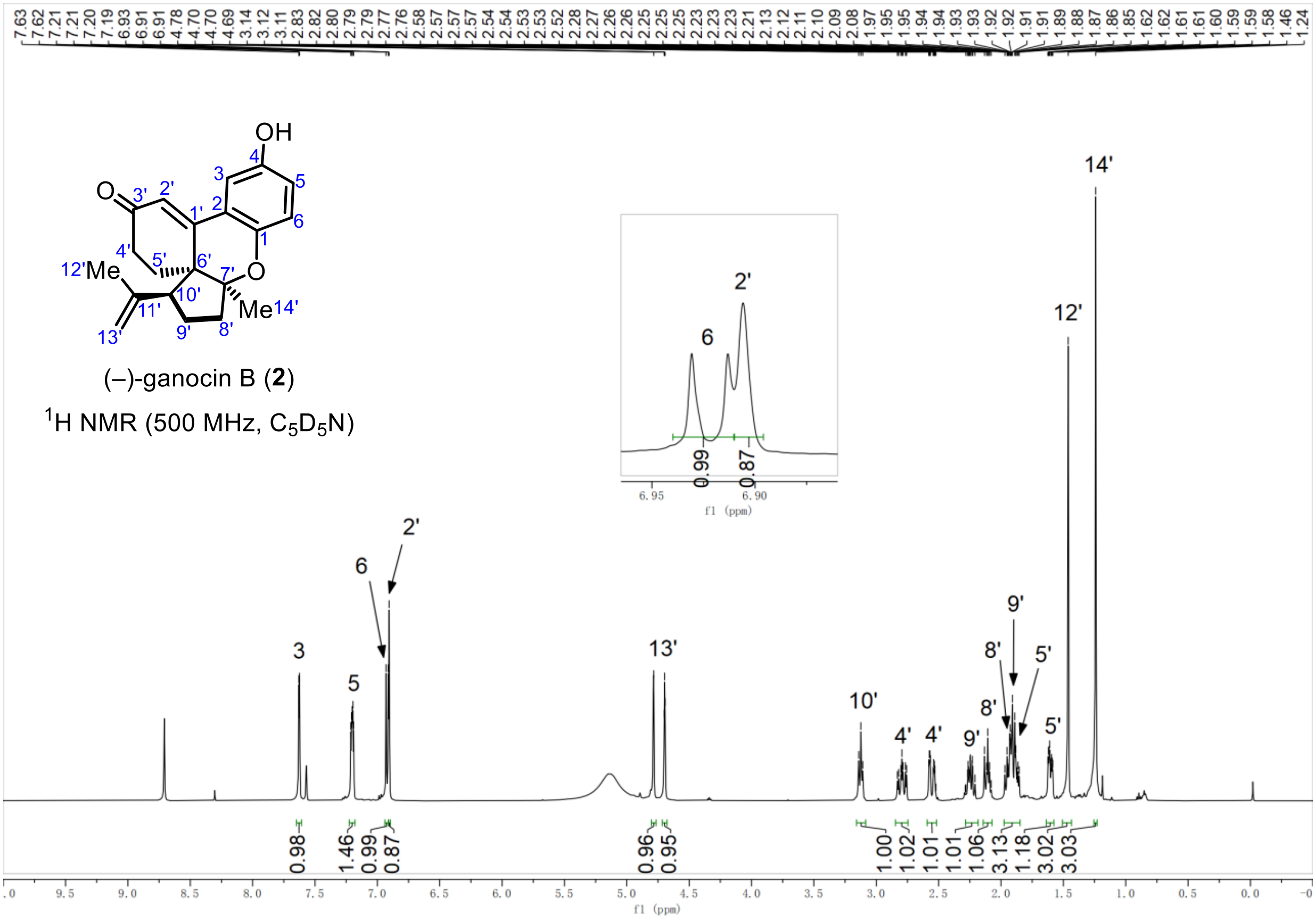

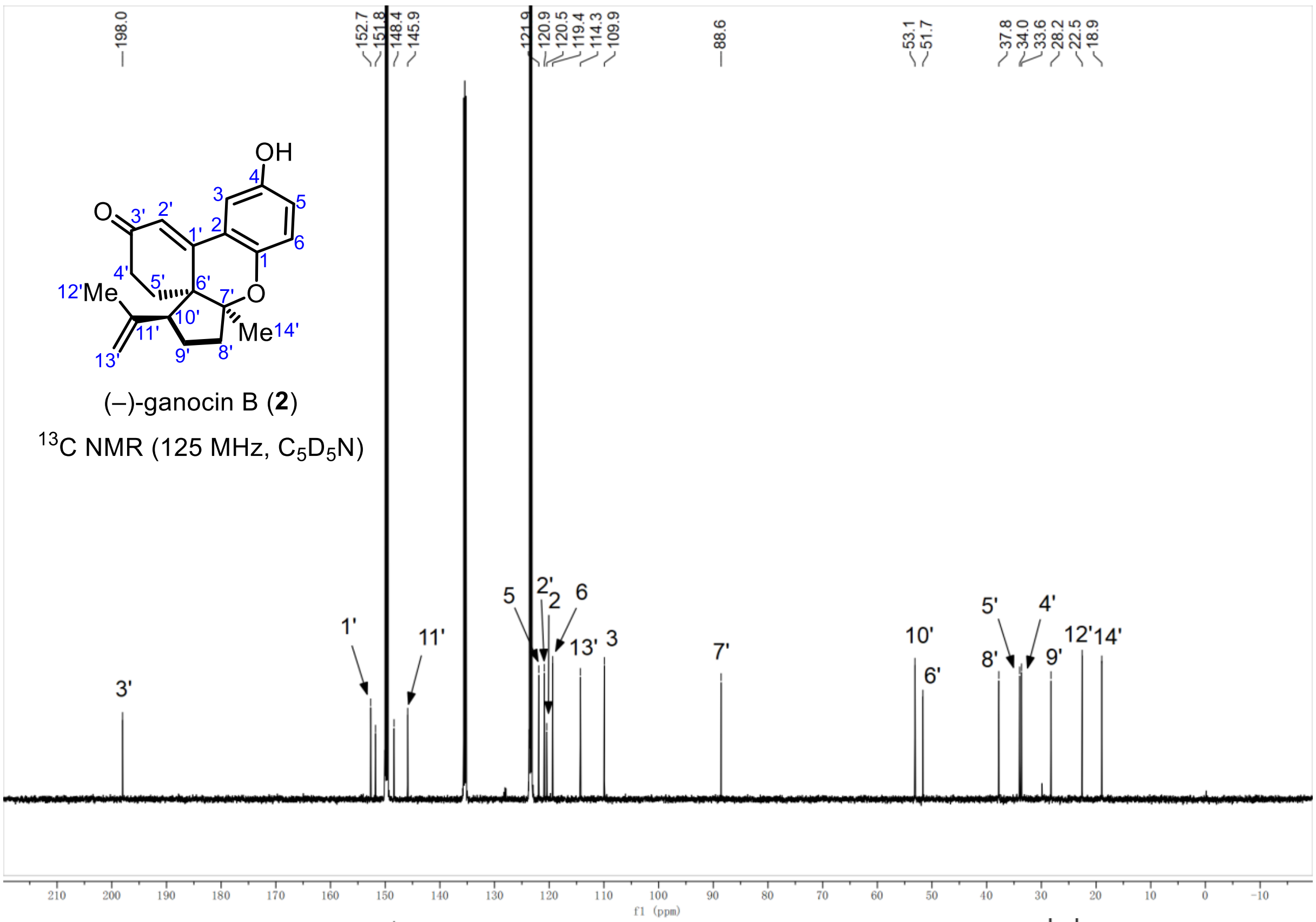

198.0
152.7
151.8
148.4
145.9
121.9
120.9
120.5
119.4
114.3
109.9
88.6
53.1
51.7
37.8
34.0
33.6
28.2
22.5
18.9
(–)-ganocin B (2)
^{13}C NMR (125 MHz, C_5D_5N)
3'
1'
11'
5
2'
2
6
13'
3
7'
10'
6'
5'
8'
4'
9'
12'
14'
f1 (ppm)

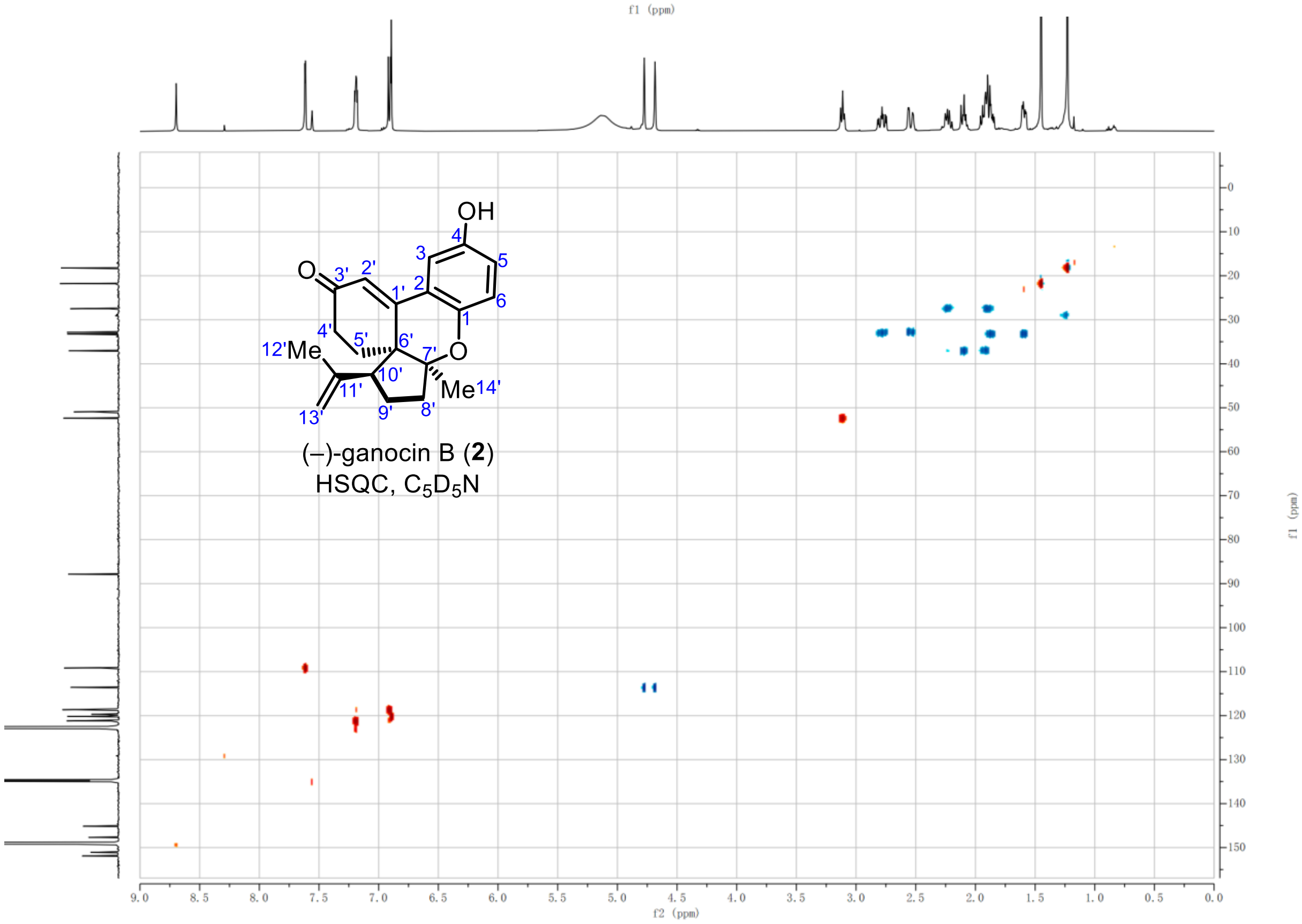

(–)-ganocin B (2)
HSQC, C_5D_5N
f2 (ppm)
f1 (ppm)

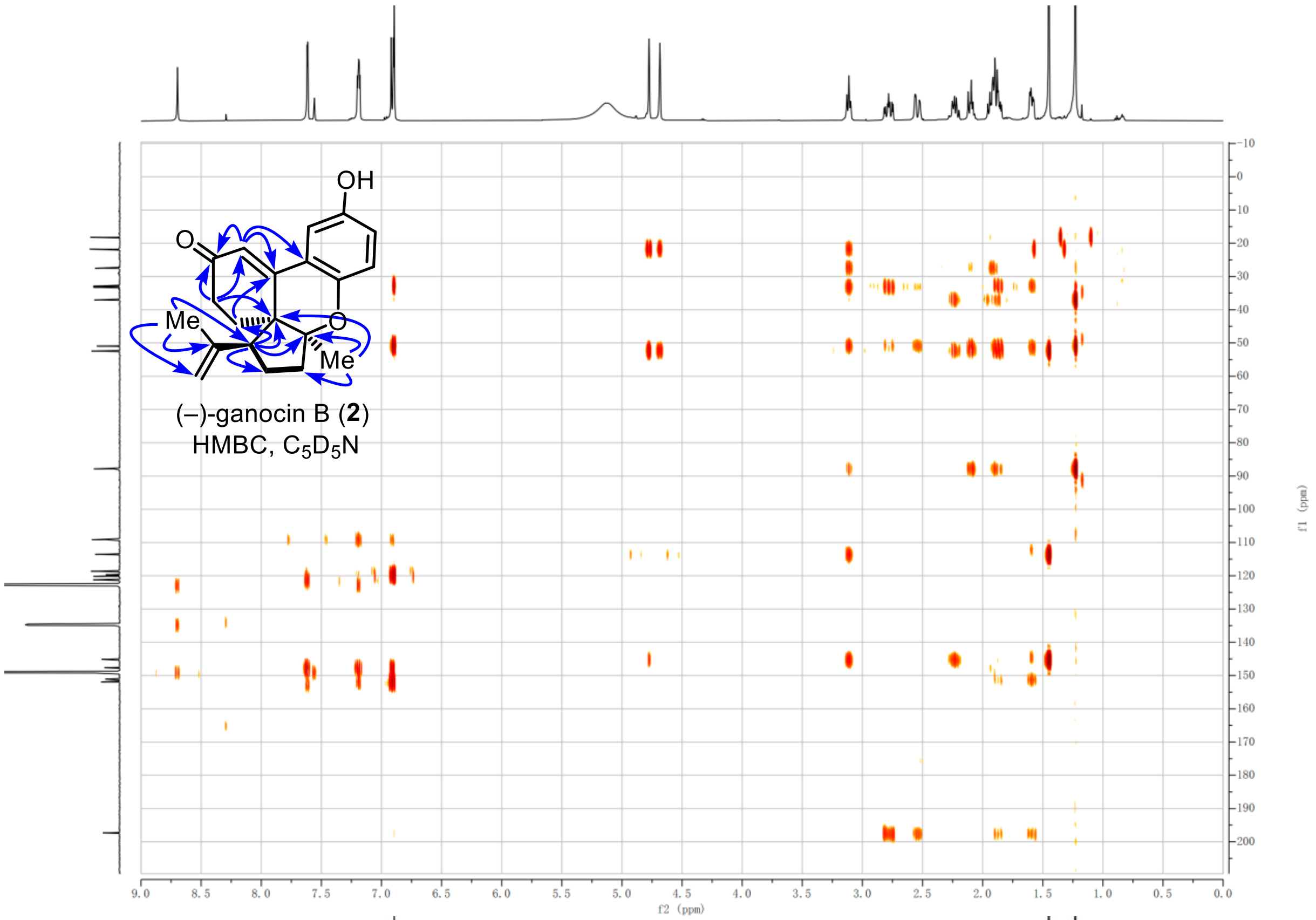
OH
O
Me
O
Me
(–)-ganocin B (2)
HMBC, C5D5N
f2 (ppm)
f1 (ppm)

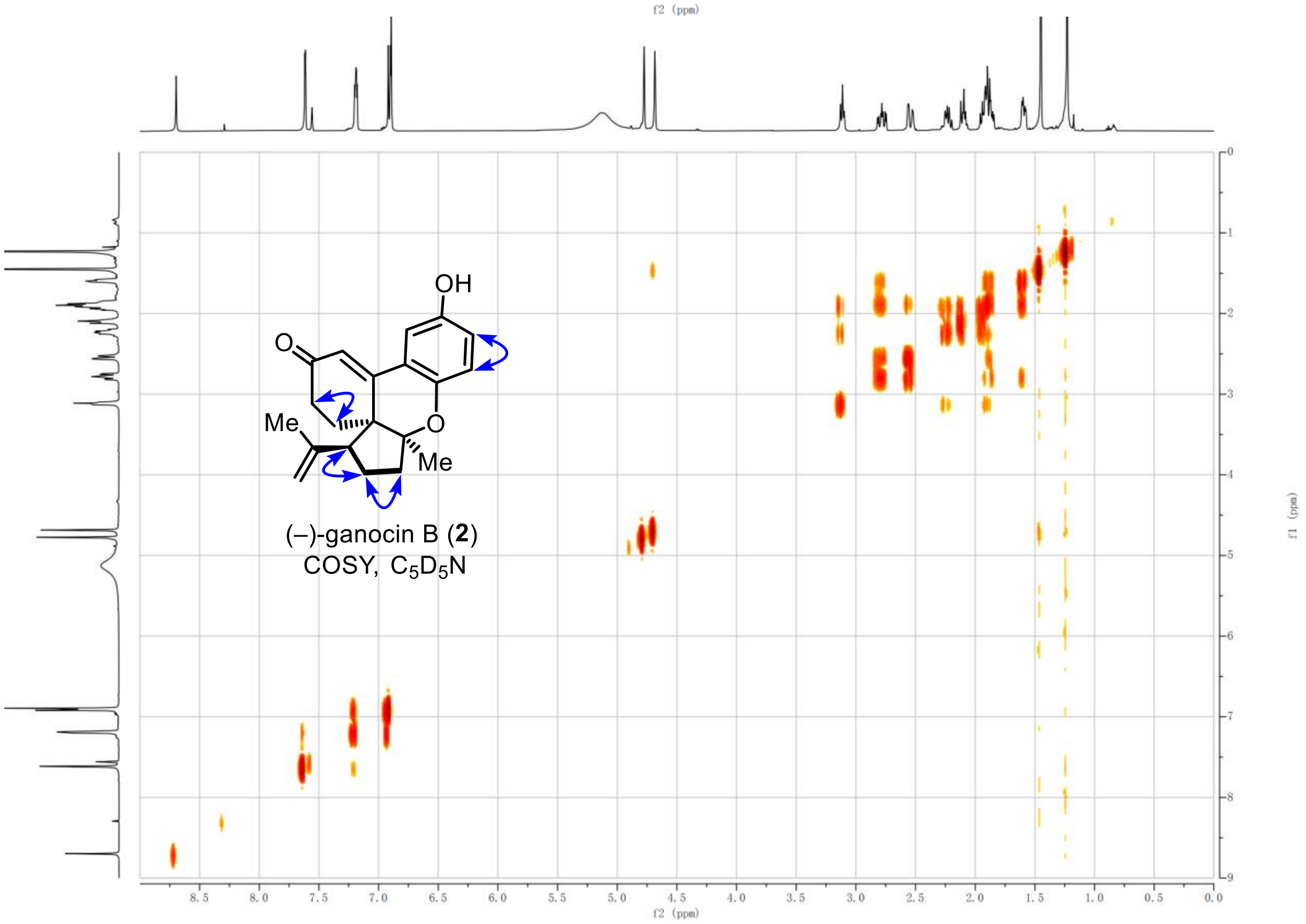
OH
O
Me
O
Me
(–)-ganocin B (2)
COSY, C5D5N
f2 (ppm)
f1 (ppm)

12. Compound **23**: No 2D NMR data are provided.

◆Thanks for the suggestion. We have provided the 2D NMR spectra of compound **23**, with all ^{1}H and ^{13}C signals fully assigned and clearly labeled. All spectra have been incorporated into the revised Supplementary Information (see revised page S66).

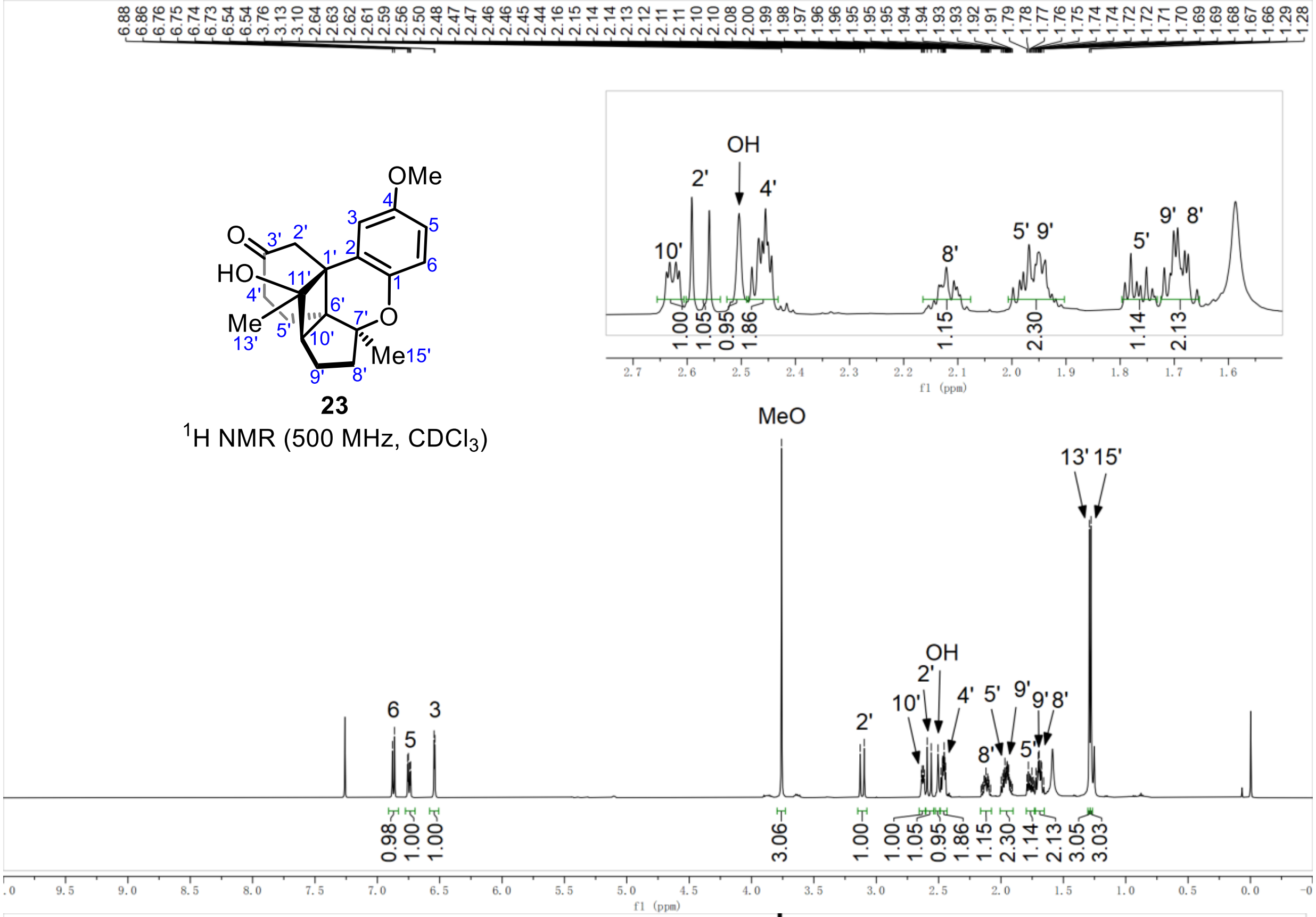

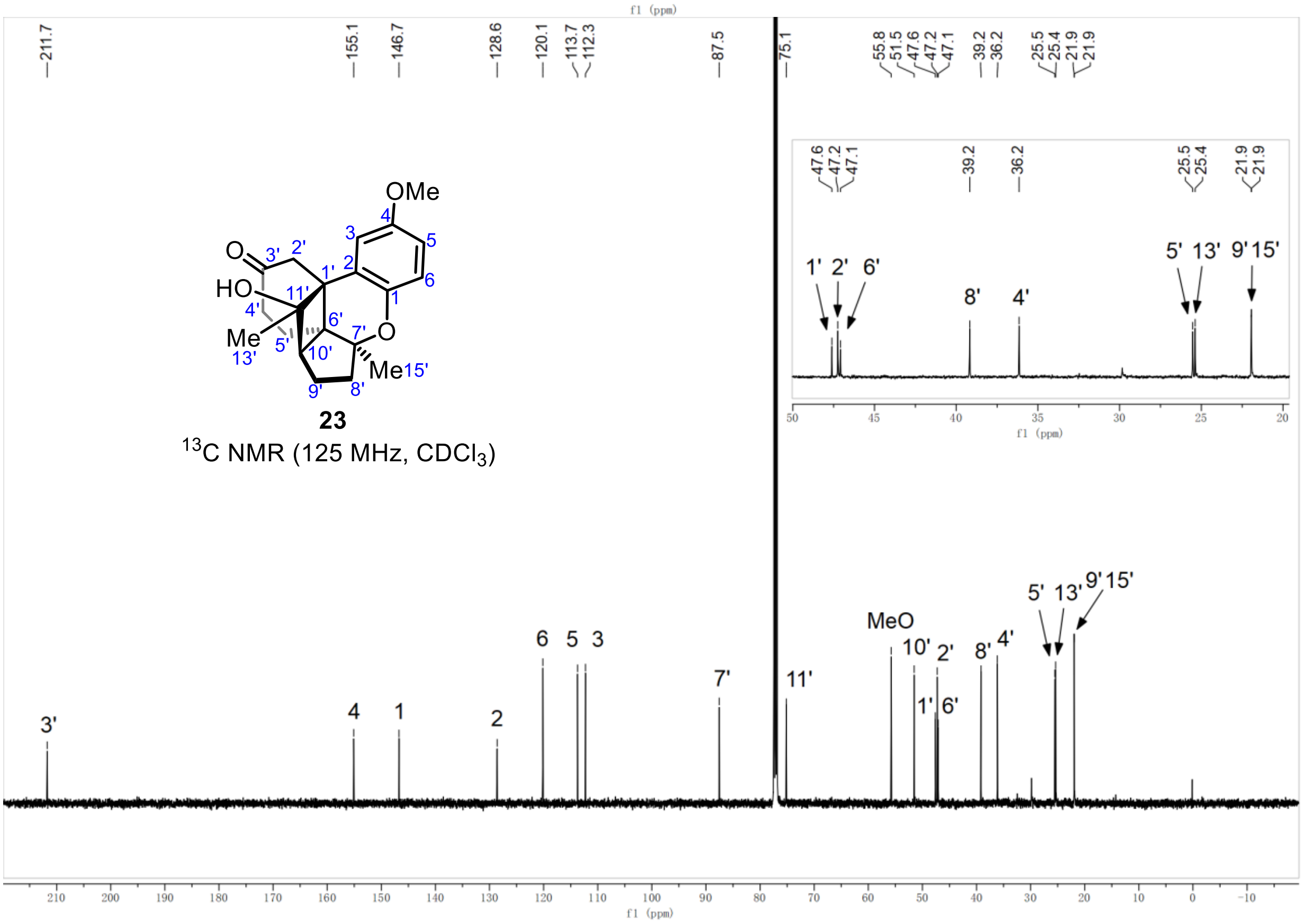

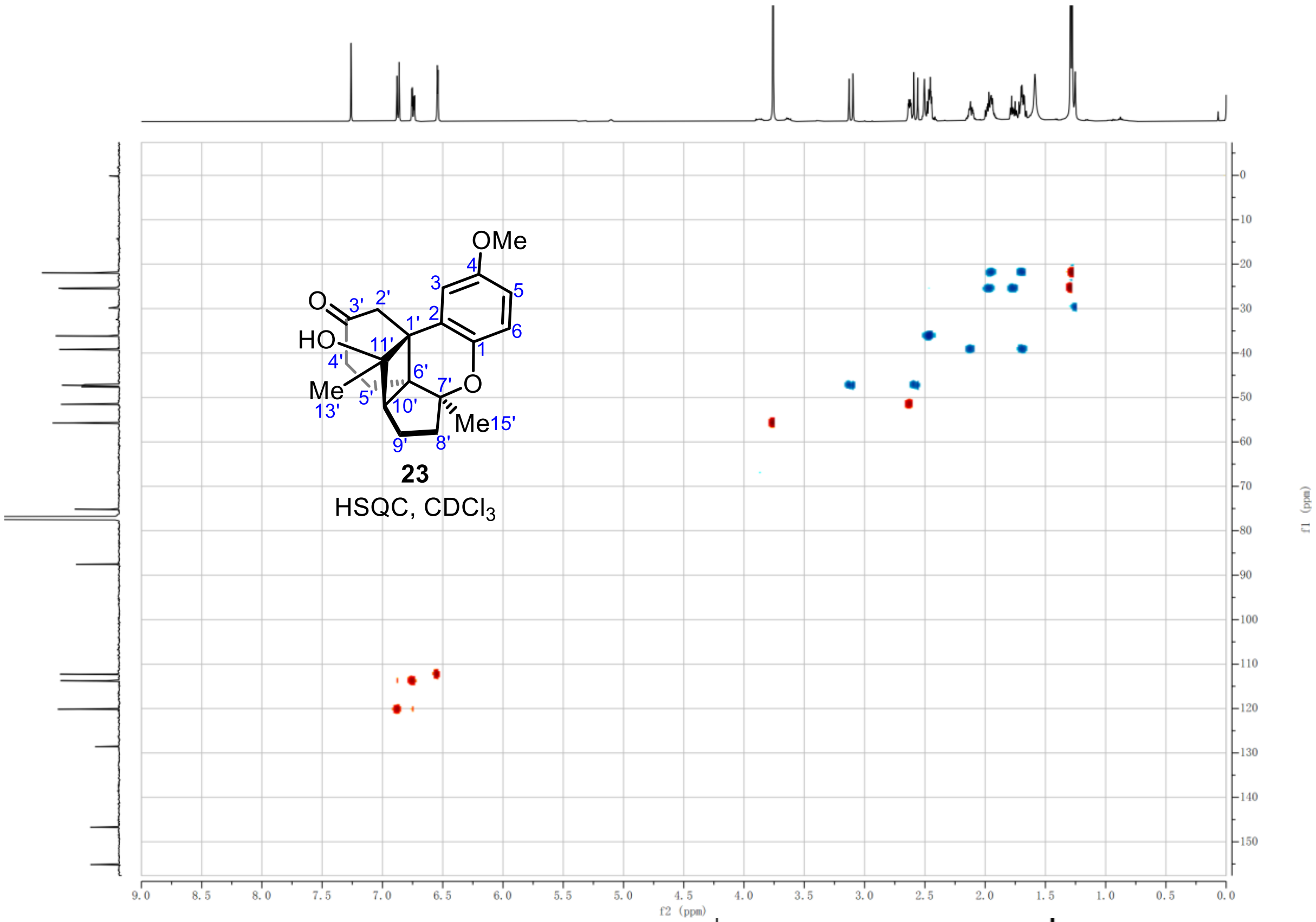
OMe
O
HO
Me
Me15'
23
HSQC, CDCl3
f2 (ppm)
f1 (ppm)

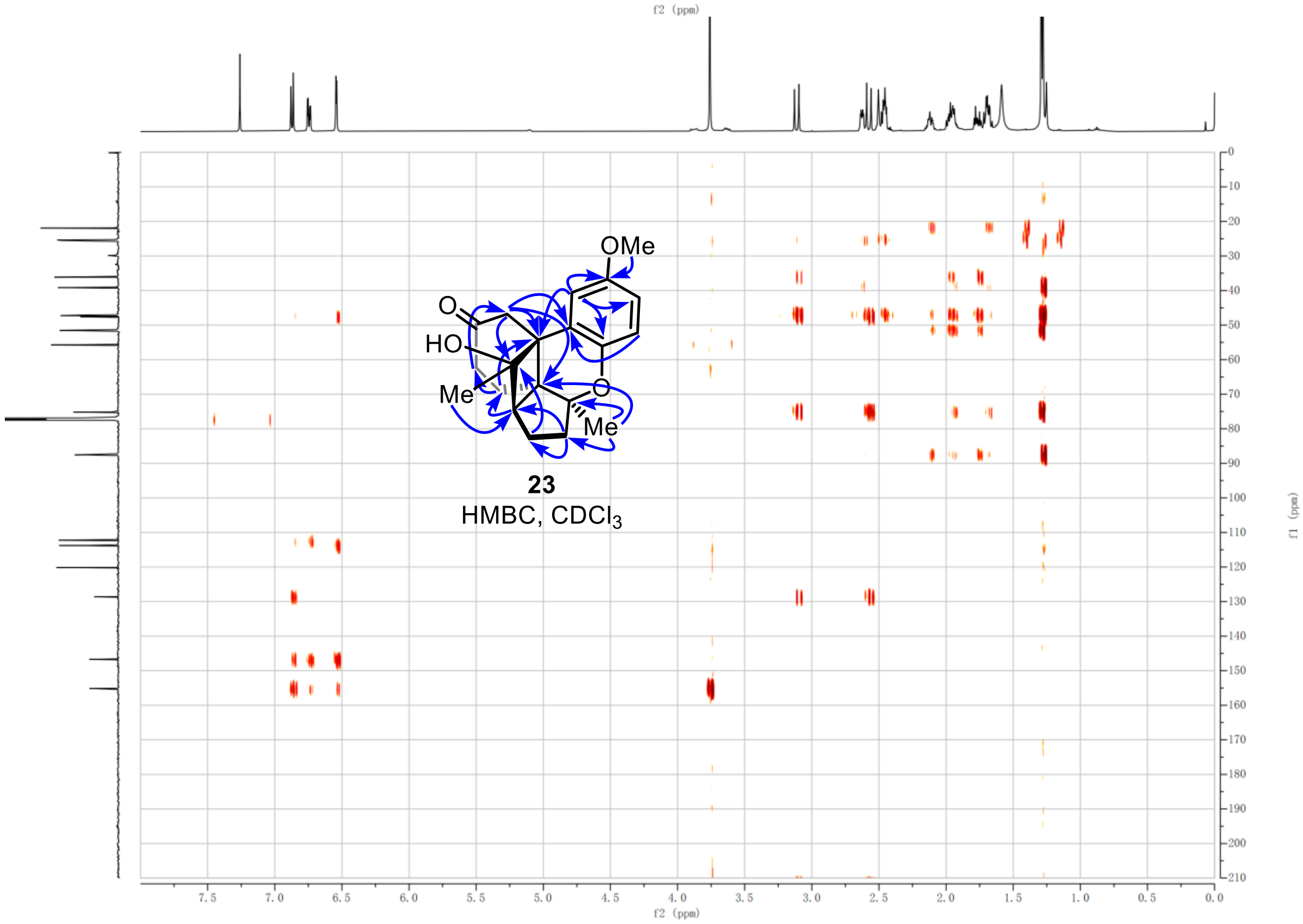
OMe
O
HO
Me
Me
23
HMBC, CDCl3
f2 (ppm)
f1 (ppm)

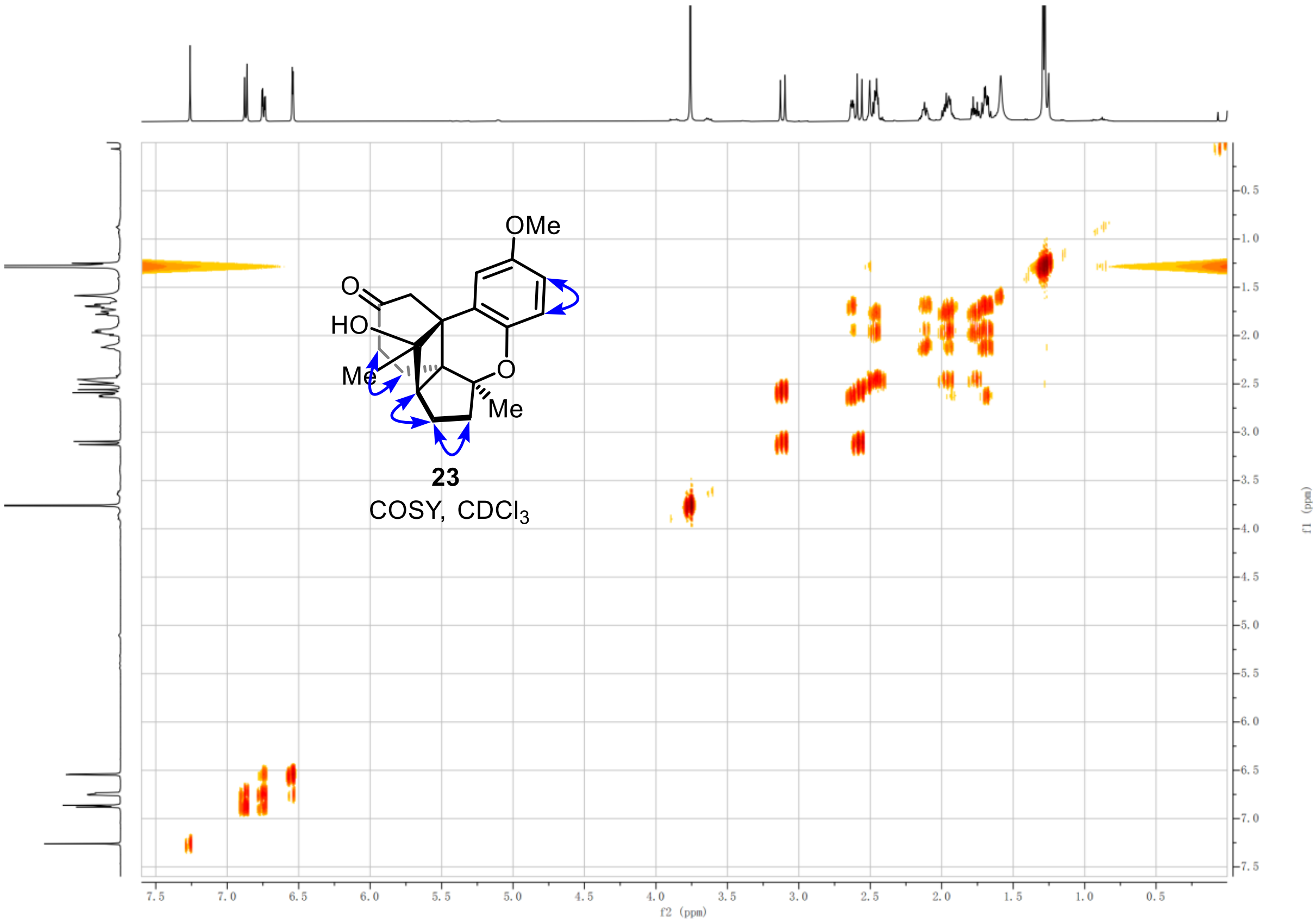

13. Compound **27**: Reported as a red oil, yet X-ray data are provided. Please clarify if this is correct.

◆Thanks for the suggestion. Compound **27** was initially isolated as a red oil, which solidified to a red solid upon refrigeration at 5 °C. We have revised "*red oil*" to "*red solid*" and determined its melting point to be 216–217 °C (see revised page S24).

14. Compounds **S18** and **S19**: Signals overlap with water; inclusion of 2D NMR spectra would strengthen the assignments.

◆Thanks for the suggestion. We have provided the 2D NMR spectra of compounds **S25** and **S26** (originally **S18** and **S19**), with all ^{1}H and ^{13}C signals fully assigned and clearly labeled. We have retested the ^{1}H NMR spectrum of compound **S25**. Due to the larger sample amount, no water peak is observed, and the integration value is correct (1.06).

The HSQC and HMBC correlations of compound **S26** confirm that the signal at 1.60–1.50 ppm (m, overlap with H_2O, 2H) corresponds to only two protons (H-8′ and H-9′), and the integration value of 3.37 is attributable to interference from residual water. All spectra have been incorporated into the revised Supplementary Information (see revised pages S70 and S72).

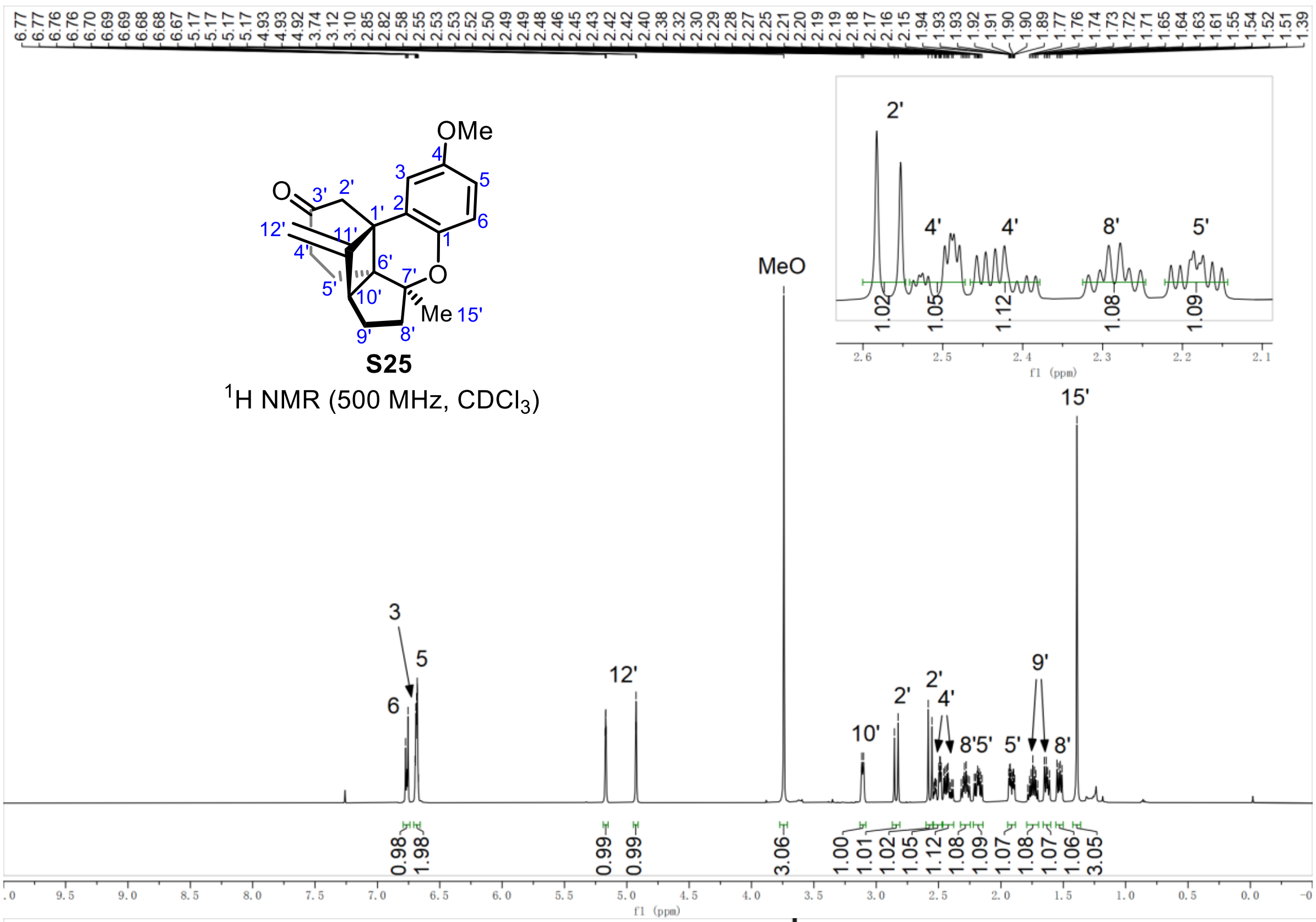
S25
^1H NMR (500 MHz, $CDCl_3$)
MeO
15'
2'
4'
8'
5'
3
5
6
12'
10'
9'
0.98
1.98
0.99
0.99
3.06
1.00
1.01
1.02
1.05
1.12
1.08
1.09
1.07
1.08
1.07
1.06
3.05
f1 (ppm)

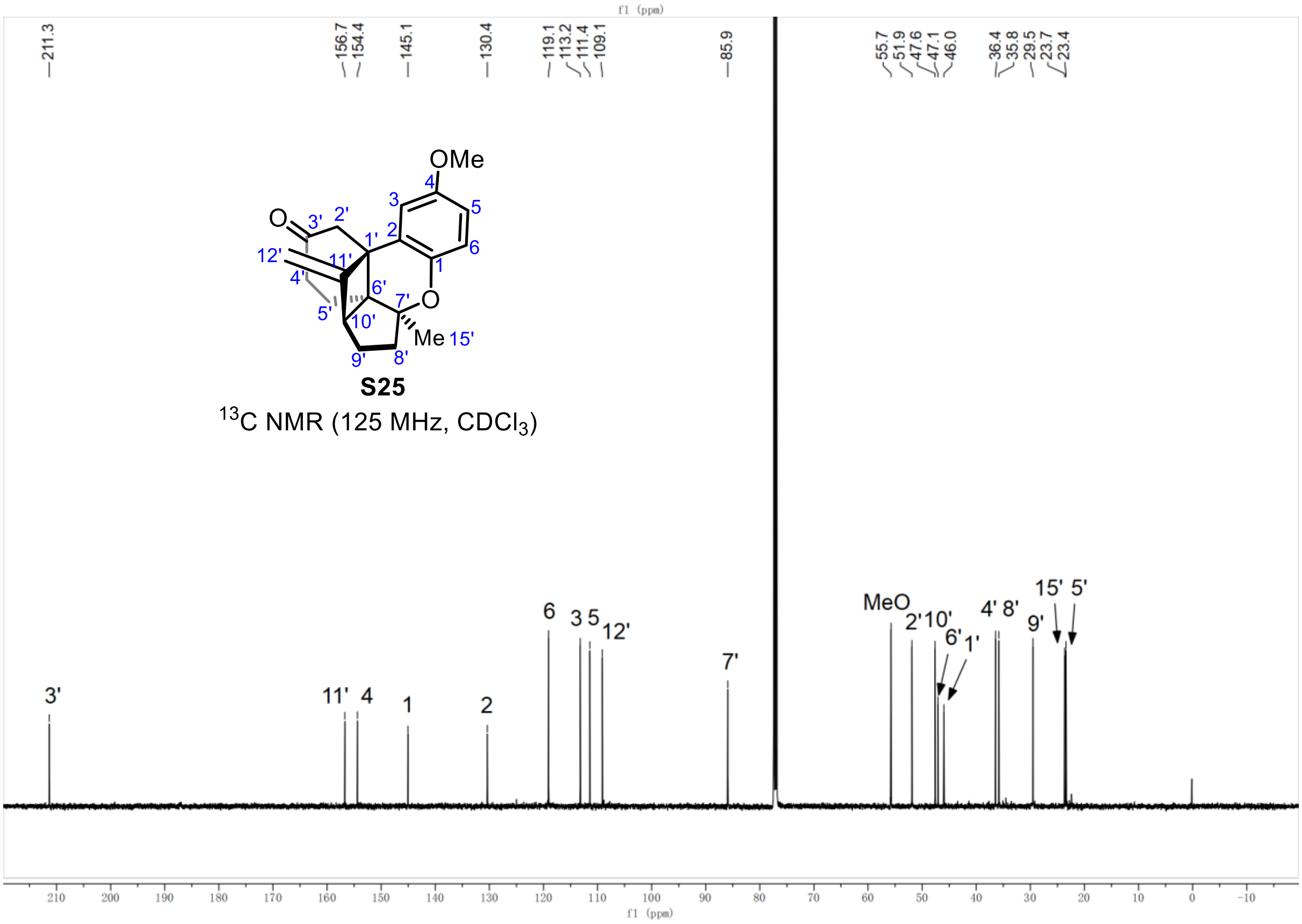
211.3
156.7
154.4
145.1
130.4
119.1
113.2
111.4
109.1
85.9
55.7
51.9
47.6
47.1
46.0
36.4
35.8
29.5
23.7
23.4
S25
^{13}C NMR (125 MHz, $CDCl_3$)
3'
11'
4
1
2
6
3
5
12'
7'
MeO
2'
10'
6'
1'
4'
8'
9'
15'
5'
f1 (ppm)

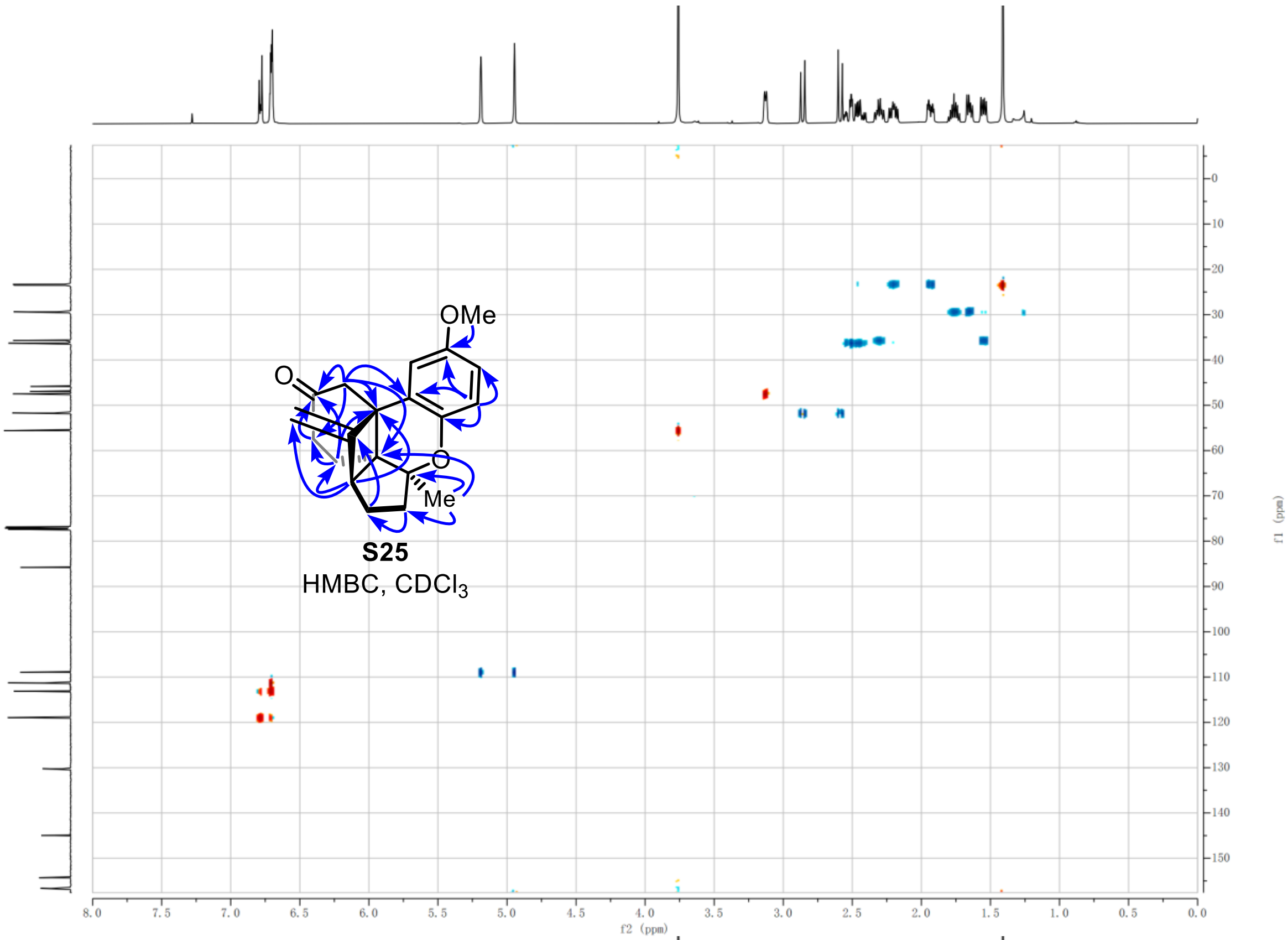
OMe
O
Me
S25
HMBC, CDCl3
f2 (ppm)
f1 (ppm)

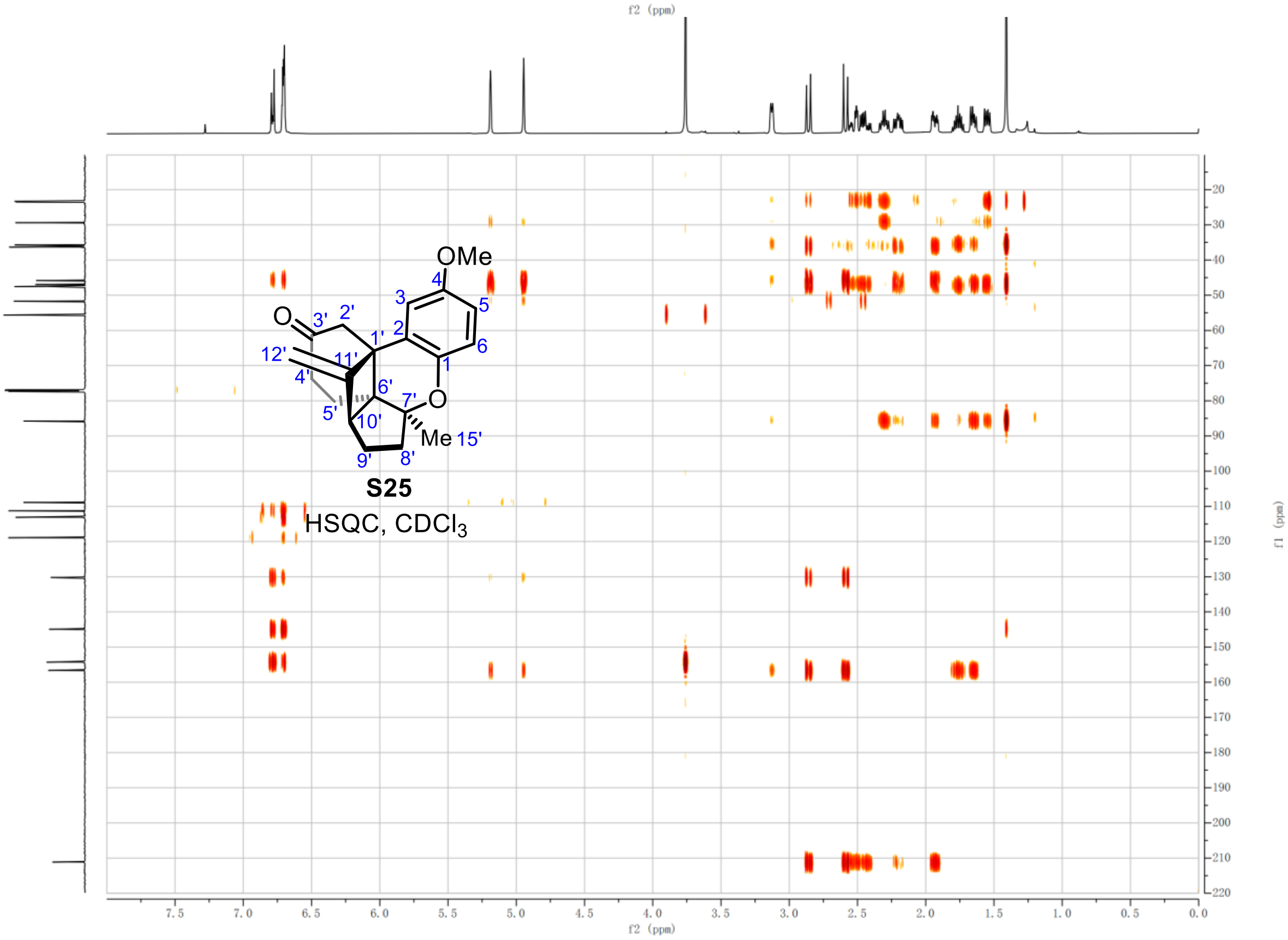
OMe
4
3
5
6
1
2
O
3'
2'
1'
12'
11'
4'
6'
5'
10'
7'
O
Me 15'
9'
8'
S25
HSQC, CDCl3
f2 (ppm)
f1 (ppm)

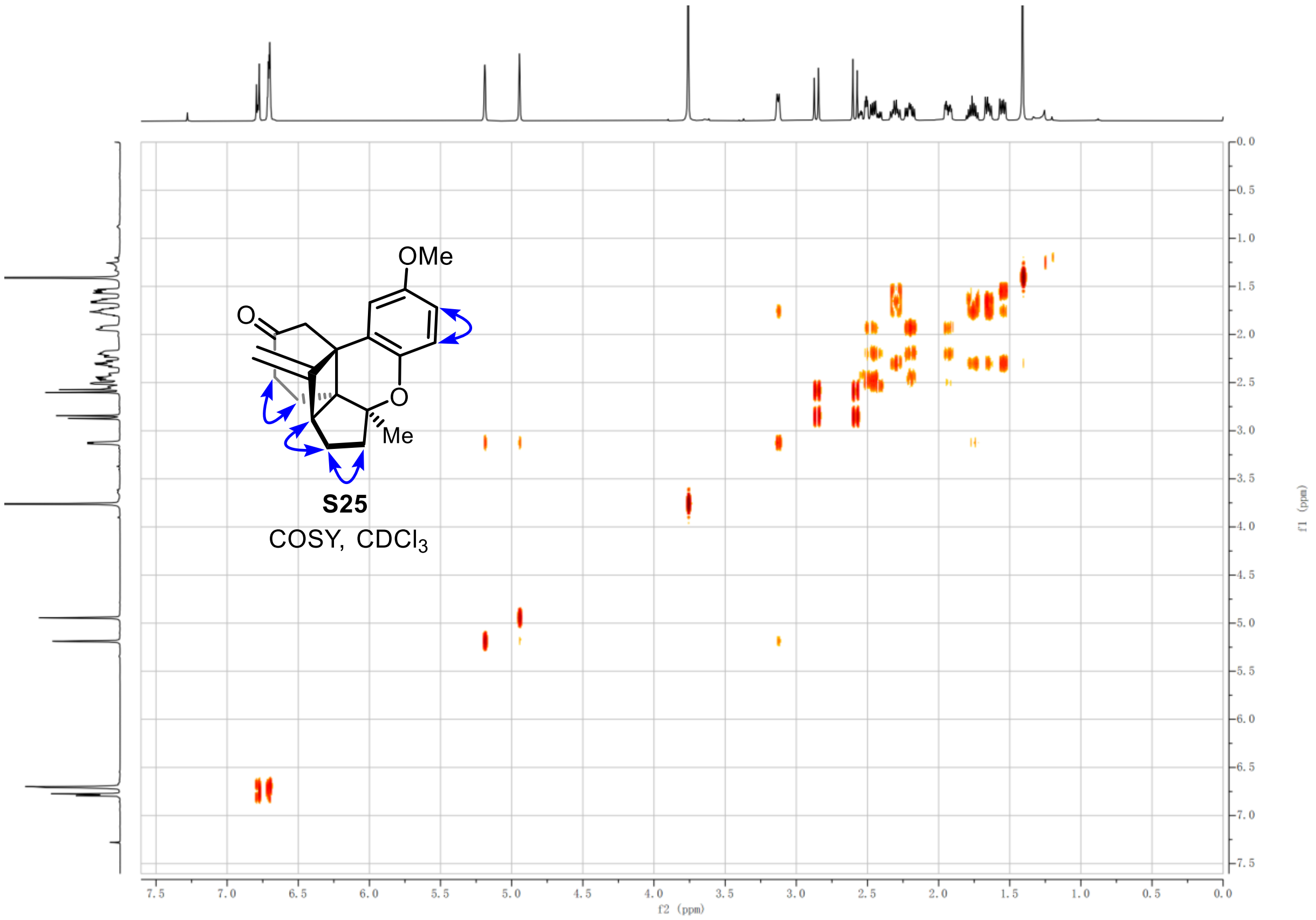

OMe
O
Me
S25
COSY, CDCl3
f2 (ppm)
f1 (ppm)

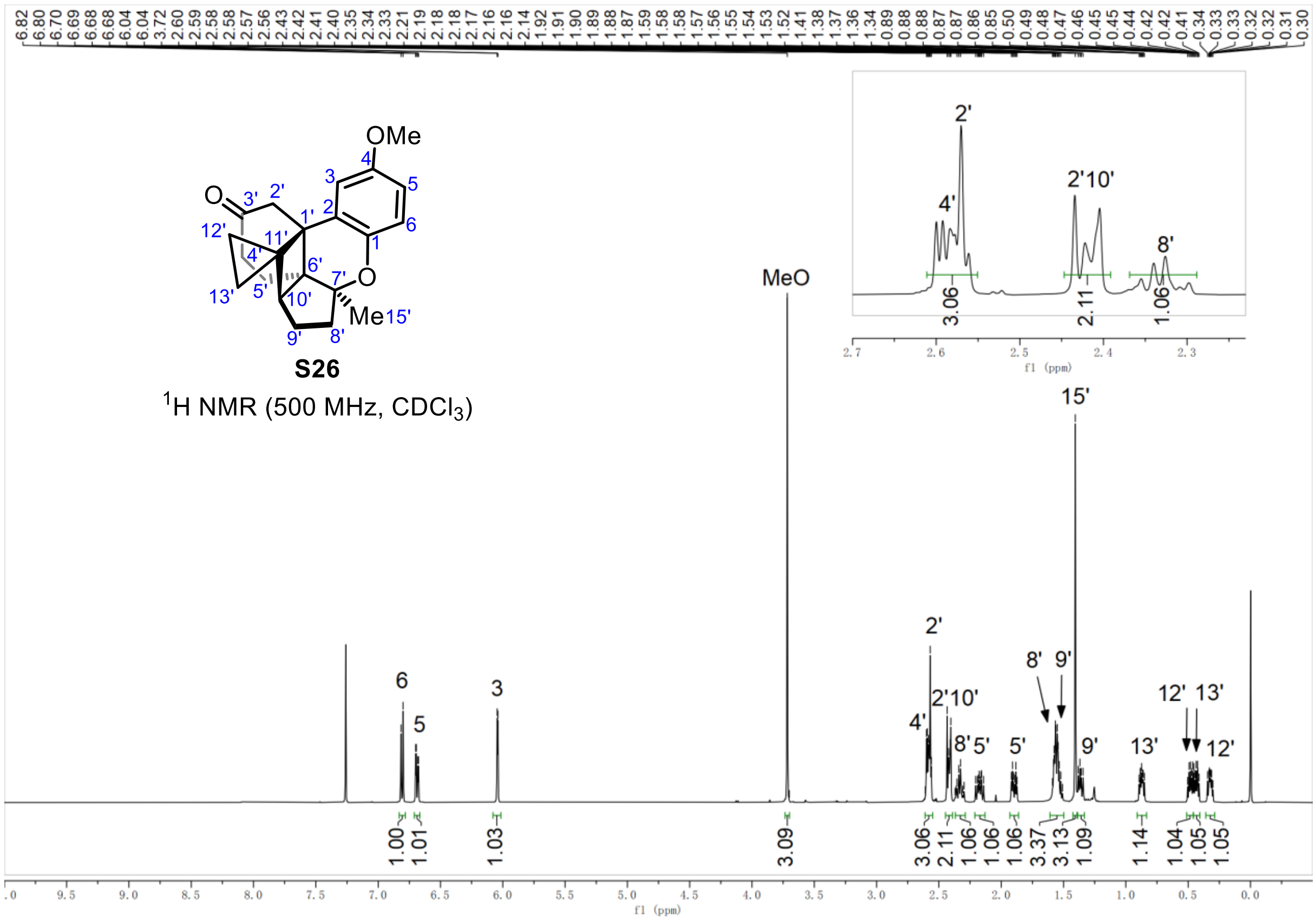

OMe
S26
1H NMR (500 MHz, CDCl3)
MeO
15'
2'
4'
2'10'
8'
6
5
3
9'
13'
12'
f1 (ppm)

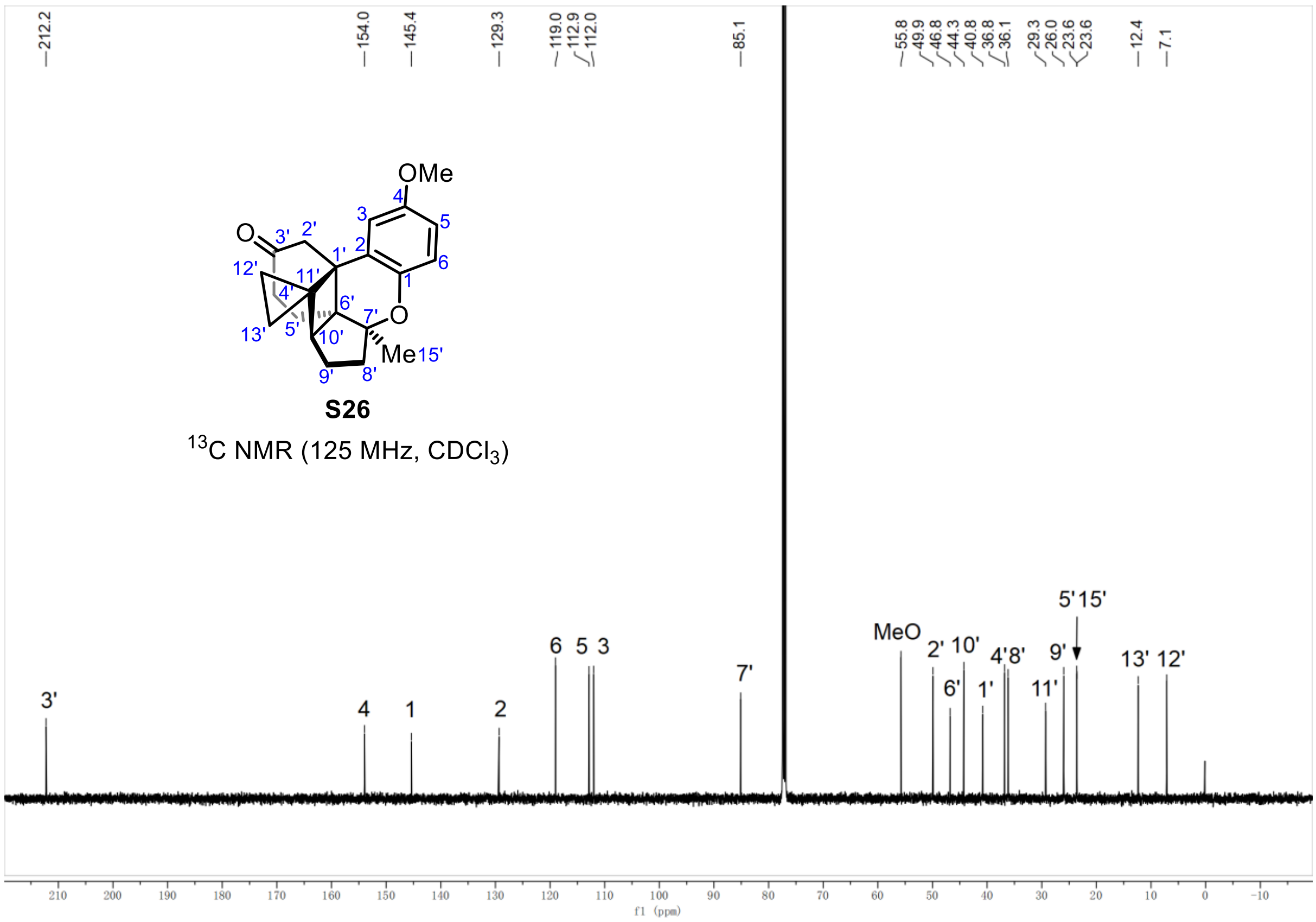

212.2
154.0
145.4
129.3
119.0
112.9
112.0
85.1
55.8
49.9
46.8
44.3
40.8
36.8
36.1
29.3
26.0
23.6
23.6
12.4
7.1
OMe
S26
13C NMR (125 MHz, CDCl3)
3'
4
1
2
6 5 3
7'
MeO
2' 10'
6'
1'
4'8'
11'
9'
5'15'
13' 12'
f1 (ppm)

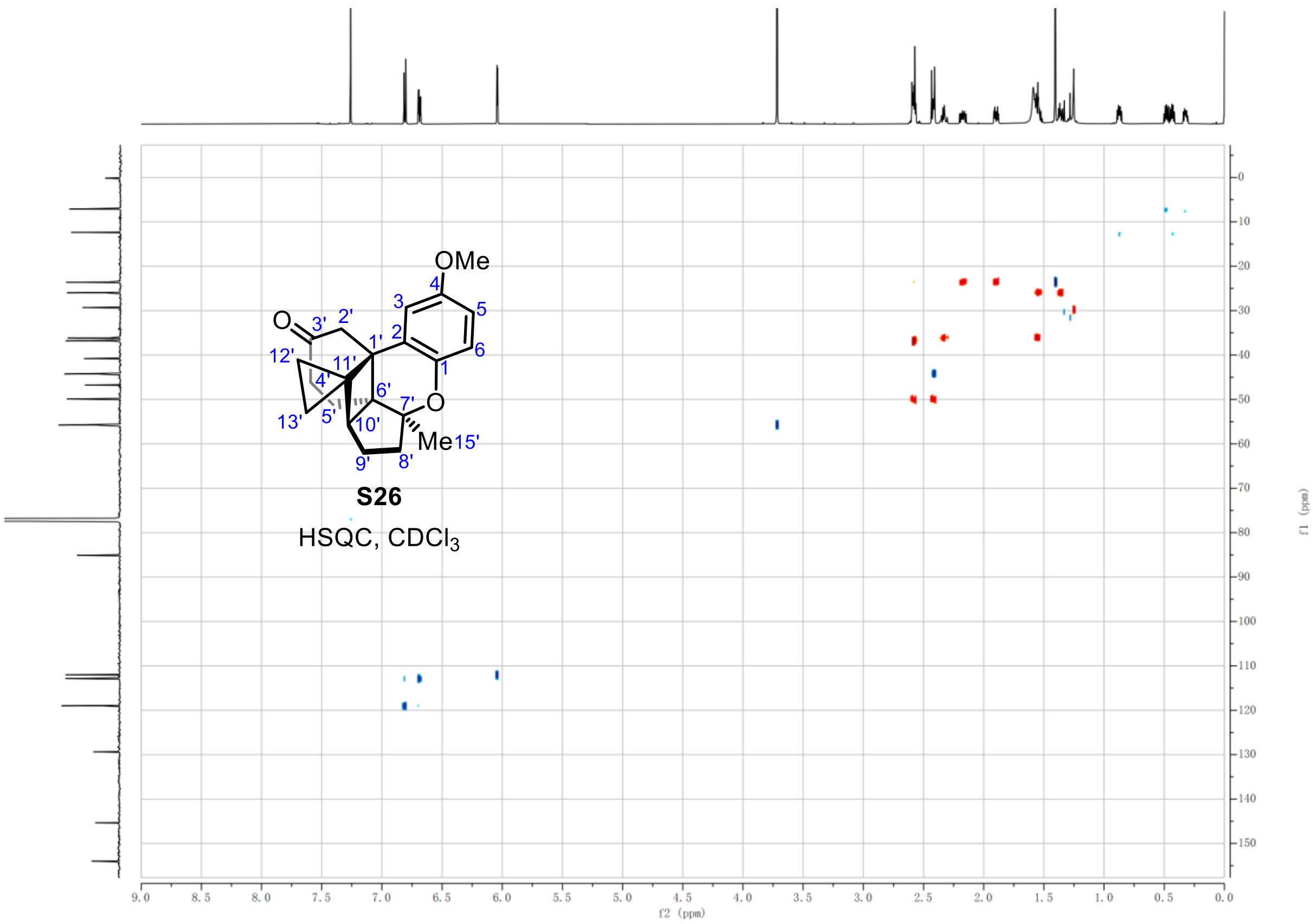

OMe
S26
HSQC, CDCl3
f2 (ppm)
f1 (ppm)

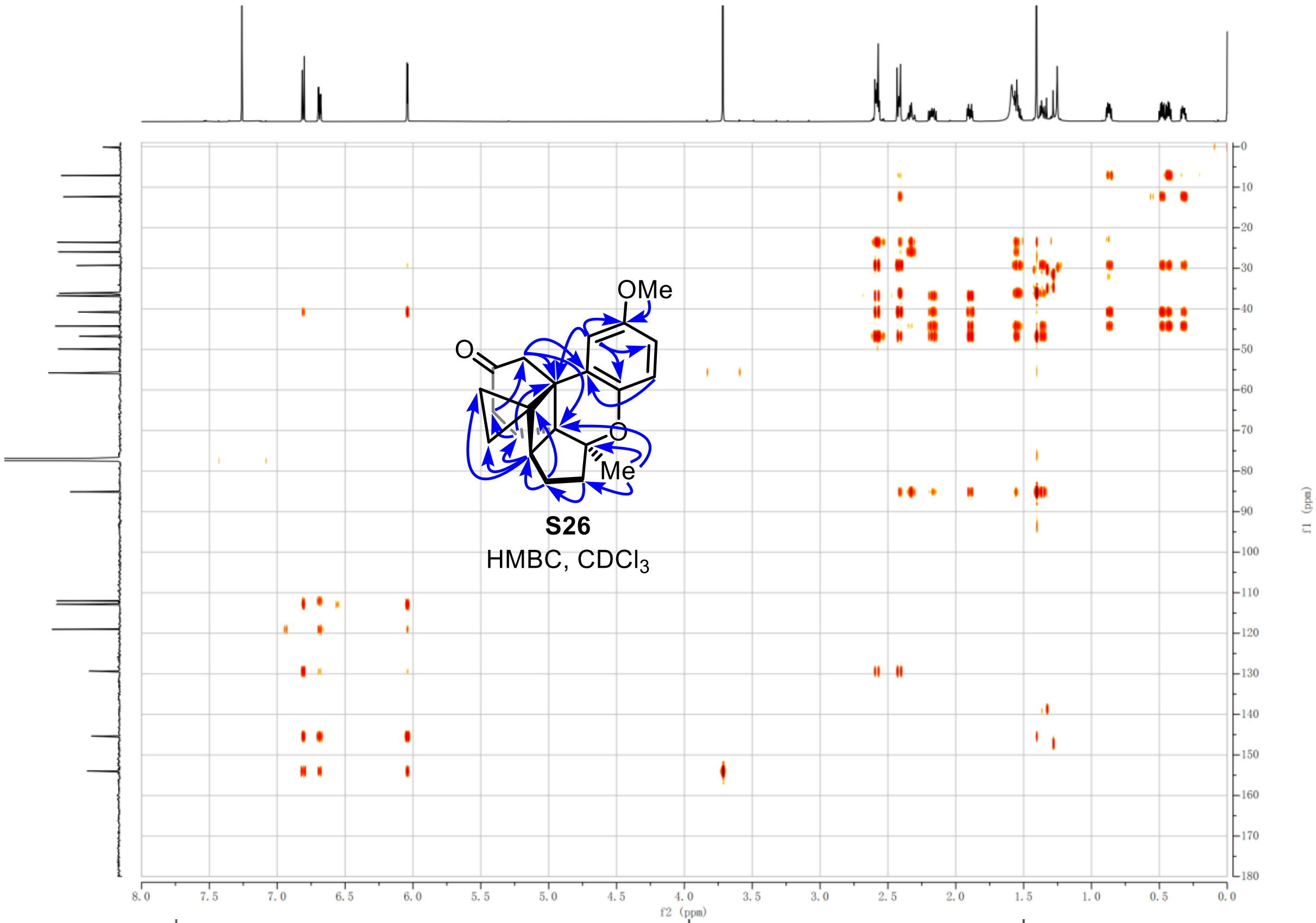
OMe
O
Me
S26
HMBC, CDCl3
f2 (ppm)
f1 (ppm)

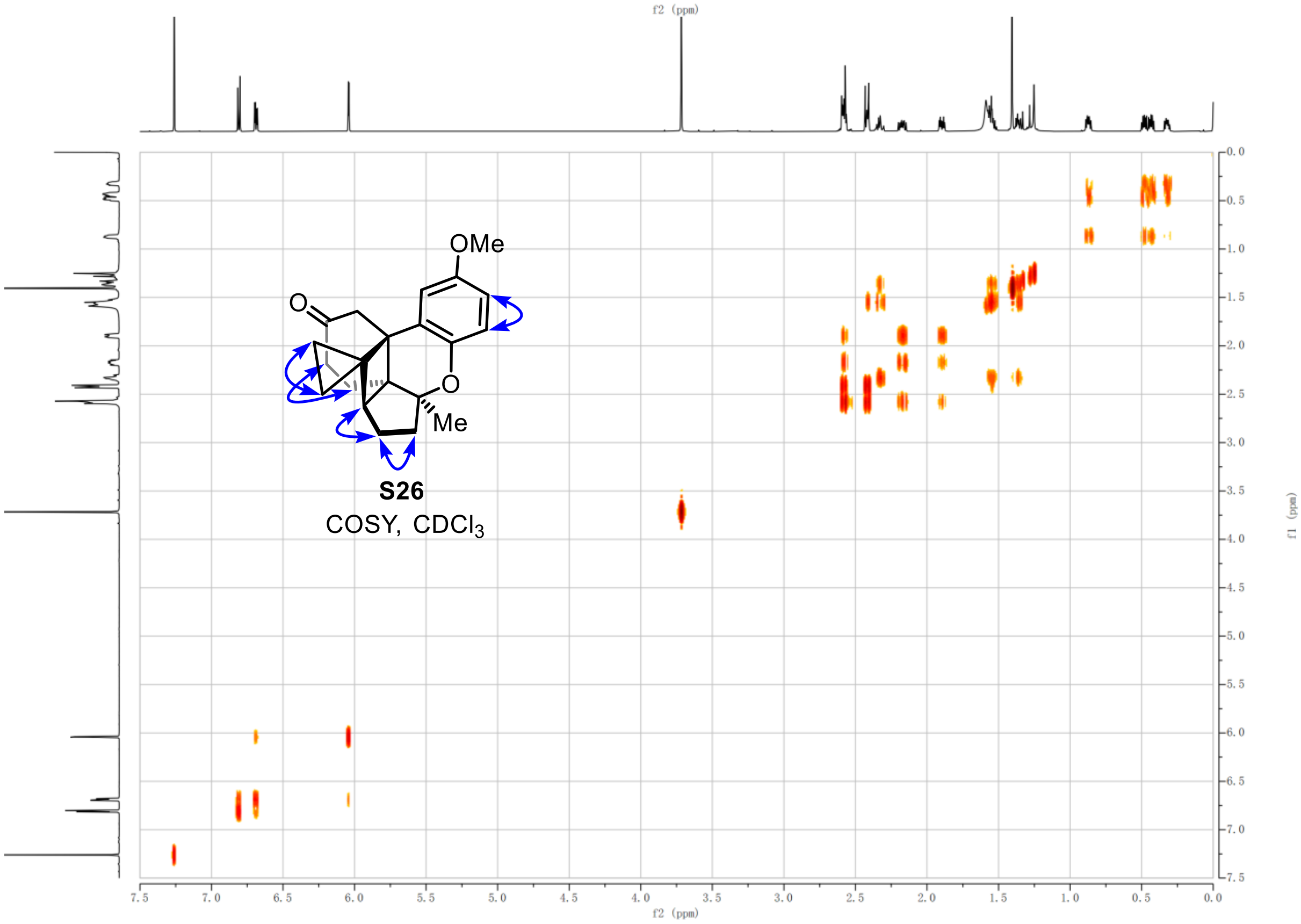
OMe
O
Me
S26
COSY, CDCl3
f2 (ppm)
f1 (ppm)

15. Compound **25**: HRMS data are missing.

◆Thanks for the suggestion. The HRMS data for compound **25** have been included in the revised Supplementary Information: **HRMS**$(ESI)^+$ calcd for $C_{22}H_{28}NaO_2^+$ $[M+Na]^+$: 347.1982; found: 347.1985 (see revised page S29).

16. Cochlearol B: No 2D NMR data are provided. The signal at 2.10–2.07 ppm overlaps with water, making assignment unclear.

◆Thanks for the suggestion. We have provided the 2D NMR spectra of (+)-cochlearol B, with almost all ^{1}H and ^{13}C signals assigned and clearly labeled. The HSQC and HMBC correlations confirm that the signal at 2.10–2.07 ppm (m, overlap, 1H) corresponds to a single proton (H-10′) and the signal at 2.04–1.99 ppm (m, 1H) corresponds to a single proton (H-4′), whereas the signal at 2.07–2.04 ppm contains only the $(CD_3)_2CO$ solvent. All spectra have been incorporated into the revised Supplementary Information (see revised page S79).

Note: we revised the ^{13}C assignments (C2', C6', C11') from the isolation literature (see page S43 of the revised Supplementary Information).

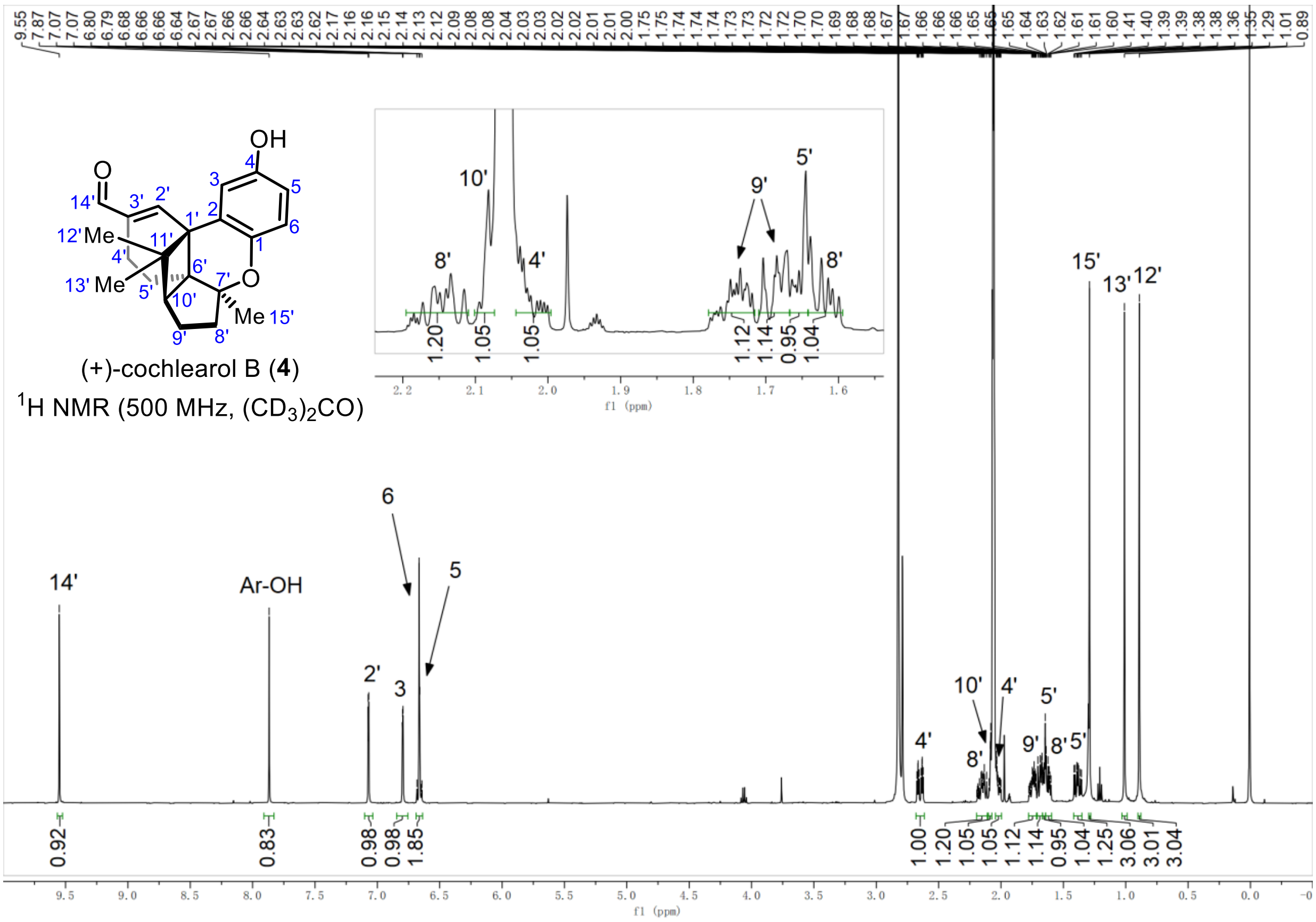

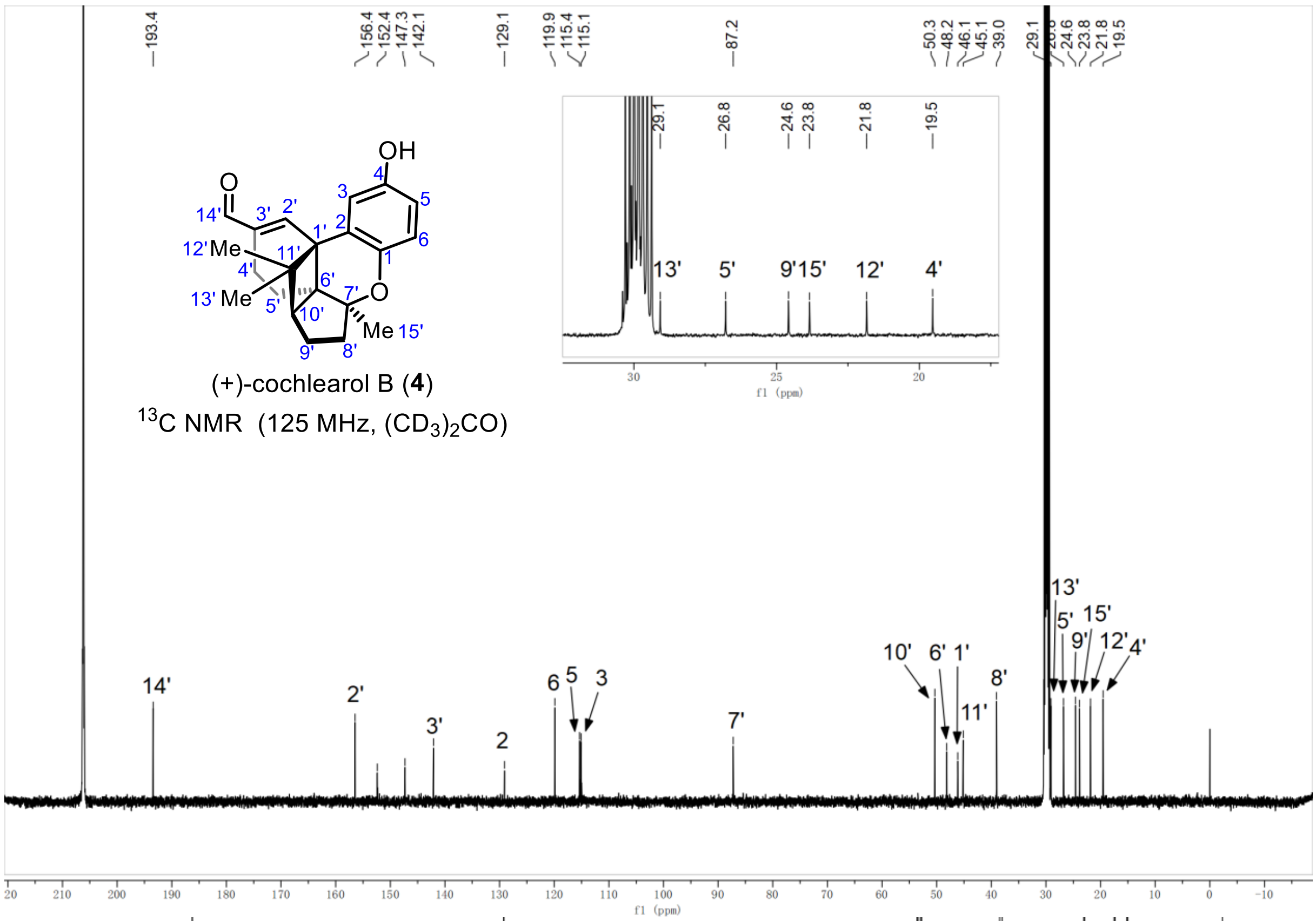
193.4
156.4
152.4
147.3
142.1
129.1
119.9
115.4
115.1
87.2
50.3
48.2
46.1
45.1
39.0
29.1
24.6
23.8
21.8
19.5
29.1
26.8
24.6
23.8
21.8
19.5
13' 5' 9'15' 12' 4'
f1 (ppm)
OH
(+)-cochlearol B (4)
^{13}C NMR (125 MHz, $(CD_3)_2CO$)
14' 2' 3' 2 6 5 3 7' 10' 6' 1' 11' 8' 13' 5' 9' 15' 12' 4'
f1 (ppm)

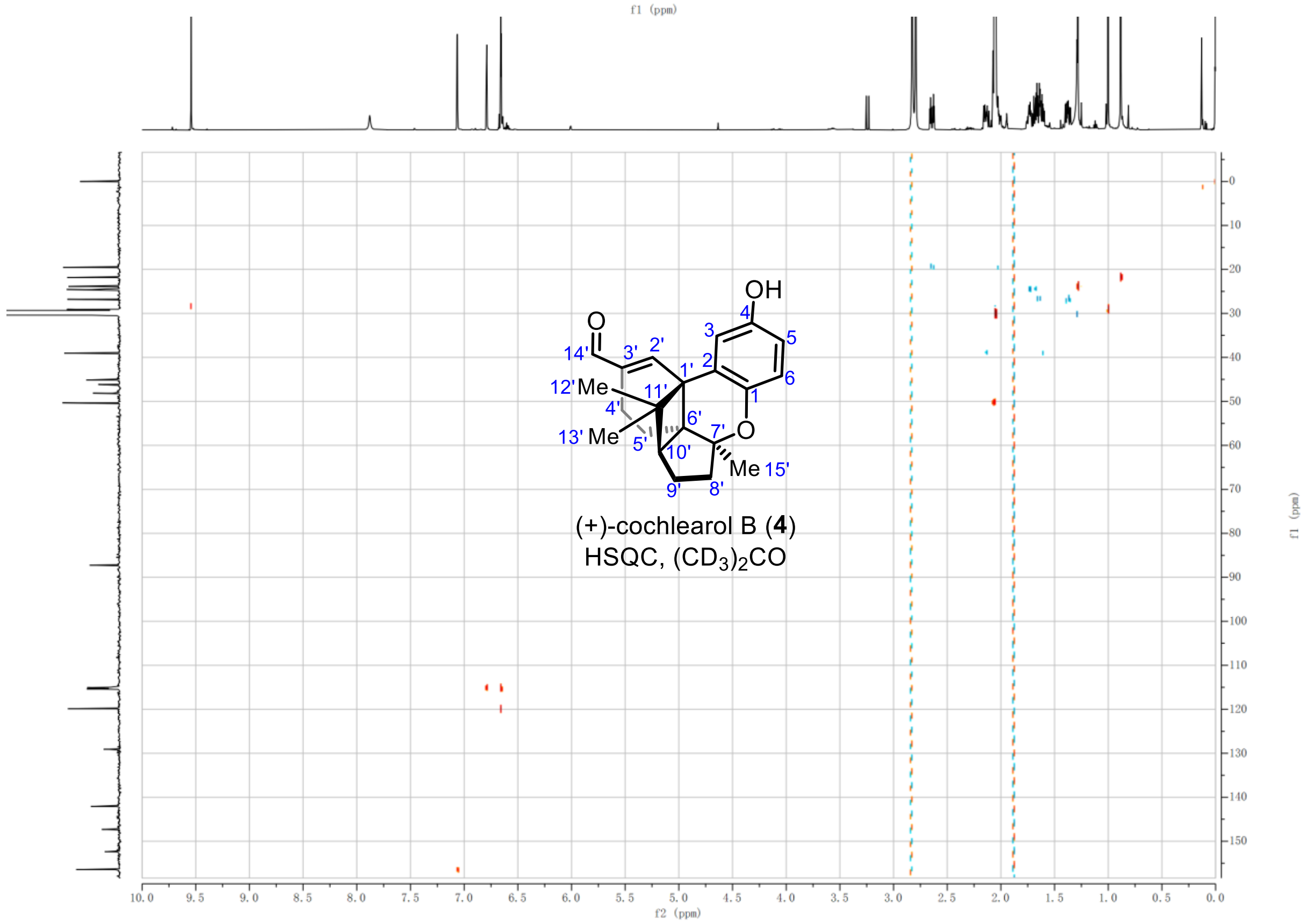
OH
(+)-cochlearol B (4)
HSQC, $(CD_3)_2CO$
f2 (ppm)
f1 (ppm)

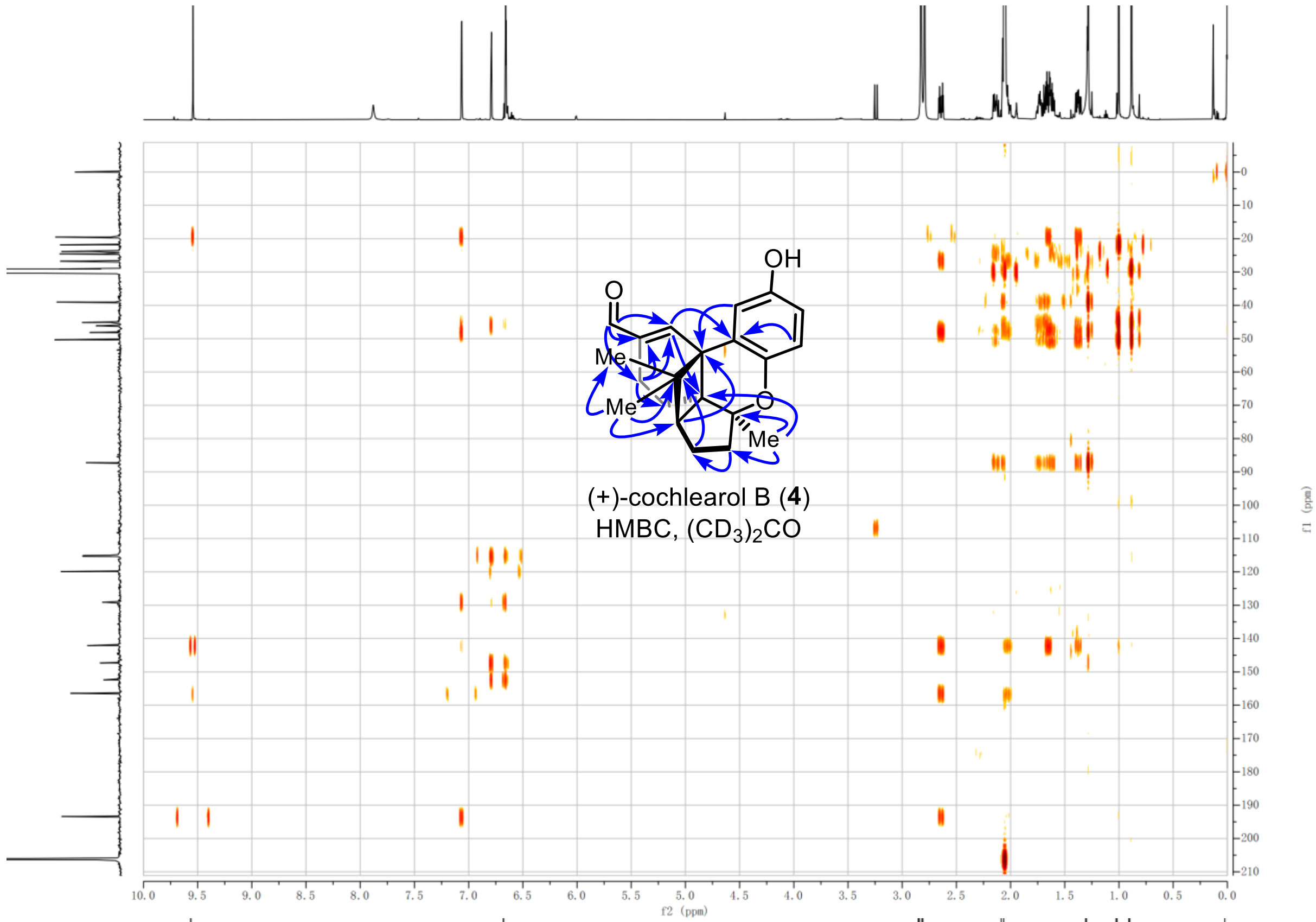

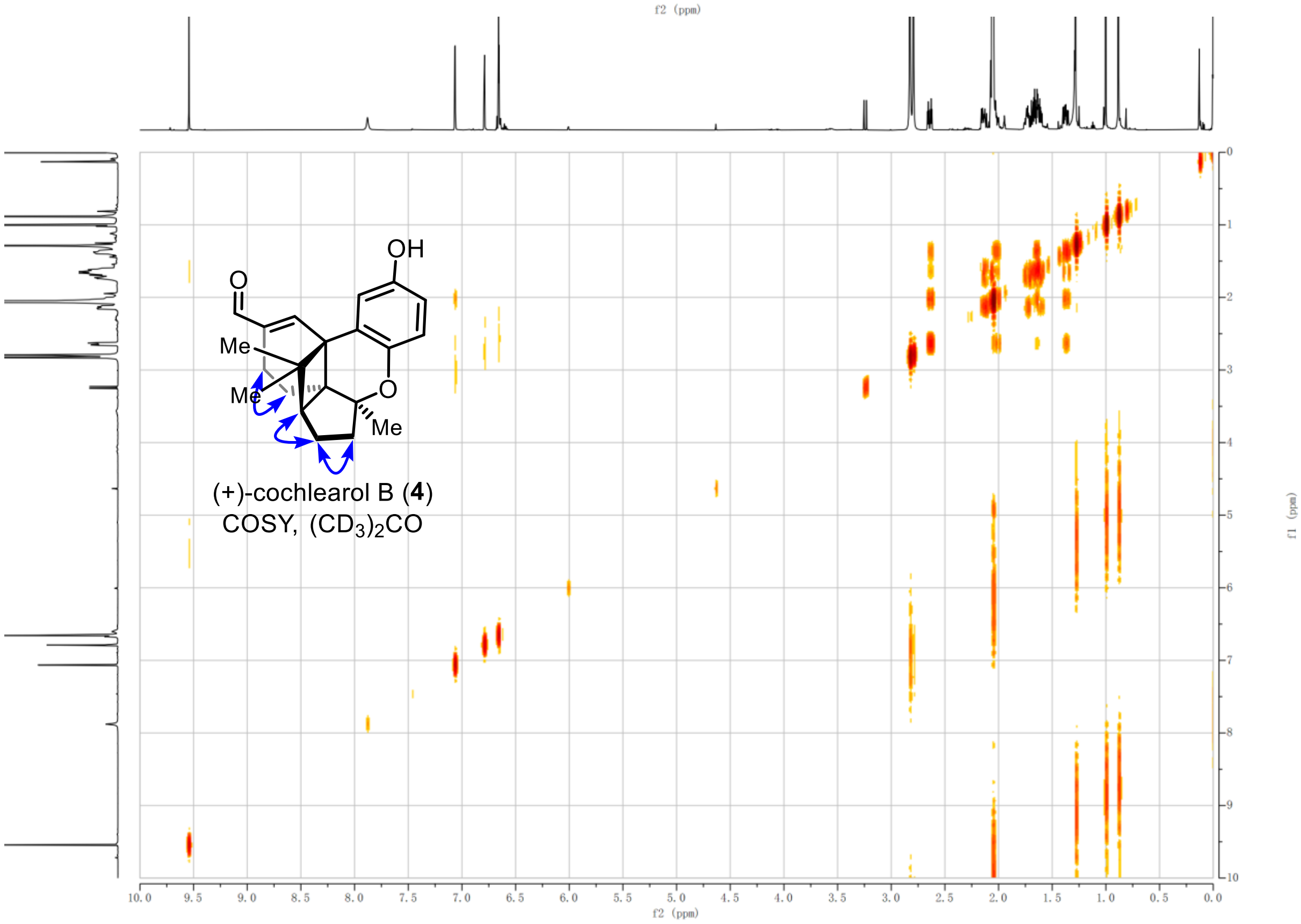

We believe these revisions have significantly strengthened the manuscript and addressed all reviewers' concerns. We are grateful for the opportunity to improve our work and remain available for any further clarifications.

Sincerely yours,

Zhiqiang Ma, Ph.D.
Corresponding Author
